# Gold(III)-Induced Amide Bond Cleavage In Vivo: A Dual Release Strategy via π-Acid Mediated Allyl Substitution

**DOI:** 10.1021/jacs.4c05582

**Published:** 2024-08-08

**Authors:** V. B. Unnikrishnan, Valerio Sabatino, Filipa Amorim, Marta F. Estrada, Claudio D. Navo, Gonzalo Jimenez-Oses, Rita Fior, Gonçalo J. L. Bernardes

**Affiliations:** † Yusuf Hamied Department of Chemistry, University of Cambridge, Lensfield Road, Cambridge CB2 1EW, U.K.; ‡ Champalimaud Centre for the Unknown, Champalimaud Foundation, Lisboa 1400-038, Portugal; § Center for Cooperative Research in Biosciences (CIC bioGune), Building 800, Derio 48160, Spain; ∥ Ikerbasque, Basque Foundation for Sciencep, Bilbao 48013, Spain; ⊥ Instituto de Medicina Molecular João Lobo Antunes, Faculdade de Medicina da Universidade de Lisboa, Av. Prof. Egas Moniz, Lisboa 1649-028, Portugal

## Abstract

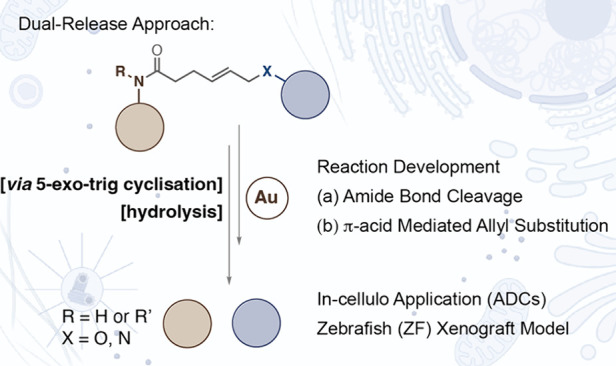

Selective cleavage of amide bonds holds prominent significance by facilitating precise manipulation of biomolecules, with implications spanning from basic research to therapeutic interventions. However, achieving selective cleavage of amide bonds via mild synthetic chemistry routes poses a critical challenge. Here, we report a novel amide bond-cleavage reaction triggered by Na[AuCl_4_] in mild aqueous conditions, where a crucial cyclization step leads to the formation of a 5-membered ring intermediate that rapidly hydrolyses to release the free amine in high yields. Notably, the reaction exhibits remarkable site-specificity to cleave peptide bonds at the C-terminus of allyl-glycine. The strategic introduction of a leaving group at the allyl position facilitated a dual-release approach through π-acid catalyzed substitution. This reaction was employed for the targeted release of the cytotoxic drug monomethyl auristatin E in combination with an antibody-drug conjugate in cancer cells. Finally, Au-mediated prodrug activation was shown in a colorectal zebrafish xenograft model, leading to a significant increase in apoptosis and tumor shrinkage. Our findings reveal a novel metal-based cleavable reaction expanding the utility of Au complexes beyond catalysis to encompass bond-cleavage reactions for cancer therapy.

## Introduction

Bioorthogonal transformations offer profound opportunities to manipulate complex biological processes within living systems.^1−4^ In recent years, bioorthogonal bond-cleavage reactions have offered exciting prospects ranging from gain-of-function studies in proteins^5−7^ to prodrug activation.^8−11^ These reactions carry vast implications, particularly in prodrug activation strategies by reducing the risk of side effects arising from toxicity that limits the maximum administrable dosages of chemotherapeutics.^12−14^ While most approaches utilize terminal caging groups, there is a growing demand for internal cleavable linkers that allow a bifunctional modality for the targeted release of payload.^15,16^ However, the existing chemistry of internal linkers is confined to uncaging a single functional group, limiting their loading capacity and diversity. This restricted dosage likely prevents the whole tumor tissue from being exposed to sufficient drug concentrations, eventually resulting in cancer recurrence and metastasis. Therefore, developing a novel class of reactions that could accommodate bifunctional linkers with dual-release capabilities of diverse functional groups could significantly broaden the therapeutic window of biorthogonal uncaging approaches.

**Figure 1:**
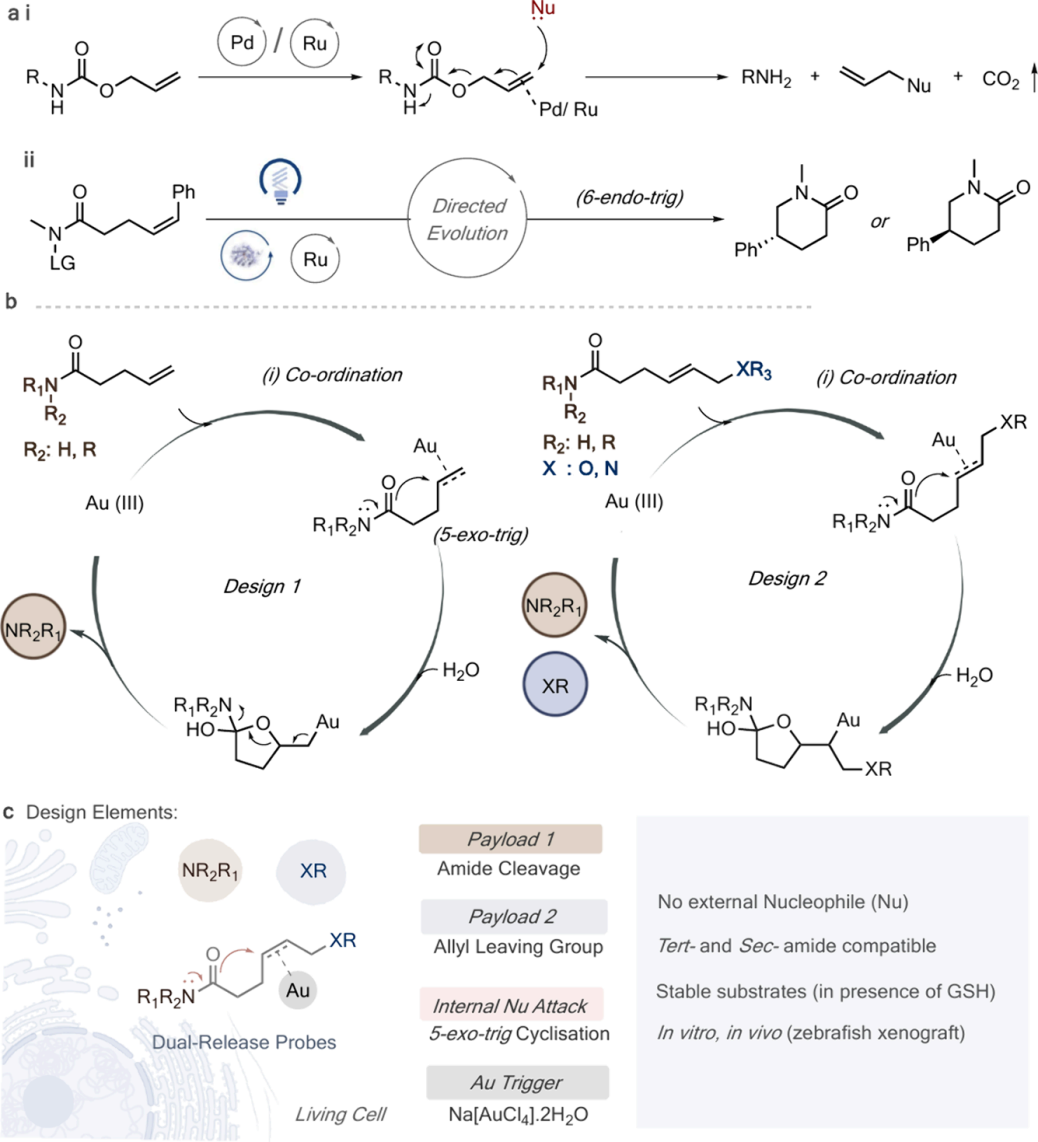
Alkenes as a transition-metal-mediated activating group. (a) (i) Allyl carbamate is a well-studied substrate with palladium (Pd) or ruthenium (Ru) as the catalyst. The activated alkene is attacked by an external nucleophile to eliminate a free amine. (ii) Pentenoic *sec*-amides are shown to form stable cyclized products with Ru photocatalysis. (b) Possibilities for an amide bond cleavage reaction via cyclization mechanism. The imine intermediate can be hydrolyzed after cyclization. Addition of a leaving group at the allyl position allows dual release of substrates possibly via β-elimination. (c) Design elements involved in the dual release strategy.

The promise of controlled prodrug activation has fueled research with functionally relevant and abundant groups like amines or hydroxyls as the preferred groups to be masked. The reactions are triggered by various stimuli, including light,^17−21^ small molecules,^22−27^ or transition metals,^28−34^ facilitating bond cleavage from bioorthogonal protecting groups that deactivate otherwise potent drugs. Notably, transition metal-mediated bond-cleavage reactions have been extensively studied for over a decade because the use of minimal stoichiometry often achieves the desired pharmacologic effect, reducing toxicity and side reactions.^35^ This aspect is further expanded by using metal nanoparticles that can accumulate in tumor cells and operate as catalysts for prodrug activation.^36−40^ Metal triggers also offer higher tissue/cell penetration capacity, higher selectivity toward the substrate, and are less prone to forming reactive oxygen species. The catalytic cycle of the cleavage reaction is often initiated via coordination between metals and electron-rich terminal handles, followed by nucleophilic substitution to break otherwise stable C–N/C–O bonds.^41^ For instance, allyl carbamate is a commonly used caging group for amines. It has been extensively studied with Ru or Pd as triggers, along with excess external nucleophiles such as thiophenol or glutathione (Figure 1a,i).^42−46^ The first deallylation reaction with an organometallic Ru complex led to the intracellular release of Alloc-protected rhodamine in HeLa cells.^47^ The reaction was later extended for a gain-of-function study in proteins by uncaging masked lysine residues with Pd as the trigger.^48^

On the other hand, Pt and Au are known to act as strong π-acid activators with similar reactivity.^49−51^ For instance, the cyclization of pentynoic acid is well-known to proceed quickly in aqueous media, with reaction times ranging from minutes to a few hours.^52−54^ Based on these studies, our group recently devised a reaction whereby an amide carbonyl could be used as an internal nucleophile to cause carbo-cyclization on propargyl handles activated by Pt.^16^ Subsequent hydrolysis of the imine from the cyclized intermediate results in the release of a secondary amine. This reaction initiated a new class of bond cleavage reaction that follows a cyclization mechanism through an intramolecular nucleophilic attack. It also offered an attractive prospect, as amides are stable relative to the corresponding carbamates.

Tanaka and co-workers expanded the scope of the reaction with the use of N-heterocyclic carbene (NHC)-Au(I) complexes to release anticancer drugs upon intramolecular nucleophilic attack of a 2-alkynyl benzamide moiety followed by hydrolysis.^55^ However, the reaction is still limited by its inability to release more than a single payload. Moreover, this reaction does not proceed well with secondary amides, as the amide nitrogen could compete as a nucleophile to yield a stable cyclized product, which results in lower yields of released amine. This increased reactivity was confirmed by Hyster and co-workers, where nitrogen-centered radicals were used to form enzyme-catalyzed intermolecular hydroamination reactions to give high levels of enantioselectivity with directed evolution (Figure 1a,ii).^56^

We hypothesized that the amide bond cleavage reaction might yield benefits if the mechanism is extendable to alkene handles. Alkenes could be derivatized as an allyl-leaving group that can be released through the internal nucleophilic attack (Figure 1b,c). The proposed route would then allow the simultaneous release of two functional groups without the engagement of an exogenous nucleophile. Second, the altered reactivity might allow us to optimize the reaction to accommodate secondary amides. This possibility would expand the scope of the reaction to payloads beyond tertiary amides and can potentially be translated to a genetically incorporable amino acid such as allylglycine.

This work demonstrates that Au(III) can trigger the simultaneous release of two different functional groups in aqueous solutions from pentenoic amides with an allyl-leaving group. The strategic position of an alkene allows the activation of the amide bond as a highly reactive iminium group via *5-exo-trig* cyclization. The reaction proceeds with high yields and rates similar to those of other established uncaging reactions promoted by transition metals. The strategy was successfully applied to small molecule prodrug activation and extended to drug release from an internalizing ADC in cancer cells. Finally, we show that Au(III)-mediated bond cleavage can activate a prodrug in a zebrafish xenograft model for treating colorectal cancer.

## Results and Discussion

### Engineering of a Gold-Triggered Uncaging Reaction

**Figure 2:**
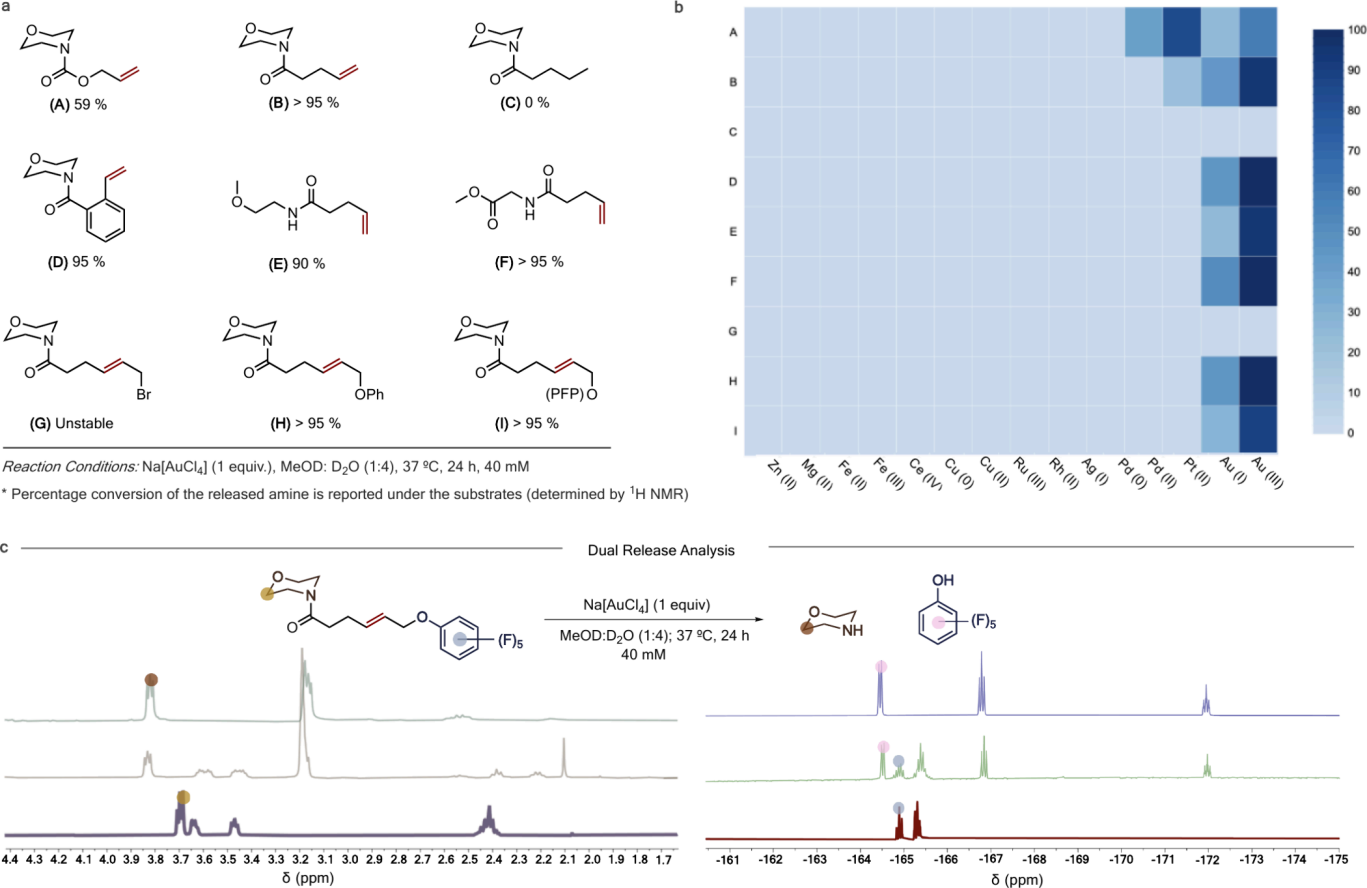
(a) Substrate scope; carbamate (A), tertiary amides (B–D), secondary amides (E, F), and dual-release model substrates (G–I) were used to survey the uncaging reaction. (b) Efficiency of the cleavage reaction under different conditions was assessed by ^1^H NMR spectroscopy (Table SI2; values). Metal complexes used in the study are (Table SI1); Zn(II): ZnSO_4_·7H_2_O, Mg(II): MgCl_2_·6H_2_O, Fe(II): FeSO_4_·7H_2_O, Fe(III): Fe_2_(SO_4_)_3_·9H_2_O, Ce(IV): Ce(NH_4_)_2_(NO_3_)_6_, Cu(I): CuSO_4_·5H_2_O + THPTA, Cu(II): CuSO_4_·5H_2_O, Ru(III): RuCl_3_·3H_2_O, Rh(II): Rh_2_(AcO)_4_, Ag(I): Ag_2_CO_3_, Pd(II): Na_2_[PdCl_4_], Pt(II): K_2_[PtCl_4_], Au(I): AuCl, Au(III): Na[AuCl_4_]. (c) ^1^H NMR spectroscopy for the uncaging of the substrate (I) in the presence of Na[AuCl_4_]. The reaction possibly generates a cyclized intermediate that undergoes hydrolysis to release morpholine. The allyl leaving group allows simultaneous release of PFP. General procedure for determining conversions by ^1^H NMR spectroscopy: substrates and metal salts/complexes were dissolved in MeOD: D_2_O(1:4) at 37 °C. The reactions were transferred to an NMR spectroscopic tube and measured at specific time points (2–24 h). Conversions were calculated based on the relative ratios of methylene peaks resulting from the starting material and the released amine product.

Initially, we created a panel of terminal handles (A–F) (Figure 2a) to screen for metals capable of alkene activation. The variants were constructed with morpholine as the amide leaving group to generate a library. The design makes it possible to monitor the reaction with NMR spectroscopy by following the chemical shift of β-H in the corresponding released amine. Allyl carbamate (Figure 2a, A) was shown to release morpholine by reaction with Na[AuCl_4_] (Au(III)) as assessed by NMR spectroscopy (59% conversion, 24 h) (Figure S1). Most importantly, the reaction proceeded without the addition of any external nucleophiles. K_2_[PtCl_4_] (Pt(II)) also showed much-improved efficiency in cleaving the carbamate bond (86%, 24 h) (Figure S2), while most other metal salts/complexes used in the study showed traces/no desired reaction (Figure 2b, panel A). Interestingly, the uncaging of carbamates with Pt was previously not reported to the best of our knowledge and can be exciting if explored further in targeted drug uncaging applications, considering the chemotherapeutic effects of Pt complexes, such as cisplatin. These results were encouraging enough to proceed with our study on screening metals for a tertiary amide substrate (Figure 2a, **B**). Pt(II) or Au(III) were expected as potential candidates for amide-bond cleavage, considering their ability to activate double bonds as seen with carbamates. As anticipated, Au(III) resulted in the release of secondary amine morpholine (95%, 24 h; Figures S3 and S4), while Pt(II) showed only trace amounts of product. All other metal salts/complexes used in the study resulted in no desired reaction (Figure 2b, panel B). In contrast, if an aliphatic amide (Figure 2a, C) with no alkene handle is used, then no free amine is released for any metal under the same uncaging conditions (Figure 2b, panel C) (Figure S5). The reaction mixture was then screened on a 2-alkenyl benzamide moiety (Figure 2a, D). The reaction proceeds well with Au(III) (95%, 24 h) (Figure 2b, panel D) (Figure S6) and shows that internal modifications within the linker can be tolerated.

Since we also aimed to translate these reactions to amino acids and peptides, it was essential to consider whether the reaction proceeds similarly with secondary amides. Therefore, we tested the efficacy of the reaction on a model secondary amide (Figure 2a, E). Here, it might be possible to have competition from the amide nitrogen as a nucleophile to yield a stable cyclized product. However, the model secondary amide uncaged to release a primary amine with Na[AuCl_4_] (Au(III)) as assessed by NMR (90%, 24 h) (Figure 2b, panel E) (Figure S7). The reaction was also tested with an N-glycine amide (Figure 2a, F) and proceeded with similar efficiency with Au(III) (>95%, 24 h) (Figure 2b, panel F) (Figure S8).

To verify our hypothesis that an allyl leaving group would allow the simultaneous release of two molecules, we expanded our initial library to accommodate internal alkene handles (Figure 2a, G–I). Allyl groups with varying levels of leaving group abilities were synthesized to test the efficacy of the reaction. Interestingly, the substrate with allyl bromide as the leaving group (Figure 2a, G) was seen to be highly unstable under the reaction conditions without any activation by metals (Figure S9). Nevertheless, stable substrates were generated with relatively poorer leaving groups such as phenol (Figure 2a, H) or pentafluorophenol (PFP) (Figure 2a, I). The uncaging of morpholine was neat as observed with both the substrates under standard reaction conditions with Au(III) (>95%, 24 h) (Figure S10). The release of phenol from (H) was difficult to assign as the shift in the peaks was insignificant. However, the release of PFP from (I) were clearly observed through shifts in peaks by ^19^F NMR spectroscopy (Figure 2c) (Figures S11 and S12), establishing simultaneous dual release of different functional groups.

Collectively, these results demonstrate a novel uncaging reaction of stable, protected tertiary, and secondary amides by using Au(III) salts that could function in water and open-air and without the need for high temperatures or external nucleophiles. The reaction proceeds with high conversions with time frames ranging from 2 to 24 h (0.5–1 equiv. Na[AuCl_4_] (Figures S13 and S14).

### Mechanistic and Kinetic Studies of the Uncaging Reaction

**Figure 3:**
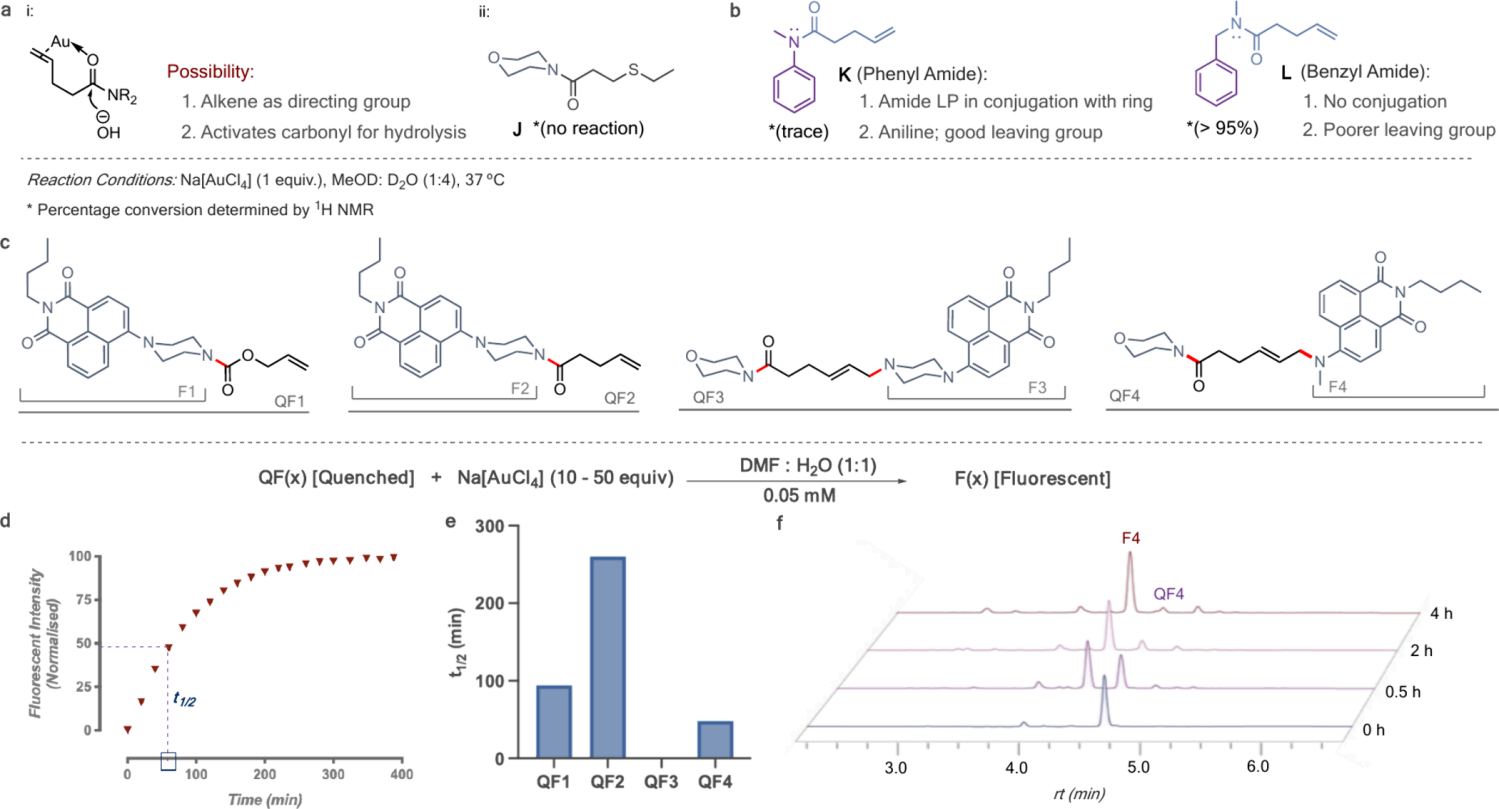
(a) (i) Mechanistic possibility involving an alkene as a directing group for Au(III) to activate a carbonyl group towards hydrolysis. (ii) Model substrate with a thiol directing group failed to release free amine, eliminating the possibility of such a mechanism. (b) Phenyl amide with its lone pair in conjugation with the ring did not show any reaction, while the benzyl amide results in complete release of the free amine, suggesting that the carbonyl group should initially act as a nucleophile through its C–O resonance form. (c) Naphthalimide-based fluorogenic probes (QF1–4) were designed to study the cleavage efficiency of the Au(III) for uncaging alkene-containing molecules. The caged naphthalimide derivatives exhibited high stability in solution, and their quenched fluorescence could be reactivated upon removal of the caging group (λ_ex_ = 445 nm, λ_em_ = 535 nm). (d) Representative example with QF4 demonstrating the increase in fluorescence intensity during the time course of the uncaging reaction with Na[AuCl_4_]. (e) Determined half-time for the uncaging reaction for QF1–4. QF3 showed no increase of fluorescence under standard reaction conditions. (f) HPLC trace showing the formation of F4 from QF4 upon treatment with Na[AuCl_4_] at 37 °C. Time = 0 h is the time point recorded prior to the addition of the Au salt.

The reaction could be argued to proceed, albeit as a minimal possibility, through a mechanism where the alkene acts as a directing group for Au towards the amide carbonyl. Such a directing group effect can, in principle, activate the carbonyl group for external nucleophilic attacks (Figure 3a,i). Although no concrete evidence for carbonyl activation with Au is found in the literature, it might still be important to consider such a mechanistic possibility. Thus, we designed a molecule (Figure 3a,ii, J) with a thiol moiety at the γ-position from the carbonyl. Thiol groups should act as good coordinating groups for Au. However, there was no release of morpholine observed under standard reaction conditions, eliminating the possibility of a mechanism involving a directing group to activate the carbonyl for hydrolysis (Figure S15).

We hypothesized the involvement of two major steps in the reaction mechanism: (i) coordination of the substrate molecule to Au(III), followed by an intramolecular attack of the carbonyl oxygen of the Au-coordinated substrate to the alkene moiety, which gives a five-membered ring intermediate. (ii) Hydration leading to an intermediate that readily decomposes to release free amine. To verify the involvement of a cyclization step, we designed a molecule with a tertiary amide attached to an aromatic system (Figure 3b, K). A conjugated system would reduce the availability of nitrogen lone pair for the intramolecular cyclization step through the carbonyl oxygen. At the same time, *N*-methyl aniline is an excellent leaving group and would speed up the reaction if the amine release was through an alternative mechanism. We also designed benzyl amide (Figure 3b, L) as a positive control where the lone pair is not involved in any conjugation and is readily available for cyclization. Under standard reaction conditions (37 °C in MeOD:D_2_O (1:4)) with stoichiometric amounts of Au(III), the phenyl analogue (K) showed only a trace of the desired reaction. In contrast, the benzyl analogue (L) released the free amine in >95% yield (Figure S16). These results strongly suggest the involvement of the carbonyl group as a nucleophile.

To further study the reaction, carbamate and alkene amide were conjugated to a naphthalimide-based fluorophore to generate fluorescently quenched probes (Figure 3c, QF1–4). Previous studies show that caged naphthalimide derivatives exhibit high stability in solution and cell media. Additionally, their quenched fluorescence could be reactivated with a marked difference upon removing the caging group.

Initially, we screened the ability of Pt(II) to uncage carbamates, as this combination was shown to yield high conversions in our studies using ^1^H NMR spectroscopy. It is known that platinum complexes form a series of reactive intermediates by successive replacement of the chloro-ligands by water or hydroxyl groups. The reaction was monitored by increasing fluorescence upon removing the protecting group to form the fluorescent probe. The fluorescence was restored over a period of 2 h with 50 equiv of preactivated K_2_[PtCl_4_] (Figure S17). We also determined the rate constant of the reaction by fitting the appearance of the fluorophore in the presence of increasing amounts of metal complexes under pseudo-first-order conditions. The reactions were found to have a second-order rate constant of 0.05 M^–1^ s^–1^, in a range comparable to those of previously reported metal-mediated uncaging reactions.

Next, Au(III) was further studied for its ability to uncage amides by conjugating it with naphthalimide-based fluorophores. Initially, we screened an amide substrate with a terminal alkene (QF2). The fluorescence was recovered with a half-life (*t*_1/2_) of 4 h with 50 equiv of Na[AuCl_4_] (Figure S18). We screened internal alkene substrates to verify the dual-release capability of the designed system. Although QF3 was shown to uncage the free amine by LC-MS (Figure S19), the change in fluorescence was scarce possibly due to ineffective quenching of the fluorophore by an allyl group compared to that of a resonance stabilized amide. Hence, we designed QF4 with a masking group directly attached to an aromatic system. Fluorescence was recovered with a *t*_1/2_ of 50 min with 10 equiv of Na[AuCl_4_] (Figure 3d,e). HPLC traces also confirmed the completion of the reaction (QF4, Figure 3f) (Figure S20). We also conducted kinetic experiments with QF4 using varying equivalents of Na[AuCl_4_]. The reaction was found to have a second-order rate constant of 0.21 ± 0.009 M^–1^ s^–1^ (Figure S21). The probe QF4 shows an increase in fluorescence of 28-fold upon removal of the caging group (Figure S22) ideal for in cellulo applications.

To determine the nature of the active species involved in the uncaging reaction, we performed kinetic experiments with carbon disulfide (CS_2_). CS_2_ acts as a catalyst poison for homogeneous and heterogeneous Au(I) reactions, although Au(III) species are unaffected. We observed that under standard reaction conditions, the reaction rates are unaffected with CS_2_ (Figure S23). This result can be attributed to the noninvolvement of Au(I) species in the reaction. However, the reaction was significantly affected by the addition of ethylenediamine tetraacetic acid (EDTA; Figure S24), possibly due to the participation of Au(III) in the reaction. These data are in agreement with our results from ^1^H NMR spectroscopy studies, where Au(I) species only generated a trace amount of product.

### Uncaging Mechanistic Study Using Quantum Mechanical Calculations

**Figure 4:**
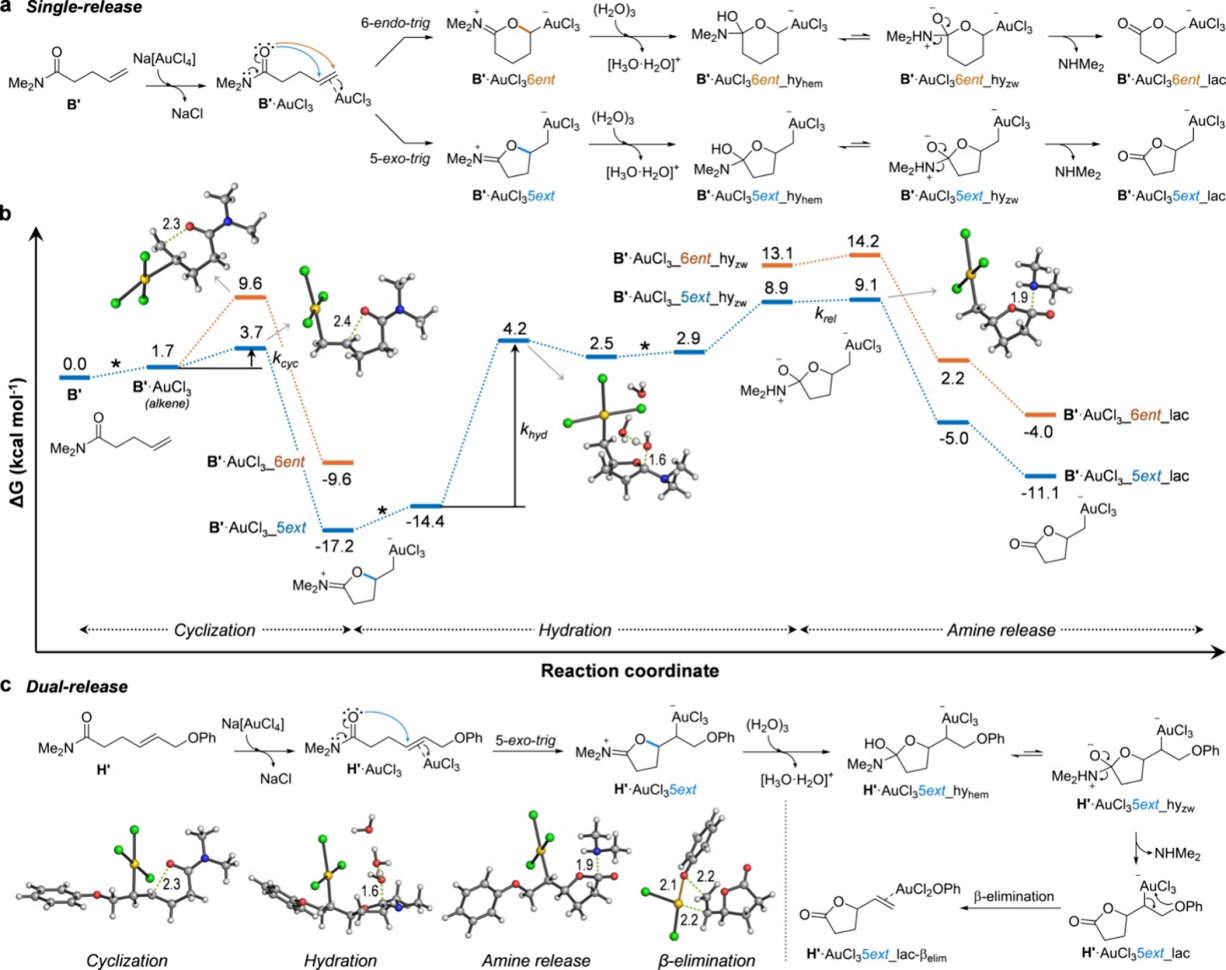
(a) Proposed mechanism for Au(III)-promoted amide cleavage (single-release) from model substrate B′. (b) Minimum energy pathways (MEP) calculated with SMD(H_2_O)/M06/6-311G(d,p)+SDD(Au) for the amide cleavage (single-release) from model substrate B′ catalyzed by Na[AuCl_4_] in water. The complete low-energy 5-*exo*-*trig* cyclization pathway (in blue) was computed, including all postulated intermediates, while only key transition states and intermediates for the more energetic (i.e., less stable) 6-*endo*-*trig* pathway (in orange) were calculated for comparison. The intrinsic activation barriers of the chemically relevant steps are labeled with their associated intrinsic rate constants (*k*_cyc_: cyclization; *k*_hyd_: hydration; *k*_rel_: amide release). When two chiral centers are generated, only the most stable diastereomer is discussed, irrespective of its absolute configuration. Breaking/forming bonds are represented with green dotted lines. Distances are given in angstrom. Free energies are given in units of kcal mol^–1^. Asterisks denote steps in which external species such as Na[AuCl_4_], NaCl, neutral and protonated water clusters, etc., enter or leave the main reaction (see Figure S25 for a more complete depiction); given the intrinsic inaccuracy of calculating the energetics of such hypothetical equilibria, relative energies of charged/neutral species and thus the global thermodynamics of the process should be considered with caution. c, Proposed mechanism for Au(III)-promoted amide and allyl leaving group decaging (dual-release) from model substrate H′ (see Figure S26 for a more complete depiction). The optimized structures of chemically relevant transition states are shown as ball-and-stick models.

Plausible reaction mechanisms for amide cleavage from model substrate B′ either alone (single-release) or including allyl leaving group cleavage from model substrate H**′** (dual-release), were interrogated through quantum mechanical (QM) calculations. (Figure 4; more complete depictions of the mechanisms are available in Figures S25 to S27). Coordination to the carbonyl is highly endergonic by ca. 10 kcal mol^–1^, while coordination of AuCl_3_ to the terminal double bond of B′ is nearly thermoneutral. The nucleophilic intramolecular attack of the amide carbonyl to the highly electrophilic π complex yields a very stable dihydrofuran iminium cation (ca. −17 kcal mol^–1^) via a nearly barrierless 5-*exo*-*trig* cyclization. The alternative 6-*endo*-*trig* cyclization has a ca. 6 kcal mol^–1^ higher activation barrier and yields a much less stable tetrahydropyran iminium cation (ca. −10 kcal mol^–1^). Therefore, such a pathway could be safely discarded, and the complete reaction pathway was computed only from the five-membered intermediate. Of note, a strong σ bond between Au(III) (which is square planar and formally develops a negative charge) and the terminal carbon is formed as a result of the cyclization. The addition of water to the iminium group was calculated to be rate-determining (intrinsic activation barrier of ca. 19 kcal mol^–1^); a cluster of three water molecules was needed to locate a transition state for this hydration step so that the positive charge developed upon nucleophilic attack at the iminium carbon can be effectively delocalized. After the release of a hydrated proton and tautomerization from the resulting hemiaminal, the tertiary amine (NHMe_2_) is finally released in a barrierless and exergonic step, with the concomitant formation of a γ-butyrolactone. Regeneration of the terminal π-alkene-Au group by ring-opening of the lactone to the linear carboxylate is largely thermodynamically unfavored by ca. 34 kcal mol^–1^. For the process to be catalytic when using substoichiometric amounts of Au(III), a final protodeauration step of the σ complex might be required (not calculated). In summary, the amide bond cleavage observed experimentally can be seen as the stepwise hydrolysis of a carbonyl activated as a transient iminium cation, formed by the action of a remote and highly electrophilic alkene-Au group.

Of note, substrate K bearing *N*-methyl aniline shows a very similar energy profile for amide bond cleavage (Supporting Figure SY), with just slightly higher intrinsic activation barriers for the 5-*exo*-*trig* cyclization (ca. 3 kcal mol^–1^) and hydration steps (ca. 18 kcal mol^–1^). Moreover, amine release after hemiaminal tautomerization is spontaneous, barrierless, and even more exergonic than with tertiary alkyl amines, as expected from the better leaving properties of the aniline group. Hence, the origin of the complete lack of reactivity found for this substrate cannot be explained in light of the calculated mechanism.

Regarding dual-release, a very similar mechanism for amide bond cleavage was calculated from model substrate H′ bearing a phenyl ether as the allyl leaving group (Figure S27). In this case, the 6-*endo*-*trig* cyclization transition state is incidentally ca. 2 kcal mol^–1^ more favored than the 5-*exo*-*trig* one due to stabilizing Au−π interactions with the phenyl ring, whereas the rest of the reaction pathway follows the expected trend described above for simpler substrates (i.e., dihydrofuran intermediates being more stable than their tetrahydropyran isomers). In any case, the cyclization step is again calculated to be very fast, and water addition to the tetrahydropyran minimum cation is likewise rate-determining with an intrinsic activation barrier of ca. 18 kcal mol^–1^ followed by the barrierless release of the tertiary amine. Finally, the allyl leaving group could only be cleaved through a β-elimination mechanism; for this, it is necessary to exchange a chloride ligand for the phenyl ether at the gold center. The intrinsic activation barrier for this step (ca. 17 kcal mol^–1^) is comparable to that of iminium hydrolysis; therefore, both release steps are predicted to occur more or less simultaneously and with similar reaction rates, following the rapid cyclization. Of note, all attempts to form a η^3^-allyl π-complex by cleaving the phenoxide anion from the AuCl_3_ σ-complex were unsuccessful. It is presumed that with better leaving groups such as amines (substrates QF3 and QF4), this second release step would be easier, perhaps involving alternative mechanisms.

### Peptide Bond-Cleavage at Allyl Glycine

Next, we focused our efforts to see if the developed reaction could be used to cleave peptide bonds selectively. Recently, Brik and co-workers reported a Au(I)-mediated cleavage of N-propargylated peptide bond under specific conditions.^57^ However, the ability to cleave an amino acid that could be incorporated into a protein would be a massive advancement.

**Figure 5:**
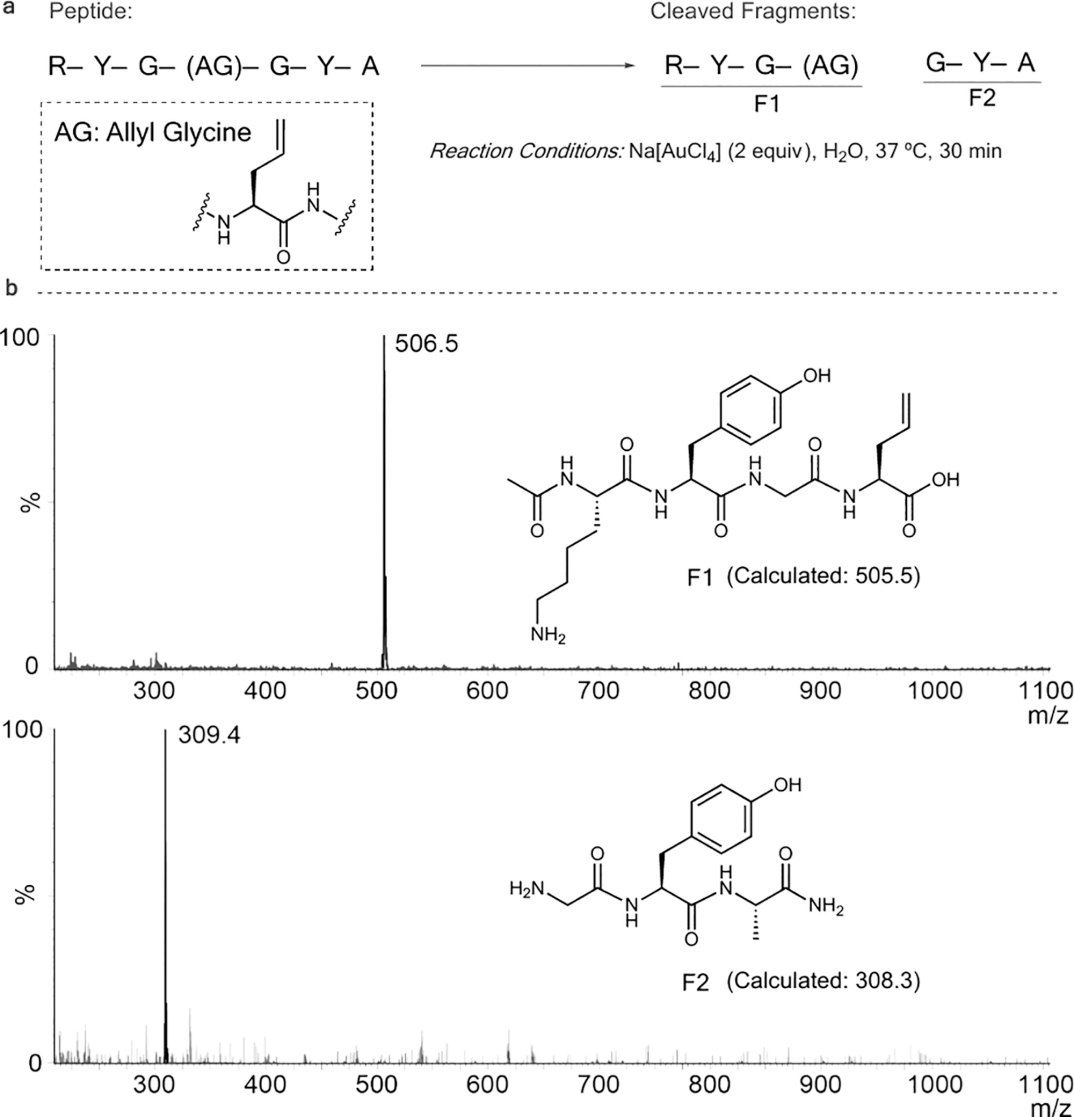
(a) Model peptide (R-Y-G-allyl glycine-G-Y-A) bond-cleavage reaction at allyl glycine with Na[AuCl_4_] at 37 °C in H_2_O. (b) Mass spectrum data that show the fragments after 30 min. The UV-trace indicates full consumption of the starting material to degraded fragments. The full spectrum is provided in Figure S29.

A couple of soluble peptides were designed and synthesized manually on a solid phase to support this possibility. The first peptide consisted of three amino acids (R-Y-G) with a C-terminal glycine protected with the alkene-amide handle (Figure S28). This modification could be a useful strategy for uncaging N-terminal peptides. The second peptide consisted of seven amino acids (R-Y-G-allyl glycine-G-Y-A) with an internal cleavage site (Figure 5a). The reactions were carried out in H_2_O:DMF (1:1) at 37 °C with 2 equiv of Na[AuCl_4_] and analyzed by LC-MS after 30 min. In both cases, we could identify the cleaved products with complete consumption of starting peptides (Figure 5b) (Figure S29).

These findings in principle could expand the applicability of the gold chemistry, beyond uncaging reactions to site-selective cleavage of designed peptide bonds. The formation of a cyclic intermediate is crucial to the success of the peptide cleavage reaction, and adjacent amino acids may influence this process by affecting the rotation around the C–N bond. Indeed, Brik and co-workers demonstrated a higher cleavage efficiency by tuning the residues at the propargyl site.^57^ Additionally, certain conformations in proteins could favor a 5-membered cyclization. While our current study includes a proof of concept for the developed reaction, further optimizations are required to optimize this method for a broader range of peptides and proteins.

### Au-Mediated Uncaging Reaction in Cells

**Figure 6:**
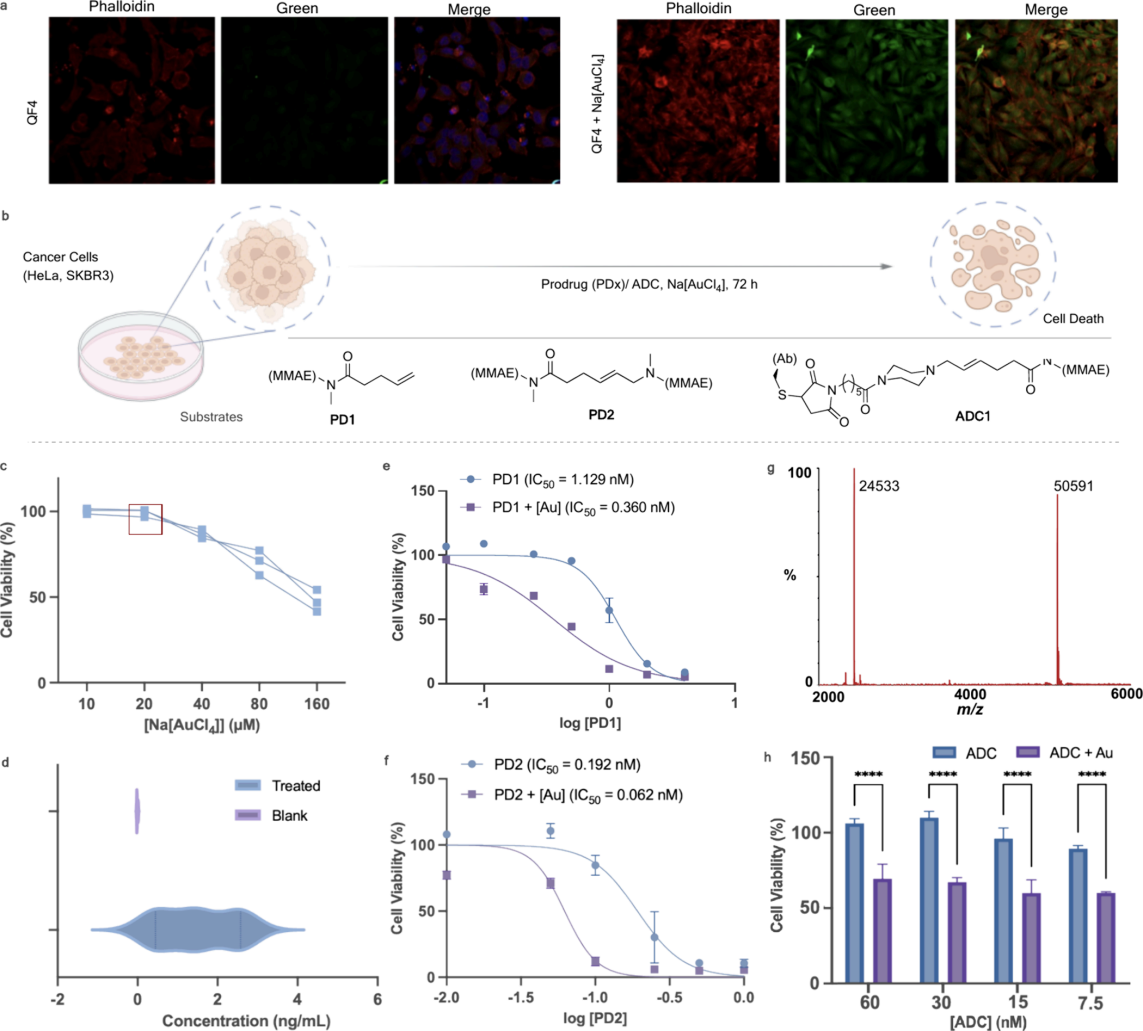
(a) Au(III) uncages the fluorogenic probe QF4 in cells. HeLa cells were exposed to QF4 in medium for 1 h followed by a wash. Cells were randomly distributed into two conditions: DMSO or Au(III) for 12 h. Confocal image of cells upon treatment shows that the progress of the reaction in cells (green channel). (b) Au(III)-mediated uncaging of drugs in cells: Substrates (PD1, PD2, and ADC1) were used in the study. (c) Toxicity screening of Na[AuCl_4_] in HeLa cells. Cells were treated with the depicted concentrations for 72 h, and viability was measured by AlamarBlue. (d) ICP-MS analysis of the cellular extracts revealed the intracellular amount of Au after incubation of Na[AuCl_4_] following several washing steps and lytic treatment. (e, f) HeLa cells were incubated with different concentrations of PD1 or PD2 for 72 h with or without Na[AuCl_4_] (20 μM, twice a day). (g) Au(III)-mediated drug decaging from an ADC; cysteine-selective and irreversible modification of an internalizing antibody thiomab an MMAE conjugating linker. Deconvoluted ESI–MS mass spectrum of the light-chain confirms the modification. (h) Cell viability of SKBR3 cells (HER++) after treatment with ADC1 and subsequent uncaging efficiency upon treatment with 20 μM Na[AuCl_4_], twice daily. Toxicity was determined by the AlamarBlue assay. Error bars represent ± standard deviation (*n* = 3). The statistical significance of the differences between groups was evaluated with the unpaired *t* test. Statistical results: ns >0.05, **P ≤ 0.01, ***P ≤ 0.001, and ****P ≤ 0.0001.

The quenched fluorescent probes (QF2 and QF4) were used to verify if the Au(III) mediated amide bond cleavage reaction would function in cells. We attempted to visualize the reaction by uncaging the quenched fluorophores in HeLa cells. For imaging, HeLa cells were incubated with QF2 or QF4 for 1 h, washed, and then further incubated with dimethyl sulfoxide (DMSO) or Na[AuCl_4_] (10 equiv) for 12 h. The control group displayed nearly no background fluorescence, but the Au(III)-treated group showed increased fluorescence (QF2, Figure S30)(QF4, Figure 6a, Figure S31). This result implies that the quenched fluorophores and Au(III) are both permeable and can react in cells to successfully release the fluorophore.

To verify the dual release, we synthesized a dual release version with a quenched fluorophore and the antineoplastic drug MMAE at two ends. HPLC traces confirm the release of the molecules with the masses verified by LC-MS (Figure S32). Building on these results, we further synthesized a conjugate combining MMAE with MMAF, two chemically distinct drugs. This conjugate also demonstrated successful dual release, showcasing the versatility and robustness of our approach (Figure S33). These findings serve as a proof of concept, highlighting the potential to deliver multiple drugs simultaneously for enhanced therapeutic effects. To simplify our investigation, we proceeded with a pentenoic amide derivative (Figure 6b, PD1)(Figure S34) of the antineoplastic drug MMAE to verify its capability to induce cell death upon activation. MMAE is the drug present in the ADC brentuximab vedotin that is in clinical use to treat patients with relapsed Hodgkin lymphoma and systemic anaplastic large-cell lymphoma and remains the payload of choice for antibody-targeted therapies.^58^ We also synthesized the dual-release substrate (Figure 5b, PD2) of MMAE to compare the efficacies and the possibility of simultaneously releasing two payloads. First, we sought to inspect the maximum tolerable concentrations of Au that could be administered. Na[AuCl_4_] did not significantly influence the viability of HeLa cells at concentrations of up to 20 μM (Figure 6c). Next, the ability of the Au salt to permeate the cells was determined using ICPMS (Figure 6d). The prodrugs (PD1 and PD2; see the SI for synthetic details) were then reacted with Na[AuCl_4_] in a cell culture. With both prodrugs, an increase of about 3-fold in toxicity could be observed for the tested concentrations when reacted with Na[AuCl_4_] over 72 h (Figure 6e,f). Also, the dual-release molecule is seen to have a much higher efficacy at identical concentrations (0.1 nM for PD1 and PD2) (Figures S35 and S36). Potentially, with the dual prodrug (PD2), a given amount of gold can effectively release more drug molecules, leading to a higher overall drug concentration and potency, considering that the availability of Au could be a limiting factor in cells. Additionally, the cell-penetrating abilities of the prodrugs and intermediate stability could also play a significant role in differences in intracellular drug release. It is important to note that for both prodrugs, the addition of Na[AuCl_4_] does not restore their toxicity to the level observed for unmodified MMAE. Although a 3-fold increase in toxicity for the prodrug activation may look modest, it is essential to mention that this is considered relevant given the slow reaction rates possible at the low concentration of Au salts tolerated by cells. It was also important to understand how the substrates respond in the presence of nucleophiles, particularly given their abundance in cellular environments. To investigate this, we initially assessed the stability of PD1 in the presence of excess glutathione, confirming its stability under these conditions (Figure S37a). Subsequently, we conducted incubation experiments in which Na[AuCl_4_] was exposed to a 1:1 ratio of PD1 and glutathione. Remarkably, our observations revealed the exclusive occurrence of the cleavage reaction without any detectable side reactions (Figure S37b). This outcome suggests that the intramolecular cyclization proceeds rapidly and outcompetes any intermolecular reactions. Overall, our data demonstrate that uncaging reactions with Au complexes are possible in cell culture and could release enough of the active drug in cells to induce cell death.

Next, we extended the tertiary amide caging group for chemically controlled drug release from an ADC. The caging group of MMAE was adapted for this purpose because MMAE is a common payload in ADC design. Ideally, Au-cleavable ADC would be stable to cleavage by endogenous extracellular or intracellular conditions. For this reason, we decided to use a maleimide bioconjugation handle coupled to MMAE for antibody modification (Figures S38–S40; SI for synthesis). We then went on and selected the internalizing antibody Trastuzumab for modification, which is specific to the HER2, found overexpressed in tumors. Site-selective conjugation is expected to occur at an engineered cysteine residue at position 205 in each light chain of the antibody, which was termed thiomab, enabling the construction of a chemically defined ADC.^59^ Complete conversion to a homogeneous ADC (Figure 6b, ADC1) was achieved after the reaction of thiomab for 1 h at 37 °C with the maleimide-MMAE drug linker in sodium phosphate buffer at pH 7.4 as assessed by LC–MS (Figure 6g). Notably, the heavy chain remained unmodified, as expected, considering the absence of reactive cysteines in the structure (Figure S40). Finally, we performed the uncaging in cells to release MMAE from the ADC. With SKBR3 (HER2+) as the model, we found ADC1 to be more toxic to cells at submicromolar concentrations in the presence of nontoxic amounts of the Au salt (Figure 6h) (Figure S41). This tertiary amide uncaging reaction should stimulate Au-mediated MMAE delivery from antibodies in the context of targeted cancer therapeutics.

### Uncaging Reaction *In Vivo*

To test the in vivo efficiency of the prodrug, we made use of the Zebrafish (ZF) xenograft model. This model is a fast in vivo platform with resolution to analyze crucial hallmarks of cancer, such as metastatic and angiogenic potentials, but it is also highly sensitive to discriminate differential anticancer therapy responses with single-cell resolution.^60−63^

We first attempted to visualize the reaction by uncaging fluorogenic QF4 in zebrafish embryos. For in vivo imaging, a set of zebrafish embryos were incubated with the probe for 24 h, washed for 1 h in the embryonic medium, and then further incubated with dimethyl sulfoxide (DMSO) or Na[AuCl_4_] for 24 h. QF4 and Na[AuCl_4_] were used at the highest nontoxic concentration to the zebrafish embryos (5 μM of QF4; 15 μM of Na[AuCl_4_]; Figure S42). The control group displays nearly no background fluorescence, while the group treated with Na[AuCl_4_] showed increased fluorescence (Figure S43). This implies that both QF4 and Na[AuCl_4_] are tissue-permeable and are capable of reacting in vivo.

**Figure 7:**
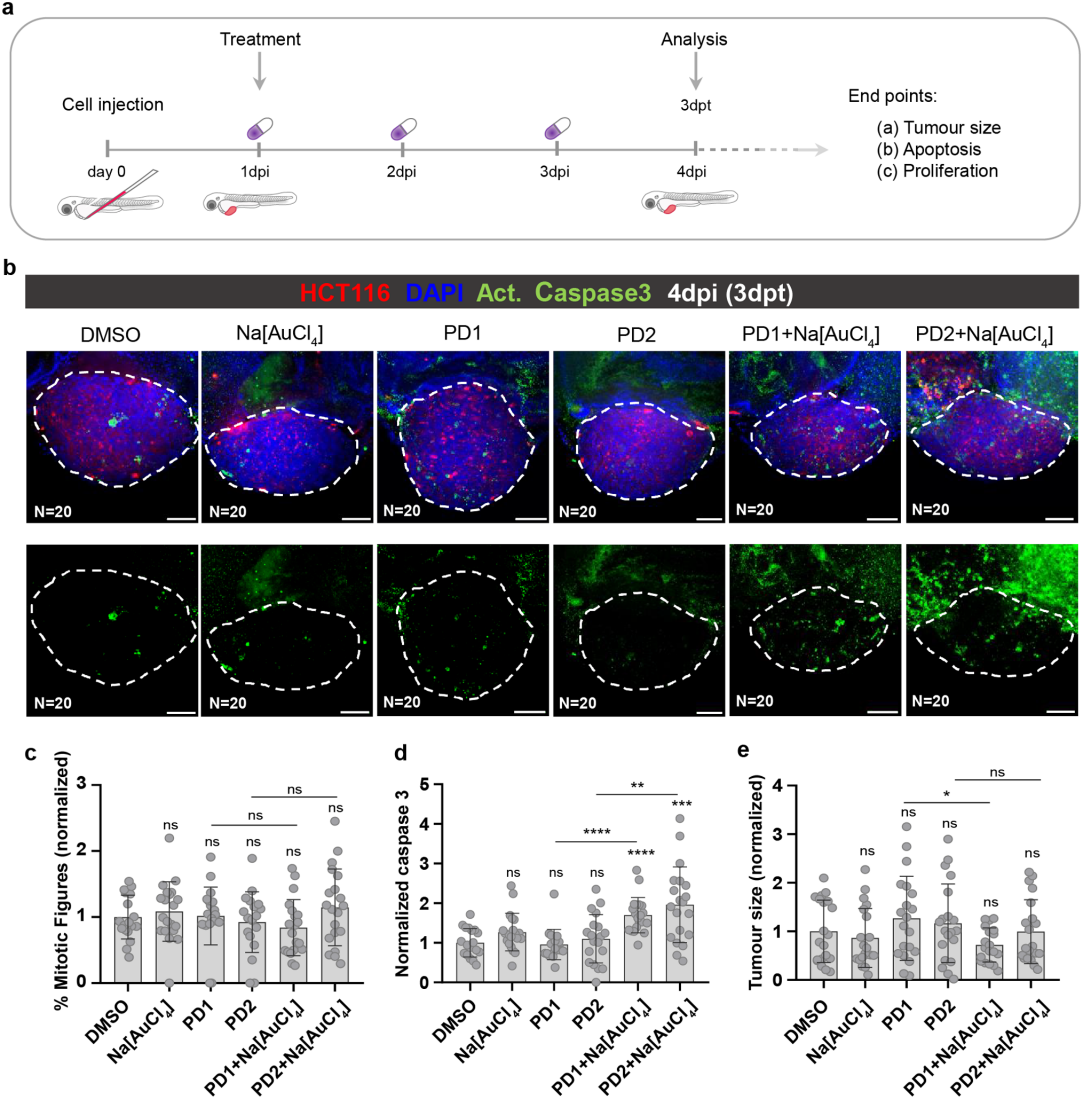
HCT116 human CRC cells were fluorescently labeled with lipophilic CM-DiI (shown in red) and injected into the perivitelline space (PVS) of 2 days post fertilization (dpf) zebrafish larvae. Zebrafish xenografts were randomly distributed into different treatment groups and were daily treated with DMSO, Na[AuCl_4_], PD1, PD2, PD1 + Na[AuCl_4_] and PD2 + Na[AuCl_4_]. At 4 dpi, zebrafish xenografts were analyzed for cell proliferation, apoptosis, and tumor size. (a) Representative scheme of the Zebrafish xenografts assay. (b) Representative maximum projections of Zebrafish xenografts on where the therapeutic effects of the different treatment conditions were analyzed. (c) Quantification of cell proliferation (mitotic figures). (d) Apoptosis (activated caspase 3 ****P < 0.0001, ***P = 0.003, **P = 0.0019); and (e) tumor size (no. of tumor cells: c, *P = 0.0147). Graphs represent fold induction (normalized values to controls) of Avg ± SEM. The number of xenografts analyzed is indicated in the representative images, each dot represents one xenograft, and the results are from two independent experiments. Statistical analysis was performed using an unpaired *t* test. Statistical results: ns >0.05, *P ≤ 0.05, **P ≤ 0.01, ***P ≤ 0.001, and ****P ≤ 0.0001. All images are anterior to the left, posterior to right, dorsal up, and ventral down. The dashed line represents the tumor area. Scale bar 50 μm.

Before measuring the efficacy of Na[AuCl_4_] in decaging prodrugs, we assessed the maximum tolerated concentration for each compound: Na[AuCl_4_], PD1, PD2, Na[AuCl_4_] + PD1, Na[AuCl_4_] + PD2 in noninjected zebrafish beginnings (Figure S42). Then colorectal cancer (CRC) HCT116 zebrafish xenografts were generated as previously described (Figure 7a).^60^ At 24 h post injection (hpi), xenografts were randomly distributed into the different treatment groups: DMSO (vehicle control), Na[AuCl_4_] (15 μM), PD1 (2 nM), PD2 (1 nM), PD1+ Na[AuCl_4_] (2 nM + 15 μM) and PD2+ Na[AuCl_4_] (1 nM + 15 μm).

Zebrafish (ZF) xenografts were analyzed at 4 days post injection (dpi), i.e., 3 days post-treatment (dpt) (Figure 7b). At 3dpt, in the single treatments of Na[AuCl_4_], PD1 or PD2, we could not observe any significant difference regarding the mitotic index, apoptosis, or tumor size (Figure 7c–e). In contrast, when Na[AuCl_4_] was combined with both PD1 and PD2, it was possible to observe a significant fold increase in apoptosis (Figure 7d: DMSO versus PD1 + Na[AuCl_4_] ****P < 0.0001; DMSO versus PD2 + Na[AuCl_4_] ***P = 0.003; PD1 versus PD1 + Na[AuCl_4_] ****P < 0.0001; and PD2 versus PD2 + Na[AuCl_4_] **P = 0.0019). Regarding tumor size, it was possible to observe a significant reduction when Na[AuCl_4_] was combined with PD1, when compared to PD1 alone (Figure 7e: PD1 versus PD1 + Na[AuCl_4_] *P = 0.0147). In general, there is a tendency for tumor shrinkage, which would probably be observed with prolonged treatment days. Nonetheless, the combination of both PD1 and PD2 with Na[AuCl_4_] induced a significant antitumoral effect (apoptosis), showing the effect of Na[AuCl_4_] to activate both prodrugs even *in vivo.*

## Conclusions

In summary, we present a novel uncaging reaction of amide bonds with gold complexes in mammalian cell cultures and living organisms. This reaction was shown to proceed by an intramolecular cyclization mechanism upon activation of an alkene by Au(III), which activates the amide carbonyl toward hydrolysis. The addition of an allyl group allowed the simultaneous release of two different functional groups. This caging strategy was adapted for the synthesis of an ADC, which results in drug release upon treatment with Au(III) salt in cancer cells. The reaction was also adapted and demonstrated to function in a colorectal cancer zebrafish xenograft model with nontoxic amounts of Au(III) to activate a prodrug of anticancer agent MMAE. Furthermore, we also explored the potential of this approach for peptide bond cleavage at allyl-glycine, showcasing its versatility and applicability in various biological contexts.

The work described here represents a significant addition to the toolbox of uncaging strategies for chemical biology applications. Indeed, the Au-mediated cleavage reaction can be accomplished in aqueous systems with high yields and reaction rates. Although the reaction is suitable for drug activation on cells inducing cytotoxicity, the overall yield is partially compromised, possibly owing to the instability of the Au salt by forming bioinorganic complexes. This issue could be improved in the future by developing Au(III)-based nanoparticles, which are known to have reduced toxicity and reach higher payload concentrations. Nevertheless, our findings contribute to the development of novel chemical tools for precision medicine and offer significant implications in chemical biology.

## Supporting Information Available

Detailed methods and characterization, and additional data (PDF)
