# Supplementary material for: Gold(III)-Induced Amide Bond Cleavage In Vivo: A Dual Release Strategy via π-Acid Mediated Allyl Substitution
Source: J Am Chem Soc. 2024 Aug 8;146(33):23240–51. doi: 10.1021/jacs.4c05582 (PMC11345771; doi:10.1021/jacs.4c05582)
Supplement: Supplementary file 1 — ja4c05582_si_001.pdf [file ja4c05582_si_001.pdf]

Supporting Information

Gold (III)-Induced Amide Bond Cleavage In Vivo: A Dual Release Strategy via π-acid Mediated Allyl Substitution

V. B. Unnikrishnan[1], Valerio Sabatino[1], Filipa Amorim[2], Marta F. Estrada[2], Claudio D. Navo[3], Gonzalo Jimenez-Oses[3,4], Rita Fior[2], and Gonçalo J. L. Bernardes[1,5]*

[1] Yusuf Hamied Department of Chemistry, University of Cambridge, Lensfield Road, Cambridge, CB2 1EW, UK.

[2] Champalimaud Centre for the Unknown, Champalimaud Foundation, 1400-038 Lisboa, Portugal.

[3] Center for Cooperative Research in Biosciences (CIC bioGune), Building 800, 48160 Derio, Spain.

[4] Ikerbasque, Basque Foundation for Science, 48013 Bilbao, Spain.

[5] Instituto de Medicina Molecular, Faculdade de Medicina, Universidade de Lisboa, 1649-028 Lisboa, Portugal.

Correspondence should be addressed to G.J.L.B.:

Tel. +44 1223 336305; E-mail: gb453@cam.ac.uk

Table of Contents

1. Materials and Methods **3**

1.1 General Information 3

1.2 Synthetic Procedure and Compound Characterization 5

1.3 NMR and Fluorescence Study Methods 33

1.4 Quantum Mechanical Calculations 34

1.5 Cell Culture Methods 35

1.6 ICP-MS Method 36

1.7 Colon Cancer Zebrafish Methods 37

2. Supporting Data **40**

2.1 ^{1}H NMR Monitoring of Uncaging Reactions 40

2.2 Kinetic and Mechanistic Studies 49

2.3 Quantum Mechanical Calculations 54

2.4 Peptide Bond Cleavage 58

2.5 Cell Studies 60

2.6 Antibody Modification and Uncaging 66

2.7 Colon Cancer Zebrafish Experiments 70

2.8 NMR Spectra 72

2.9 Quantum Mechanical Calculations (Additional Data) 86

3. References **102**

1. Materials and Methods

1.1. General Information

Solvents, Reagents and Materials | All reactions were carried out under a positive pressure of nitrogen unless otherwise stated. Oven dried glassware was heated to 140 ºC and cooled under high vacuum. All reagents were purchased from commercial sources and were used as received. DCM, MeOH, Et_2O and toluene were freshly distilled over CaH_2 under an atmosphere of argon. THF was pre-dried over CaH_2 and then distilled over $LiAlH_4/Ph_3CH$. Dry DMF was obtained from commercial sources and used directly. DIPEA and TEA were distilled over CaH_2 and stored under argon over 4 Å mol. sieves. Unless otherwise stated, water for HPLC or LC-MS was HPLC grade and water for synthesis was de-ionised. Flash column chromatography was performed on Material Harvest 60 Å silica. Analytical TLC was visualized by UV at 254 nm, ninhydrin or $KMnO_4$ stain as appropriate.

Characterization | NMR spectra were recorded on Bruker 400-AVIII, DPX-400 or 500-AVIII HD Smart Probe as appropriate. The residual solvent peaks were used as an internal reference for chemical shift (^{1}H-NMR $CDCl_3$ δ 7.27 ppm, D_2O δ 4.79 ppm, MeOD δ 3.31 ppm; ^{13}C-NMR $CDCl_3$ δ 77.0 ppm). Data are presented as follows: chemical shift (ppm), multiplicity (br. = broad, s = singlet, d = doublet, t = triplet, q = quartet, hept = heptet, m = multiplet), coupling constant *J*, and integration. High resolution mass spectra were obtained with a Thermo Fischer Orbitrap or Waters Xevo LC-MS and ionized by electrospray (ESI). Absorbance and fluorescence spectra or endpoints were measured in a BMG Clariostar in Greiner 300 µL 96-well, U-bottom, clear polypropylene plates.

High Performance-Liquid Chromatography | HPLC was performed on a HPLC: Agilent Infinity 1260 II system with a semi-preparative Column YMC-Triart C18, 250 x 10 mmI.D., S-5 µm, 12 nm, TA12S05-2510WT. Crude product was dissolved in 1:1 MeCN/H_2O and sterile filtered at 0.2 µm with EMD Millipore™ polyethersulfone syringe filters. 1 mL crude product aliquots were injected into the column and flow was maintained at 8.0 mL min^{-1} with solvent mixtures of MeCN/H_2O + 0.1% TFA. UV-vis absorbance was monitored at 210 and 254 nm to indicate when to begin manual collection of fractions. MeCN was evaporated from the fractions under a flow of compressed air, and the remaining H_2O/TFA solution flash frozen in liquid nitrogen then lyophilised.

Liquid Chromatography-Mass Spectrometry method for analysis of protein conjugation | LC-MS was performed on a Waters Acquiry UPLC system equipped with a single quadrupole mass detector using an Acquity UPLC protein BEH C4 column, 300 Å, (1.7 mm, 2.1 × 50 mm). Solvents A (H_2O with 0.01% formic acid) and B (71% MeCN, 29% H_2O and 0.075% formic acid) were used as the mobile phase at a flow rate of 0.2 mL min^{-1}. The gradient used was: 0-12 min 28-71.2% A/B, 12-13 min 71.2-100% A/B, 13-16 min 100% A/B, 16-16.5 min 100%-28% A/B, 16.5-20 min 28% A/B. The electrospray source was operated with a capillary voltage of 3.0 kV and a cone voltage of 20 V. Nitrogen was used as the desolvation gas at a total flow of 800 L h^{-1} with cone volume at 1 L h^{-1}. Desolvation temperature was set to 400 ºC. Total mass spectra were reconstructed from the ion series using the MaxEnt algorithm preinstalled on MassLynx software (version 4.1 from Waters) according to the manufacturer's instructions. To obtain the ion series described, the major protein peak(s) of the chromatogram were selected for integration and further analysis.

1.2. Synthetic Procedures and Compound Characterization

Allyl morpholine-4-carboxylate (A)[1]

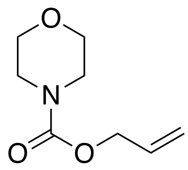

DIPEA (600 µL, 3 equiv, 3.44 mmol) was dissolved in freshly distilled DCM (10 mL) in an oven dried flask and allyl chloroformate (122 µL, 1 equiv, 1.15 mmol) was added, followed by dropwise addition of morpholine (100.0 µL, 1 equiv, 1.15 mmol) at room temperature. After 6 h, the reaction was diluted with 3 M HCl (20 mL) and extracted with DCM (3 x 10 mL). The combined organic phases were dried over $MgSO_4$ and evaporated *in vacuo*. The crude material was purified by flash chromatography with 30% EtOAc/petrol on silica (50 g) to give the product (120 mg, 701 µmol, 61.1%) as a colourless liquid.

R_f (80% EtOAc/ Hex) 0.45

^{1}H NMR (400 MHz, MeOD) δ 5.98 (ddt, *J* = 17.3, 10.7, 5.5 Hz, 1H), 5.32 (dq, *J* = 17.3, 1.7 Hz, 1H), 5.23 (dq, *J* = 10.5, 1.4 Hz, 1H), 4.61 (dt, *J* = 5.5, 1.5 Hz, 2H), 3.72 – 3.43 (m, 8H).

^{13}C-NMR δ 155.1, 132.9, 117.6, 66.5, 66.2, 44.3, 43.9.

ESI-MS: calcd. for $C_8H_{13}NO_3$ $[M+H]^+$ m/z 171.1, found 171.0

1-Morpholinopent-4-en-1-one (B)[2]

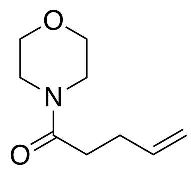

DIPEA (2.10 mL, 3 equiv., 12.1 mmol) was dissolved in freshly distilled DCM (10 mL) in an oven dried flask and pent-4-enoic anhydride (0.734 mL, 1 equiv., 4.02 mmol) was added, followed by dropwise morpholine (0.347 mL, 1.0 equiv., 4.02 mmol) at room temperature. After 6 h, the reaction was diluted with 3 M HCl (20 mL) and extracted with DCM (3 x 10 mL). The combined organic phases were dried over $MgSO_4$ and evaporated *in vacuo*. The crude material was purified by flash chromatography with 30% EtOAc/petrol on silica (50 g) to give the product (**2**) (350 mg, 2.07 mmol, 51.5%) as a colourless liquid.

R_f (80% EtOAc/ Hex) 0.4

^{1}H NMR (400 MHz, $CDCl_3$) δ 5.83 (dddd, J = 20.1, 10.4, 5.3, 2.5 Hz, 1H), 5.15 – 4.89 (m, 2H), 3.78 – 3.34 (m, 8H), 2.38 (d, J = 3.1 Hz, 4H).

^{13}C-NMR (101 MHz, $CDCl_3$) δ 155.1, 132.9, 117.6, 66.5, 66.2, 44.3, 43.9.

ESI-MS: calcd. for $C_9H_{15}NO_2$ $[M+H]^+$ m/z 169.1, found 169.0

1-Morpholinopentan-1-one (C)[3]

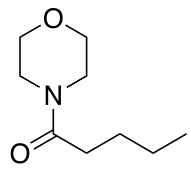

DIPEA (1.5 mL, 1.5 equiv., 8.6 mmol) was dissolved in freshly distilled ACN (10 mL) in an oven dried flask and pentanoic acid (0.94 mL, 1.5 equiv., 8.6 mmol) was added, followed by the addition of HATU (3.2 g, 1.5 equiv., 8.6 mmol). After 15 minutes at room temperature, morpholine was added dropwise and allowed to stir for 6 h. The reaction was diluted with 3 M HCl (20 mL) and extracted with EtOAc (3 x 25 mL). The combined organic phases were dried over $MgSO_4$ and evaporated *in vacuo*. The crude material was purified by flash chromatography with 20% EtOAc/ Hexane on silica (60 g) to give the product (690 mg, 4 mmol, 70%) as a colourless liquid.

R_f (60% EtOAc/ Hex) 0.5

^{1}H NMR (400 MHz, $CDCl_3$) δ 3.70 – 3.39 (m, 8H), 2.36 – 2.26 (m, 2H), 1.67 – 1.55 (m, 2H), 1.36 (dq, *J* = 14.7, 7.4 Hz, 2H), 0.92 (t, *J* = 7.3 Hz, 3H).

^{13}C-NMR (101 MHz, $CDCl_3$) δ δ 171.9, 66.9, 66.7, 46.1, 41.8, 33.4, 32.8, 27.37, 26.9, 22.6, 22.2, 13.9, 13.7.

ESI-MS: calcd. for $C_9H_{17}NO_2$ $[M+H]^+$ m/z 171.1, found 172.2

Morpholino(2-vinylphenyl)methanone (D)

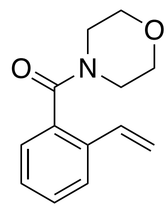

DIPEA (0.176 mL, 1.5 equiv., 1.01 mmol) was dissolved in freshly distilled ACN (10 mL) in an oven dried flask and 2-vinylbenzoic acid (0.1 g, 1.0 equiv., 0.675 mmol) was added, followed by the addition of HATU (0.385 g, 1.5 equiv., 1.01 mmol). After 15 minutes at room temperature, morpholine (0.1 mL, 1.5 equiv., 1.01 mmol) was added dropwise and allowed to stir for 6 h. The reaction was diluted with 3 M HCl (20 mL) and extracted with EtOAc (3 x 25 mL). The combined organic phases were dried over $MgSO_4$ and evaporated *in vacuo*. The crude material was purified by flash chromatography with 60% EtOAc/ Hexane on silica (60 g) to give the product (90 mg mg, 0.675 mmol, 61%) as a colourless oil.

R_f (80% EtOAc/ Hex) 0.40

^{1}H NMR (500 MHz, $CDCl_3$) δ 7.58 (d, *J* = 7.8 Hz, 1H), 7.37 (td, *J* = 7.6, 1.5 Hz, 1H), 7.30 (td, *J* = 7.5, 1.2 Hz, 1H), 7.22 (dd, *J* = 7.6, 1.5 Hz, 1H), 6.75 (dd, *J* = 17.4, 11.0 Hz, 1H), 5.76 (d, *J* = 17.5 Hz, 1H), 5.35 (d, *J* = 11.0 Hz, 1H), 3.91 – 3.72 (m, 4H), 3.60 – 3.49 (m, 2H), 3.23 – 3.14 (m, 2H).

^{13}C-NMR (126 MHz, $CDCl_3$) δ 169.6, 134.6, 134.4, 133.5, 129.3, 128.0, 126.4, 125.6, 116.9, 77.6, 77.3, 77.2, 77.0, 76.8, 66.9, 66.9, 47.3, 42.0.

ESI-MS: calcd. for $C_{13}H_{15}NO_2$ $[M+H]^+$ m/z 217.2, found 218.2

***N*-(2-methoxyethyl)pent-4-enamide (E)**[4]

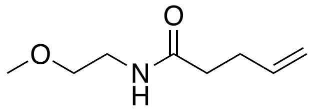

4-pentenoic acid (448 µL, 1.1 equiv., 4.39 mmol) was dissolved in DCM (10 mL) and DIPEA (2.09 mL, 3 equiv., 12.0 mmol) was added followed by EDC·HCl (842 mg, 1.1 equiv., 4.39 mmol) at room temperature. The reaction was stirred for 1 h before 2-methoxyethylamine (347 µL, 1.0 equiv., 4 mmol) was added dropwise. The reaction was stirred for a further 6 h before diluting with 3 M HCl (20 mL) and extracting with DCM (3 x 10 mL). The combined organic phases were dried over $MgSO_4$, filtered, and evaporated in vacuo. The crude material was purified by flash column chromatography with 70% EtOAc/Hex on silica to give the desired product (250.0 mg, 1.59 mmol, 39.8%) as a clear oil.

R_f (80% EtOAc/ Hex) 0.3

^{1}H NMR (400 MHz, $CDCl_3$) δ 5.83 – 5.73 (m, 1H), 5.06 – 4.93 (m, 2H), 3.41 (t, J = 1.8 Hz, 4H), 3.32 (d, J = 1.1 Hz, 3H), 2.36 (qt, J = 6.6, 3.3 Hz, 2H), 2.29 – 2.21 (m, 2H).

^{13}C-NMR (101 MHz, $CDCl_3$) δ 172.3, 137.0, 115.5, 71.2, 58.7, 39.1, 35.8, 29.6.

ESI-MS: calcd. for $C_8H_{15}NO_2$ $[M+H]^+$ m/z 157.1, found 157.2

methyl pent-4-enoylglycinate (F)

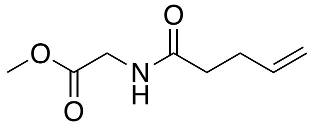

Methyl glycinate (300 mg, 1 equiv., 3.37 mmol) was dissolved in freshly distilled DCM (10 mL) in an oven dried flask and DIPEA (2.93 mL, 5 equiv., 16.8 mmol) was added, followed by dropwise pent-4-enoic anhydride (615 µL, 1 equiv., 3.37 mmol) at room temperature. After 6 h, the reaction was diluted with 3 M HCl (20 mL) and extracted with DCM (3 x 10 mL). The combined organic phases were dried over $MgSO_4$ and evaporated *in vacuo*. The crude material was purified by flash chromatography with 30% EtOAc/petrol on silica (50 g) to give the product (300 mg, 1.75 mmol, 52%) as a colourless liquid.

R_f (80% EtOAc/ Hex) 0.44

^{1}H NMR (400 MHz, $CDCl_3$) δ 5.81 (ddt, J = 16.8, 11.1, 6.3 Hz, 1H), 5.11 – 4.93 (m, 2H), 4.02 (d, J = 4.9 Hz, 2H), 3.73 (d, J = 1.4 Hz, 3H), 2.46 – 2.25 (m, 4H).

^{13}C-NMR (101 MHz, $CDCl_3$) δ 172.4, 170.5, 136.8, 115.7, 52.4, 41.2, 35.5, 29.4.

HR-MS (ESI): calcd. for $C_8H_{13}NO_3$ $[M+H]^+$ m/z 171.0968, found 171.0970

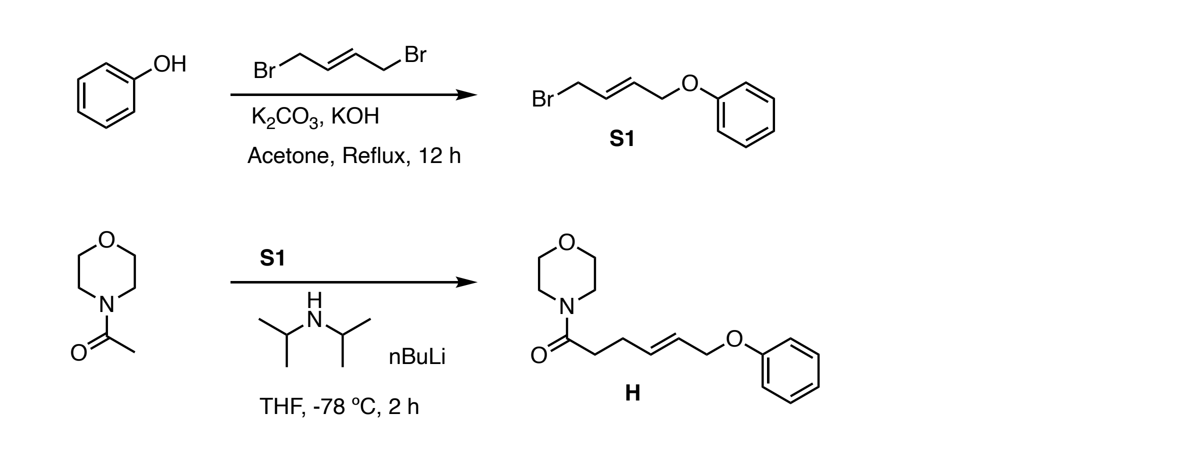

Scheme 1. Synthetic route for 1-morpholino-6-phenoxyhex-4-en-1-one (**H**)**.** The first step involves a controlled substitution of a 1,4-dibromobut-2-ene with phenol to form (4-bromobut-2-en-1-yl)oxybenzene (**S1**). **S1** (w/o further purification) was used in a subsequent addition with N-acetylmorpholine to form 1-morpholino-6-phenoxyhex-4-en-1-one (**H**)

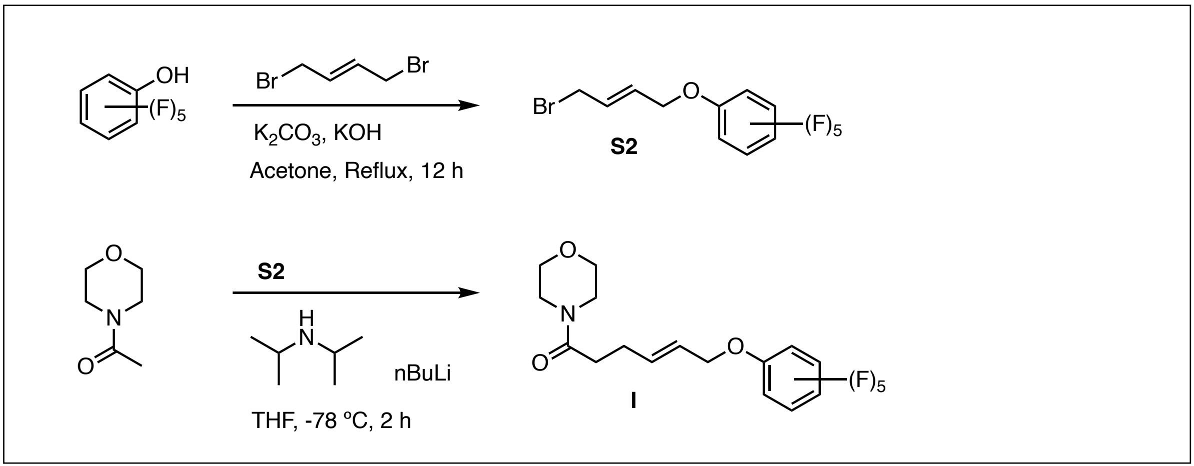

Scheme 2. Synthetic route for 1-morpholino-6-(perfluorophenoxy)hex-4-en-1-one (**I**). The first step involves a controlled substitution of a 1,4-dibromobut-2-ene with pentafluorophenol to form 1-(4-bromobut-2-en-1-yl)oxy-pentafluorobenzene (**S2**). **S2** (w/o further purification) was used in a subsequent addition with N-acetylmorpholine to form 1-morpholino-6-(perfluorophenoxy)hex-4-en-1-one (**I**)

1-morpholino-6-phenoxyhex-4-en-1-one (H)

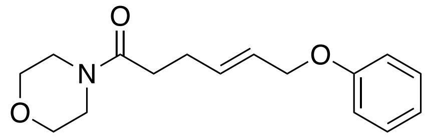

K_2CO_3 (0.646 g, 1 equiv., 4.67 mmol), KOH (0.131 g, 0.5 equiv., 2.33 mmol) and phenol (0.528 g, 1.2 equiv., 5.61 mmol) were dissolved in acetone. 1,4-dibromobut-2-ene (1 g, 1 equiv, 4.67 mmol) was added to the mixture and the reaction was stirred for 12 h under reflux. The crude product was extracted with DCM (3 x 20 mL) and used in next step without further purification. Diisopropylamine (0.5 mL, 1 equiv., 3.52 mmol) was dissolved in THF (10 mL) at -78 ºC and nBuLi (1.6 M) (2.2 mL, 1 equiv, 3.52 mmol) was added dropwise and allowed to stir for 1 h. N-acetyl morpholine (0.45 mL, 1.2 equiv., 3.87 mmol) was dissolved in THF (5 mL) and was added slowly keeping the temperature at –78 ºC and the reaction stirred for 1h. This reaction mixture was then added through cannula to a solution of (4-bromobut-2-en-1-yl)oxybenzene **S1** (0.8 g, 1 equiv., 3.52 mmol) in THF (10 mL) at –78 ºC. The reaction mixture was then left to warm up to 20 ºC over 1.5 h. the reaction was diluted with 0.1 M HCl (50 mL) and extracted with DCM (3 x 50 mL). The crude material was purified by flash chromatography with 30% EtOAc/petrol on silica (50 g) to give the product (0.53 g, 1.9 mmol, 55%) as a colourless liquid. $\mathbf{R_f}$ (60% EtOAc/ Hex) 0.3

^{1}H NMR (400 MHz, $CDCl_3$) δ 7.33 – 7.26 (m, 2H), 6.93 (dd, *J* = 20.3, 7.7 Hz, 3H), 5.97 – 5.70 (m, 2H), 4.47 (d, *J* = 5.8 Hz, 2H), 3.73 – 3.40 (m, 8H), 2.44 (d, *J* = 4.9 Hz, 4H).

^{13}C-NMR (101 MHz, $CDCl_3$) δ δ 170.7, 158.6, 133.6, 129.5, 129.4, 125.9, 120.8, 114.7, 68.4, 66.9, 66.6, 45.9, 41.9, 32.3, 27.8.

ESI-MS: calcd. for $C_{16}H_{21}NO_3$ $[M+H]^+$ m/z 275.3, found 276.2

1-Morpholino-6-(perfluorophenoxy)hex-4-en-1-one (I)

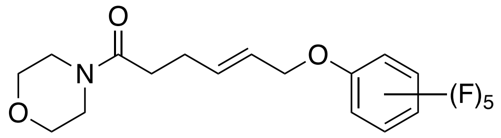

K_2CO_3 (1.12 g, 1.5 equiv., 8.14 mmol) and pentafluorophenol (1 g, 1 equiv., 5.43 mmol) were dissolved in acetone. 1,4-dibromobut-2-ene (1.162 g, 1 equiv, 5.43 mmol) was added to the mixture and the reaction was stirred for 12 h under reflux. The crude product was extracted with DCM (3 x 20 mL) and used in next step without further purification. Diisopropylamine (0.5 mL, 1.1 equiv., 3.46 mmol) was dissolved in THF (10 mL) at -78 ºC and nBuLi (1.6 M) (2.16 mL, 1.1 equiv, 3.46 mmol) was added dropwise and allowed to stir for 1 h. N-acetyl morpholine (0.40 mL, 1.1 equiv., 3.46 mmol) was dissolved in THF (5 mL) and was added slowly keeping the temperature at –78 ºC and the reaction stirred for 1h. This reaction mixture was then added through cannula to a solution of **S2** (1 g, 1 equiv., 3.15 mmol) in THF (10 mL) at –78 ºC. The reaction mixture was then left to warm up to 20 ºC over 1.5 h. the reaction was diluted with 0.1 M HCl (50 mL) and extracted with DCM (3 x 50 mL). The combined organic phases were dried over $MgSO_4$ and evaporated *in vacuo*. The crude material was purified by flash chromatography with 30% EtOAc/petrol on silica (50 g) to give the product (0.8 g, 3.15 mmol, 69%) as a colourless liquid. $\mathbf{R_f}$ (60% EtOAc/ Hex) 0.40

^{1}H NMR (400 MHz, $CDCl_3$) δ 5.92 – 5.80 (m, 1H), 5.78 – 5.67 (m, 1H), 4.58 (d, *J* = 6.6 Hz, 2H), 3.75 – 3.38 (m, 8H), 2.49 – 2.32 (m, 4H).

^{13}C-NMR (101 MHz, $CDCl_3$) 170.4, 137.0, 124.7, 66.9, 66.6, 45.8, 41.9, 32.1, 27.5.

ESI-MS: calcd. for $C_{16}H_{16}F_5NO_3$ $[M+H]^+$ m/z 365.3, found 366.3

3-(methylthio)-1-morpholinopropan-1-one (J)

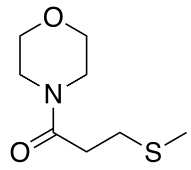

1-morpholinoprop-2-en-1-one (0.3 g, 1 equiv., 2.13 mmol) was dissolved in DCM (10 mL). DIPEA (1.11 mL, 3 equiv., 6.38 mmol) was added followed by ethanethiol (0.15 mL, 1 equiv., 2.13 mmol). The reaction was allowed to stir at 25 °C for 12 hour. The solvent was evaporated, and the crude purified by column chromatography with 50 - 60 % EtOAc/Hex on silica (70 g) to give the desired product 1-morpholinoprop-2-en-1-one (0.3 g, 2.13 mmol, 70%).

R_f (60% EtOAc/ Hex) 0.43

^{1}H NMR (500 MHz, $CDCl_3$) δ 3.74 – 3.46 (m, 8H), 2.87 (t, *J* = 7.6 Hz, 2H), 2.68 – 2.55 (m, 4H), 1.29 (t, *J* = 7.4 Hz, 3H).

^{13}C-NMR (101 MHz, $CDCl_3$) δ 169.9, 66.9, 66.6, 45.9, 42.0, 33.5, 26.9, 26.4, 14.8.

ESI-MS: calcd. for $C_9H_{17}NO_2S$ $[M+H]^+$ m/z 203.2, found 204.1

***N*-methyl-*N*-phenylpent-4-enamide (K)**[5]

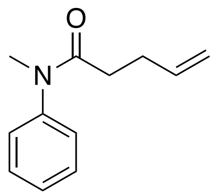

N-methylaniline (303 µL, 1 equiv., 2.80 mmol) was dissolved in freshly distilled DCM (10 mL) in an oven dried flask and DIPEA (975 µL, 2 equiv., 5.60 mmol) was added, followed by dropwise pent-4-enoic anhydride (512 µL, 1 equiv., 2.80 mmol) at room temperature. After 6 h, the reaction was diluted with 3 M HCl (20 mL) and extracted with DCM (3 x 10 mL). The combined organic phases were dried over $MgSO_4$ and evaporated *in vacuo*. The crude material was purified by flash chromatography with 30% EtOAc/petrol on silica (50 g) to give the product (120 mg, 634 µmol, 22.6%) as a colour liquid.

R_f (80% EtOAc/ Hex) 0.66

^{1}H NMR (400 MHz, $CDCl_3$) δ 7.62 – 7.02 (m, 5H), 5.86 – 5.62 (m, 1H), 4.94 (t, J = 13.8 Hz, 2H), 3.29 (s, 3H), 2.26 (dt, J = 65.5, 7.5 Hz, 4H).

^{13}C-NMR (101 MHz, $CDCl_3$) δ 172.3, 144.1, 137.5, 129.7, 127.8, 127.7, 127.3, 114.9, 37.3, 33.5, 29.5.

ESI-MS: calcd. for $C_{12}H_{15}NO$ $[M+H]^+$ m/z 189.1, found 189.0

***N*-benzyl-*N*-methylpent-4-enamide (L)**[6]

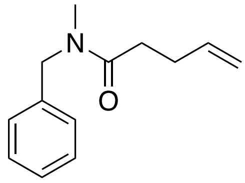

N-methyl-1-phenylmethanamine (213 µL, 1 equiv, 1.65 mmol) was solved in freshly distilled DCM (10 mL) in an oven dried flask and DIPEA (575 µL, 2 equiv, 3.30 mmol) was added, followed by dropwise pent-4-enoic anhydride (302 µL, 1 equiv, 1.65 mmol) at room temperature. After 6 h, the reaction was diluted with 3 M HCl (20 mL) and extracted with DCM (3 x 10 mL). The combined organic phases were dried over $MgSO_4$ and evaporated *in vacuo*. The crude material was purified by flash chromatography with 30% EtOAc/petrol on silica (50 g) to give the product (150 mg, 738 µmol, 44.7%) as a colour liquid.

R_f (80% EtOAc/ Hex) 0.6

^{1}H NMR (400 MHz, $CDCl_3$) δ 7.50 – 7.11 (m, 5H), 5.98 – 5.80 (m, 1H), 5.23 – 4.91 (m, 2H), 4.60 (d, J = 24.1 Hz, 2H), 2.96 (d, J = 13.5 Hz, 3H), 2.63 – 2.37 (m, 4H).

^{13}C-NMR (101 MHz, CDCl3) δ 172.4, 137.6, 128.9, 128.6, 128.0, 115.2, 53.3, 50.8, 34.8, 34.0, 32.4, 29.2.

ESI-MS: calcd. for $C_{13}H_{17}NO$ $[M+H]^+$ m/z 203.1, found 203.0

6-bromo-2-butyl-1*H*-benzo[*de*]isoquinoline-1,3(2*H*)-dione (S3)[7]

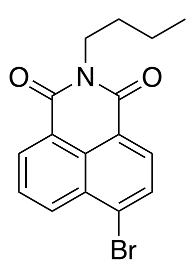

4-bromo-1,8-naphthalic anhydride (2.00 g, 1.0 equiv., 7.21 mmol) was dissolved in EtOH (10 mL) and butyl-amine (502 mg, 1.0 equiv., 7.21 mmol) was added. The solution was stirred at 80 °C for 24 h. The liquor was then cooled, and the product filtered, washed with H_2O, and dried in vacuum to give the product (760 mg, 2.29 mmol, 31.7%) as a white solid.

^{1}H NMR (300 MHz, $CDCl_3$) δ 8.62 (dt, J = 7.3, 1.0 Hz, 1H), 8.51 (dt, J = 8.5, 1.1 Hz, 1H), 8.37 (dd, J = 7.9, 0.9 Hz, 1H), 8.00 (dd, J = 7.9, 0.9 Hz, 1H), 7.81 (dd, J = 8.5, 7.3 Hz, 1H), 4.21 – 4.09 (m, 2H), 1.78 – 1.62 (m, 2H), 1.44 (dt, J = 14.7, 7.4 Hz, 2H), 0.98 (t, J = 7.3 Hz, 3H).

^{13}C-NMR (75 MHz, $CDCl_3$) δ 163.5, 163.5, 133.1, 131.9, 131.0, 130.5, 130.1, 128.9, 128.0, 123.1, 122.2, 99.9, 40.3, 30.1, 20.3, 13.8.

ESI-MS: calcd. for $C_{16}H_{15}BrNO_2$ $[M+H]^+$ m/z 332.1, found 332.5

2-butyl-6-(piperazin-1-yl)-1*H*-benzo[*de*]isoquinoline-1,3(2*H*)-dione (F1-3)[7]

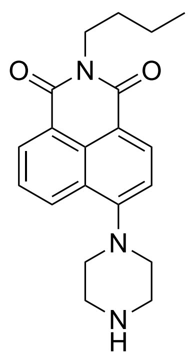

6-bromo-2-butyl-1*H*-benzo[*de*]isoquinoline-1,3(2*H*)-dione **(S3)** (250 mg, 1.0 equiv., 0.75 mmol) was dissolved in DMF and piperazine (250 mg, 4.0 equiv., 2.97 mmol) was added. The reaction was allowed to reflux at 170 ºC for 24 h. The solvent was evaporated, and the crude purified by column chromatography with 5% MeOH/ DCM on silica to give the desired product (163 mg, 0.48 mmol, 64%) as a yellow powder.

^{1}H NMR (300 MHz, $CDCl_3$) δ 8.65 (dd, *J* = 7.3, 1.1 Hz, 1H), 8.61 – 8.55 (m, 1H), 7.80 (dd, *J* = 8.5, 7.3 Hz, 1H), 7.35 (d, *J* = 8.0 Hz, 1H), 4.25 – 4.11 (m, 2H), 3.61 (d, *J* = 37.4 Hz, 7H), 1.77 – 1.64 (m, 2H), 1.43 (dt, *J* = 14.6, 7.4 Hz, 2H), 0.98 (t, *J* = 7.3 Hz, 3H).

^{13}C-NMR (75 MHz, $CDCl_3$) δ 164.6, 164.3, 153.8, 132.6, 131.9, 129.5, 129.4, 126.8, 126.0, 122.9, 118.2, 116.9, 116.0, 113.2, 49.7, 44.5, 40.5, 30.0, 20.2, 13.7.
ESI-MS: calcd. for $C_{20}H_{24}N_3O_2$ $[M+H]^+$ m/z 338.2, found 338.5

2-butyl-6-(methylamino)-1*H*-benzo[*de*]isoquinoline-1,3(2*H*)-dione (F4)[7]

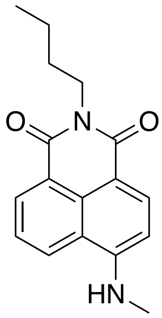

A mixture of 4-bromo-*N*-alkylnaphthalimide (1.50 g, 1.0 equiv., 4.53 mmol) and methylamine (869 mg, 2.5 equiv., 11.19 mmol as 40% aqueous solution) was dissolved in 50 mL of DMF, and triethylamine (456 mg, 1.0 equiv., 4.51 mmol) was then added. The solution was refluxed for 18 h (170 ºC) and, after cooling to room temperature, the solvent evaporated and the crude purified by column chromatography with 5% MeOH/ DCM on silica to give the desired product (663 mg, 2.07 mmol, 45.7%) as a yellow powder.

^{1}H NMR (300 MHz, $CDCl_3$) δ 8.60 (dd, J = 7.3, 1.1 Hz, 1H), 8.51 (d, J = 8.4 Hz, 1H), 8.07 (dd, J = 8.5, 1.1 Hz, 1H), 7.63 (dd, J = 8.4, 7.3 Hz, 1H), 6.73 (d, J = 8.4 Hz, 1H), 4.22 – 4.12 (m, 2H), 3.16 (s, 3H), 1.77 – 1.66 (m, 2H), 1.49 – 1.38 (m, 2H), 0.98 (t, J = 7.3 Hz, 3H).

^{13}C NMR (75 MHz, $CDCl_3$) δ 163.6, 163.5, 133.1, 131.9, 131.1, 131.0, 130.5, 130.1, 128.9, 128.0, 123.1, 122.2, 99.9, 40.3, 30.2, 20.4, 13.8

ESI-MS (ESI): calcd. for $C_{17}H_{19}N_2O_2$ $[M+H]^+$ m/z 282.1, found 282.0

Allyl 4-(2-butyl-1,3-dioxo-2,3-dihydro-1*H*-benzo[*de*]isoquinolin-6-yl)piperazine-1-carboxylate (QF1)

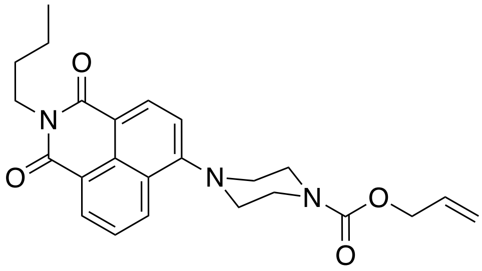

2-butyl-6-(piperazin-1-yl)-1H-benzo[de]isoquinoline-1,3(2H)-dione (60 mg, 1 equiv., 0.18 mmol) was dissolved in dry toluene (10 ml). Allyl chloroformate (38 µL, 2 equiv., 0.36 mmol) was added followed by DIPEA (62 µL, 2 equiv., 0.36 mmol). The reaction was allowed to reflux at 120 °C for 24 h. The solvent was evaporated, and the crude purified by column chromatography with 80 % EtOAc/petrol on silica to give the desired product (27 mg, 64 µmol, 36%) as a yellow powder.

R_f (80% EtOAc/ Hex) 0.6

^{1}H NMR (400 MHz, $CDCl_3$) δ 8.65 – 8.41 (m, 3H), 7.75 (t, J = 7.9 Hz, 1H), 7.25 (d, J = 8.0 Hz, 1H), 6.00 (ddd, J = 16.4, 10.9, 5.4 Hz, 1H), 5.33 (dd, J = 35.4, 13.8 Hz, 2H), 4.69 (d, J = 5.6 Hz, 2H), 4.20 (t, J = 7.6 Hz, 2H), 3.85 (t, J = 4.9 Hz, 4H), 3.25 (t, J = 4.9 Hz, 4H), 1.73 (p, J = 7.5 Hz, 2H), 1.46 (dt, J = 14.9, 7.5 Hz, 2H), 1.00 (t, J = 7.3 Hz, 3H).

^{13}C NMR (101 MHz, $CDCl_3$) δ 164.4, 163.9, 155.4, 155.1, 132.8, 132.3, 131.2, 129.7, 126.3, 126.0, 123.4, 117.7, 117.6, 115.3, 66.3, 52.9, 43.9, 40.1, 30.2, 20.4, 13.8.

HR-MS (ESI): calcd. for $C_{24}H_{27}N_3O_4$ $[M+H]^+$ m/z 422.2074, found 422.2077

2-butyl-6-(4-(pent-4-enoyl)piperazin-1-yl)-1*H*-benzo[*de*]isoquinoline-1,3(2*H*)-dione (QF2)

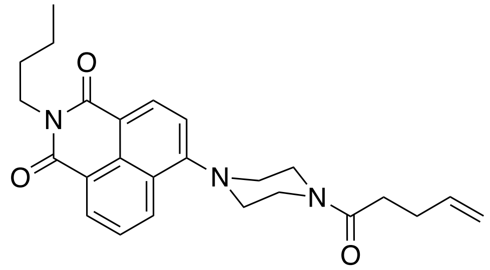

2-butyl-6-(piperazin-1-yl)-1H-benzo[de]isoquinoline-1,3(2H)-dione (25 mg, 1equiv., 74 µmol) was dissolved in dry toluene (10 ml). Pent-4-enoic anhydride (68 µL, 5 equiv., 0.37 mmol) was added followed by DMAP (18 mg, 2 equiv., 0.15 mmol). The reaction was allowed to reflux at 120 ºC for 24 h. The solvent was evaporated, and the crude purified by column chromatography with 80% EtOAc/petrol on silica to give the desired product (12 mg, 29 µmol, 39%) as a yellow powder.

R_f (80% EtOAc/ Hex) 0.55

^{1}H NMR (400 MHz, $CDCl_3$) δ 8.70 – 8.39 (m, 3H), 7.76 (t, J = 7.9 Hz, 1H), 7.25 (d, J = 8.0 Hz, 1H), 5.93 (m, 1H), 5.10 (dd, J = 26.5, 13.6 Hz, 2H), 4.20 (t, J = 7.6 Hz, 2H), 3.98 (s, 2H), 3.83 (t, J = 4.9 Hz, 2H), 3.26 (m, 4H), 2.52 (m, 4H), 1.73 (m, 2H), 1.47 (m, 2H), 1.00 (t, J = 7.3 Hz, 3H).

^{13}C NMR (101 MHz, $CDCl_3$) δ 171.1, 164.4, 163.9, 155.0, 137.2, 132.3, 131.2, 129.8, 129.7, 126.3, 126.1, 123.5, 117.7, 115.5, 115.4, 53.1, 45.7, 41.7, 40.1, 32.6, 30.2, 29.2,20.4, 13.8.

HR-MS (ESI): calcd. for $C_{25}H_{29}N_3O_3$ $[M+H]^+$ m/z 420.2282, found 420.2282

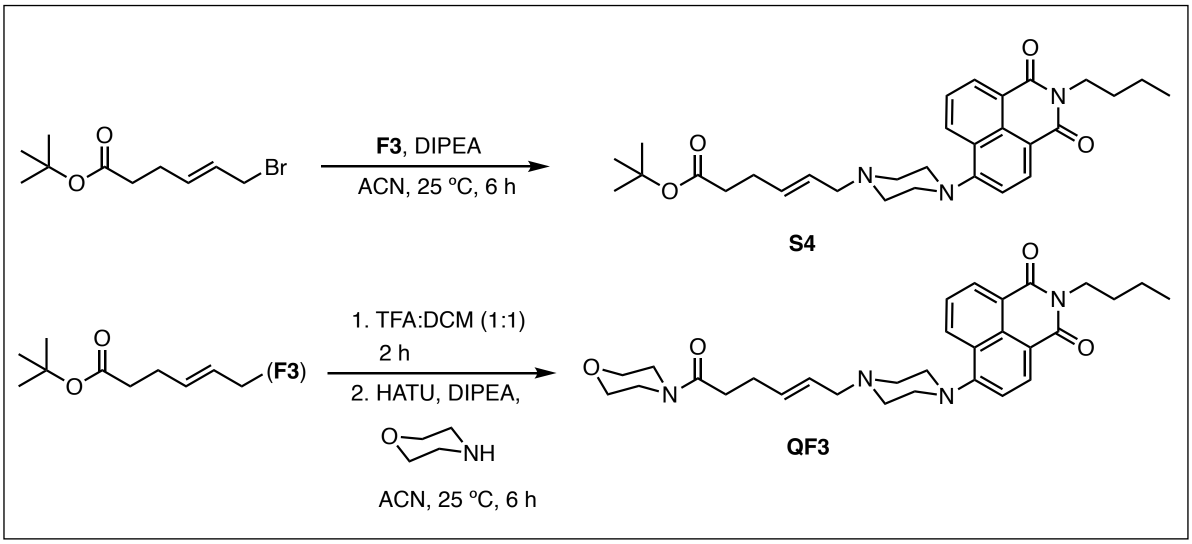

Scheme 3. Synthetic route for **QF3.** The first step involves the substitution of a *tert*-butyl-6-bromohex-4-enoate with 2-butyl-6-(piperazin-1-yl)-1*H*-benzo[*de*]isoquinoline-1,3(2*H*)-dione (**F3**) to form **S4**, which (w/o further purification) was used in a subsequent amide coupling reaction with morpholine to form **QF3**.

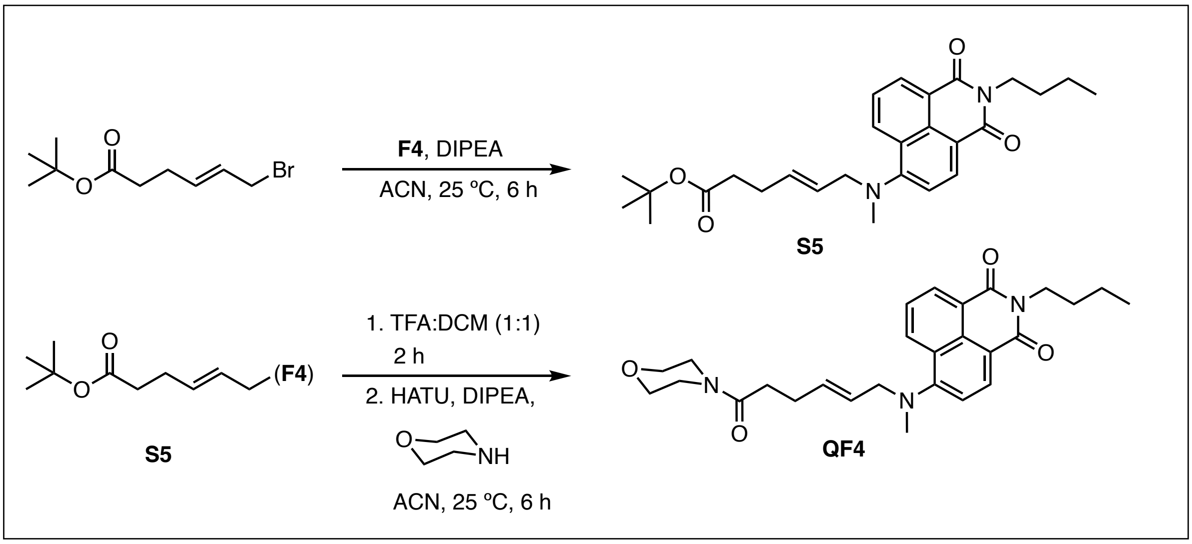

Scheme 4. Synthetic route for **QF4.** The first step involves the substitution of a *tert*-butyl-6-bromohex-4-enoate with 2-butyl-6-(methylamino)-1*H*-benzo[*de*]isoquinoline-1,3(2*H*)-dione (**F4**) to form **S5**, which (w/o further purification) was used in a subsequent amide coupling reaction with morpholine to form **QF3**.

2-butyl-6-(4-(6-morpholino-6-oxohex-2-en-1-yl)piperazin-1-yl)-1*H* benzo[*de*]isoquinoline-1,3(2*H*)-dione (QF3)

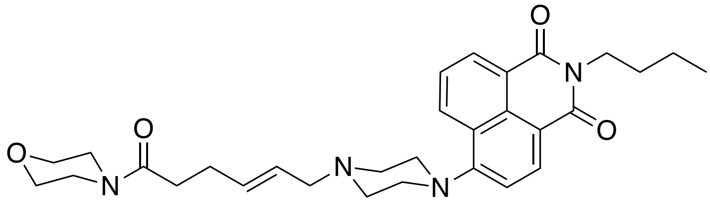

2-butyl-6-(piperazin-1-yl)-1*H*-benzo[*de*]isoquinoline-1,3(2*H*)-dione (**F3**) (100 mg, 1 equiv., 296 µmol) was dissolved in ACN (15 mL) followed by the addition of DIPEA (41 µL, 1 equiv., 296 µmol). After 5 min, tert-butyl-6-bromohex-4-enoate (73 mg, 1 equiv., 296 µmol) was added and the reaction stirred for 6 h at room temperature. The crude **S4** formed was rota-evaporated, redissolved in DCM: TFA (1:1) (4 mL) and stirred for 2 h to form the acid. The solvent was rota-evaporated, and the crude acid was used in the next step without further purification.

The crude acid (40 mg, 1 equiv., 89 µmol) was redissolved in ACN (15 mL) and HATU (135 mg, 4.0 eq., 356 µmol) added followed by DIPEA (31 µL, 2.0 eq., 178 µmol) at room temperature. After 10 min, morpholine (25 µL, 3.0 eq., 267 µmol) was added in ACN (5 mL). The reaction was stirred for 6 h then evaporated *in vacuo*. The crude product was dissolved in DCM (30 mL) and washed with 3 M HCl (10 mL), sat. aq. $NaHCO_3$ solution (10 mL) and water (10 mL). The organic phase was further extracted thrice with DCM (75 mL), dried over $MgSO_4$, filtered, and evaporated *in vacuo*. The crude material was purified by column chromatography with EtOAc/ Hexane on silica (60 g) to give the 2-butyl-6-(4-(6-morpholino-6-oxohex-2-en-1-yl)piperazin-1-yl)-1*H* benzo[*de*]isoquinoline-1,3(2*H*)-dione (**QF3**) (25 mg, 48 µmol, 54%) as a yellow powder.

R_f (80% EtOAc/ Hex) 0.45

^{1}H NMR (400 MHz, CD_3CN) δ 8.53 – 8.35 (m, 3H), 7.72 (dd, *J* = 8.5, 7.3 Hz, 1H), 7.26 (d, *J* = 8.1 Hz, 1H), 5.81 – 5.68 (m, 1H), 5.64 – 5.51 (m, 1H), 4.15 – 4.05 (m, 2H), 3.55 (ddq, *J* = 45.3, 15.9, 4.8 Hz, 8H), 3.26 (t, *J* = 4.8 Hz, 4H), 3.06 (dd, *J* = 6.5, 1.2 Hz, 2H), 2.71 (t, *J* = 4.7 Hz, 4H), 2.43 (ddd, *J* = 7.8, 6.6, 1.4 Hz, 2H), 2.39 – 2.30 (m, 2H), 1.74 – 1.62 (m, 2H), 1.42 (h, *J* = 7.3 Hz, 2H), 0.98 (t, *J* = 7.4 Hz, 3H).

^{13}C NMR (101 MHz, CD_3CN) δ 170.6, 164.1, 163.6, 155.9, 133.1, 131.9, 130.4, 130.4, 129.6, 127.2, 125.9, 125.7, 123.3, 117.3, 116.5, 114.9, 66.5, 66.4, 60.2, 52.9, 52.7, 45.7, 41.6, 39.6, 32.1, 29.9, 27.7, 20.1, 13.2

ESI-MS: calcd. for $C_{30}H_{38}N_4O_4$ $[M+H]^+$ m/z 518.6, found 519.5

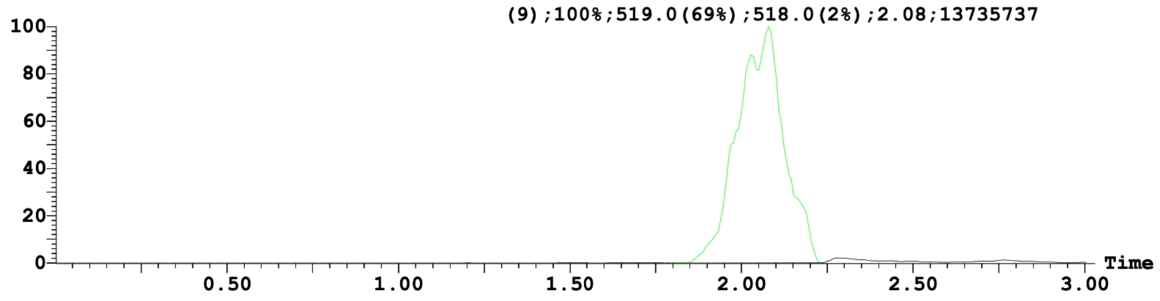

LC trace of **FQ3**: **R_t** = 2.08 min – peak confirmed by mass spectrometry.

2-butyl-6-(methyl(6-morpholino-6-oxohex-2-en-1-yl)amino)-1*H*-benzo[*de*]isoquinoline-1,3(2*H*)-dione (QF4)

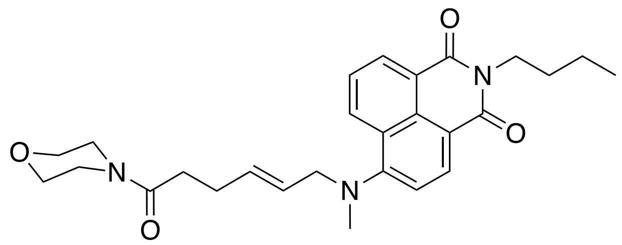

2-butyl-6-(methylamino)-1*H*-benzo[*de*]isoquinoline-1,3(2*H*)-dione (**F4**) (100 mg, 1 equiv., 354 µmol) was dissolved in ACN (15 mL) followed by the addition of DIPEA (92 µL, 1.5 equiv., 531 µmol). After 5 min, tert-butyl-6-bromohex-4-enoate (176 mg, 2 equiv., 708 µmol) was added and the reaction stirred for 6 h at room temperature. The crude **S5** formed was rota-evaporated, redissolved in DCM: TFA (1:1) (4 mL) and stirred for 2 h to form the acid. The solvent was rota-evaporated, and the crude acid was used in the next step without further purification.

The crude acid (15 mg, 1 equiv., 38 µmol) was redissolved in ACN (5 mL) and HATU (43 mg, 3.0 eq., 0.1 mmol) added followed by DIPEA (13 µL, 2.0 eq., 76 µmol) at room temperature. After 10 min, morpholine (7 µL, 2.0 eq., 76 µmol) was added in ACN (5 mL). The reaction was stirred for 6 h then evaporated *in vacuo*. The crude product was dissolved in DCM (30 mL) and washed with 3 M HCl (10 mL), sat. aq. $NaHCO_3$ solution (10 mL) and water (10 mL). The organic phase was further extracted thrice with DCM (75 mL), dried over $MgSO_4$, filtered, and evaporated *in vacuo*. The crude material was purified by column chromatography with EtOAc/ Hexane on silica (60 g) to give **QF4** (8 mg, 0.02 mmol, 50%) as a yellow powder.

R_f (80% EtOAc/ Hex) 0.50

ESI-MS: calcd. for $C_{27}H_{33}N_3O_4$ $[M+H]^+$ m/z 463.5, found 464.2

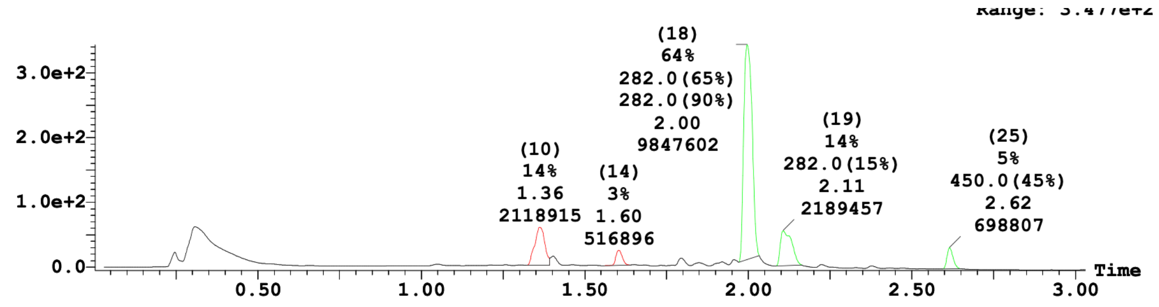

LC trace of crude **S5** (used w/o further purification): **R_t** = 2.00 min – peak confirmed by mass spectrometry.

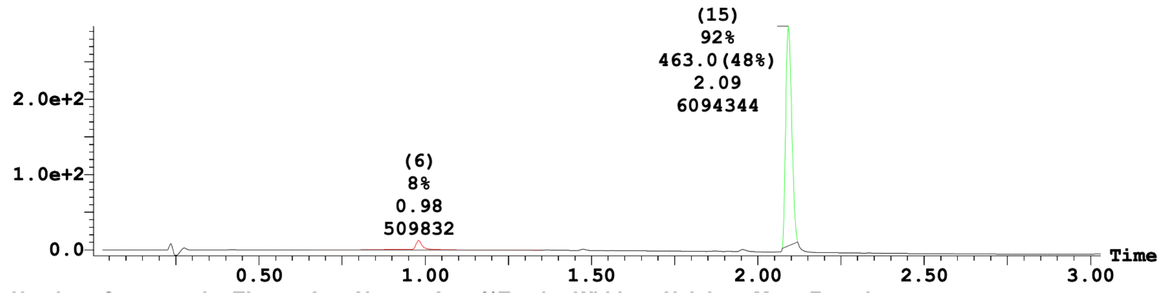

LC trace of **FQ4**: **R_t** = 2.09 min – peak confirmed by mass spectrometry.

PD1

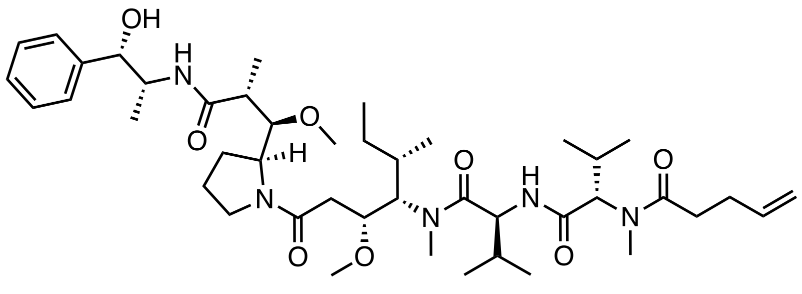

4-pentenoic acid (5.23 mg, 3.0 eq., 52.2 μmol) was solved in ACN (5 mL) and HATU (19.9 mg, 3.0 eq., 52.2 μmol) added followed by DIPEA (9 μL, 3.0 eq., 52.2 μmol) at room temperature. After 10 mins, MMAE (12.5 mg, 1.0 eq., 17.4 μmol) was added in one portion. The reaction was stirred for 6 h then evaporated *in vacuo*. The crude product was dissolved in DCM (10 mL) and washed with 3 M HCl (5 mL), water basified to ~pH 8 with sat. aq. $NaHCO_3$ solution (5 mL) and water (5 mL). The organics were dried over $MgSO_4$, filtered and evaporated *in vacuo*. The crude material was purified by column chromatography with 7-10% MeOH/ DCM on silica to give the desired product (8 mg, 17.4 μmol, 60%) as a white solid.

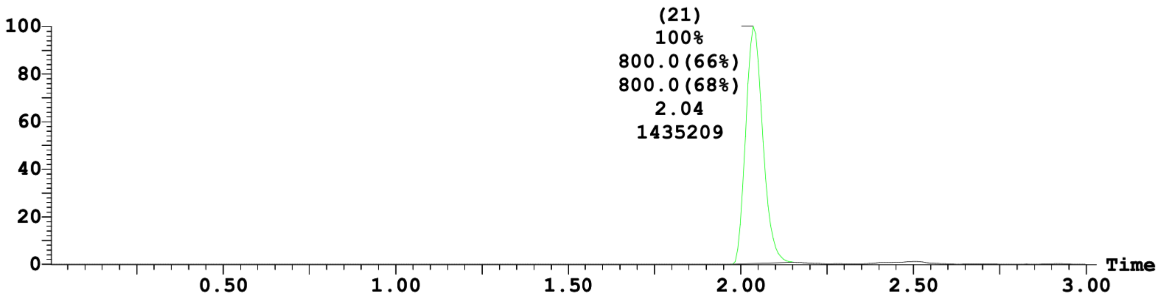

LC trace of **PD1**: $\mathbf{R_t}$ = 2.04 min – peak confirmed by mass spectrometry.

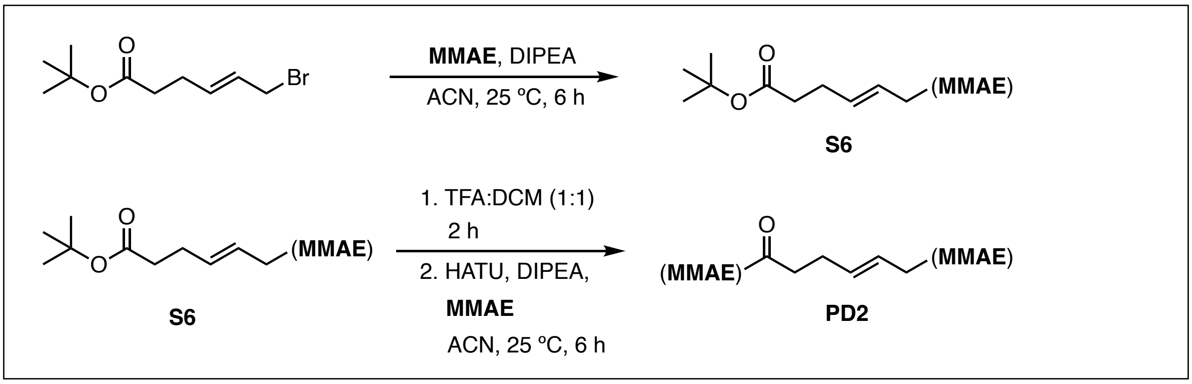

Scheme 5. Synthetic route for **PD2.** The first step involves the substitution of a *tert*-butyl-6-bromohex-4-enoate with MMAE to form **S6**, which (w/o further purification) was used in a subsequent amide coupling reaction with MMAE to form **PD2**.

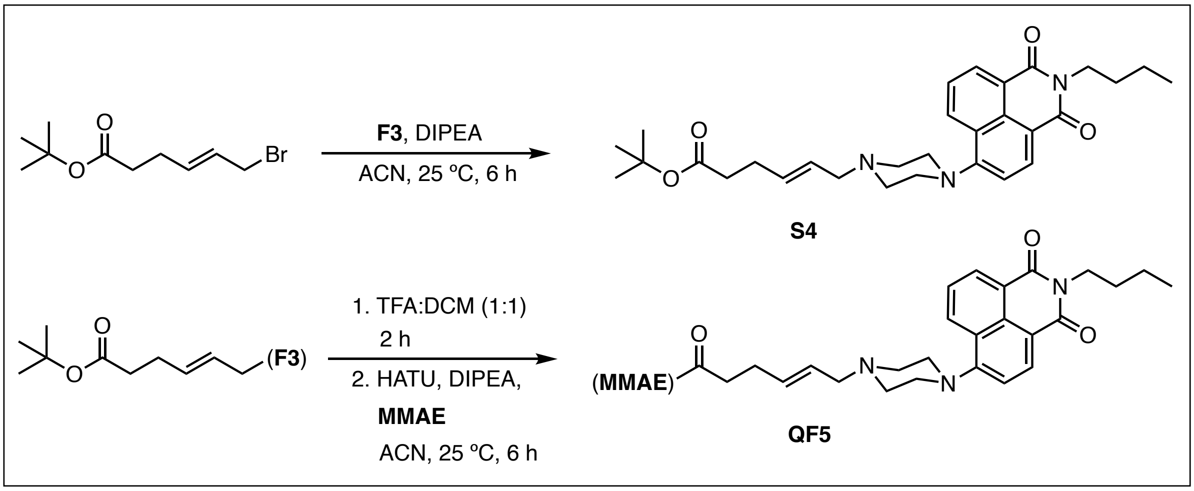

Scheme 6. Synthetic route for **MMAE-F3.** The first step involves the substitution of a *tert*-butyl-6-bromohex-4-enoate with **F3** to form **S4**, which (w/o further purification) was used in a subsequent amide coupling reaction with MMAE to form **MMAE-F3**.

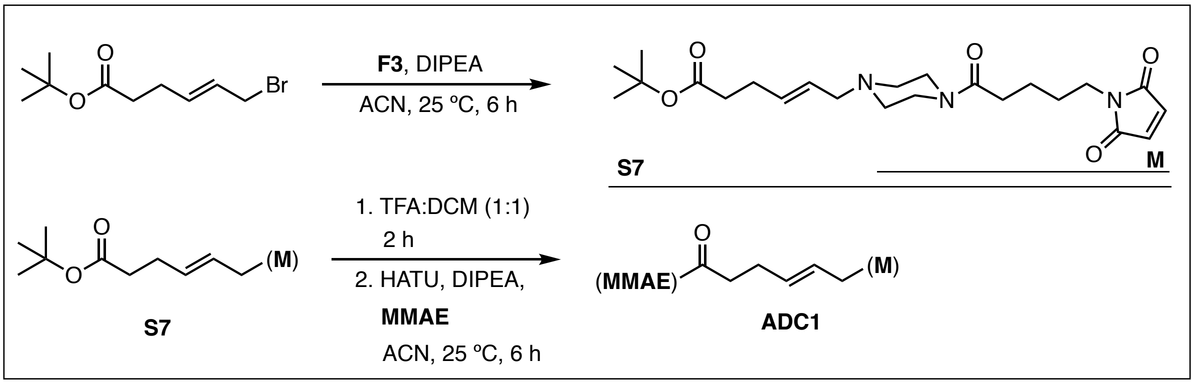

Scheme 7. Synthetic route for **MMAE-F3.** The first step involves the substitution of a *tert*-butyl-6-bromohex-4-enoate with **F3** to form **S4**, which (w/o further purification) was used in a subsequent amide coupling reaction with MMAE to form **MMAE-F3**.

PD2

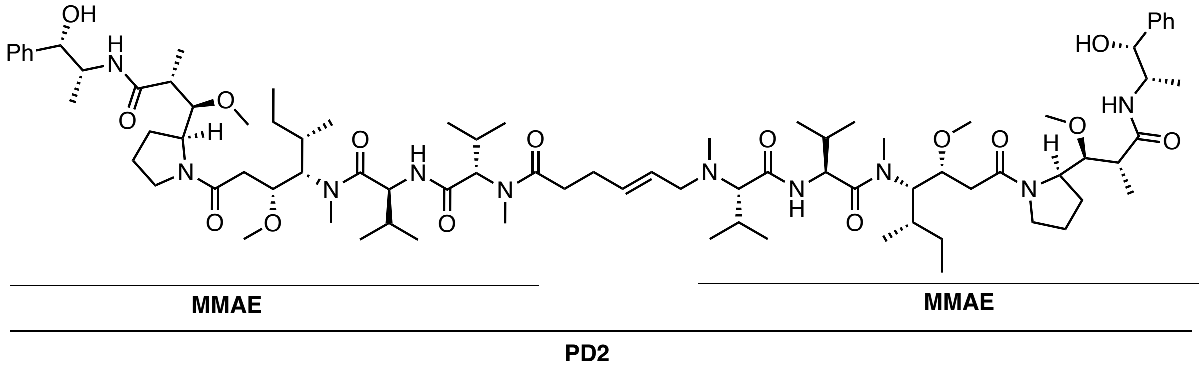

MMAE (28.5 mg, 1 equiv., 39.7 µmol) was dissolved in ACN (2 mL) followed by the addition of DIPEA (21 µL, 3 equiv., 119 µmol). After 5 min, tert-butyl-6-bromohex-4-enoate (40 mg, 4 equiv., 159 µmol) was added and the reaction stirred for overnight at room temperature. The crude **S6** formed was rota-evaporated redissolved in DCM: TFA (1:1) (4 mL) and stirred for 2 h to form the acid. The crude acid (22 mg, 1 equiv, 27 µmol) was redissolved in ACN (5 mL) and HATU (30 mg, 3.0 eq., 80 µmol) added followed by DIPEA (14 µL, 3.0 eq., 80 µmol) at room temperature. After 10 mins, MMAE (19 mg, 1.0 eq., 27 µmol) was added in one portion. The reaction was stirred for 6 h then evaporated *in vacuo*. The crude product was extracted with EtOAc and the organics were dried over $MgSO_4$, filtered and evaporated *in vacuo*. The crude material was purified by HPLC to give the desired product (14 mg, 9.1 µmol, 35%) as a white solid.

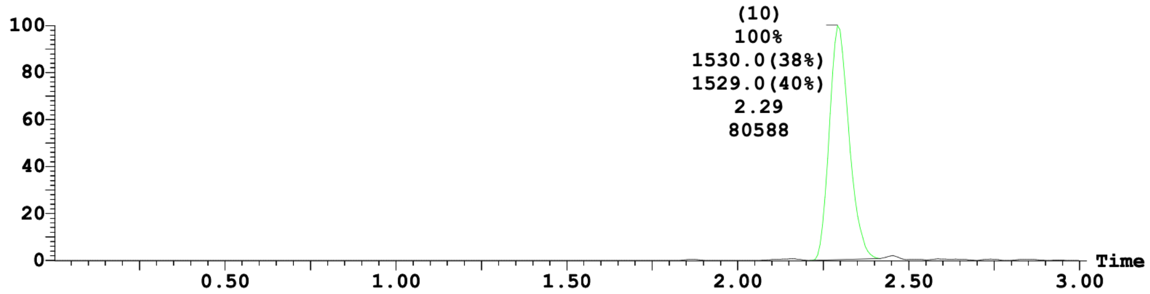

LC trace of **PD2**: $\mathbf{R_t}$ = 2.29 min – peak confirmed by mass spectrometry.

MMAE-F3

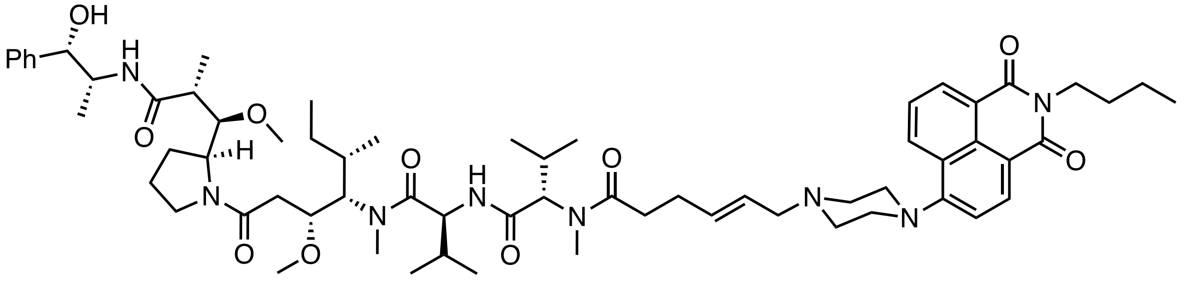

MMAE F3

MMAE-F3

The crude **S4** (synthesis described earlier) was rota-evaporated redissolved in DCM: TFA (1:1) (4 mL) and stirred for 2 h to form the acid. The crude acid (13 mg, 2 equiv., 28 µmol) was redissolved in ACN (5 mL) and HATU (21 mg, 4 equiv., 56 µmol) added followed by DIPEA (10 µL, 4 equiv., 56 µmol) at room temperature. After 10 mins, MMAE (10 mg, 1 equiv., 14 µmol) was added in one portion. The reaction was stirred for 6 h then evaporated *in vacuo*. The crude product was dissolved in DCM (10 mL) and washed with 3 M HCl (5 mL), water basified to ~pH 8 with sat. aq. $NaHCO_3$ solution (5 mL) and water (5 mL). The organics were dried over $MgSO_4$, filtered and evaporated *in vacuo*. The crude material was purified by HPLC to give the desired product (7 mg, 6 µmol, 40%) as a yellow solid. LC-MS trace shown in Figure **S21.**

MMAF-MMAE

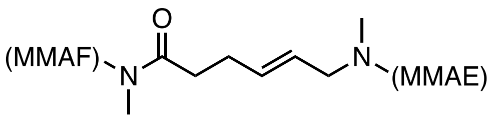

The crude **S6** formed was rota-evaporated redissolved in DCM: TFA (1:1) (4 mL) and stirred for 2 h to form the acid. The crude acid (25 mg, 1 equiv, 30 μmol) was redissolved in ACN (5 mL) and HATU (15 mg, 1.3 eq., 40 μmol) added followed by DIPEA (10 μL, 2.0 eq., 60 μmol) at room temperature. After 10 mins, MMAF (29 mg, 1.3 eq., 40 μmol) was added in one portion. The reaction was stirred for 6 h then evaporated *in vacuo*. The crude material was purified by HPLC to give the desired product (8 mg, 5 μmol, 20%) as a white solid.

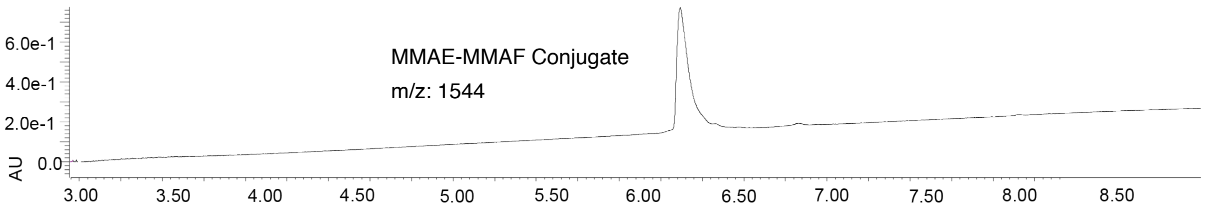

LC trace of **MMAF-MMAE**: $\mathbf{R_t}$ = 6.25 min – peak confirmed by mass spectrometry.

ADC1

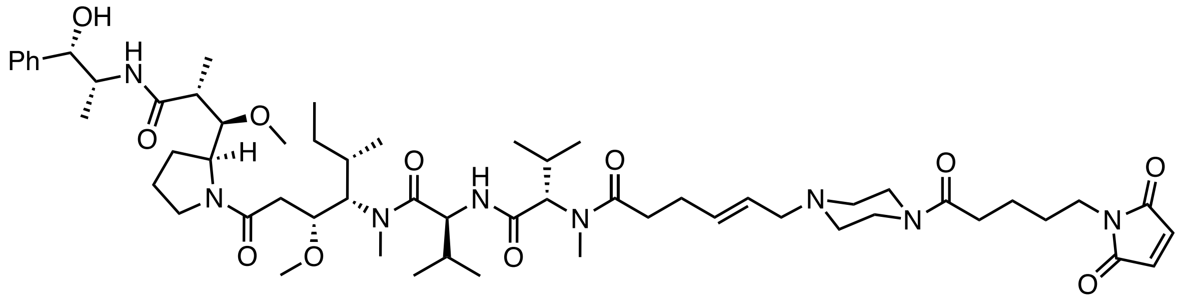

MMAE **Maleimide (M)**

ADC1

Maleimide derivative (**M**) (50 mg, 1 equiv., 0.18 mmol) was dissolved in ACN (10 mL) followed by the addition of DIPEA (62 µL, 2 equiv., 0.36 mol). After 5 min, tert-butyl-6-bromohex-4-enoate (49 mg, 1.1 equiv., 0.2 mmol) was added and the reaction stirred for overnight at room temperature. The crude **S7** formed was rota-evaporated redissolved in DCM: TFA (1:1) (4 mL) and stirred for 2 h to form the acid. The crude acid (22 mg, 1 equiv., 56 µmol) was redissolved in ACN (5 mL) and HATU (64 mg, 3.0 eq., 0.17 mmol) added followed by DIPEA (29 µL, 3.0 eq., 0.17 mmol) at room temperature. After 10 mins, MMAE (40 mg, 1.0 eq., 56 µmol) was added in one portion. The reaction was stirred for 6 h then evaporated *in vacuo*. The crude product was extracted with EtOAc and the organics were dried over $MgSO_4$, filtered and evaporated *in vacuo*. The crude material was purified by HPLC to give the desired product (15 mg, 14 µmol, 25%) as a white solid.

1.3 NMR and Fluorescence Assay Methods

NMR Assay: Substrates (~5 mg) were dissolved in MeOD (0.175 mL), and the metal salts were dissolved in D_2O (0.525 mL). Sonication was required for complexes with limited aqueous solubility such as AuCl. The solution of metal salts was then added to the solution of substrates, and this marked as time t = 0 min. The reactions were monitored at various time points. All reactions were performed at 37 ^{o}C in thermal shaker.

Fluorescence Assay: 100 μM solutions of compound (**QF**) were prepared in 50% deionized H_2O/DMF (v/v). Solutions of Pt or Au salts were prepared to give twice their desired final concentration in 50% deionized H_2O/DMF (v/v). For some conditions metal complexes were pre-incubated in H_2O /DMF 50% for 0 to 6 h at 37 ^{o}C. 100 μL aliquots of each caged fluorophores and platinum complexes were pipetted into a 96-well plate. Reaction progress was monitored at 37 °C by the increasing fluorescence of compounds: excitation 445 nm, emission 540 nm, bandwidth 20 nm. Studies with CS_2 or ethylene diamine tetra acetic acid (EDTA) were performed following the same protocol but adding 3 equiv. of the poisons relative to the metals. All reactions were performed in triplicate and in three independent days.

1.4 Quantum Mechanical Calculations. Full geometry optimizations and transition structure (TS) searches were carried out with Gaussian 16[8] using the M06 hybrid functional[9] and 6-311G(d,p) basis set for C, O, N, Cl, and H, and SDD[10,11] effective core potential for Au atoms in combination with ultrafine integration grids. Bulk solvent effects in water were considered implicitly through the SMD polarizable continuum model.[12] The possibility of different conformations was taken into account for all structures. Frequency analyses were carried out at the same level used in the geometry optimizations, and the nature of the stationary points was determined in each case according to the appropriate number of negative eigenvalues of the Hessian matrix. Thermal corrections were obtained at 298.15 K. The quasiharmonic approximation reported by Truhlar *et al.* was used to replace the harmonic oscillator approximation for the calculation of the vibrational contribution to enthalpy and entropy.[13] Scaled frequencies were not considered. Mass-weighted intrinsic reaction coordinate (IRC) calculations were carried out using the Hessian-based predictor-corrector integrator scheme by Hratchian and Schlegel[14,15] in order to ensure that the TSs indeed connected the appropriate reactants and products. Gibbs free energies (ΔG) were used for the discussion on the relative stabilities of the considered structures. The lowest energy conformer for each calculated stationary point (**Supporting Figures S25-S27**) was considered in the discussion; all the computed structures can be obtained from authors upon request. Electronic energies, entropies, enthalpies, Gibbs free energies, lowest frequencies and Cartesian coordinates of the calculated structures are summarized in **Supporting Table 3**.

1.5 Cell Culture Methods

HeLa (HeLa ATCC® CCL-2™) cells were kindly provided by the Shankar Balasubramanian (Department of Chemistry, University of Cambridge, UK) group, and were grown in a humidified incubator at 37 ºC under 5% CO2 with 90% humidity and split at approximately 80% confluence using Trypsin-EDTA solution 0.25%. HeLa cells were grown in high glucose DMEM (+ pyruvate) supplemented with 10% heat inactivated FBS, 100 units/mL penicillin and 100 µg/mL streptomycin, 1% non-essential amino acids, 2 mM GlutaMax™ and 10 mM HEPES.

Confocal Imaging: HeLa cells were grown in a humidified incubator at 37 ºC under 5% CO2 with 90% humidity and split at approximately 80% confluence using Trypsin-EDTA solution 0.25%. The metal (12 µM) was added after the slides were prepared (300 µL/ well; 1 * 10^5 cells/ mL) and was incubated at 37 ^oC overnight. Fluorophore (1 µL, 10 mM stock) was added and incubated for 90 minutes. Stain (Hoeschst; 10 mg/ mL) was added and incubated at 37 ^oC for 5-10 minutes. Paraformaldehyde (4 % in PBS was used to fix the cells before carrying out the imaging in confocal microscope.

Cell Viability Assay: HeLa cells were grown to 80% confluency before seeding in 200 µL at 5000 cells/well into a Corning Costar 96-well clear, flat bottom plate. Determination of cell viability was as per supplier's guidelines. The HeLa cells were incubated with the prodrug (PD) for 1 hour to allow internalization. Following this, the media was replaced, and Na[$AuCl_4$] (20 µM) was added twice a day for the subsequent 72 hours. The "twice a day" addition refers to the supplementation of the gold catalyst every 12 hours during the 72-hour incubation period. After incubation for 72 h, AlamarBlue solution (20 µL) was added followed by incubation for 6 h. Fluorescence was then measured at (λ_{ex} 530-560 nm, λ_{em} 590 nm). Cell viability was calculated as 100 x F_{cells} / $F_{control}$. **F** refers to the fluorescence of a given well.

1.6 Inductively Coupled Plasma Mass Spectrometry (ICP-MS) Method

Na[$AuCl_4$] (2mL, 50 μM) was added to a 6-well plate containing about 300 000 SKBR-3 cells. The plate was then incubated for 1h at 37 ºC, 5% CO_2. After incubation, the cells were washed with PBS buffer (2x 1mL) and lysed with HNO_3 68% (1 mL). A fraction of the lysate (500 μL) was added to a solution of HNO_3/H2O_2 (500 μL) and the mixture boiled at 180 ºC for cell digestion. The mixture was then transferred to an Eppendorf tube and centrifuged. A fraction of the supernatant (500 μL) was then added to an Eppendorf tube containing MQ water (500 μL) before analysis.[16]

1.7 Colon Cancer Zebrafish Xenograft Methods

Animal Care and Handling: In vivo experiments were performed in zebrafish model (Danio rerio) *Nacre*, which were maintained and handled in accordance with European Animal Welfare Legislation and Champalimaud Fish Platform Program.

Cell Culture: Human colon cancer cell line HCT116 was donated by Dr. Ângela Relógio from Charité Medical University of Berlin. Cells were cultured in filtered Dulbecco's Modified Eagle Medium (DMEM) High Glucose (Biowest) supplemented with 10% Fetal Bovine Serum (FBS) (Sigma-Aldrich) and 1% Penicillin-Streptomycin (P/S) 10,000 U/mL (Hyclone). Cells were maintained in an incubator (inCu Safe) with a humidified atmosphere containing 5% CO2 at 37°C.

Cell Staining: Cells at 70-80% confluence cultured in T-75 flask were washed with 1X Dulbecco's Phosphate Buffered Saline (DPBS) and stained in flasks with a fluorescent lipophilic dye diluted in DPBS 1X at 4 μL/mL (Vybrant CM- DiI; Molecular Probes, Life Technologies) for 15 minutes at 37°C. After this period, cells were dissociated with EDTA-DPBS 1X – EDTA 2mM, followed by mechanically detachment with cell scrapers. Cells were centrifuged for 4 min at 300g and re-suspended in complete cell culture medium. Trypan Blue Exclusion Method was performed to assess viability and counting. Cell suspension was centrifuged one last time for 4 minutes at 300 g and resuspended in 1X DPBS to a final concentration of 0.25×10^{6}cells/μL.

Zebrafish Xenograft Injection: Zebrafish larvae were anesthetized with Tricaine 1X and fluorescently labelled cancer cells were microinjected into the perivitelline space (PVS) of 48 hours post fertilization (hpf) anesthetized zebrafish larvae. After injection, xenografts were left on Embryonic (E3) medium (5 mM NaCl, 0.17 mM KCl, 0.33 mM $CaCl_2$, 0.33 mM $MgSO_4$, 10–5 % methylene blue) and maintained at 34°C until the

next day. At 24hpi, successful injected zebrafish xenografts were sorted into classes according to the tumor size. Xenografts were then randomly distributed into the different treatment groups.

Zebrafish Xenograft Drug Administration: Xenografts were randomly distributed in groups: DMSO (vehicle control), Na[$AuCl_4$] (15µM), PD1 (2nm), PD2 (1nm), PD1+ Na[$AuCl_4$] (2nm + 15µM) and PD2+Na[$AuCl_4$] (1nm + 15µm). PD1 and PD2 were re-suspended in DMSO and then diluted in E3 medium for 3 (4 dpi), consecutive days, being the media renewed daily, as well as the removal of dead xenografts.

Maximum Tolerated Concentration Assay: To assess the maximum tolerated concentration (MTC) of each drug in zebrafish larvae, a MTC assay was performed based in the concentrations used previously in the *in vitro* experiment. Groups of 50 zebrafish larvae with 3dpf were exposed to different drug concentrations during three consecutive days, being replaced every day. Toxicity was daily examined by counting the total number of dead larvae and checking the presence of cardiac edemas and curved tails.

Immunofluorescence: At the end of the assay, zebrafish xenografts were sacrificed with an overdose of Tricaine 25X and fixed in 4% formaldehyde (PFA) overnight, followed by storage in methanol at −20 °C. Antibodies used: primary antibody anti-caspase3 (rabbit CST; 1:100); secondary antibody Alexa goat anti-rabbit 650 (1:400); nuclei were counterstained with DAPI (1:100).

Imaging and Quantification: All images were obtained using a Zeiss LSM 710 fluorescence confocal microscope. Quantification was performed according to literature.[17]

Statistical Analysis: Statistical analysis was performed using the GraphPad Prism software version 8. All data were challenged by two normality tests – the D'Agostino & Pearson and the Shapiro-Wilk normality tests. A Gaussian distribution was only assumed for datasets that pass both normality tests and were analyzed by an unpaired t test with Welch's correction. By opposition, datasets that do not pass one or both normality tests were analyzed by Mann-Whitney test, an unpaired and non-parametric t test. For all the statistical analysis, P-value (P) is from a two-tailed test with a confidence interval of 95%. Statistical differences were considered significant whenever $P<0.05$ and statistical output was represented by stars as follows: non-significant (ns)>0.05, *≤0.05, **≤0.01, ***≤0.001 and ***≤0.0001. All the graphs presented the results as Avg ± standard error of the mean (SEM).

2. Supporting Data

2.1 ^{1}H-NMR Monitoring of Uncaging Reactions

Entry	Metal (Oxidation State)	Metal Complex/ Salt
**1**	Zinc(II) [Zn(II)]	$ZnSO_4.7H_2O$
**2**	Magnesium(II) [Mg(II)]	$MgCl_2.6H_2O$
**3**	Iron(II) [Fe(II)]	$FeSO_4.7H_2O$
**4**	Iron(III) [Fe(III)]	$Fe_2(SO_4)_3.9H_2O$
**5**	Cerium(IV) [Ce(IV)]	$Ce(NH_4)_2(NO_3)_6$
**6**	Copper(I) [Cu(I)]	$CuSO_4.5H_2O$ + THPTA
**7**	Copper(II) [Cu(II)]	$CuSO_4.5H_2O$
**8**	Ruthenium(III) [Ru(III)]	$RuCl_3.3H_2O$
**9**	Rhodium(II) [Rh(II)]	$Rh_2(AcO)_4$
**10**	Silver(I) [Ag(I)]	Ag_2CO_3
**11**	Palladium(0) [Pd(0)]	$Na_2[PdCl_4]$ + Na Ascorbate
**12**	Palladium(II) [Pd(II)]	$Na_2[PdCl_4]$
**13**	Platinum(II) [Pt(II)]	$K_2[PtCl_4]$
**14**	Gold(I) [Au(I)]	AuCl
**15**	Gold(III) [Au(III)]	$Na[AuCl_4]$

Supporting Table 1. List of metal salts/ complexes used in the study. Metal (O.S) mentioned throughout the manuscript refers to the corresponding salt/ complex mentioned here.

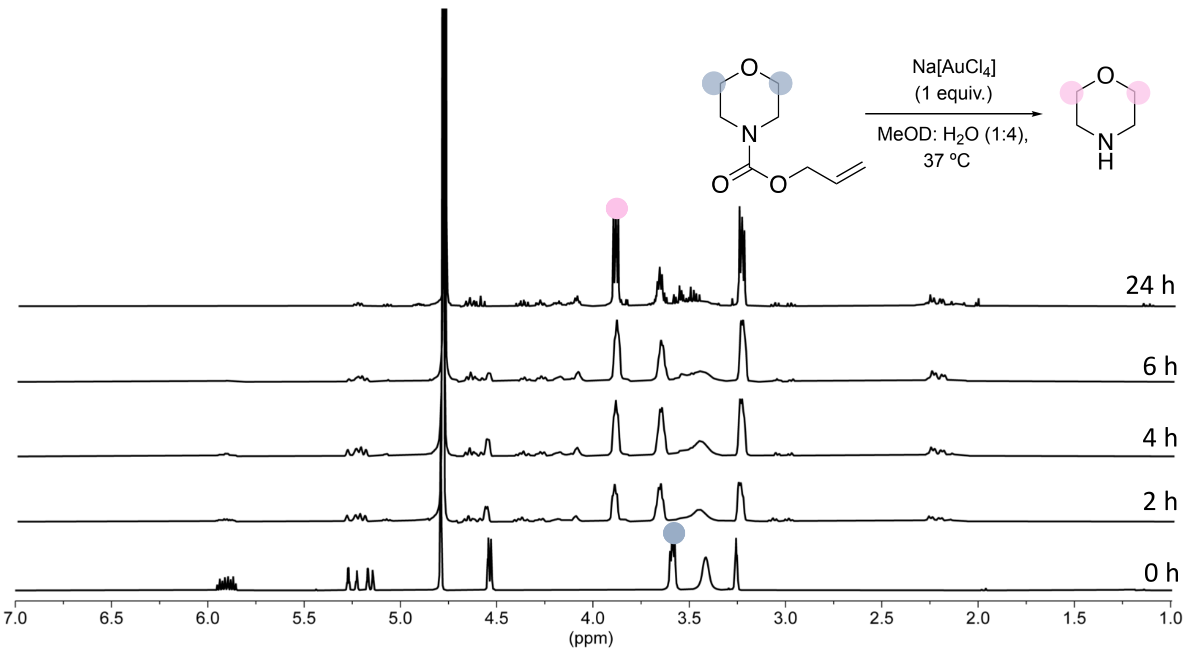

Supporting Figure 1. Uncaging of terminal allyl carbamate (**A**) monitored by ^{1}H-NMR with 1 equivalents of Na[AuCl$_4$].

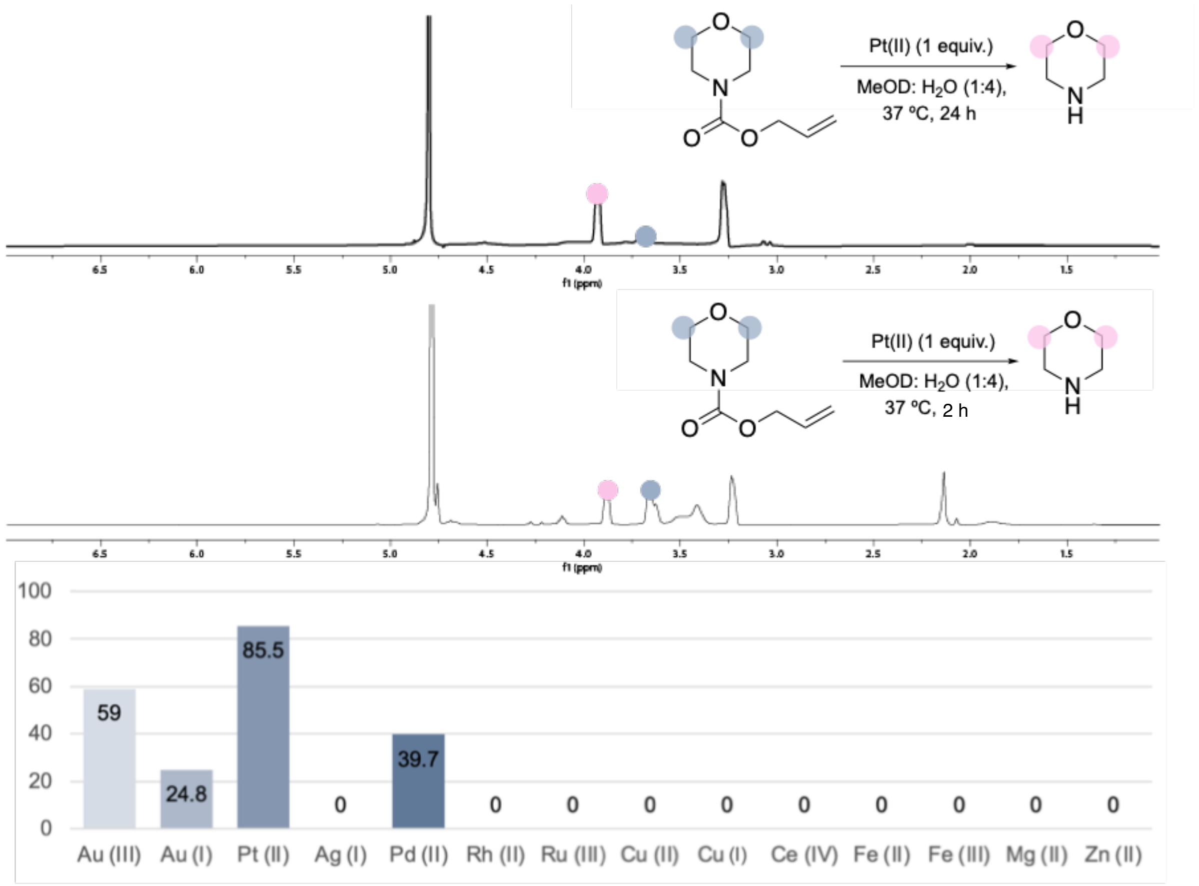

Supporting Figure 2. Uncaging of terminal allyl carbamate (**A**) monitored by ^{1}H-NMR with 1 equivalents of K_2PtCl_4. The reaction yielded 85 % conversions after 24 h at 37 ºC. A metal screen reveals that the reaction proceeds moderately with Au(III) [59 %] and Pd(II) [40%].

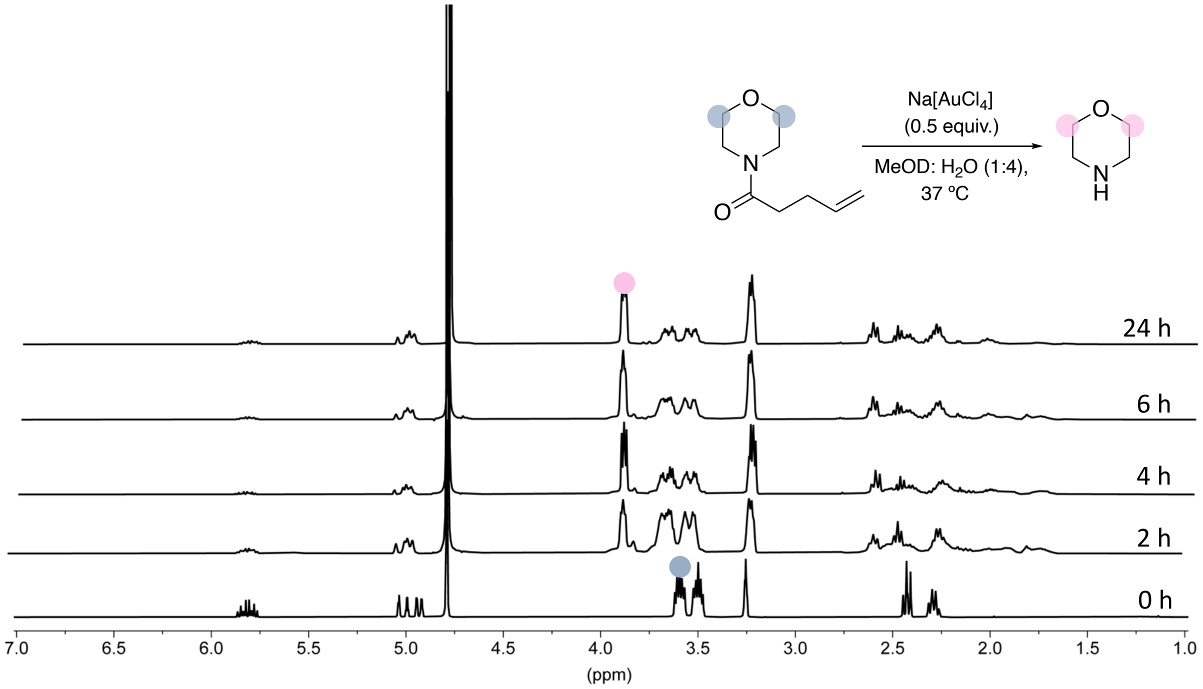

Supporting Figure 3. Uncaging of a pentenoic amide (**B**) monitored by ^{1}H-NMR with 0.5 equivalents of Na[AuCl$_4$]. The reaction yielded 62% conversions after 24 h at 37 ºC.

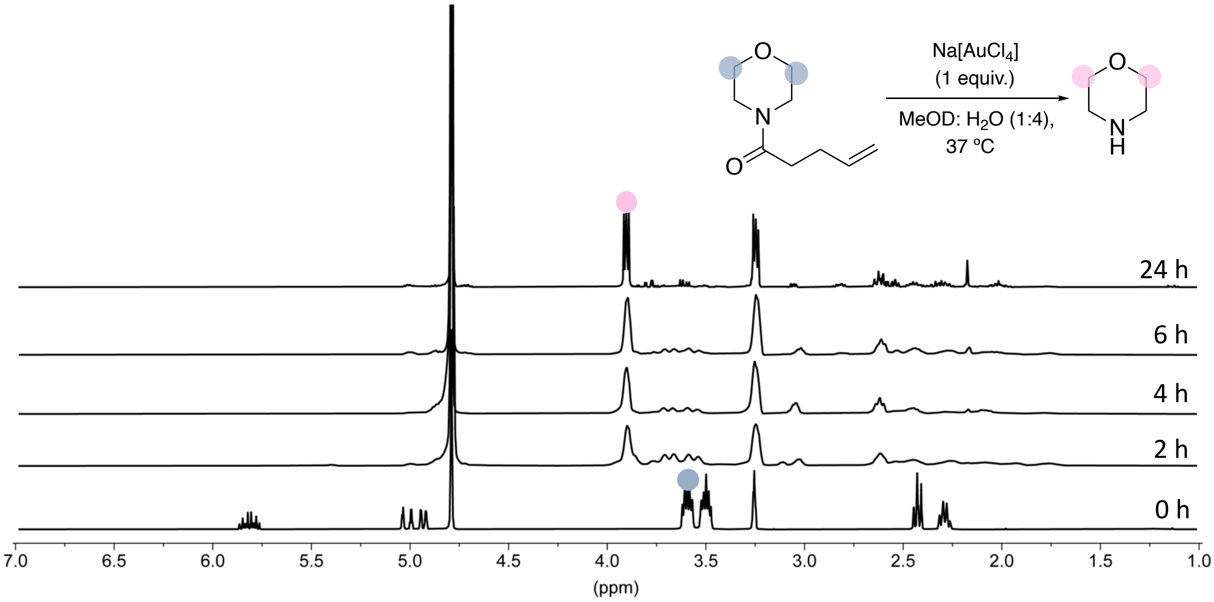

Supporting Figure 4. Uncaging of a pentenoic amide (**B**) monitored by ^{1}H-NMR with 1 equivalents of Na[AuCl$_4$].

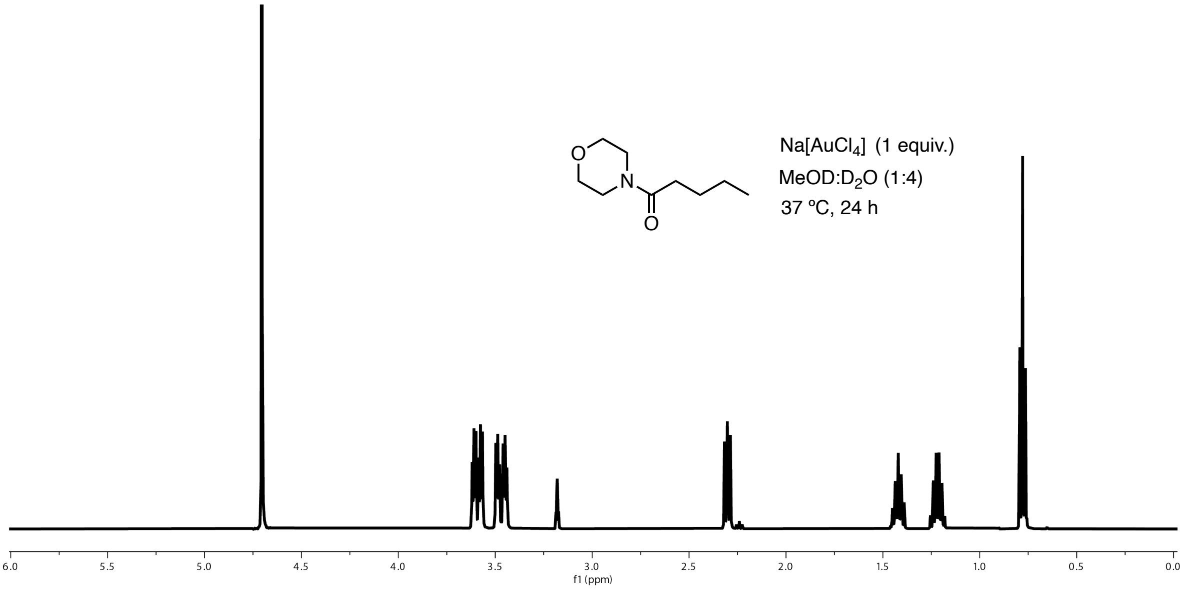

Supporting Figure 5. Stability study of aliphatic amide (**C**) monitored by ^{1}H-NMR with Na[AuCl$_4$]. Here, the free amine is not released under the uncaging conditions.

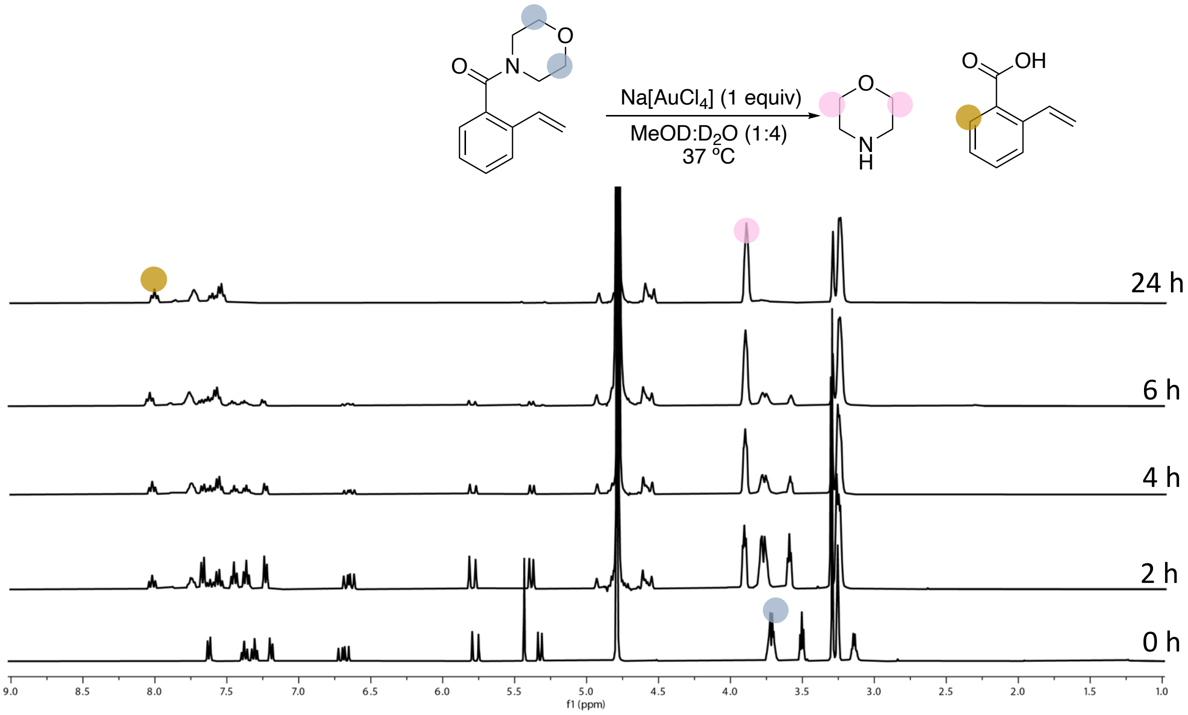

Supporting Figure 6. Uncaging of a 2-alkenyl benzamide moiety (**D**) monitored by ^{1}H-NMR with 1 equivalents of Na[AuCl$_4$]. The reaction yielded 95 % conversions after 24 h at 37 ºC. This shows that internal modifications within the linker can be tolerated.

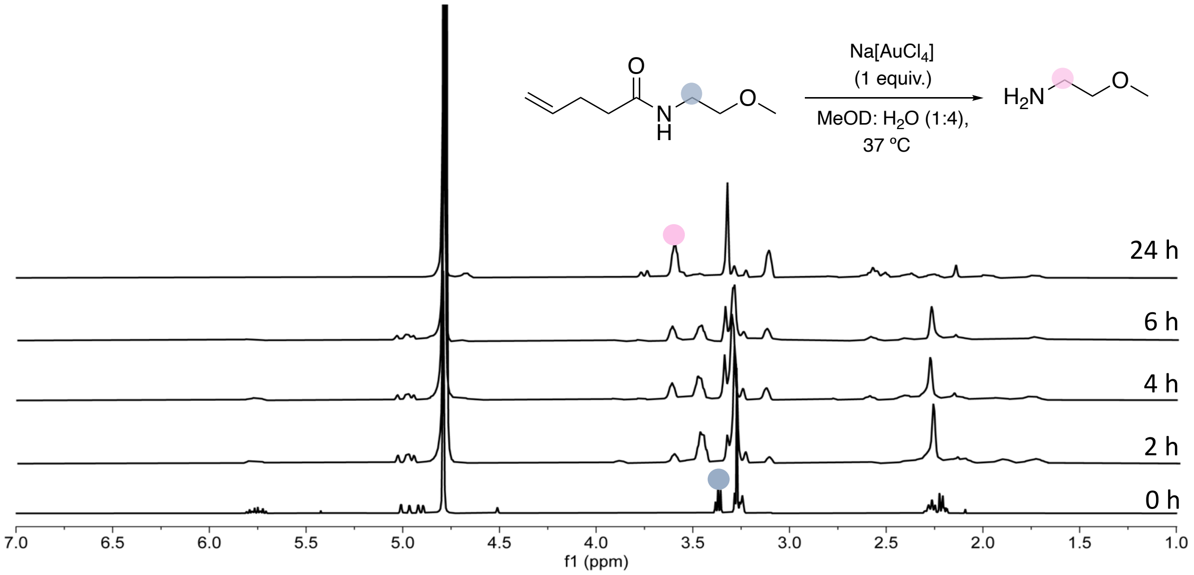

Supporting Figure 7. Uncaging of a pentenoic secondary amide (**E**) monitored by ^{1}H-NMR with 1 equivalents of Na[$AuCl_4$].

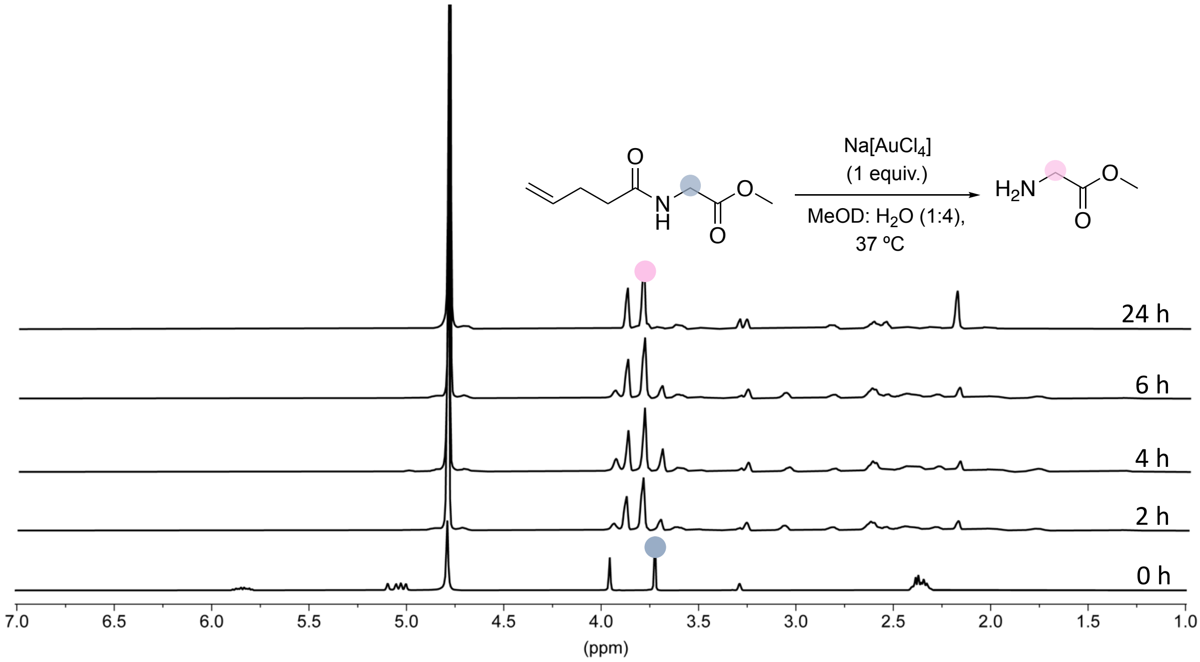

Supporting Figure 8. Uncaging of a pentenoic N-Glycine amide (**F**) monitored by ^{1}H-NMR with 1 equivalents of Na[$AuCl_4$].

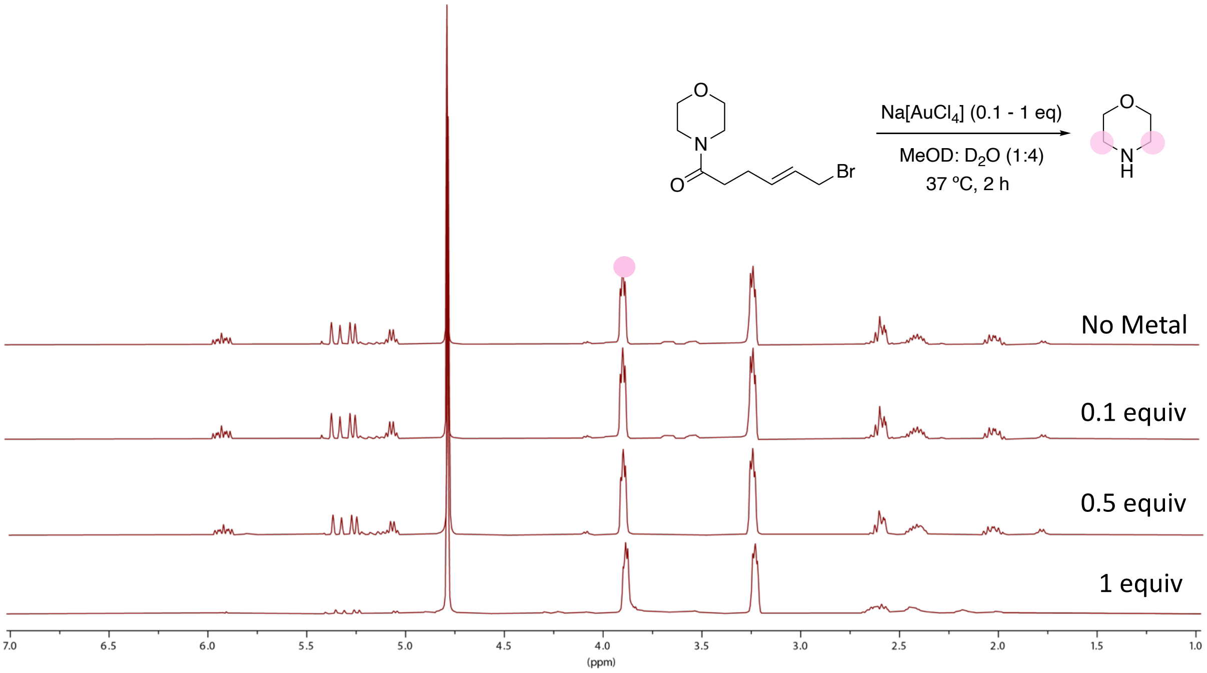

Supporting Figure 9. Uncaging of a pentenoic amide with allyl bromide as the leaving group (**G**) monitored by ^{1}H-NMR. The substrate was found to be unstable and release amine with no metal after 2 h at 37 °C.

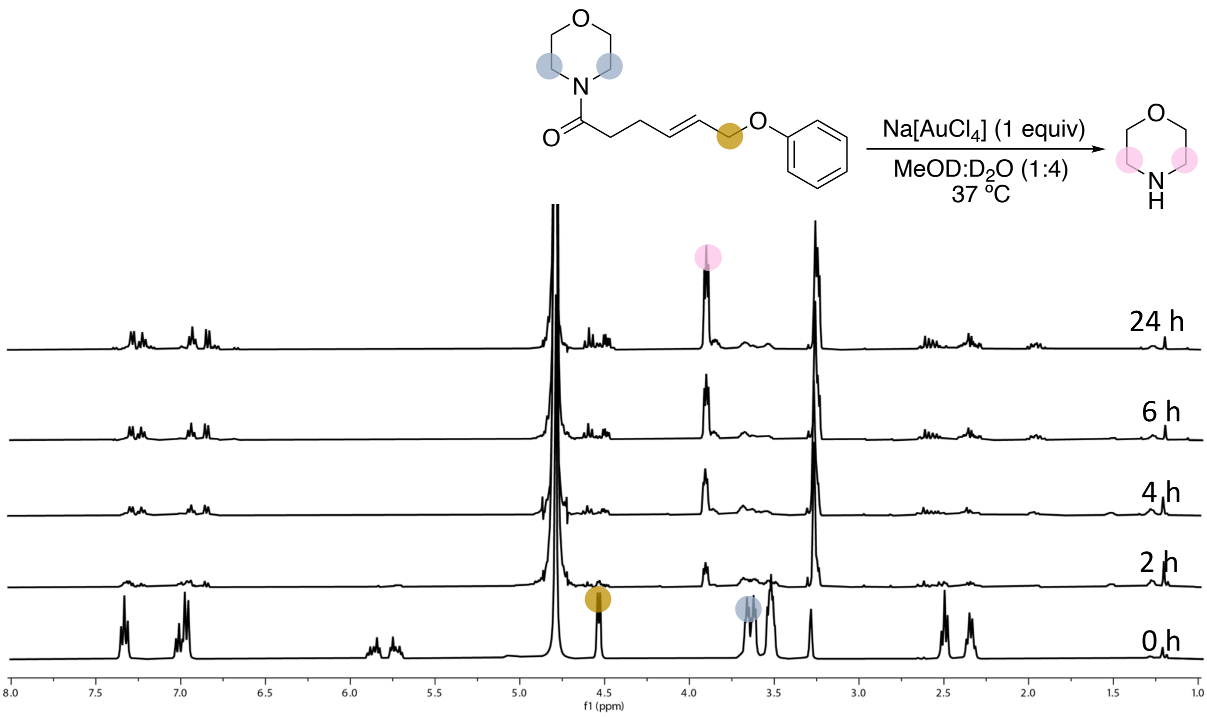

Supporting Figure 10. Uncaging of a pentenoic amide with phenol as the leaving group (**H**) monitored by ^{1}H-NMR with 1 equivalents of Na[AuCl$_4$]. A poorer leaving group allowed the generation of a stable substrate. Secondary amine release was observed, but phenol peaks were difficult to assign as the shifts were unclear.

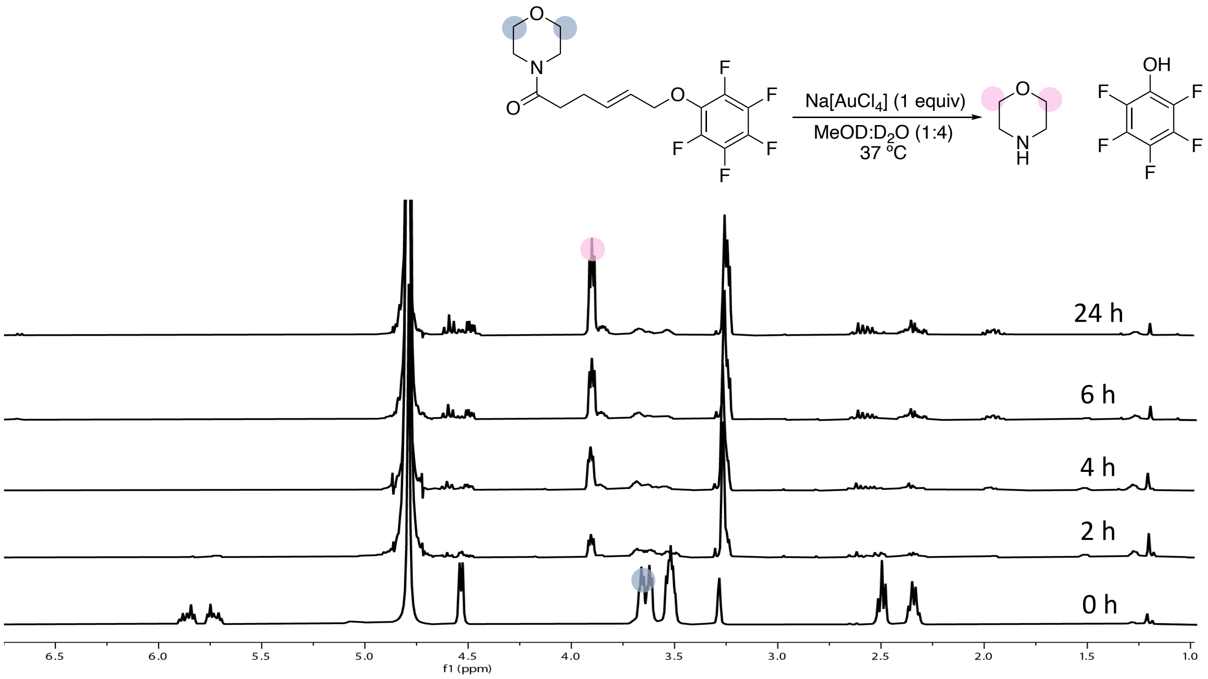

Supporting Figure 11. Uncaging of a pentenoic amide with pentafluorophenol as the leaving group (**I**) monitored by ^{1}H-NMR with 1 equivalents of Na[AuCl$_4$]. Secondary amine morpholine release was observed in high yields.

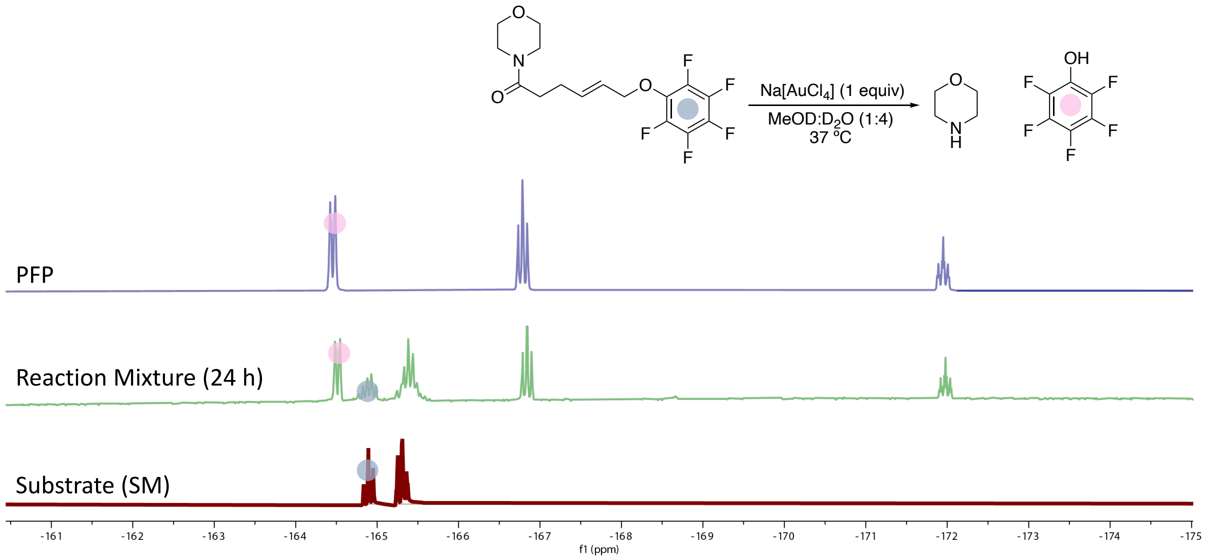

Supporting Figure 12. Uncaging of a pentenoic amide with pentafluorophenol as the leaving group (**I**) monitored by ^{19}F-NMR with 1 equivalents of Na[AuCl$_4$]. Pentafluorophenol release was cleanly observed.

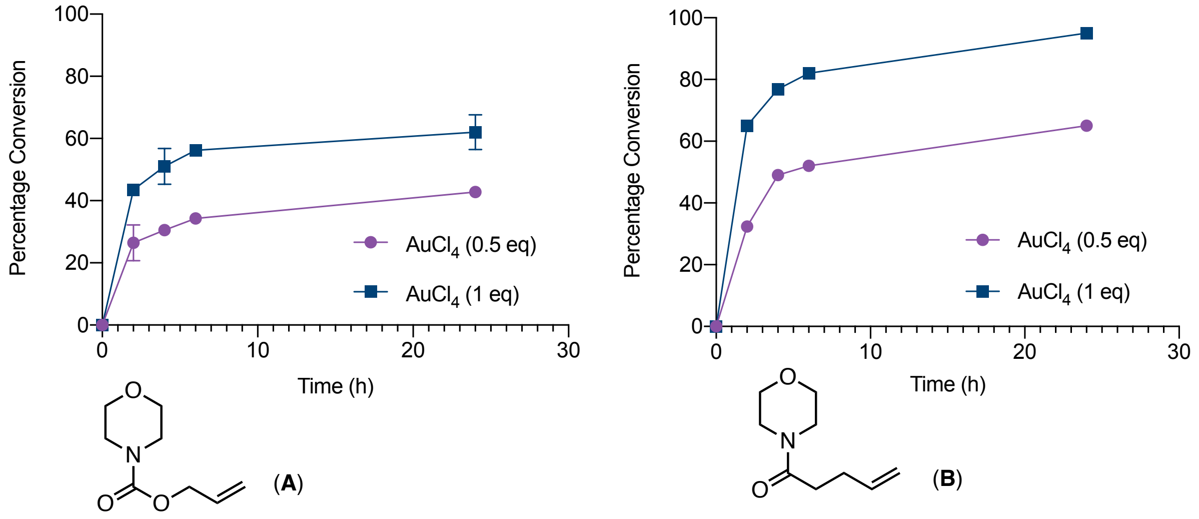

Supporting Figure 13. Summary of NMR kinetic studies with substrates **A** and **B**. The reaction proceeds upto 60% with 0.5 equiv. of Na[$AuCl_4$] after 24 h at 37 ºC. The analysis represents the average of three independent experiments. Error bars represent the SEM.

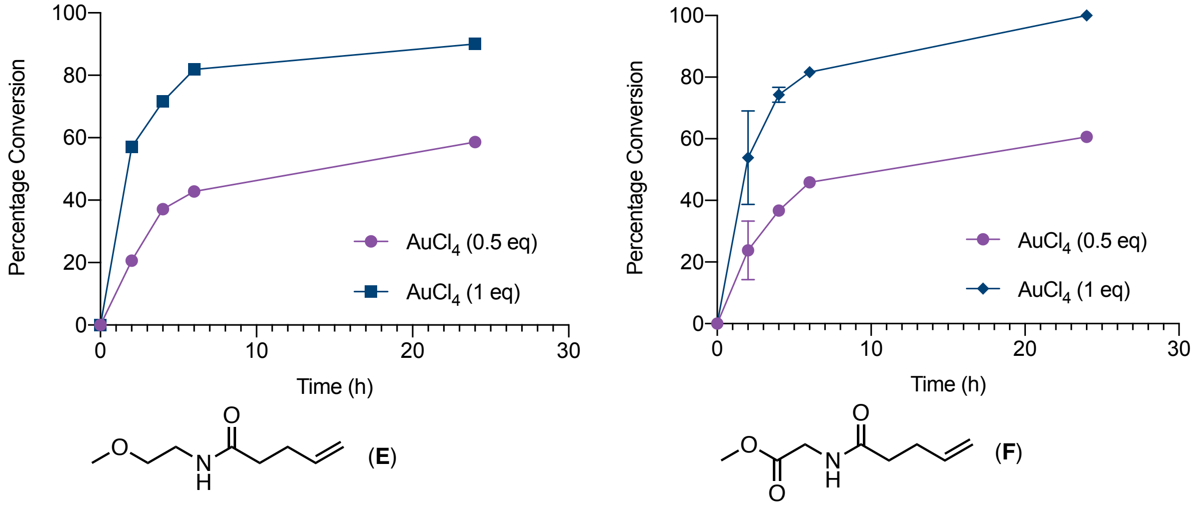

Supporting Figure 14. Summary of NMR kinetic studies with substrates **A** and **B**, **E** and **F**. The reaction proceeds upto 60% with 0.5 equiv. of Na[$AuCl_4$] after 24 h at 37 ºC. The analysis represents the average of three independent experiments. Error bars represent the SEM.

	Au (III)	Au (I)	Pt (II)	Pd (II)	Ag (I)	Rh (II)	Ru (III)	Cu (II)	Cu (0)	Ce (IV)	Fe (II)	Fe (III)	Mg (II)	Zn (II)
**A**	59	24.8	85.5	39.7	0	0	0	0	0	0	0	0	0	0
**B**	95	43.8	20	0	0	0	0	0	0	0	0	0	0	0
**C**	0	0	0	0	0	0	0	0	0	0	0	0	0	0
**D**	95	44.2	0	0	0	0	0	0	0	0	0	0	0	0
**E**	95	25	0	0	0	0	0	0	0	0	0	0	0	0
**F**	95	50	0	0	0	0	0	0	0	0	0	0	0	0
**G**	*	*	*	*	*	*	*	*	*	*	*	*	*	*
**H**	95	44.2	0	0	0	0	0	0	0	0	0	0	0	0
**I**	90	27	0	0	0	0	0	0	0	0	0	0	0	0

Supporting Table 2. Efficiency of the uncaging reaction under different conditions was assessed by ^{1}H NMR spectroscopy. The values provided here corresponds to the heat map (**Figure 2b**) in the main manuscript. *Substrate (**G**) was unstable and uncaged without any metal trigger.

2.2. Mechanistic and Kinetic Studies

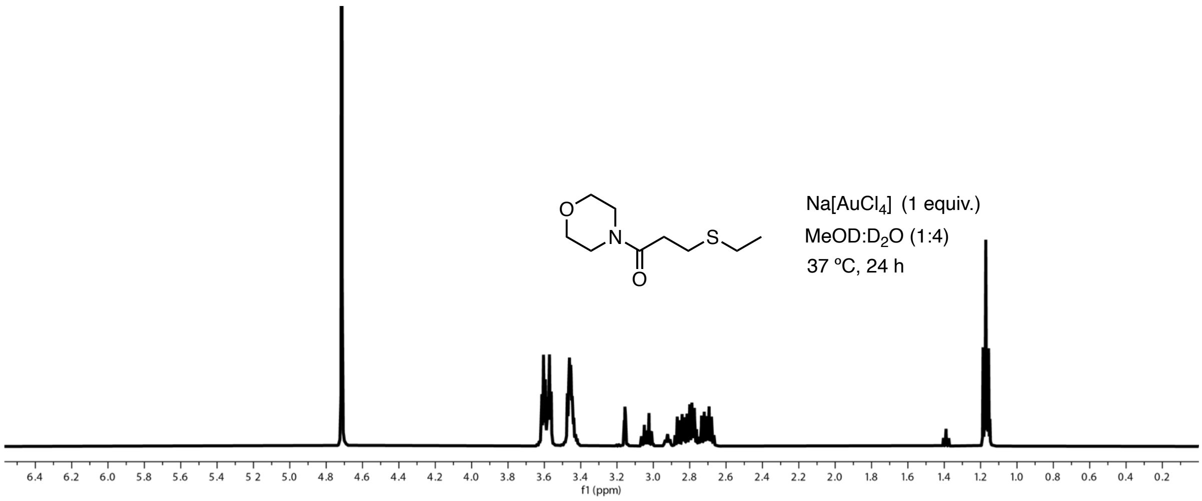

Supporting Figure 15. Control molecule with a thiol moiety at the γ-position from the carbonyl did not release morpholine (peaks between 3.3-3.7) under standard reaction conditions, possibly eliminating a mechanism involving a directing group to activate the carbonyl for hydrolysis.

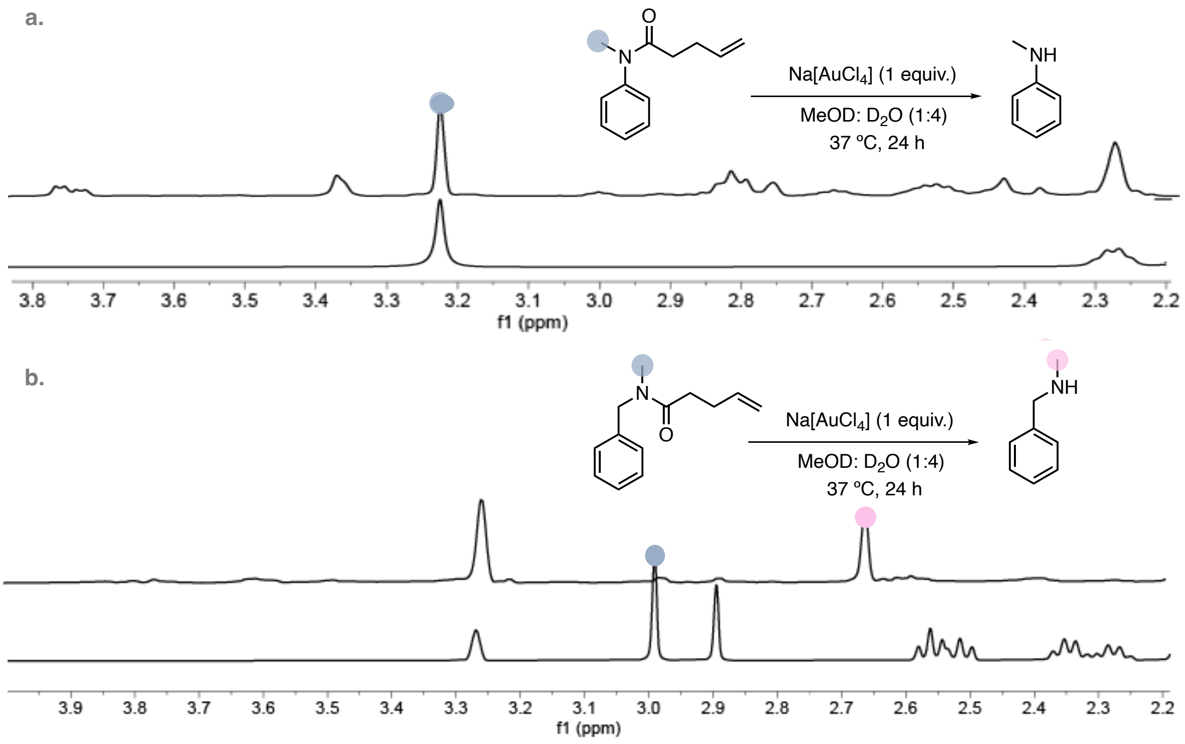

Supporting Figure 16. The reaction with phenyl-amide (**K**) did not proceed while benzyl-amide (**L**) released the free amine with stoichiometric amounts of Au(III), as assessed by NMR.

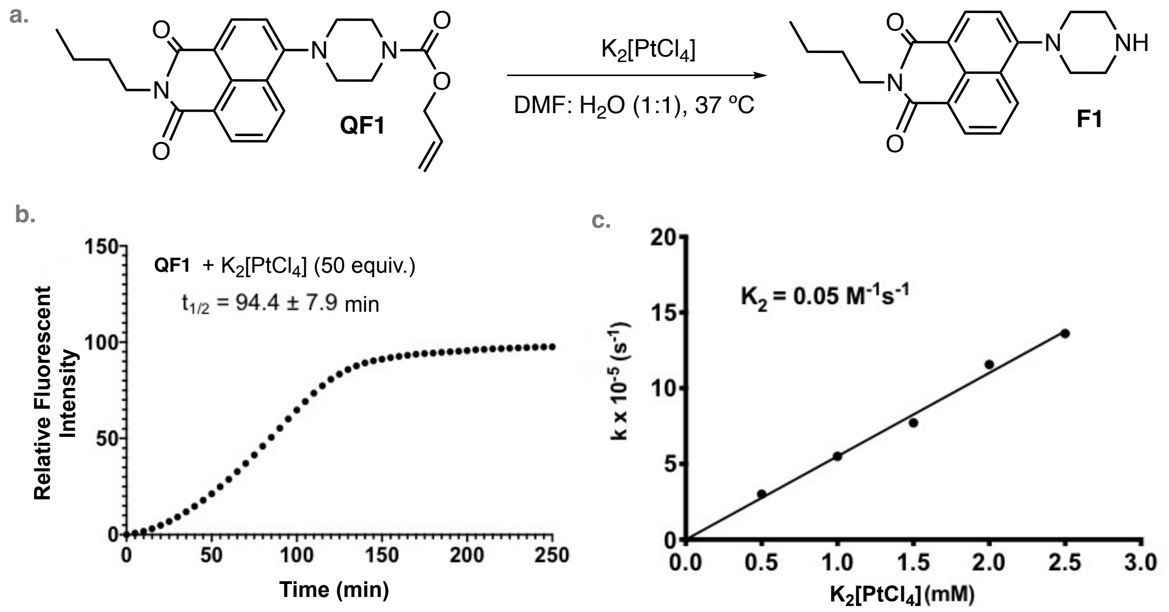

Supporting Figure 17. Fluorescence kinetic studies for carbamate uncaging reaction; (a) Scheme for uncaging fluorescent probe containing carbamate moiety **(b)** Changes in fluorescence intensity during the time course of the reaction with 50 equiv. of Au(III) **(c)** Calculation of rate constant using pseudo first order conditions.

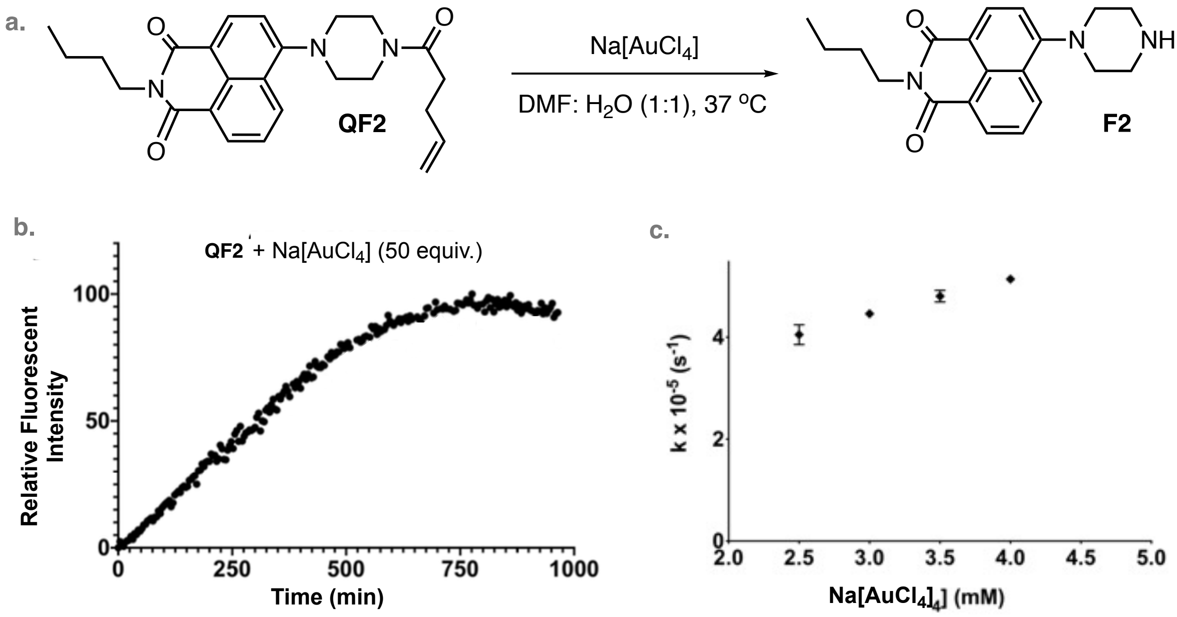

Supporting Figure 18. Fluorescence kinetic studies for amide uncaging reaction; (a) Scheme for uncaging fluorescent probe containing amide moiety **(b)** Changes in fluorescence intensity during the time course of the reaction with 50 equiv. of Au(III) **(c)** Calculation of rate constant using pseudo first order conditions.

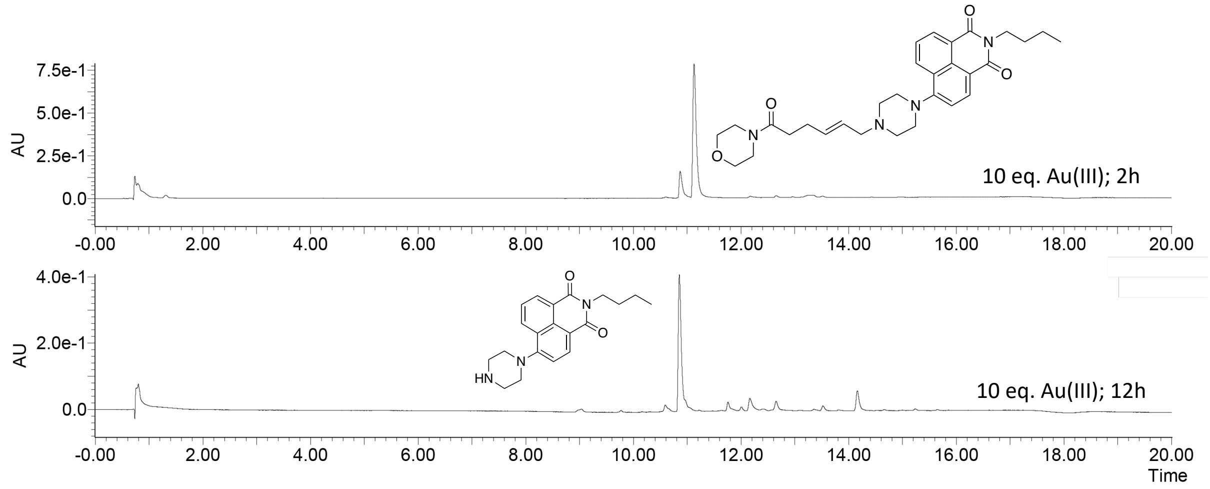

Supporting Figure 19. LC-MS trace of **QF3** and **QF3** + Na[$AuCl_4$]. A complete release of the **F3** was observed with 10 equivalence of Na[$AuCl_4$] at 37 ºC after 12 h. Retention time 11.2 corresponds to the starting material and 10.9 to the uncaged product.

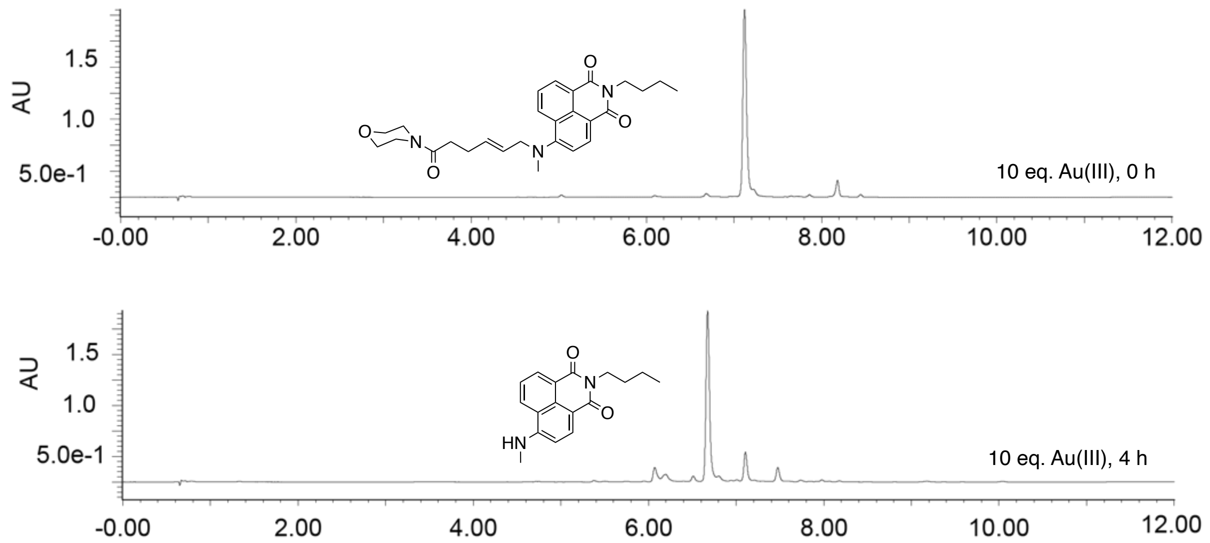

Supporting Figure 20. LC-MS of **QF4** and **QF4** + Na[$AuCl_4$]. A complete release of the **F4** was observed under standard reaction conditions. Retention time 7.2 corresponds to the starting material and 6.6 to the uncaged product.

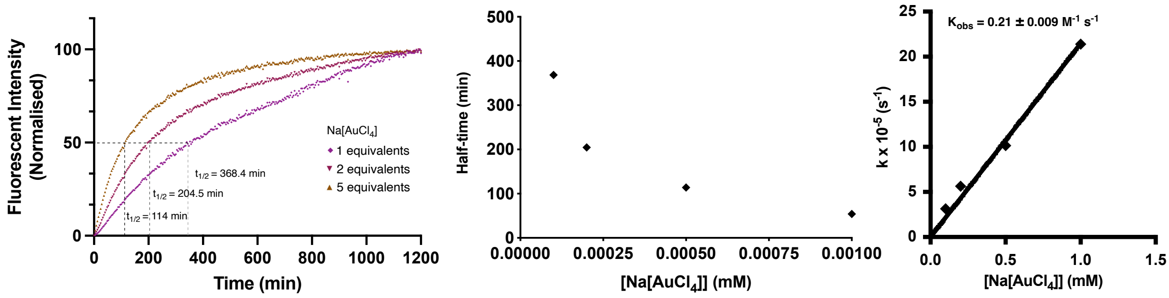

Supporting Figure 21. The plot shows the reaction rate of the payload release as a function of Na[AuCl4] concentration. Increasing the equivalents of Na[AuCl4] results in faster reaction rates, demonstrating the dependence of reaction kinetics on metal concentration. Kinetic experiments were conducted, and the data were fitted to a first-order kinetic model, yielding a rate constant of 0.21 ± 0.009 M^{-1} s^{-1}.

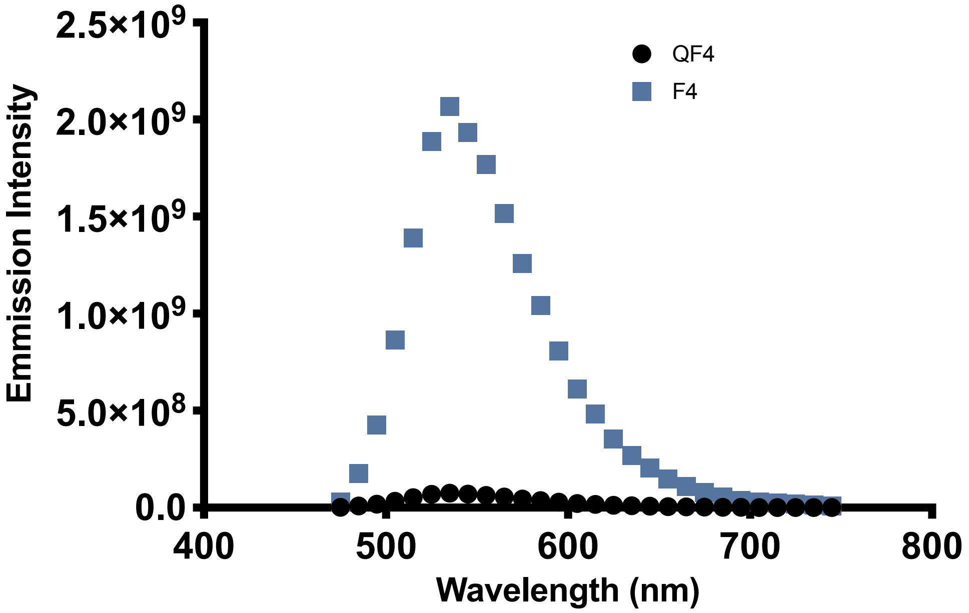

Supporting Figure 22. Emmission spectrum of **QF4** and **F4**.The uncaged fluorophore is 28 fold more fluorescent that the quenched version (λ_{ex} = 445 nm, λ_{em} = 535 nm).

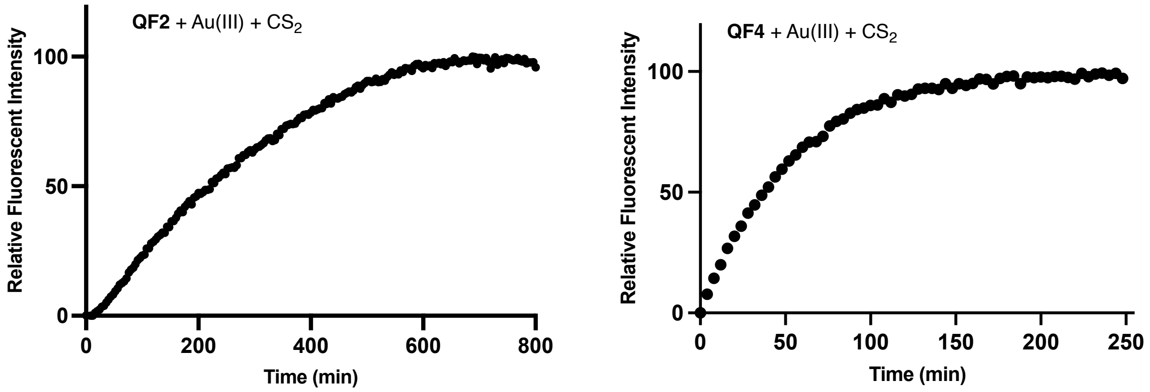

Supporting Figure 23. CS_2 acts as a catalyst poison for homogeneous and heterogeneous Au(I) reactions, although Au(III) species are unaffected. However, the reaction rates are unaffected with CS_2 suggestinga possible non-involvement of Au(I) species in the reaction.

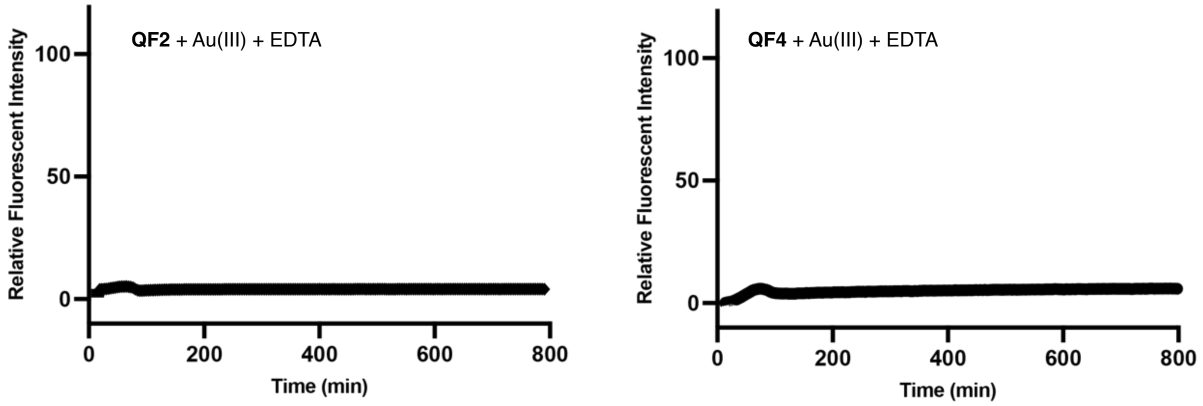

Supporting Figure 24. The reaction was significantly affected by the addition of ethylenediamine tetraacetic acid, possibly due to the participation of Au(III) in the reaction. CS_2.

2.3 Quantum mechanical calculations

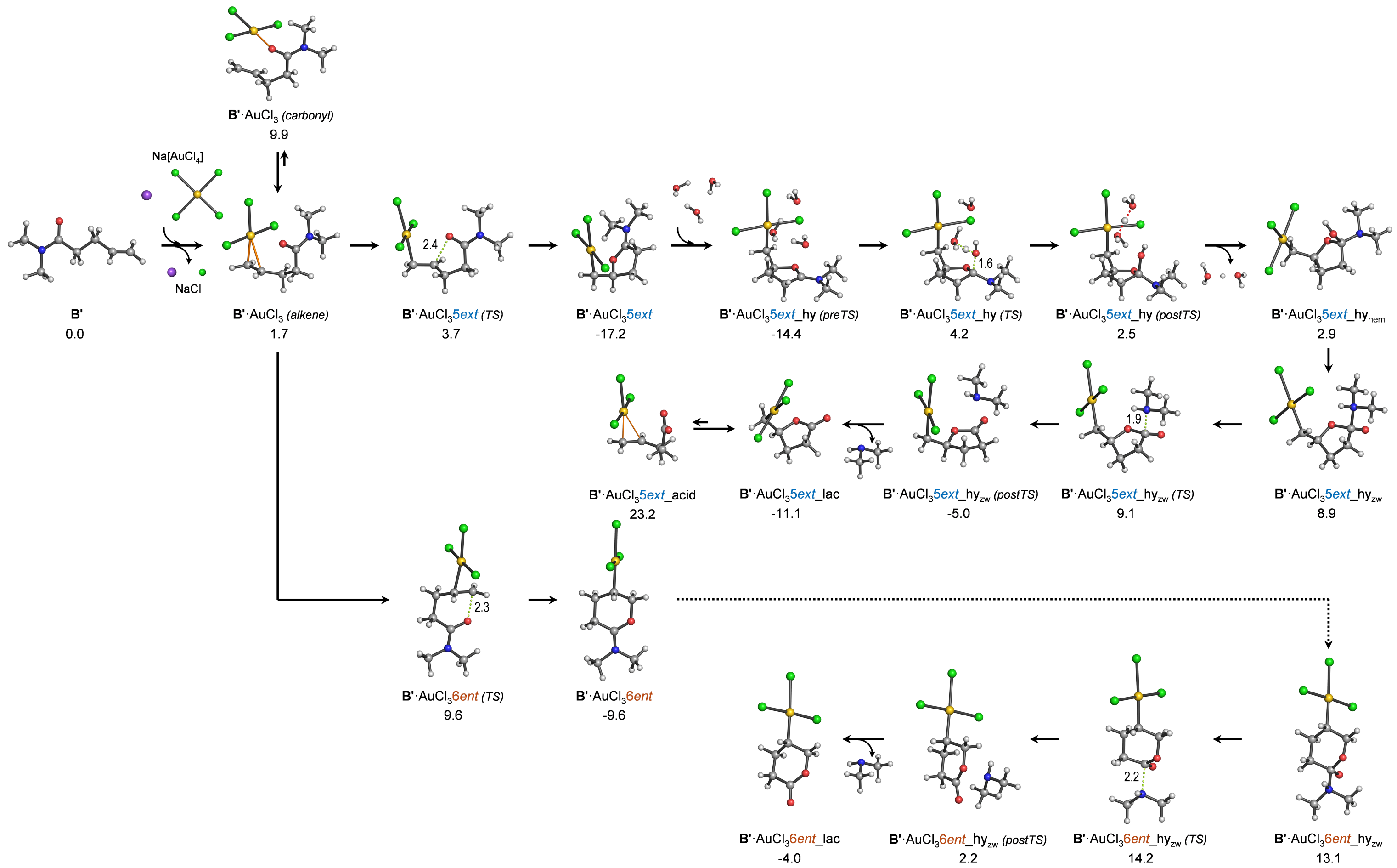

Supporting Figure 25. Lowest-energy structures calculated with SMD(H_2O)/M06/6-311+G(d,p)+SDD(Au) level for amide decaging (single-release) from model compound **B'** catalyzed by Na[$AuCl_4$] in water. When two chiral centers are generated, only the most stable diastereomer is reported, irrespective of its absolute configuration. Breaking/forming bonds are represented with green dotted lines. Hydrogen bonds are represented with red dotted lines. π(Au–C) and σ(Au–O) bonds are represented with orange solid lines. Distances are given in angstrom. Free energies are given in kcal mol^{-1}.

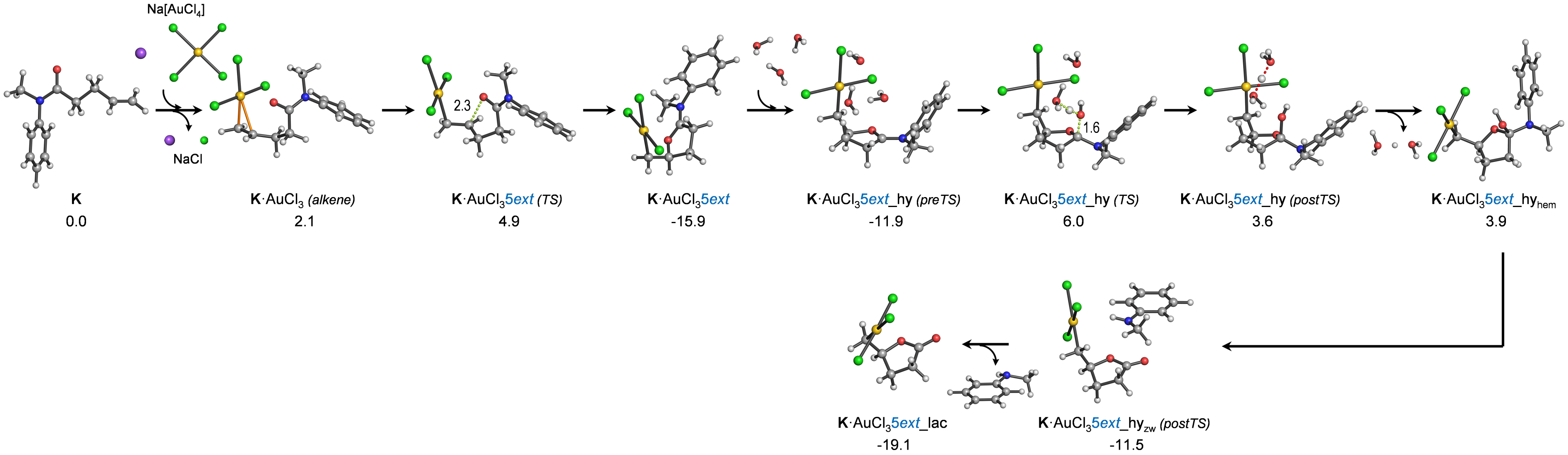

Supporting Figure 26. Lowest-energy structures calculated with SMD(H_2O)/M06/6-311+G(d,p)+SDD(Au) level for amide decaging (single-release) from model compound **K** catalyzed by Na[$AuCl_4$] in water. When two chiral centers are generated, only the most stable diastereomer is reported, irrespective of its absolute configuration. Breaking/forming bonds are represented with green dotted lines. Hydrogen bonds are represented with red dotted lines. π(Au–C) bonds are represented with orange solid lines. Distances are given in angstrom. Free energies are given in kcal mol^{-1}.

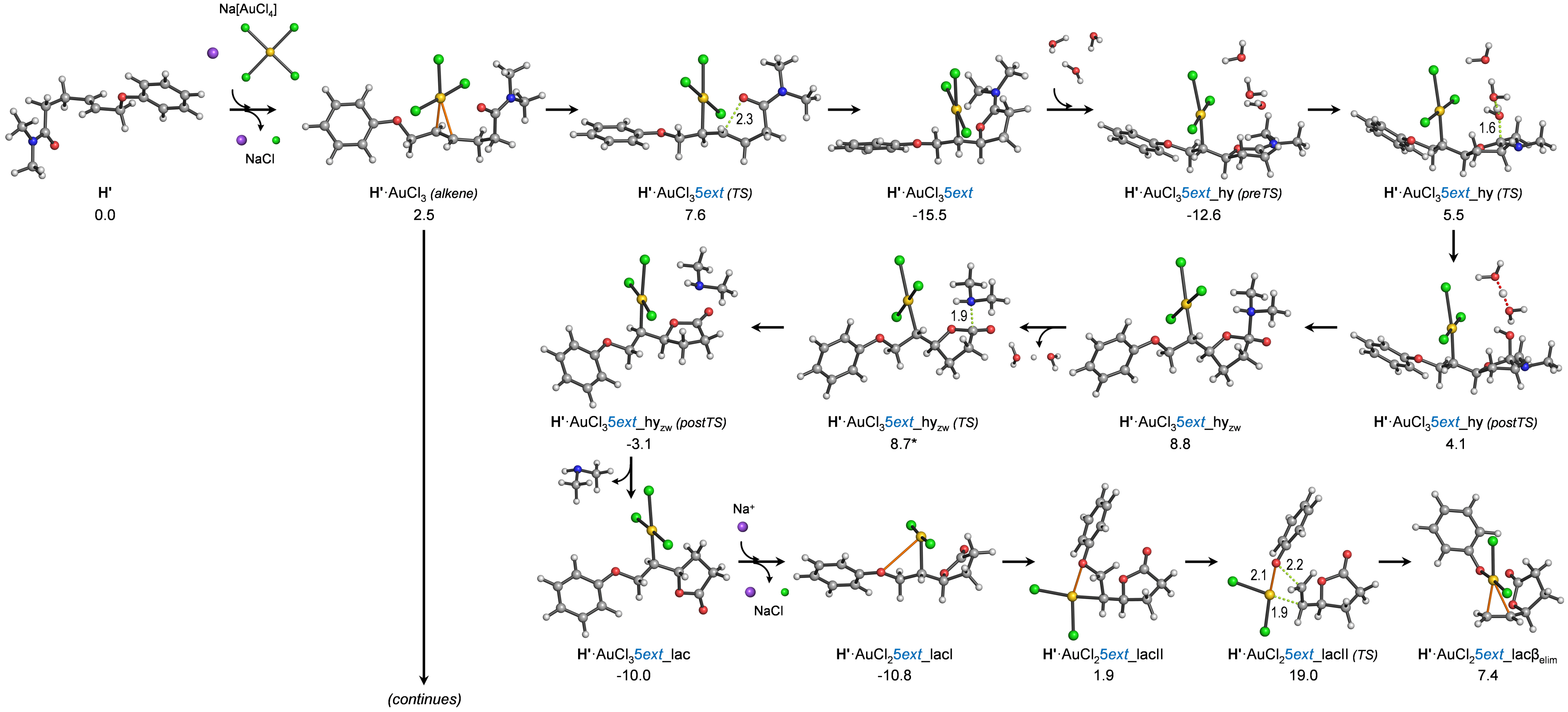
Na[AuCl4]
NaCl
2.3
1.6
1.9
2.1
2.2
1.9
Na+
NaCl
H'
0.0
H'·AuCl3 (alkene)
2.5
H'·AuCl35ext (TS)
7.6
H'·AuCl35ext
-15.5
H'·AuCl35ext_hy (preTS)
-12.6
H'·AuCl35ext_hy (TS)
5.5
H'·AuCl35ext_hyzw (postTS)
-3.1
H'·AuCl35ext_hyzw (TS)
8.7*
H'·AuCl35ext_hyzw
8.8
H'·AuCl35ext_hy (postTS)
4.1
H'·AuCl35ext_lac
-10.0
H'·AuCl25ext_lacI
-10.8
H'·AuCl25ext_lacII
1.9
H'·AuCl25ext_lacII (TS)
19.0
H'·AuCl25ext_lacβelim
7.4
(continues)

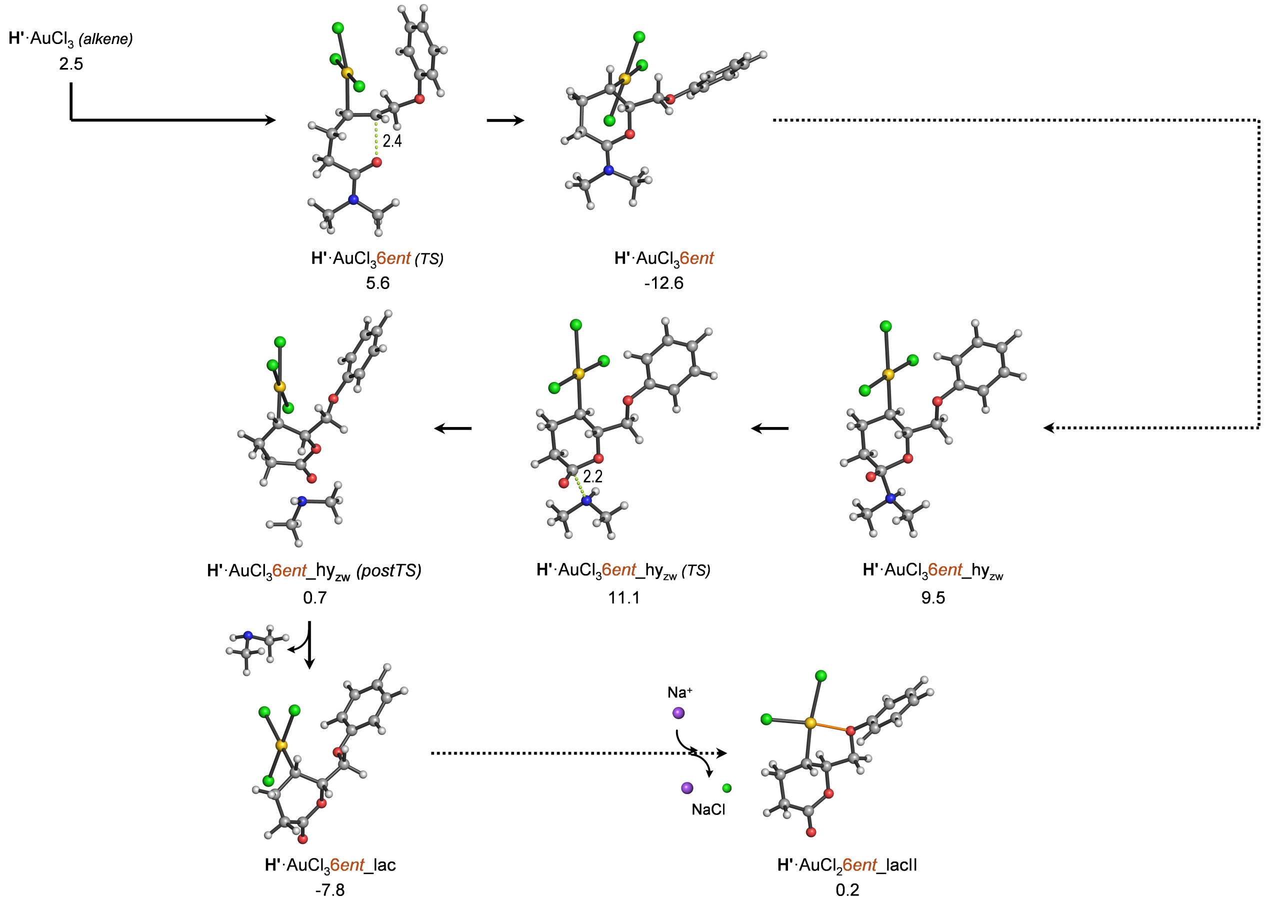

Supporting Figure 27. Lowest-energy structures calculated with SMD(H_2O)/M06/6-311+G(d,p)+SDD(Au) level for amide and allyl leaving group decaging (dual-release) from model compound **H'** catalyzed by Na[$AuCl_4$] in water. When two chiral centers are generated, only the most stable diastereomer is reported, irrespective of its absolute configuration. Breaking/forming bonds are represented with green dotted lines. Hydrogen bonds are represented with red dotted lines. π(Au–C) and σ(Au–O) bonds are represented with orange solid lines. Distances are given in angstrom. Free energies are given in kcal mol^{-1}. Asterisks denote virtually barrierless steps in which the transition state is equal or even lower in free energy than the preceding intermediate due to numeric errors in the estimation of vibrational entropy.

2.4. Peptide Bond Cleavage Reactions

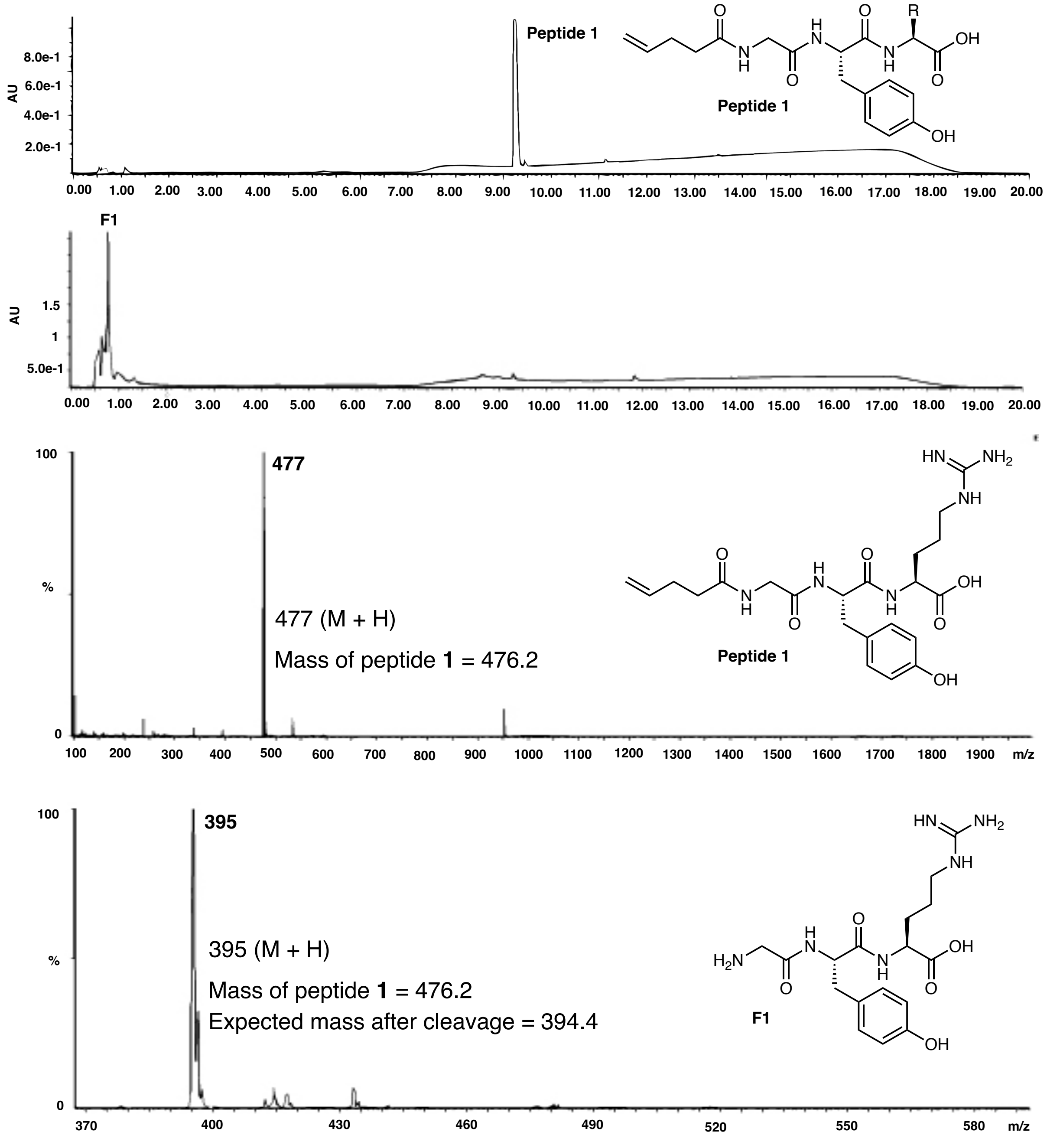

Supporting Figure 28. Model peptide (Pentenoic amide-G-Y-R) (**Peptide 1**). Bond-cleavage reaction at the N-terminus of a peptide masked with pentenoic amide. The reaction is triggered with Na[AuCl$_4$] at 37 °C in H_2O. LC-MS data that shows the fragment (**F1**) after 30 minutes. The UV-trace indicates full consumption of the starting material to the uncaged fragment.

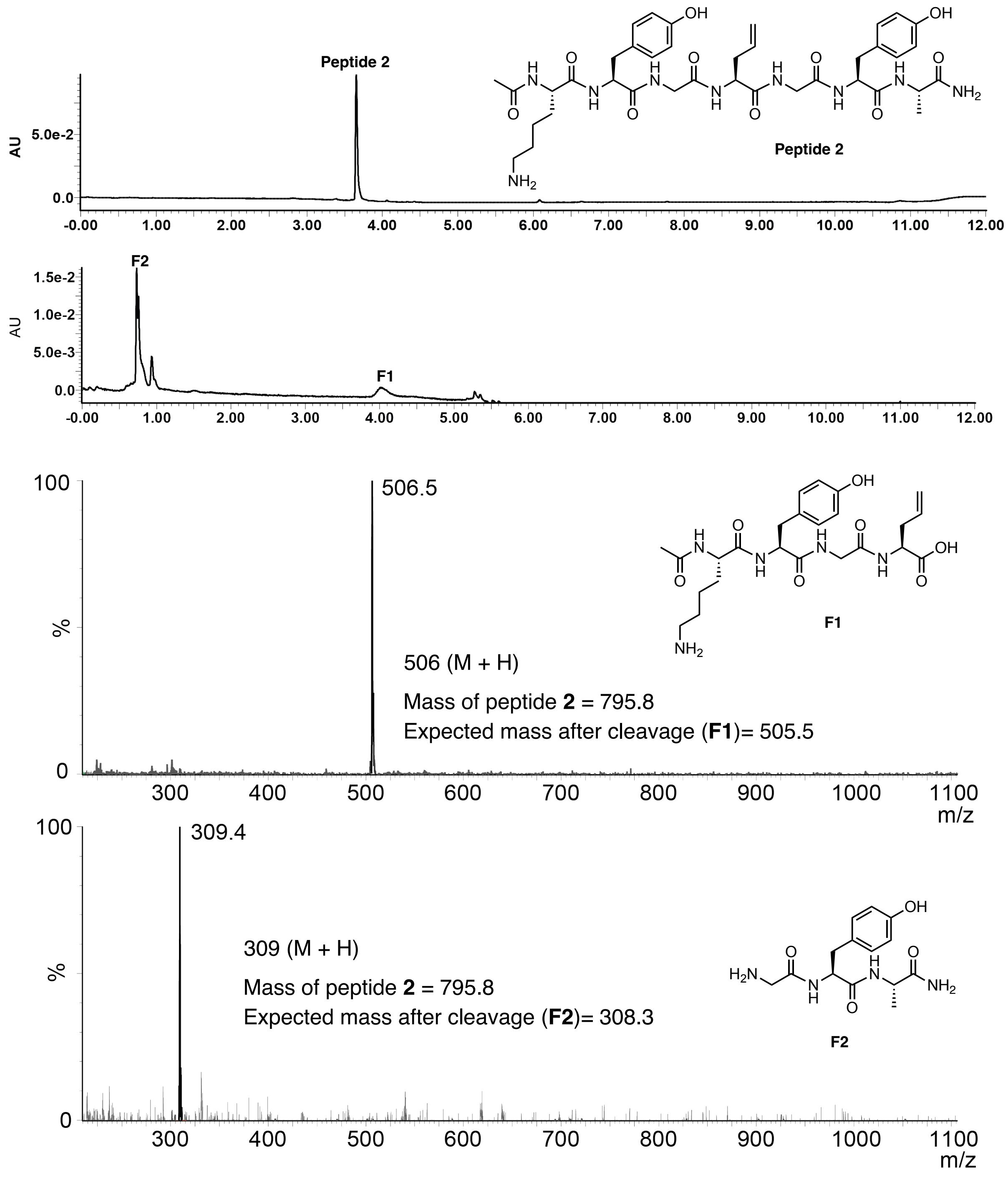

Supporting Figure 29. Model peptide (K-Y-G-AllylGlycine-G-Y-A) (**Peptide 2**). Bond-cleavage reaction at allyl glycine with Na[AuCl$_4$] at 37 °C in H_2O. LC-MS data that shows the fragments (**F1** and **F2**) after 30 minutes. The UV-trace indicates full consumption of the starting material to degraded fragments.

2.5. Cell Studies

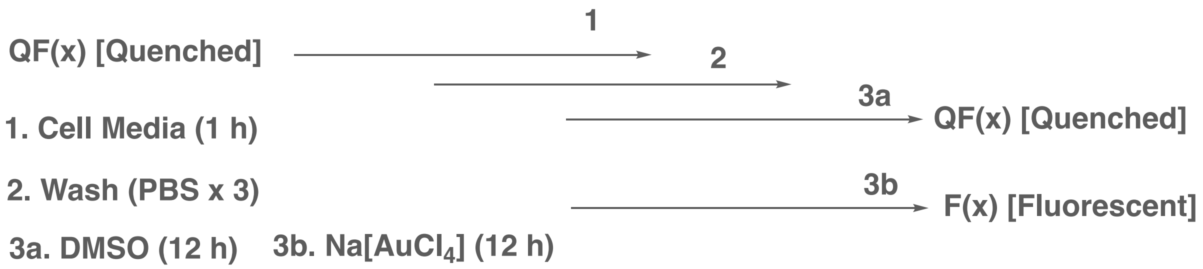

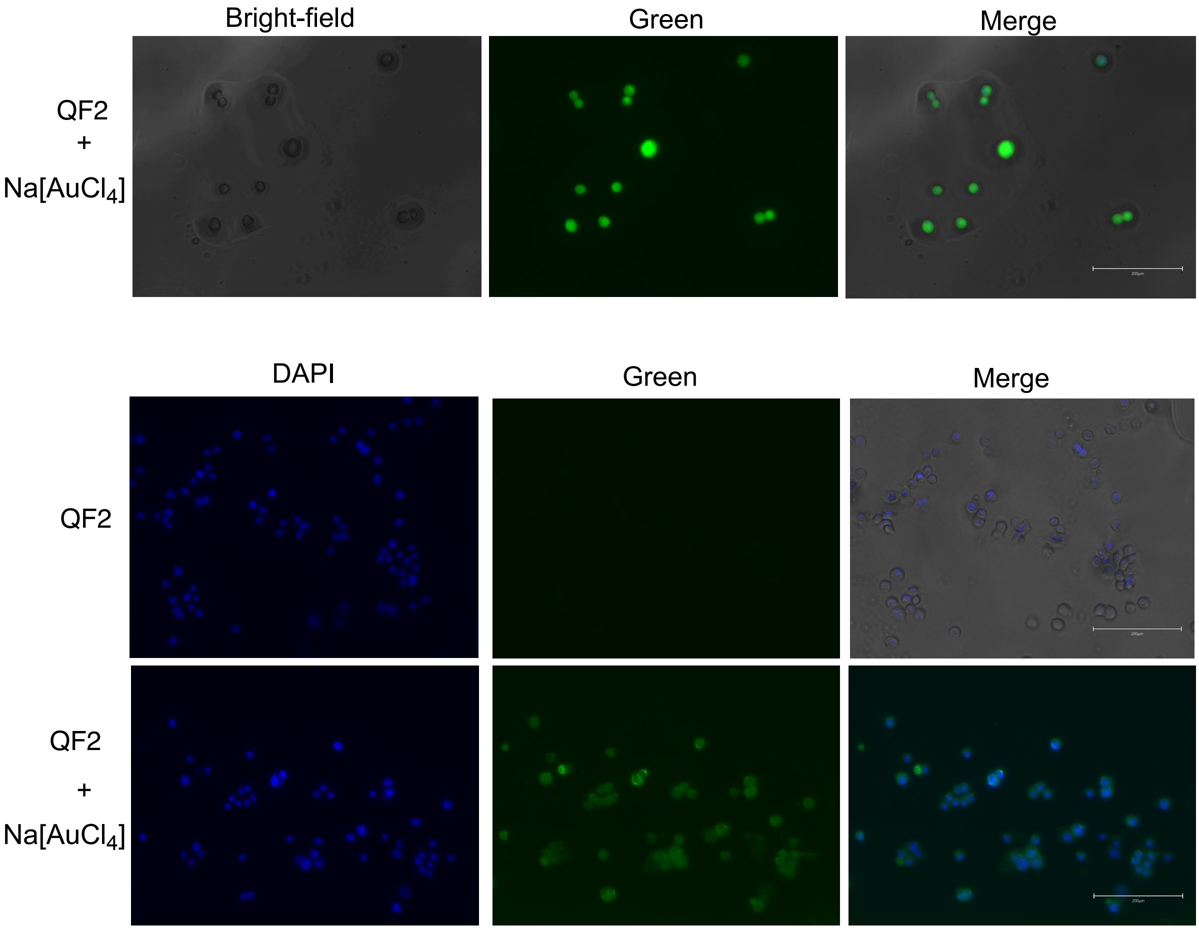

Supporting Figure 30. Au(III)-mediated uncaging of pentenoic amide fluorophore (**QF2**) in SKBR3 breast cancer cells followed by epifluorescence microscopy. DAPI EVOS light cube was used to image nuclei stained by Hoechst 33342. Images of SKBR3 cells incubated for 1 h with caged **QF2** followed by a wash. The cells were randomly distributed into two conditions: DMSO or Na[$AuCl_4$] [Au(III)] for 12 h. The caged naphthalimide derivatives exhibits high stability in cells (Panel 2, green channel) and their quenched fluorescence could be reactivated upon removal of the caging group (λ_{ex} = 445 nm, λ_{em} = 545 nm). An increase in fluorescence was observed in cells treated with caged fluorophore and Na[$AuCl_4$] [Au (III)] (Panel 1 and Panel 3, green channel) demonstrating that the reaction works in cells.

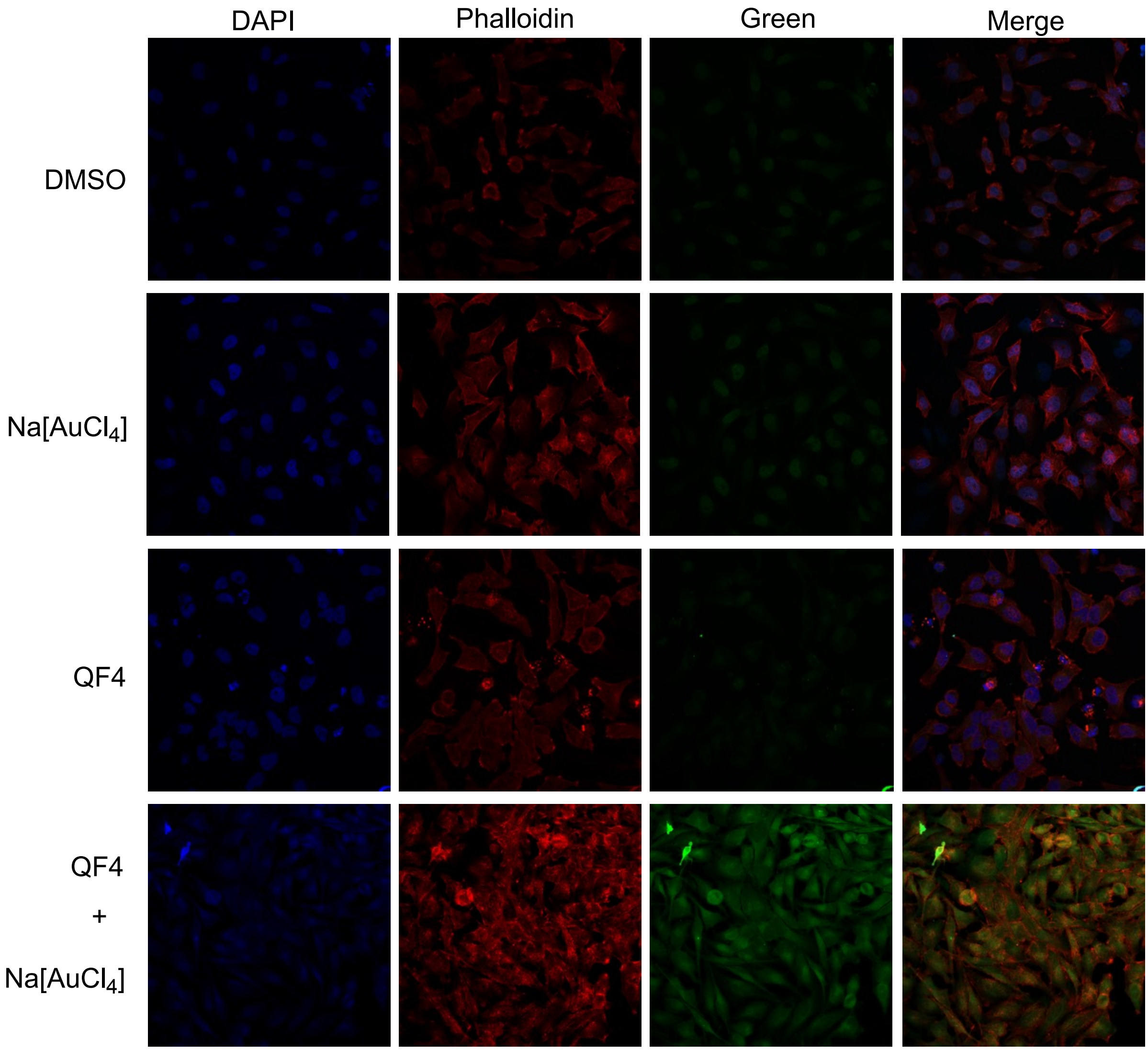

Supporting Figure 31. Au(III)-mediated uncaging of pentenoic amide fluorophore (**QF4**) in HeLa cells followed by confocal microscopy. DAPI EVOS light cube was used to image nuclei stained by Hoechst 33342. Images of HeLa cells incubated for 1 h with caged **QF4** followed by a wash. The cells were randomly distributed into two conditions: DMSO or Na[$AuCl_4$] [Au(III)] for 12 h. The caged naphthalimide derivatives exhibits high stability in cells (Panel 3, green channel) and their quenched fluorescence could be reactivated upon removal of the caging group (λ_{ex} = 445 nm, λ_{em} = 545 nm). An increase in fluorescence was observed in cells treated with caged fluorophore and Na[$AuCl_4$] [Au (III)] (Panel 4, green channel) demonstrating that the reaction works *in cells*.

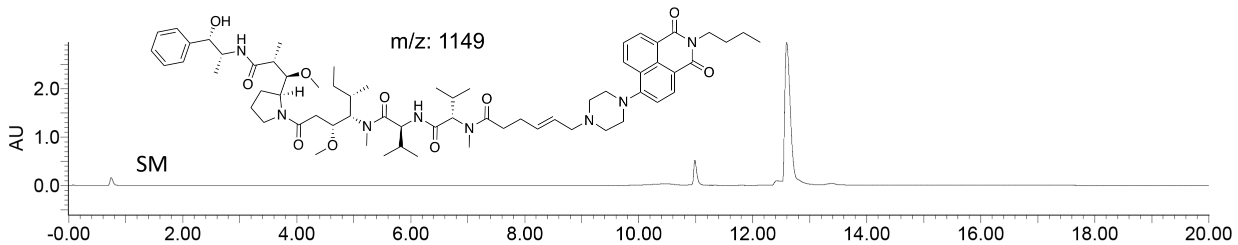

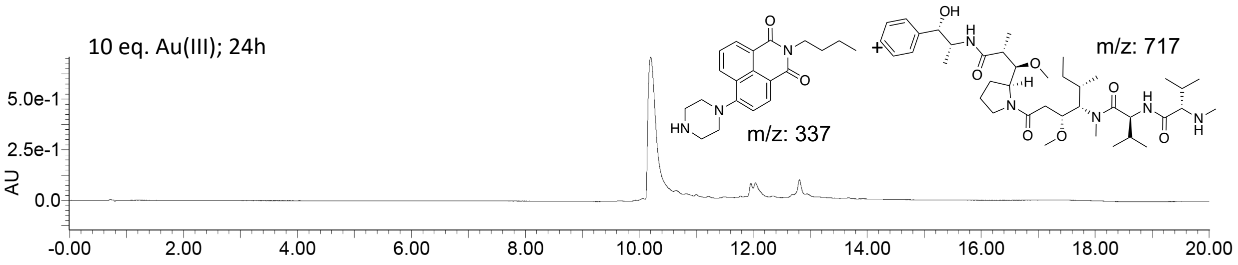

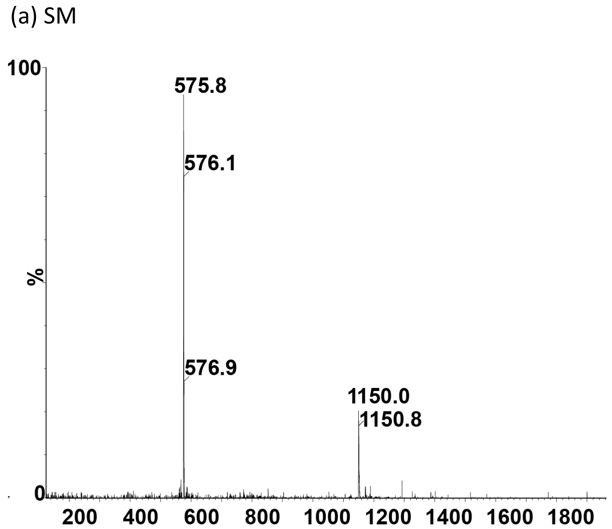

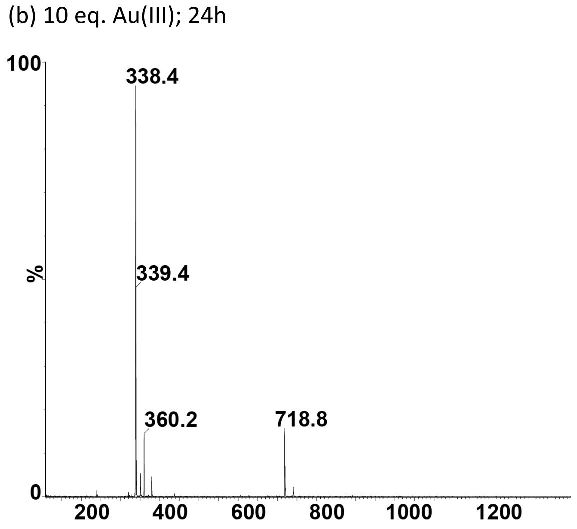

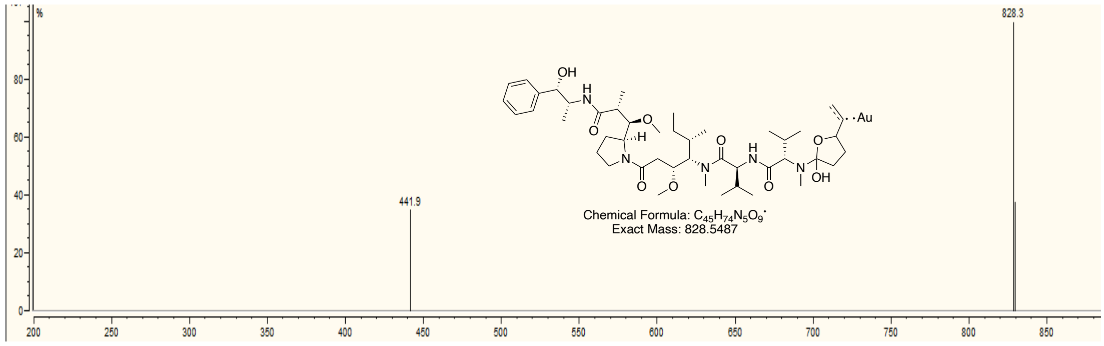

Supporting Figure 32. LC-MS spectrum for the reaction of a fluorescent probe (**FQ5**) with Na[AuCl$_4$] (10 equivalents). Retention time 12.3 corresponds to the starting material and 10.1 to the uncaged products. A possible intermediate was observed at 2 h.

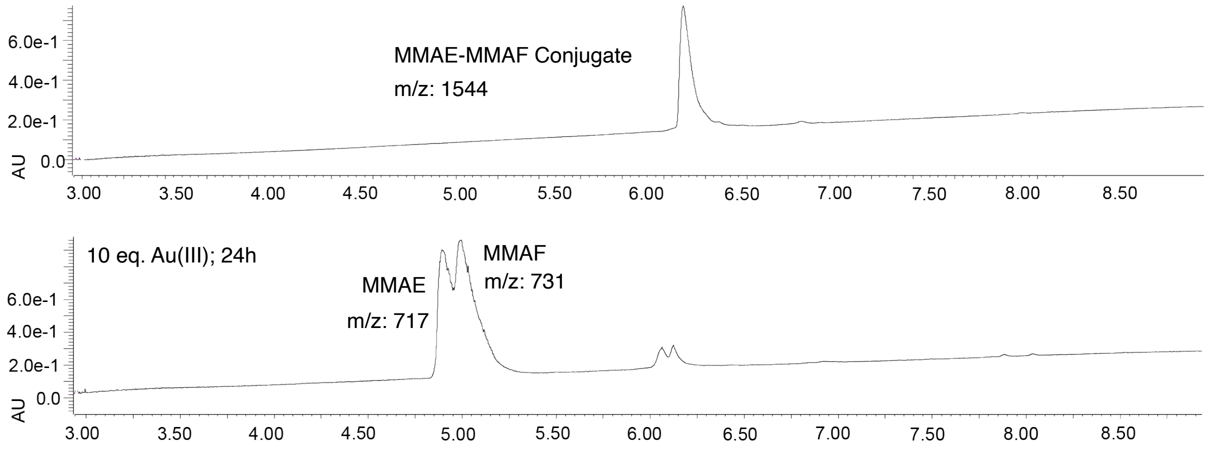

Supporting Figure 33. LC-MS spectrum for the reaction of a MMAE-MMAF conjugate with Na[AuCl$_4$] (10 equivalents). Retention time 6.25 corresponds to the starting material. Retention times 5 and 5.1 to the uncaged products. A possible intermediate was observed at 2 h.

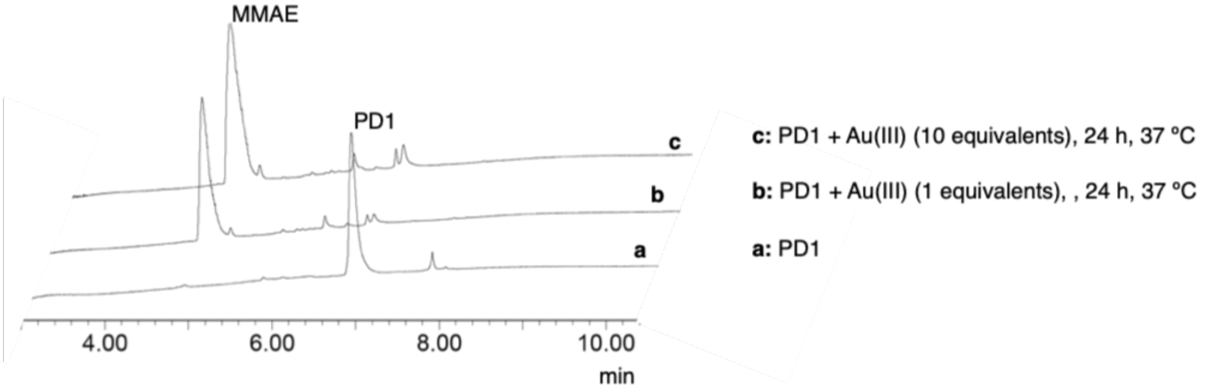

Supporting Figure 34. Reaction of PD1 at varying concentrations of Na[AuCl$_4$]. MMAE is released after 24 h at 37 ºC irrespective of the metal concentration.

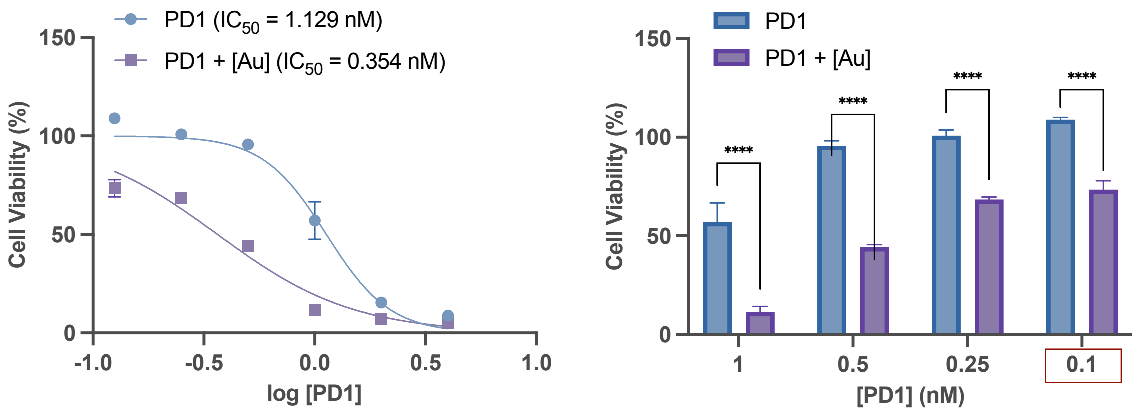

Supporting Figure 35. Toxicity screening of **PD1** and **PD1** + Au(III) in HeLa cells. Toxicity was determined by AlamarBlue assay. Error bars represent ± s.d. (n = 3). The statistical significance of the differences between groups was evaluated with the unpaired t test. Statistical results: ns > 0.05, **P ≤ 0.01, ***P ≤ 0.001 and ****P ≤ 0.0001.

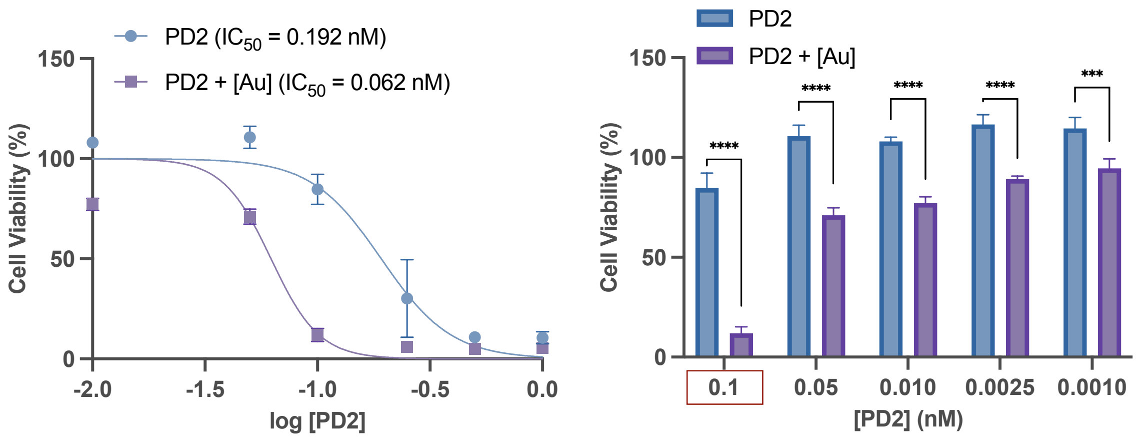

Supporting Figure 36. Toxicity screening of **PD2** and **PD2** + Au(III) in HeLa cells. Toxicity was determined by AlamarBlue assay. Error bars represent ± s.d. (n = 3). The statistical significance of the differences between groups was evaluated with the unpaired t test. Statistical results: ns > 0.05, **P ≤ 0.01, ***P ≤ 0.001 and ****P ≤ 0.0001.

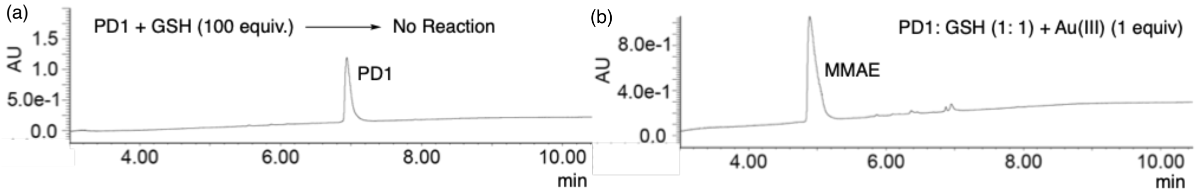

Figure S37. (a) The prodrug's stability was evaluated by incubating it with an excess of glutathione. The stability plot shows that the prodrug remains intact under these conditions, indicating resistance to nucleophilic attack by glutathione. (b) To examine the selectivity of the cleavage reaction, Na[AuCl4] was incubated with a 1:1 ratio of prodrug and glutathione. The results demonstrate that the cleavage reaction proceeds exclusively, with no detectable side reactions involving glutathione.

2.6. Antibody Modification and Uncaging

2.6.1 Antibody Modification:

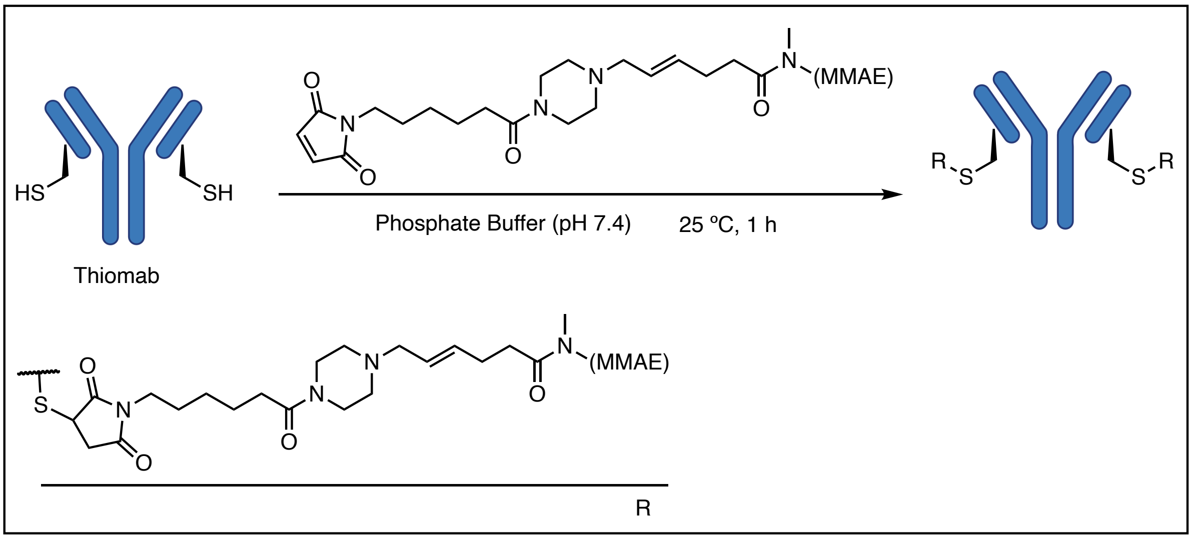

Scheme 8. Synthetic route for the generation of homogenous ADC. Thiomabmab is specific to the HER2, found overexpressed in tumours. Site-selerctive conjugation is expected to occur at the engineered cysteine residues in each light chain of thiomab, enabling the construction of a chemically defined ADC.[18]

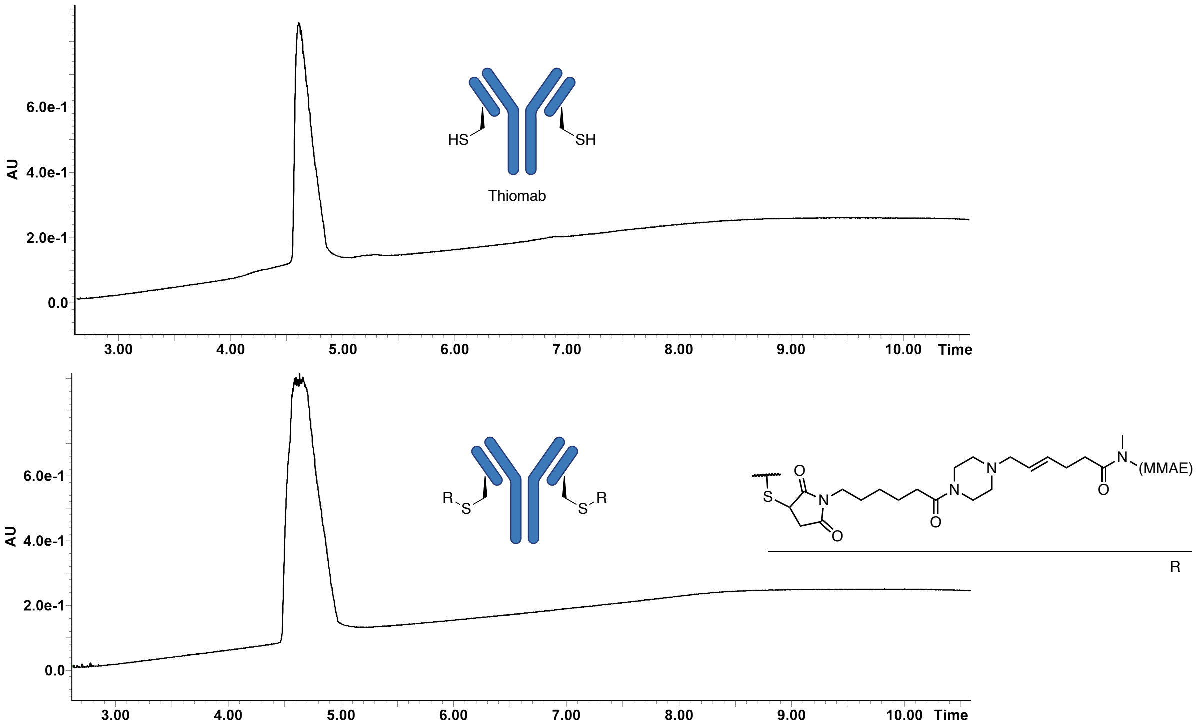

Supporting Figure 38. Chromatograms of thiomab antibody and purified **ADC1** after dialysis with a Slide-A-Lyzer™ MINI Dialysis Device into PBS (3x).

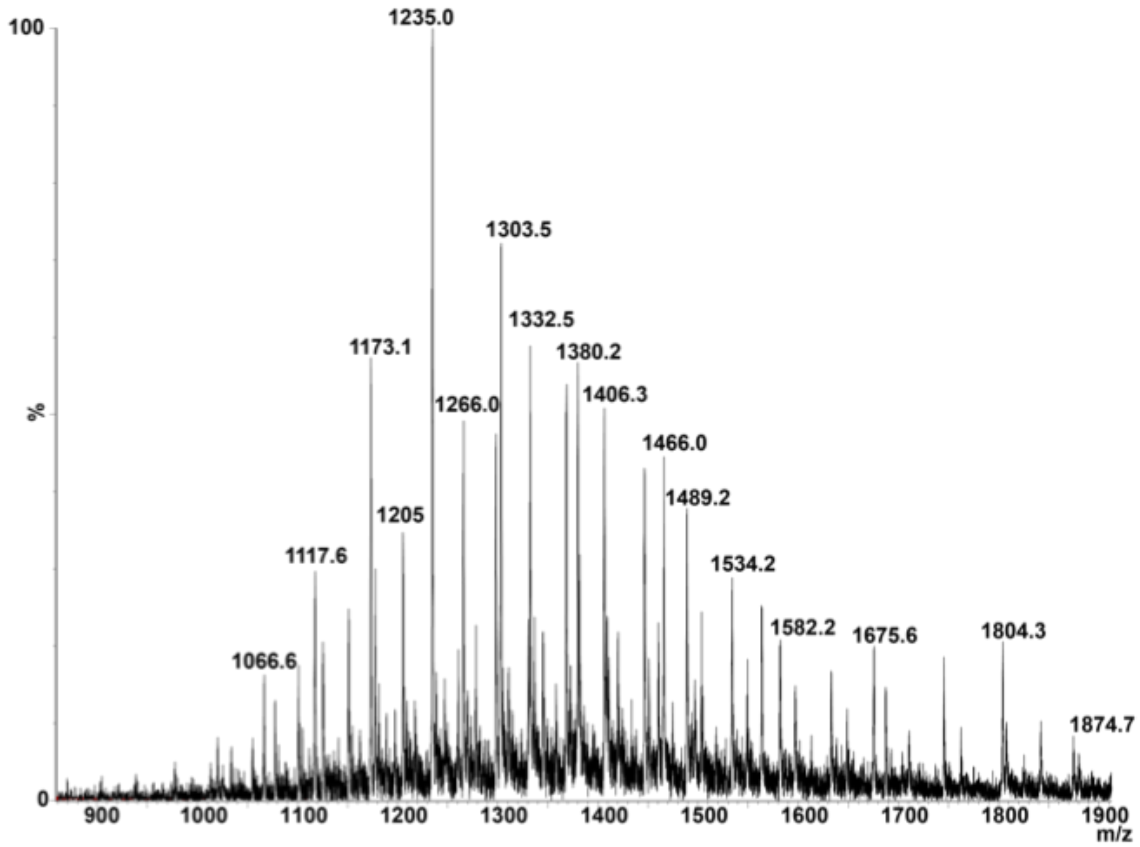

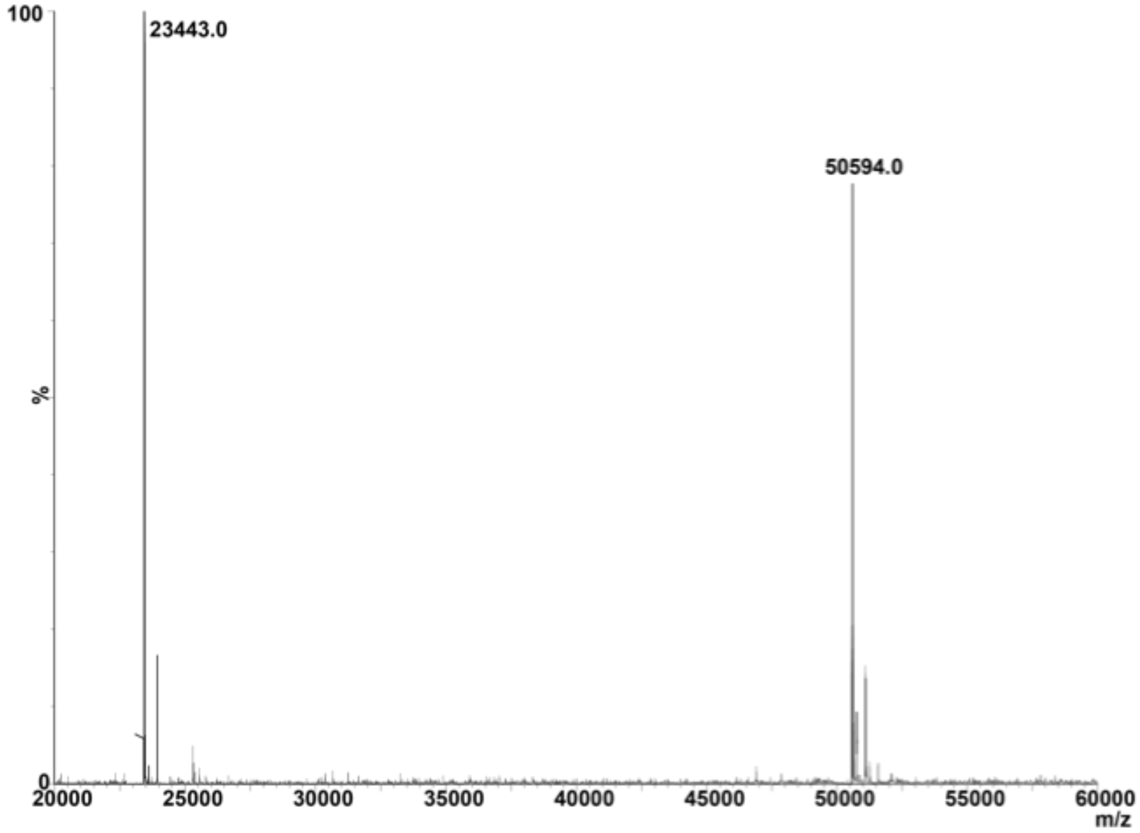

Supporting Figure 39. MS Data for pure thiomab antibody. Thiomabmab is specific to the HER2, found overexpressed in tumours. Site-selective conjugation is expected to occur at the engineered cysteine residues in each light chain of thiomab.

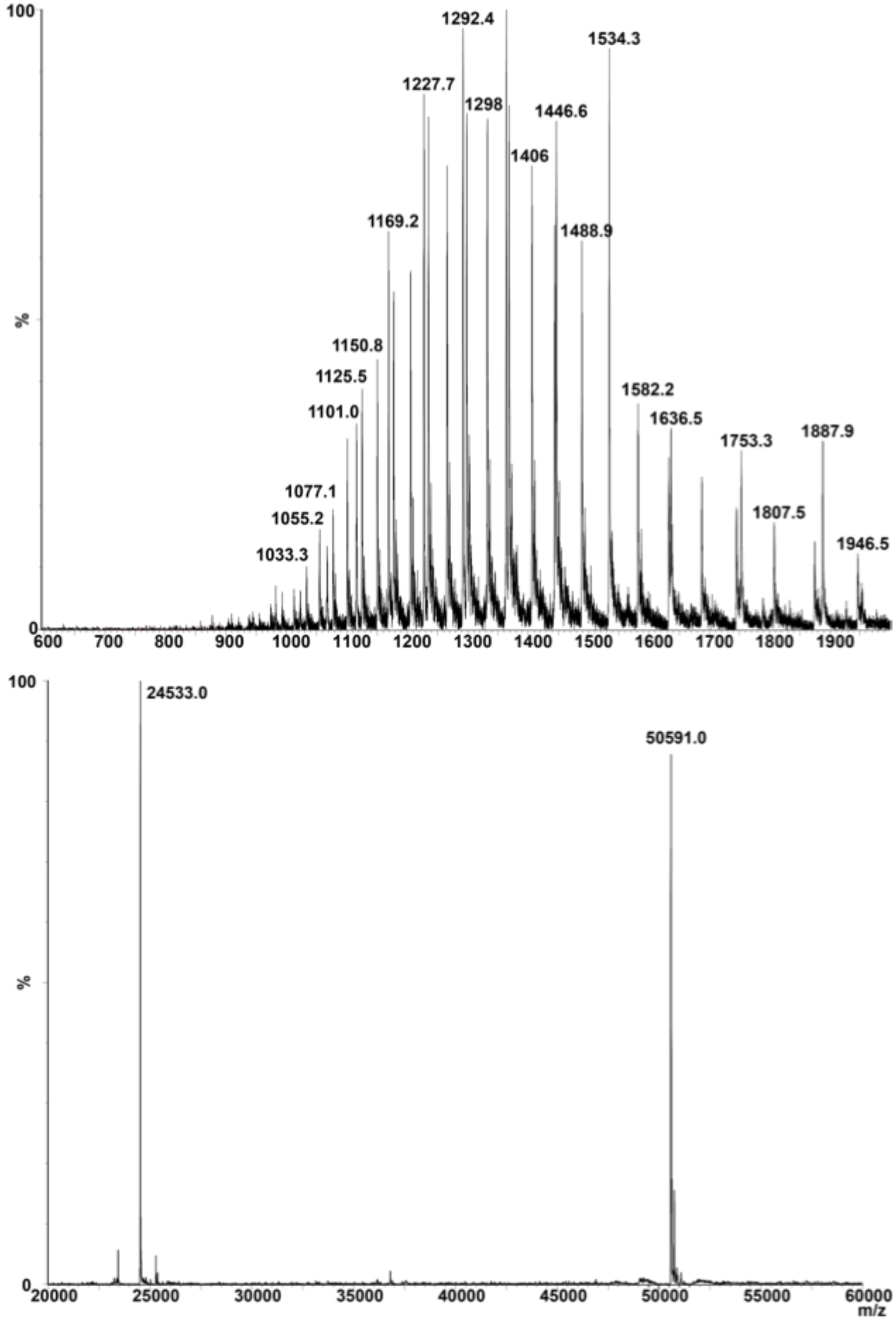

Supporting Figure 40. Complete conversion to a homogeneous ADC was achieved after the reaction of thiomab for **1** h at 37 °C with the maleimide-MMAE drug linker in sodium phosphate buffer at pH 7.4 as assessed by LC−MS.

2.6.2 Antibody Drug Conjugate (ADC) Uncaging:

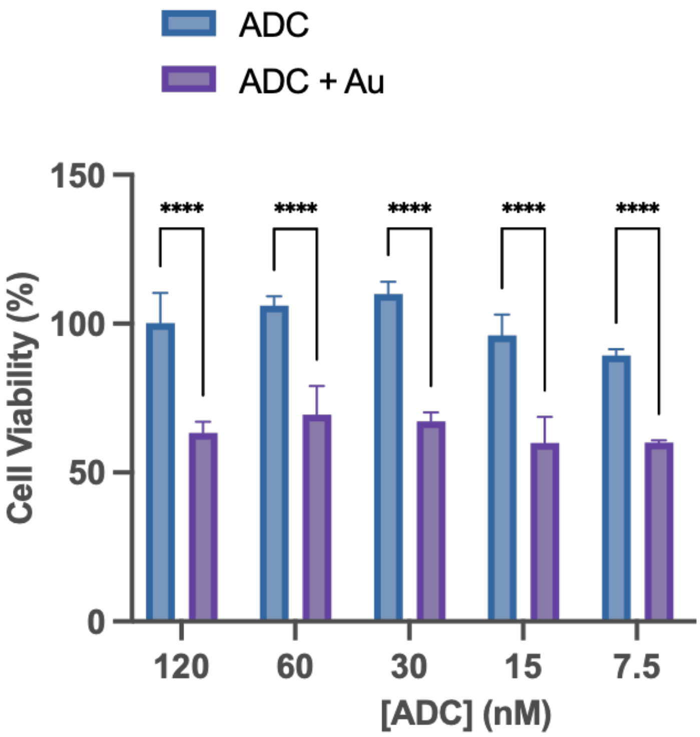

Supporting Figure 41. Toxicity screening of **ADC1** and **ADC1** + Au(III) in HeLa cells. Toxicity was determined by AlamarBlue assay. Error bars represent ± s.d. (n = 3). The statistical significance of the differences between groups was evaluated with the unpaired t test. Statistical results: ns > 0.05, **P ≤ 0.01, ***P ≤ 0.001 and ****P ≤ 0.0001.

2.7. Gold-Mediated Drug Uncaging in a Colon Cancer Zebrafish Xenograft

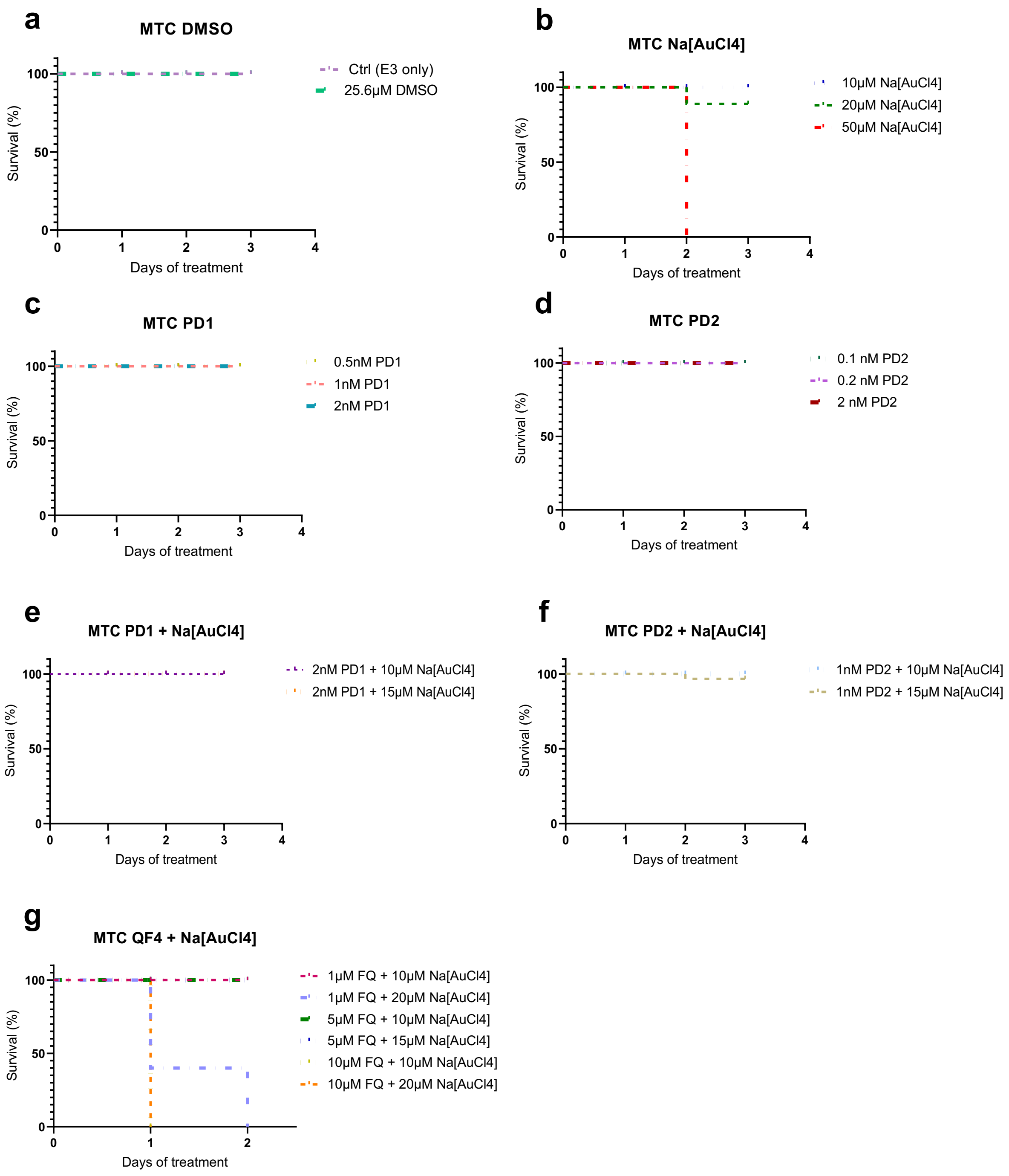

Supporting Figure S42. Maximum Tolerated Dose Concentration assay: Starting at 3 dpf (days post fertilization) different groups of zebrafish larvae were exposed to descending concentrations of (a) DMSO, (b) Na[$AuCl_4$], (c) **PD1**, (d) **PD2**, (e) **PD1**+Na[$AuCl_4$], (f) **PD2**+Na[$AuCl_4$] and (g) **QF4**+Na[$AuCl_4$]. Around 50 zebrafish larvae were used per condition. The concentrations used were determined based on the *in vitro* experiments.

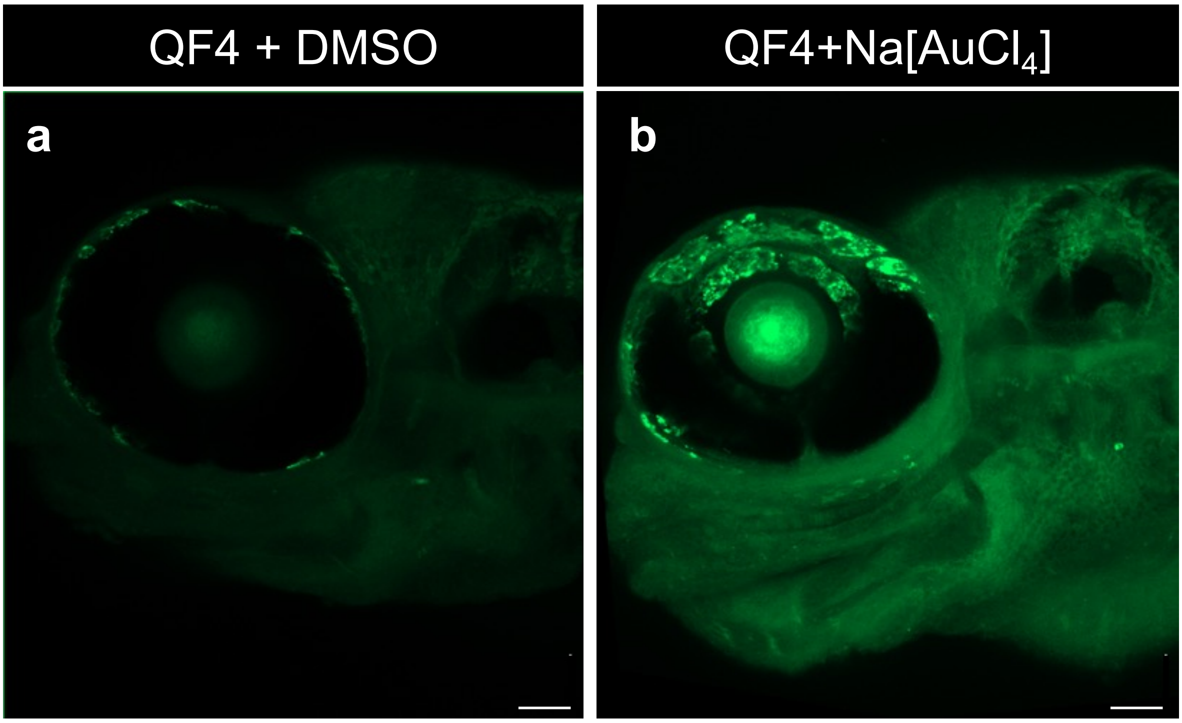

Supporting Figure S43. Na[$AuCl_4$] activates the **QF4** *in vivo***:** Zebrafish larvae were exposed to QF4 (5μM) diluted in embryonic medium for 24 h, followed by a 1 h wash with embryonic medium alone. Larvae were randomly distributed into two conditions: DMSO or Na[$AuCl_4$] (15μM) for 24 h. Representative confocal images of zebrafish larvae exposed to (a) **QF4** + DMSO and (b) **QF4** + Na[$AuCl_4$].

2.8. NMR spectra

Allyl morpholine-4-carboxylate (A)

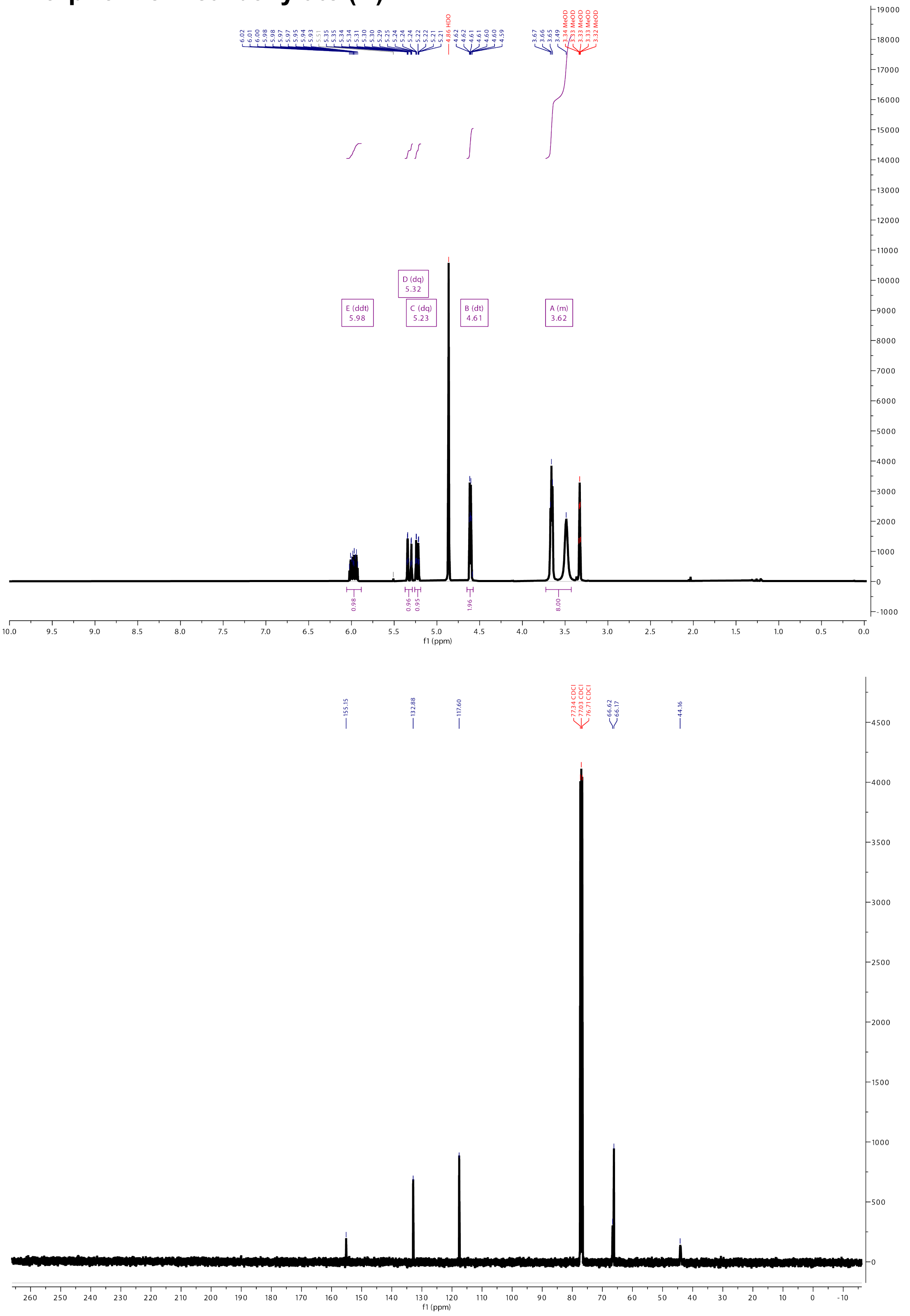

^{1}H-NMR and ^{13}C-NMR of **A**.

1-Morpholinopent-4-en-1-one (B)

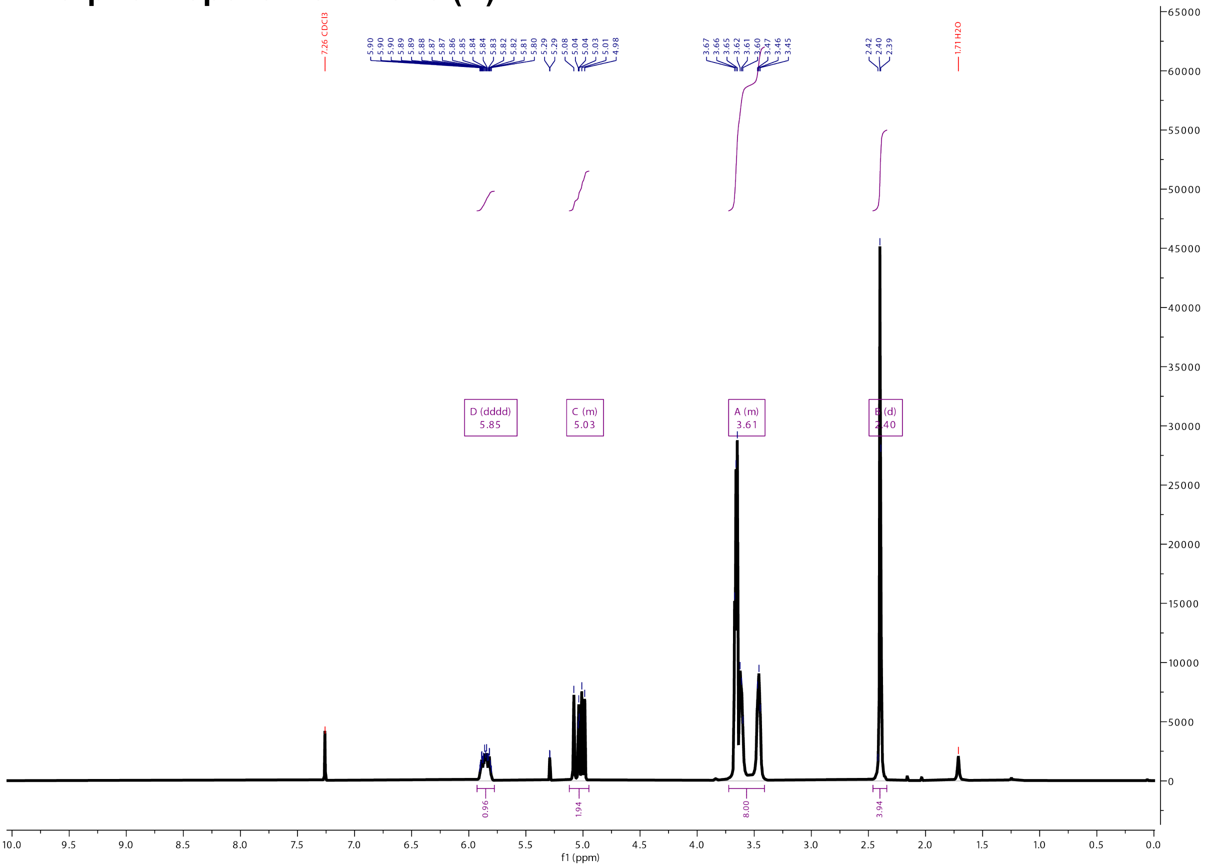

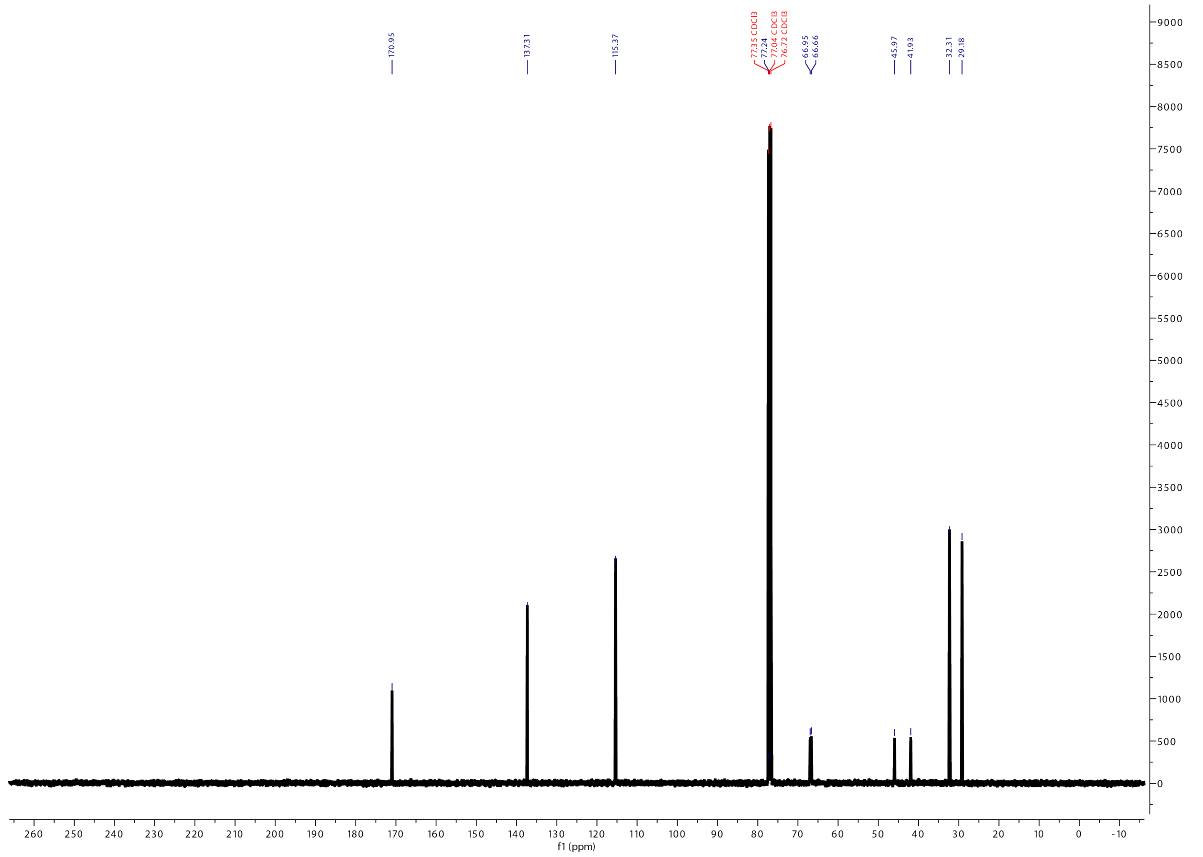

^{1}H-NMR and ^{13}C-NMR of **B**.

1-Morpholinopentan-1-one (C)

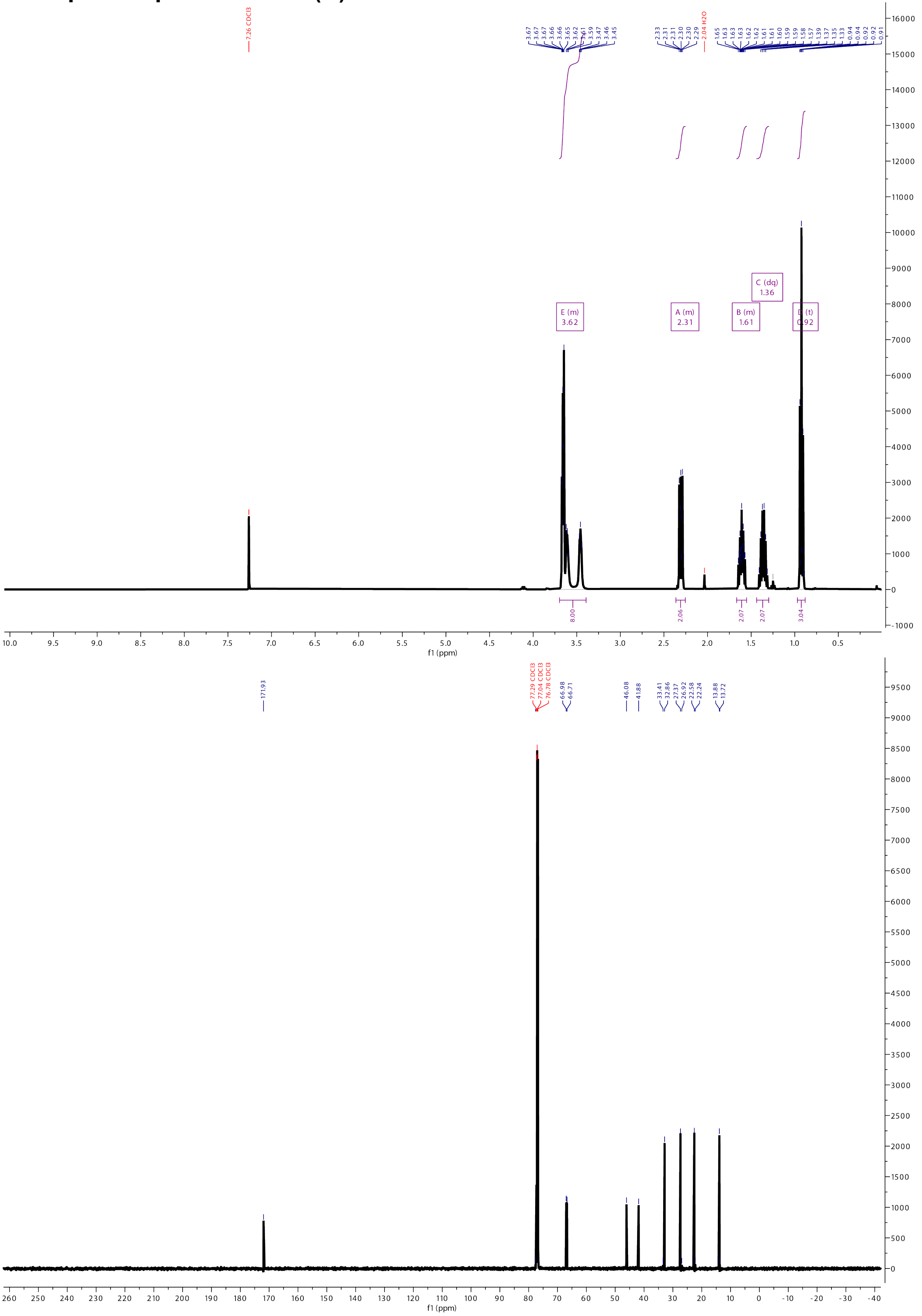

^{1}H-NMR and ^{13}C-NMR of **C**.

Morpholino(2-vinylphenyl)methanone (D)

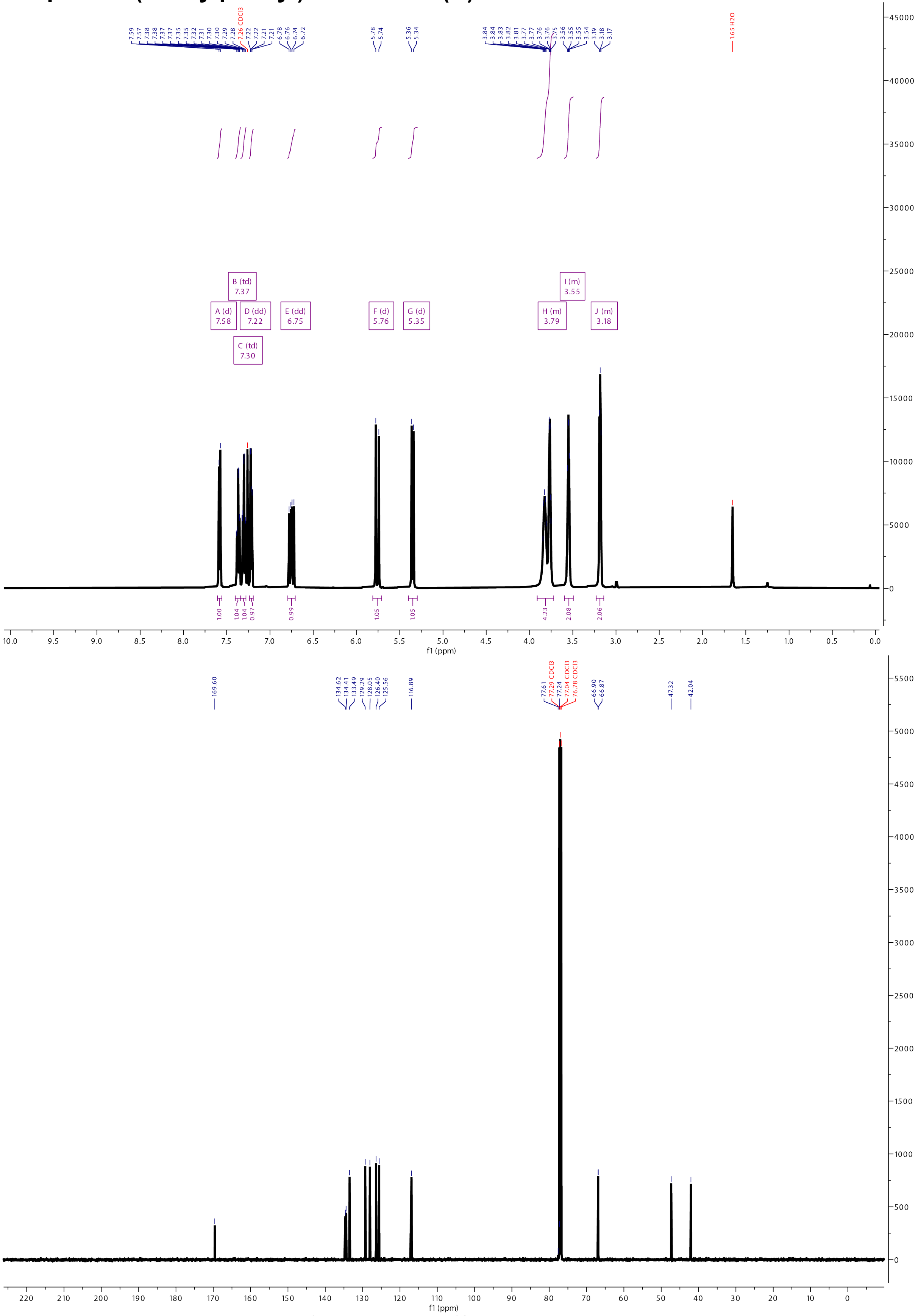

^{1}H-NMR and ^{13}C-NMR of **D**.

N-(2-methoxyethyl)pent-4-enamide (E)

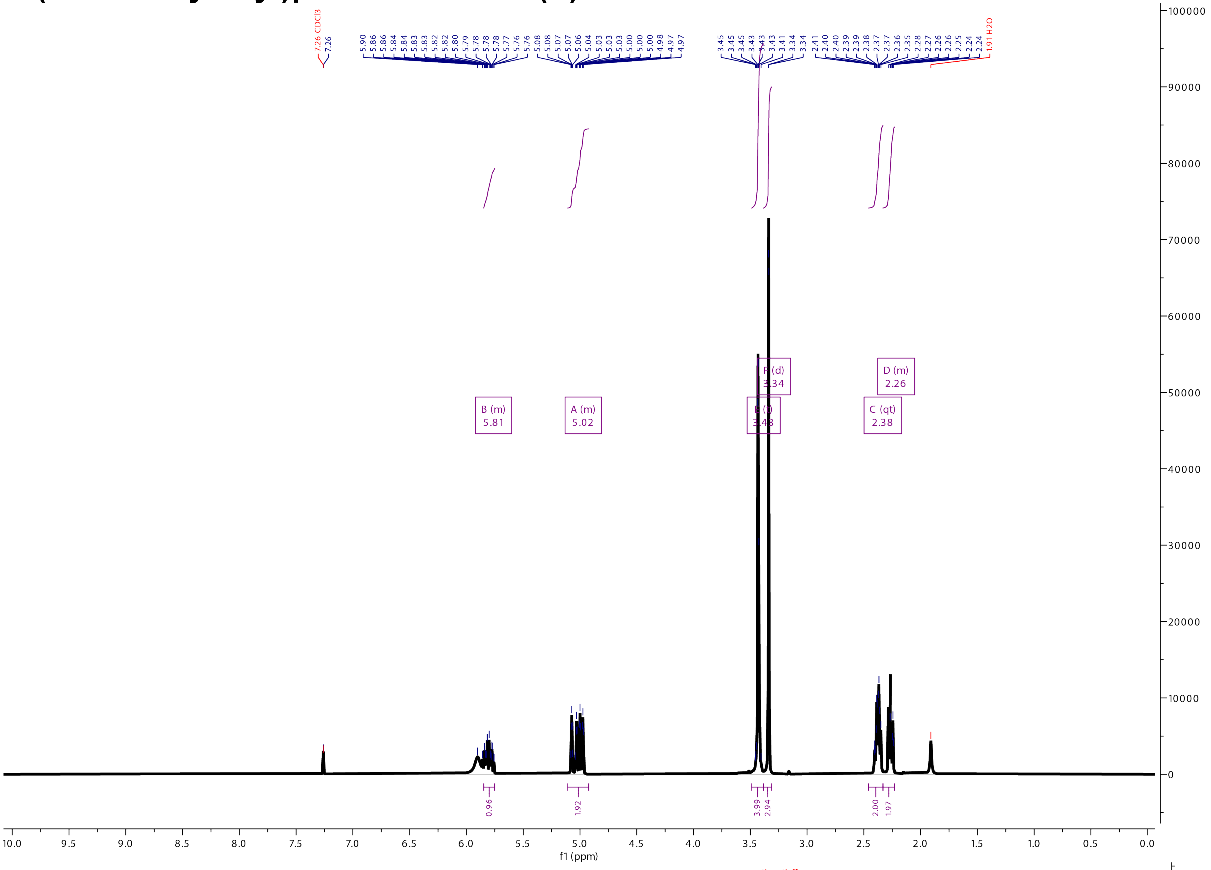

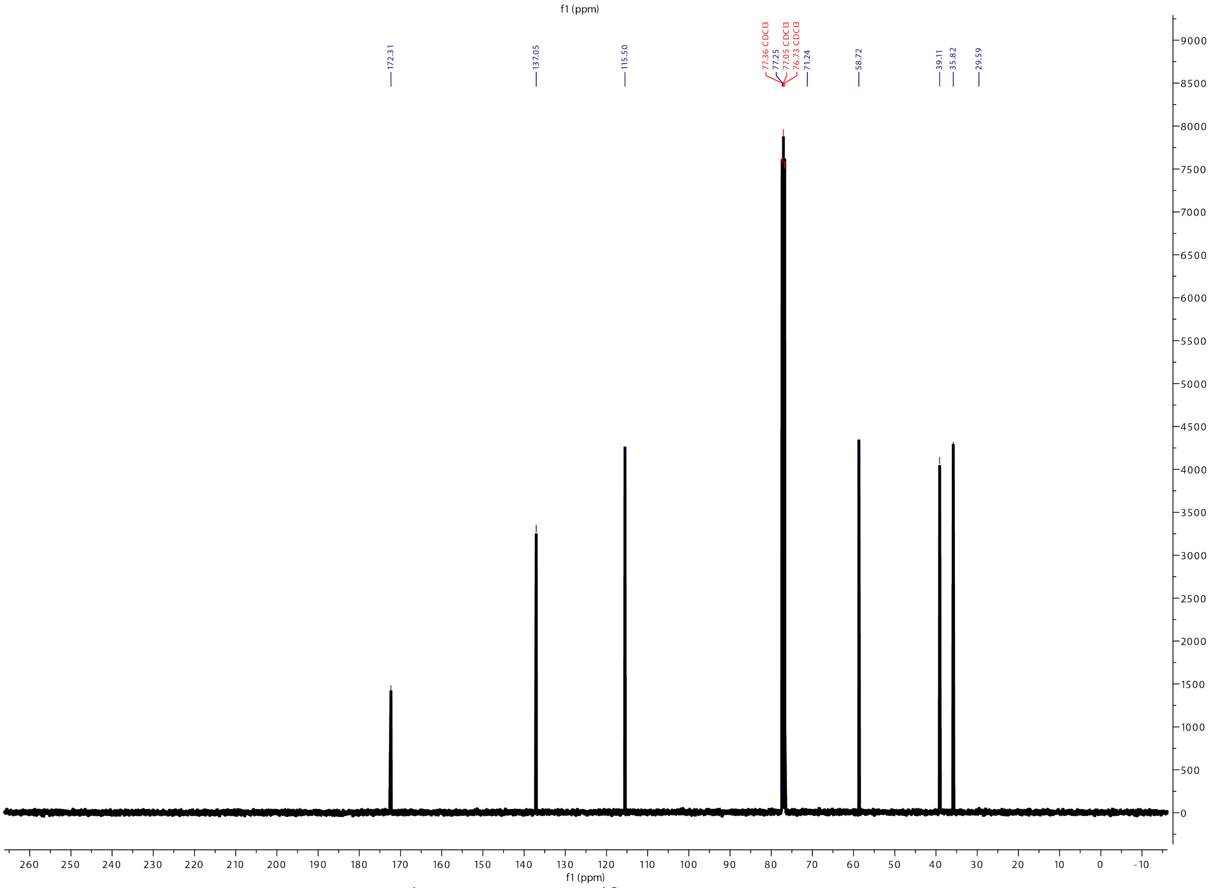

^{1}H-NMR and ^{13}C-NMR of **E**.

Methyl pent-4-enoylglycinate (F)

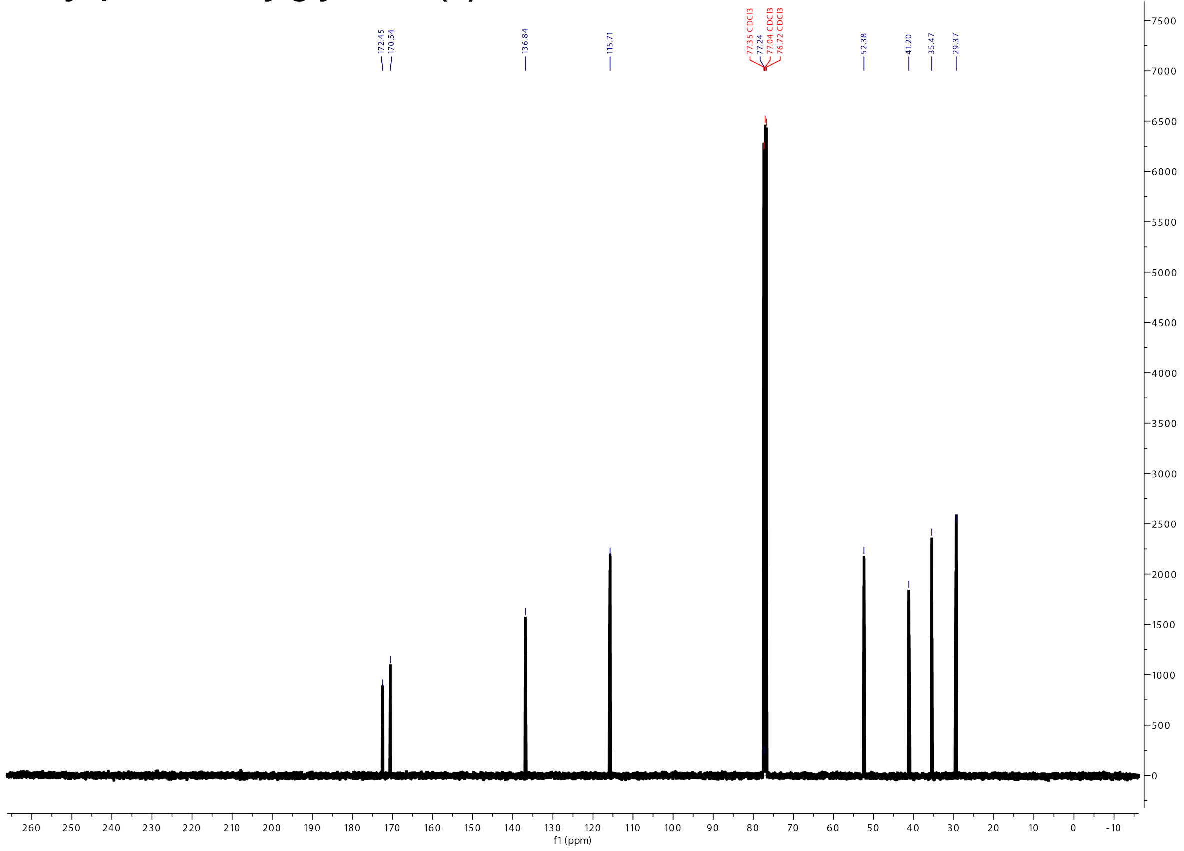

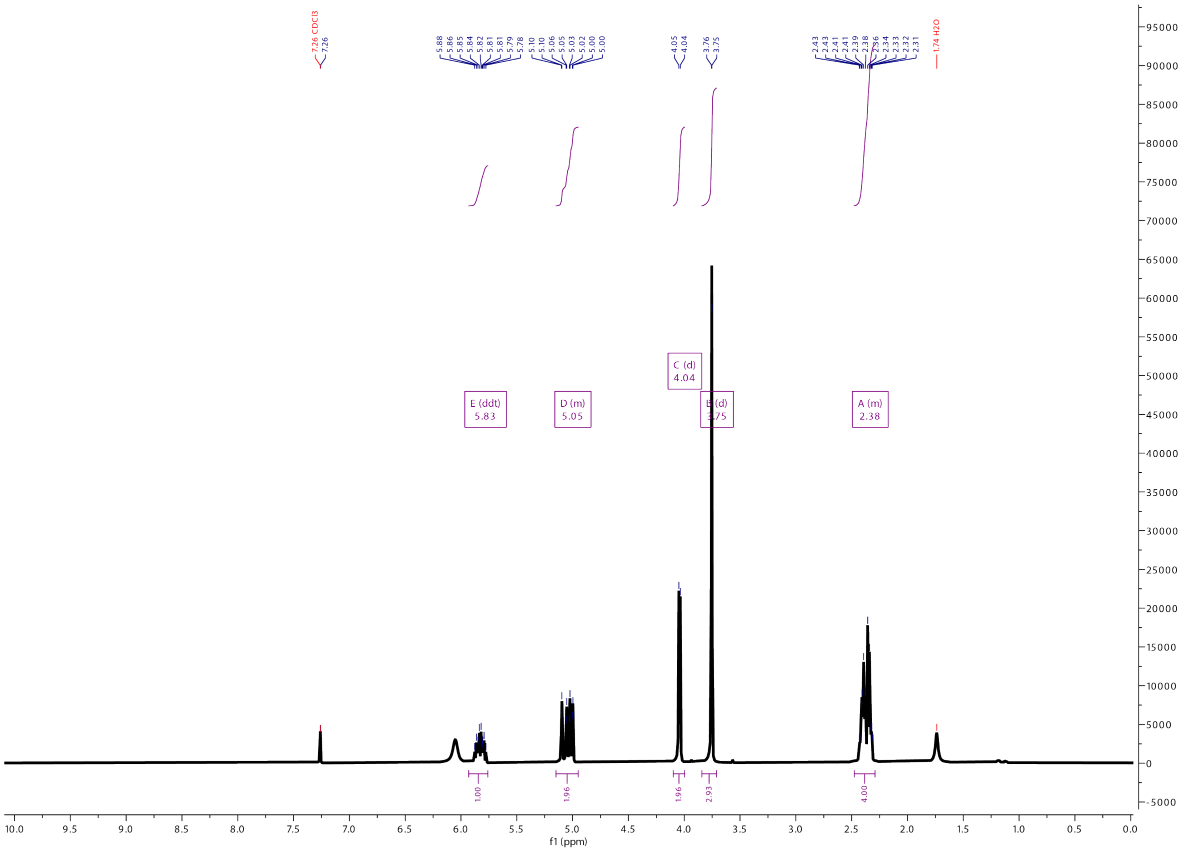

^{1}H-NMR and ^{13}C-NMR of **F**.

1-Morpholino-6-phenoxyhex-4-en-1-one (H)

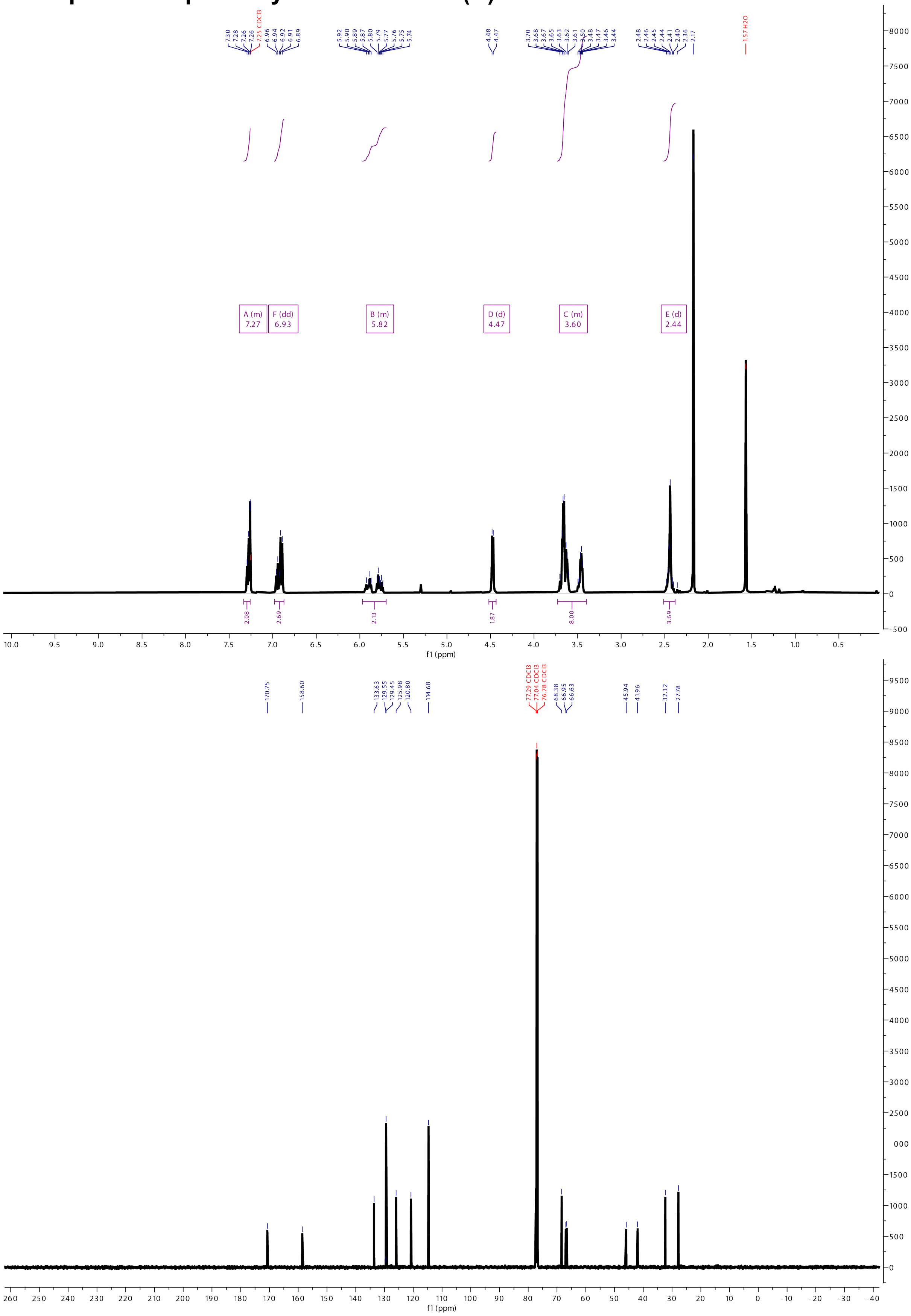

^{1}H-NMR and ^{13}C-NMR of **H**.

1-Morpholino-6-(perfluorophenoxy)hex-4-en-1-one (I)

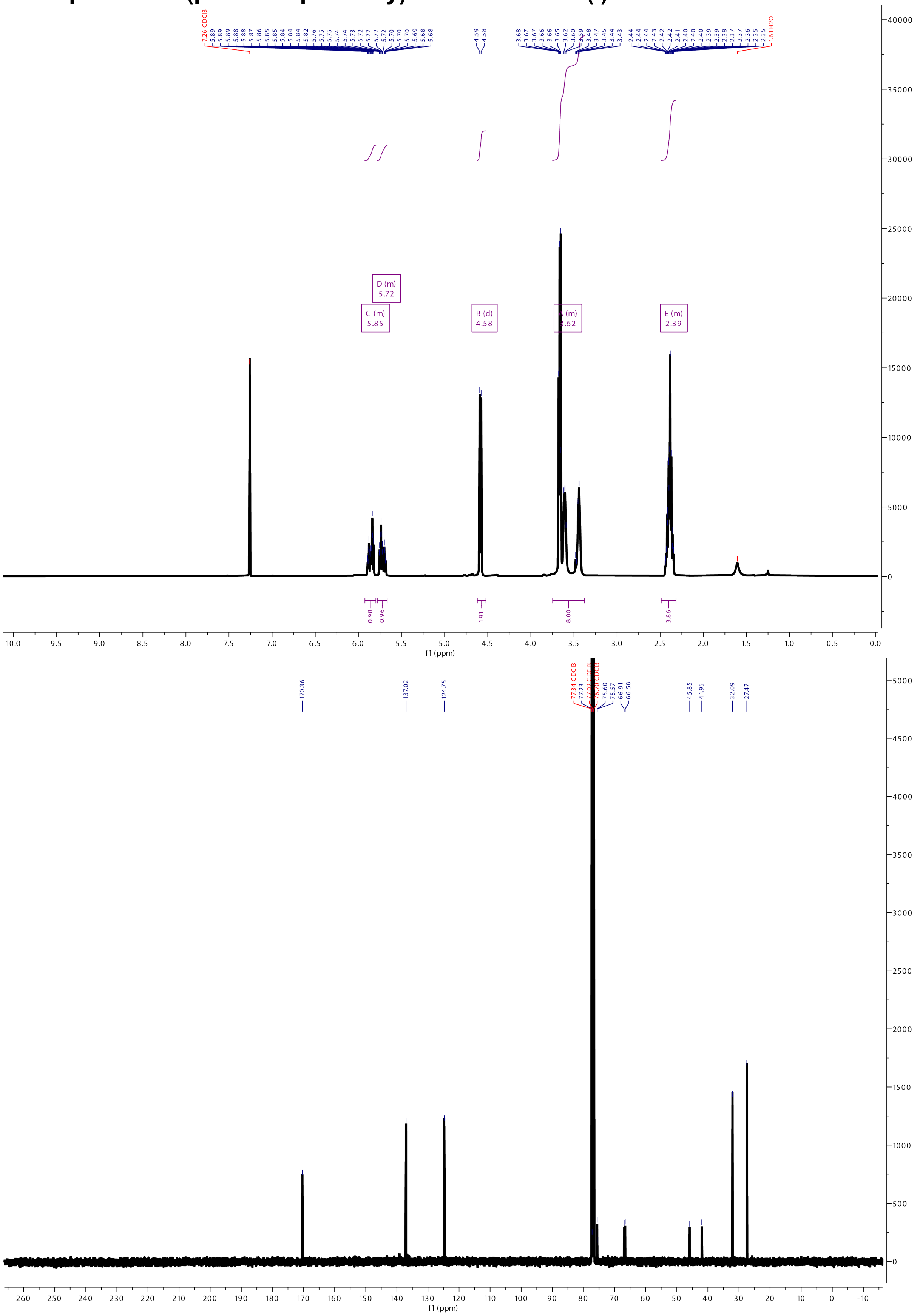

^{1}H-NMR and ^{13}C-NMR of **I**.

3-(methylthio)-1-morpholinopropan-1-one (J)

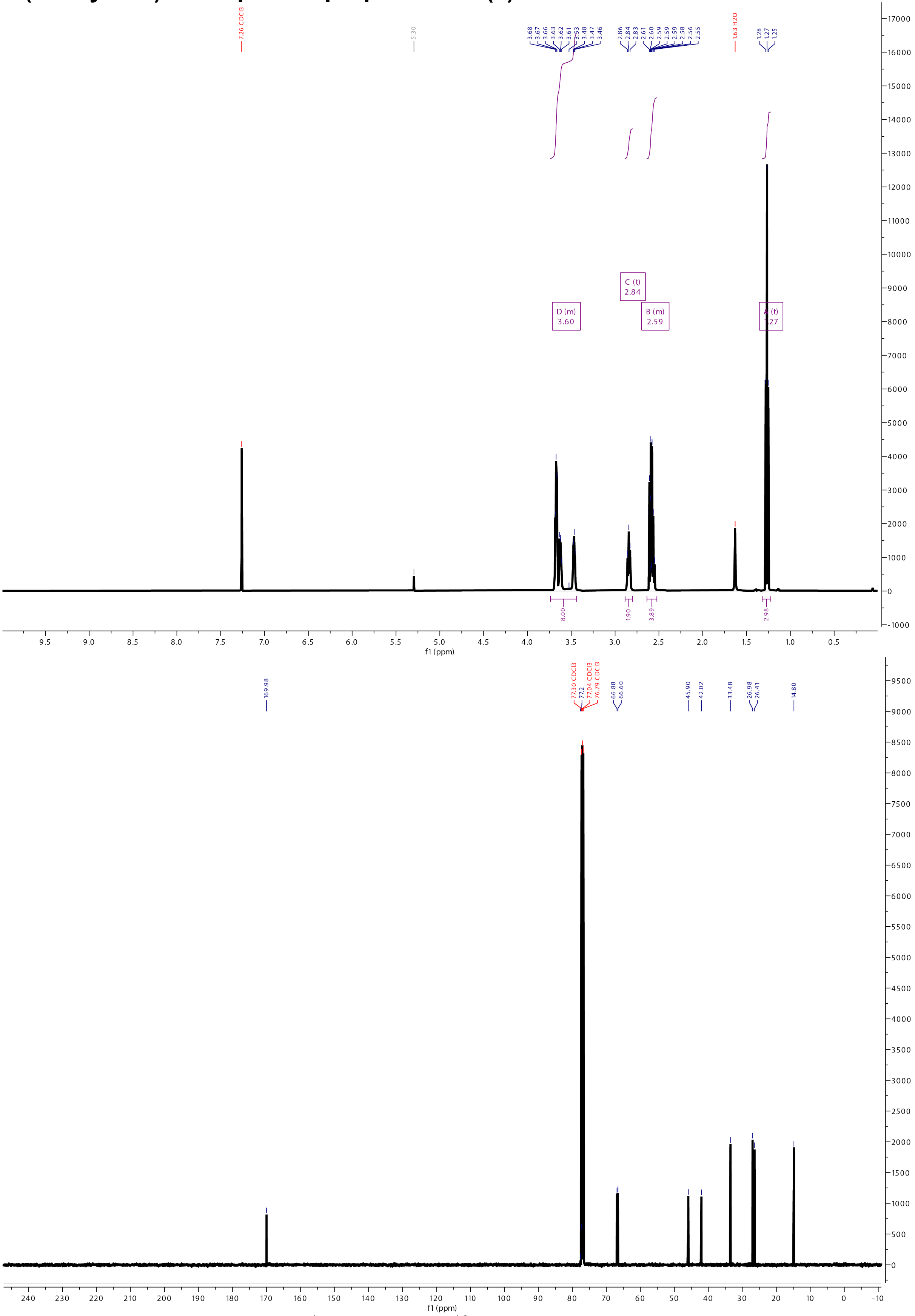

^{1}H-NMR and ^{13}C-NMR of **J**.

***N*-methyl-*N*-phenylpent-4-enamide (K)**

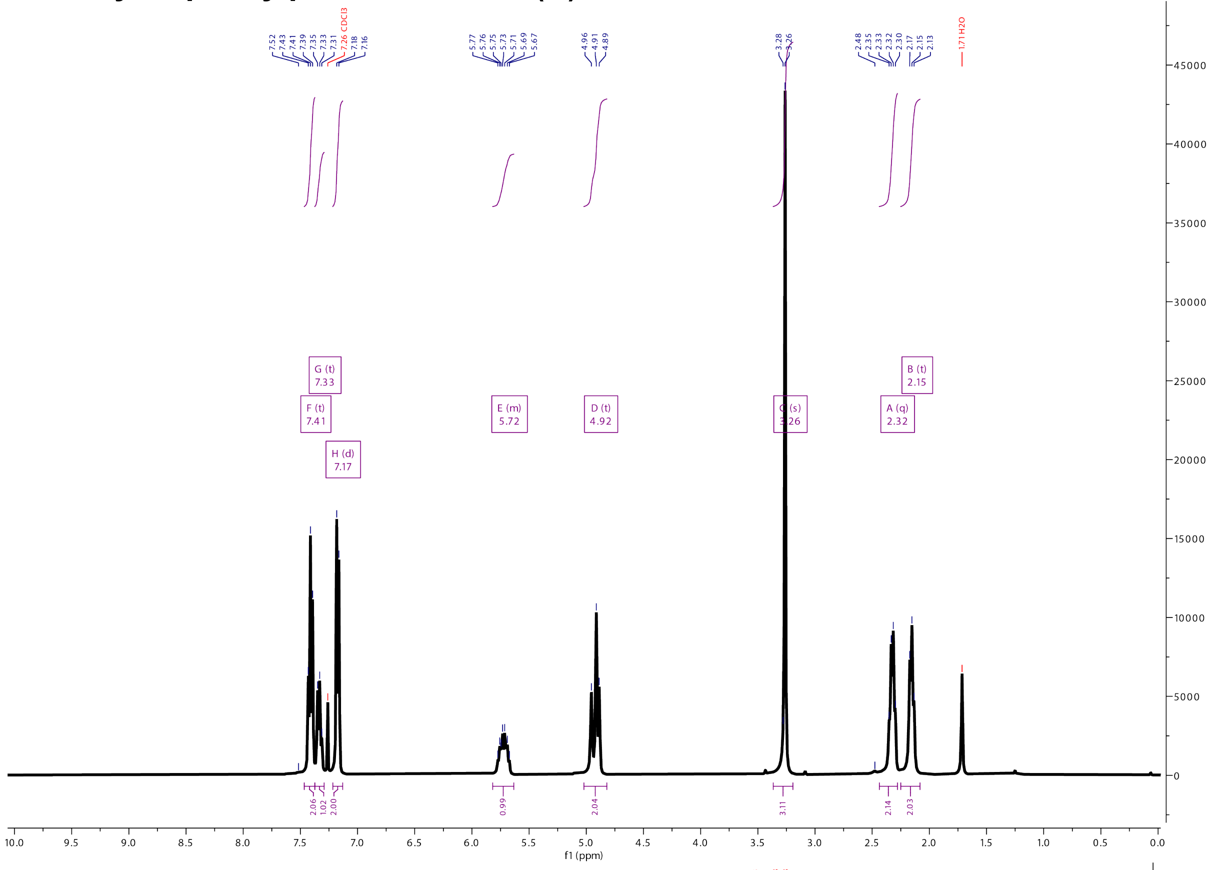

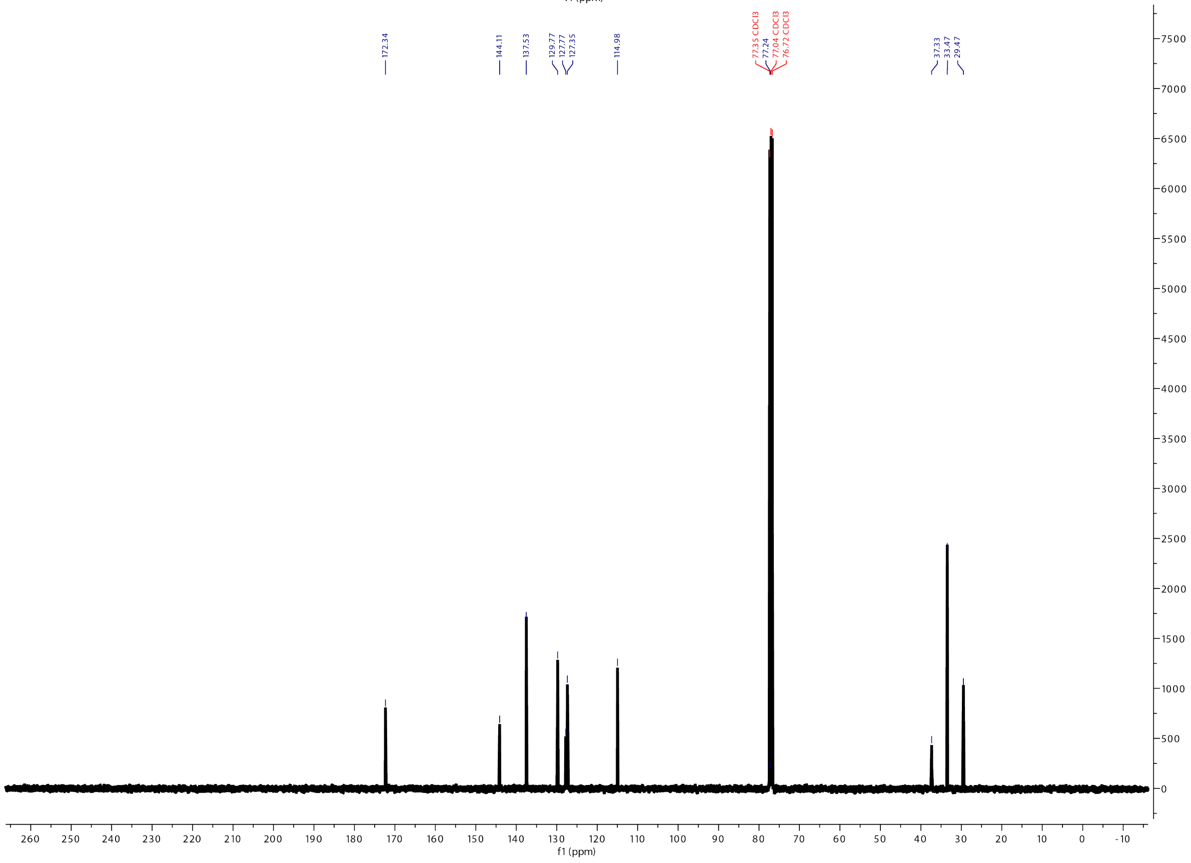

^{1}H-NMR and ^{13}C-NMR of **K**.

***N*-benzyl-*N*-methylpent-4-enamide (L)**

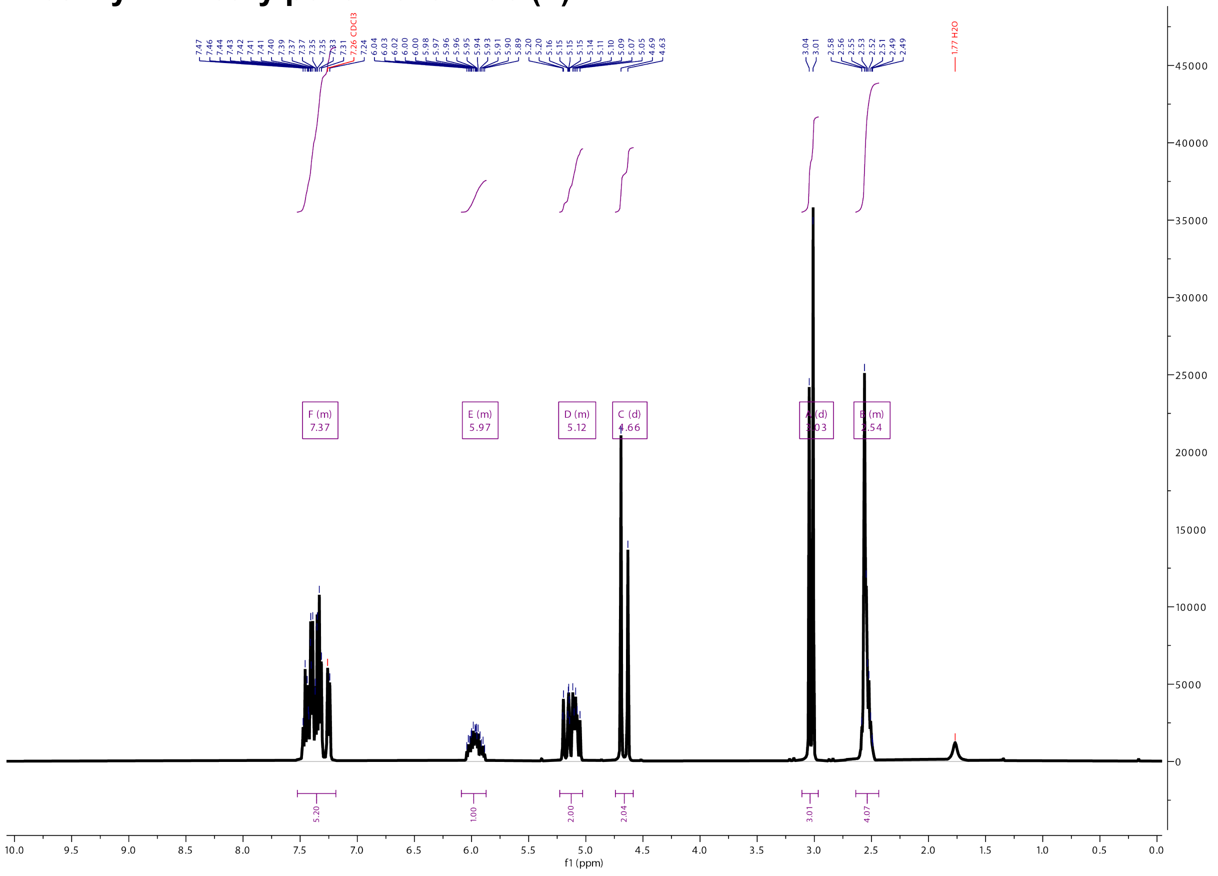

^{1}H-NMR of **L**.

Allyl 4-(2-butyl-1,3-dioxo-2,3-dihydro-1*H*-benzo[*de*]isoquinolin-6-yl)piperazine-1-carboxylate (QF1)

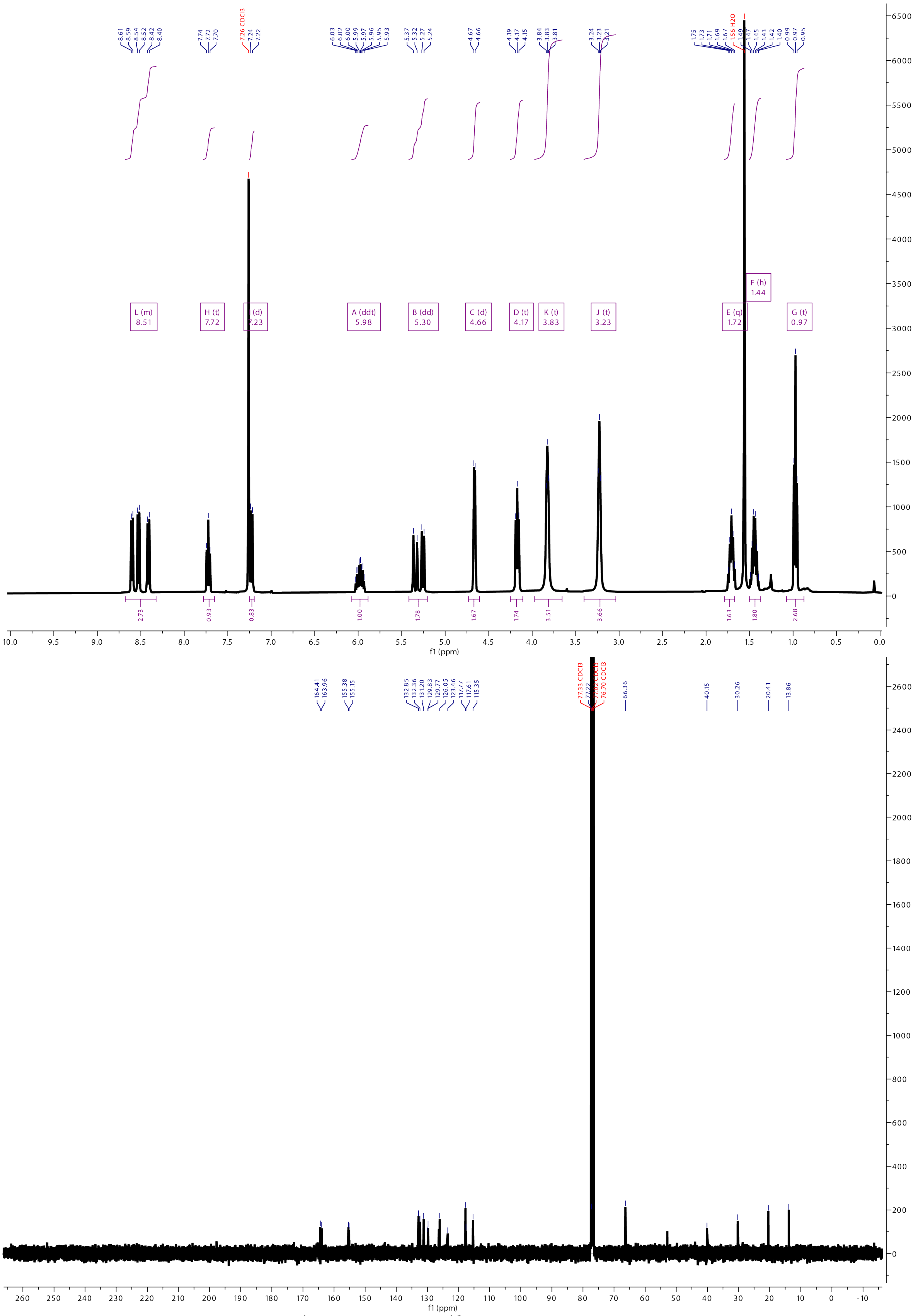

^{1}H-NMR and ^{13}C-NMR of **QF1**.

2-butyl-6-(4-(pent-4-enoyl)piperazin-1-yl)-1*H*-benzo[*de*]isoquinoline-1,3(2*H*)-dione (QF2)

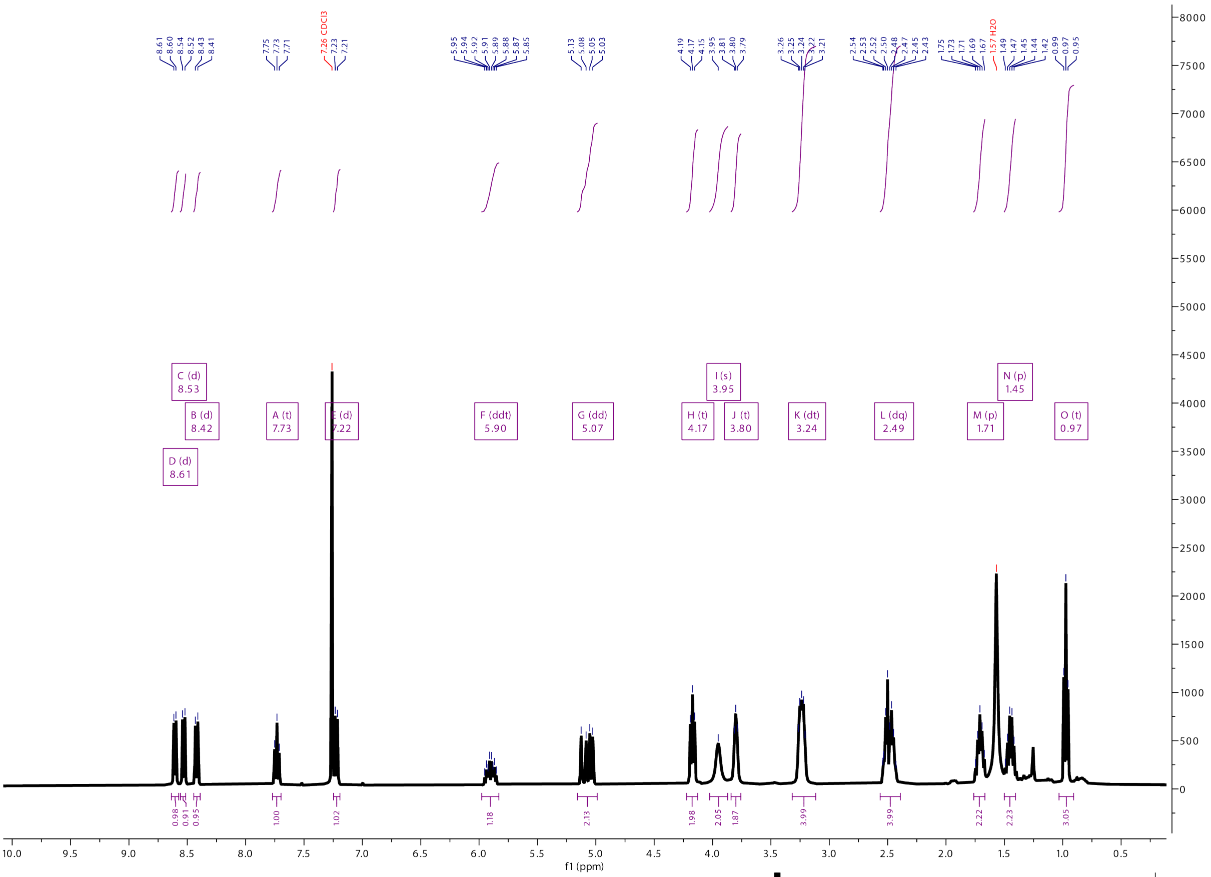

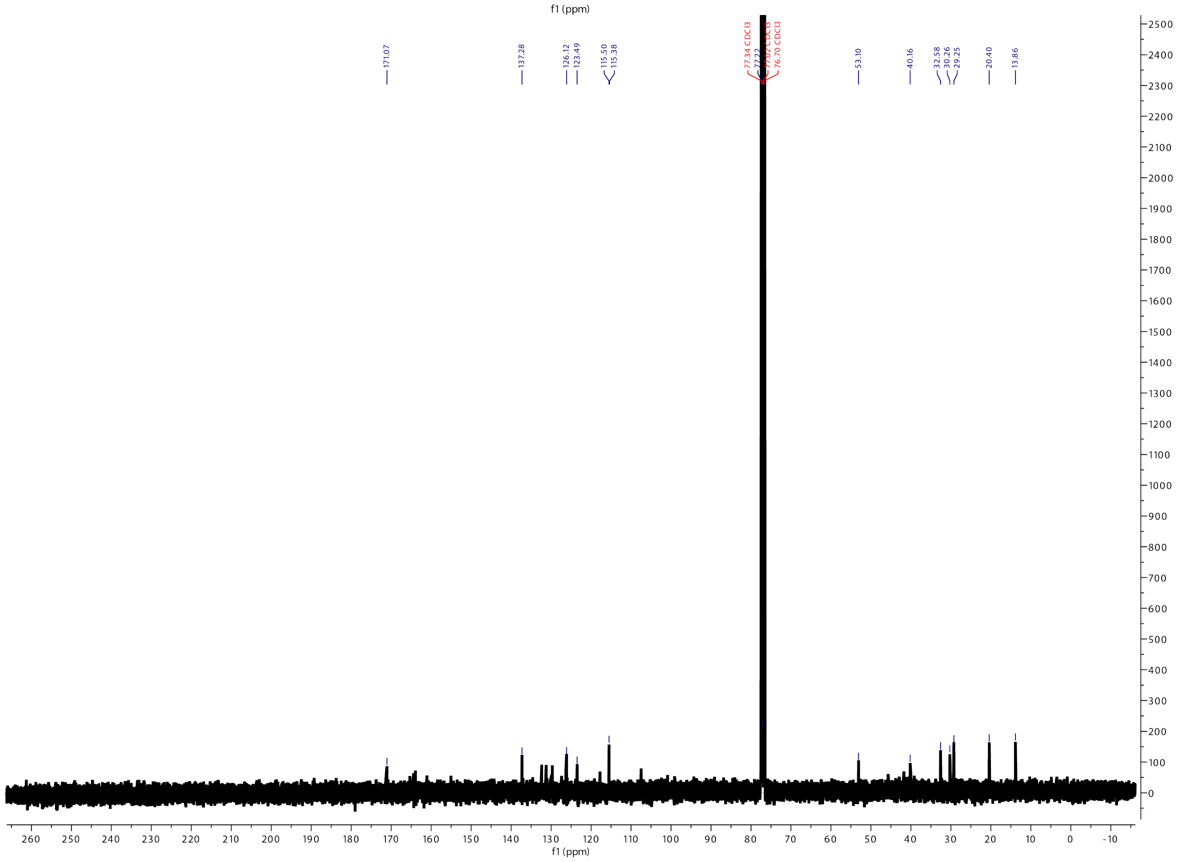

^{1}H-NMR and ^{13}C-NMR of **QF2**.

2-butyl-6-(4-(6-morpholino-6-oxohex-2-en-1-yl)piperazin-1-yl)-1*H* benzo[*de*]isoquinoline-1,3(2*H*)-dione (QF3)

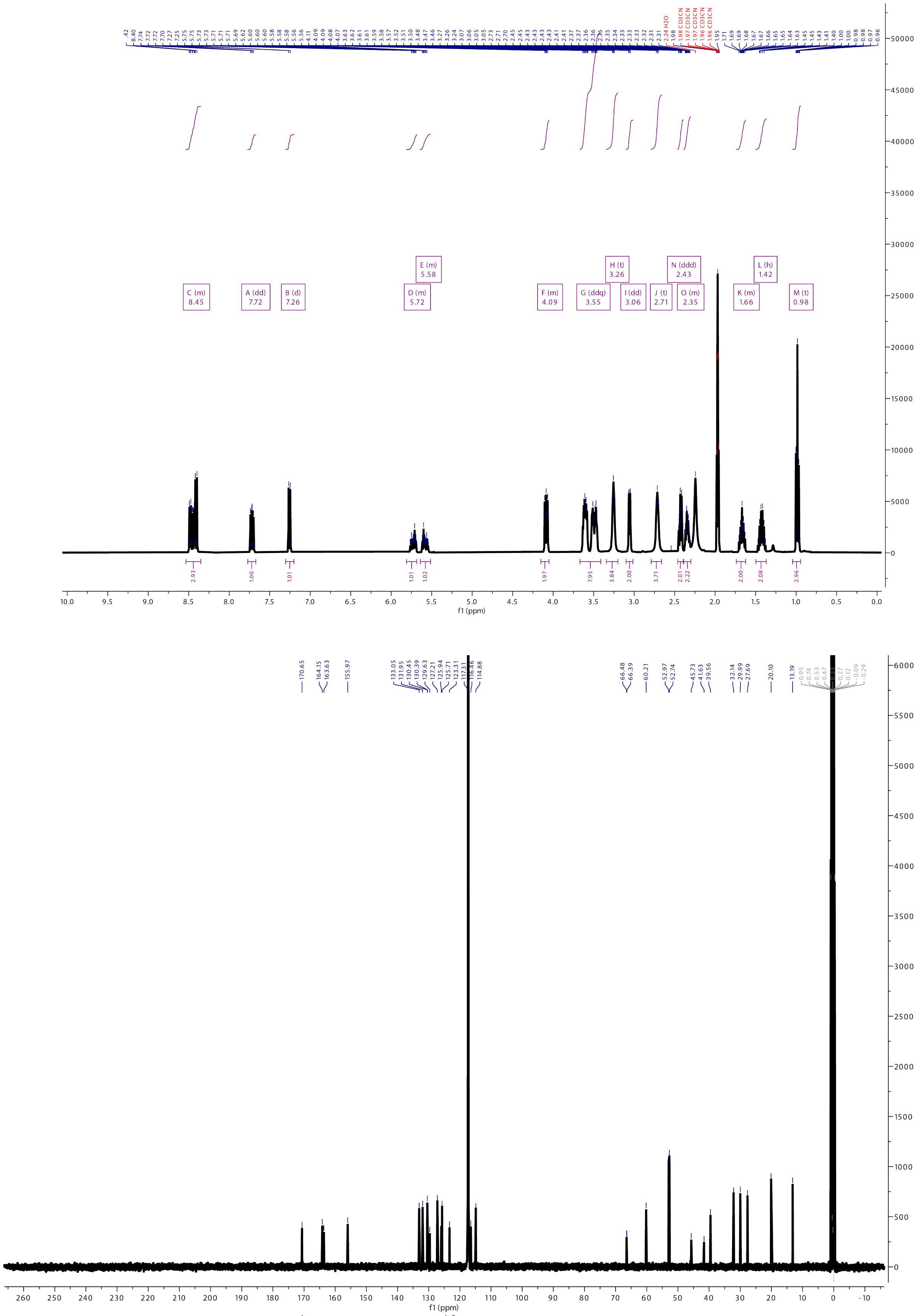

^{1}H-NMR and ^{13}C-NMR of **QF3**.

2.9 Quantum Mechanical Calculations (Additional Data)

Supporting Table 3. Energies, entropies, and lowest frequencies of the lowest energy calculated structures.[a]

Structure	E_{elec} (Hartree)	E_{elec} + ZPE (Hartree)	H (Hartree)	S (cal mol^{-1} K^{-1})	G (Hartree)	Lowest freq. (cm^{-1})	# of imag freq.
Na[$AuCl_4$]	–2138.989325	–2138.984344	–2138.973295	107.3	–2139.022655	27.1	0
NaCl	–622.581389	–622.580846	–622.577039	55.8	–622.603571	238.4	0
Na	–162.190498	–162.190498	–162.188138	35.3	–162.204927		0
$(H_2O)_3$	–229.289294	–229.216353	–229.207988	79.2	–229.245641	179.9	0
$[H_3O{\cdot}H_2O]^+$	–153.290485	–153.231983	–153.226363	65.6	–153.256709	41.8	0
$NHMe_2$	–135.097542	–135.005858	–135.000554	64.6	–135.031265	229.6	0
NHMePh	–326.739971	–326.595989	–326.587809	83.0	–326.627225	104.7	0
**B'**	–404.356795	–404.166735	–404.154577	105.1	–404.203101	48.1	0
**B'**·$AuCl_3$ *(carbonyl)*	–1920.756576	–1920.560729	–1920.541823	141.1	–1920.606447	32.0	0
**B'**·$AuCl_3$ *(alkene)*	–1920.771041	–1920.574393	–1920.555967	137.4	–1920.619449	44.7	0
**B'**·$AuCl_3$*5ext (TS)*	–1920.766388	–1920.571087	–1920.552842	140.7	–1920.616210	–73.2	1
**B'**·$AuCl_3$*5ext*	–1920.805056	–1920.605400	–1920.587821	135.8	–1920.649632	20.4	0
**B'**·$AuCl_3$*5ext*_hy *(preTS)*	–2150.111227	–2149.837993	–2149.811347	175.8	–2149.890732	21.4	0
**B'**·$AuCl_3$*5ext*_hy *(TS)*	–2150.084146	–2149.811558	–2149.787972	160.3	–2149.861145	–508.5	1
**B'**·$AuCl_3$*5ext*_hy *(postTS)*	–2150.088669	–2149.814212	–2149.790682	161.9	–2149.863789	27.8	0
**B'**·$AuCl_3$*5ext*_hy_{hem}	–1996.774689	–1996.561276	–1996.542468	142.4	–1996.606543	15.5	0
**B'**·$AuCl_3$*5ext*_hy_{zw}	–1996.765742	–1996.551683	–1996.532952	139.6	–1996.596943	34.6	0
**B'**·$AuCl_3$*5ext*_hy_{zw} *(TS)*	–1996.764861	–1996.551698	–1996.533373	137.2	–1996.596658	–179.8	1
**B'**·$AuCl_3$*5ext*_hy_{zw} *(postTS)*	–1996.783032	–1996.571582	–1996.551441	147.8	–1996.619048	40.0	0
**B'**·$AuCl_3$*5ext*_lac	–1861.675683	–1861.556678	–1861.542655	119.4	–1861.597550	26.8	0
**B'**·$AuCl_3$*5ext*_acid	–1861.616350	–1861.500875	–1861.485767	123.0	–1861.542943	58.0	0
**B'**·$AuCl_3$*6ent (TS)*	–1920.756733	–1920.561084	–1920.542598	142.1	–1920.606889	–224.4	1
**B'**·$AuCl_3$*6ent*	–1920.791894	–1920.592086	–1920.573888	141.0	–1920.637497	25.3	0
**B'**·$AuCl_3$*6ent*_hy_{zw}	–1996.759787	–1996.544876	–1996.526030	143.4	–1996.590184	11.7	0
**B'**·$AuCl_3$*6ent*_hy_{zw} *(TS)*	–1996.755078	–1996.542640	–1996.523556	145.0	–1996.588441	–135.8	1
**B'**·$AuCl_3$*6ent*_hy_{zw} *(postTS)*	–1996.772661	–1996.560417	–1996.540304	149.0	–1996.607558	20.3	0
**B'**·$AuCl_3$*6ent*_lac	–1861.663857	–1861.544673	–1861.530194	122.5	–1861.586265	34.4	0
**K**	–595.988082	–595.746193	–595.730844	125.8	–595.786248	21.2	0
**K**·$AuCl_3$ *(alkene)*	–2112.402369	–2112.153768	–2112.132178	154.8	–2112.201918	31.1	0
**K**·$AuCl_3$*5ext (TS)*	–2112.397319	–2112.149752	–2112.128529	159.0	–2112.197587	–93.3	1
**K**·$AuCl_3$*5ext*	–2112.435150	–2112.183572	–2112.162924	154.0	–2112.230650	7.9	0
**K**·$AuCl_3$*5ext*_hy *(preTS)*	–2341.740401	–2341.414704	–2341.385185	189.3	–2341.469948	28.1	0
**K**·$AuCl_3$*5ext*_hy *(TS)*	–2341.714328	–2341.389213	–2341.362644	174.7	–2341.441419	–443.1	1
**K**·$AuCl_3$*5ext*_hy *(postTS)*	–2341.719882	–2341.393037	–2341.366513	177.3	–2341.445232	22.3	0
**K**·$AuCl_3$*5ext*_hy_{hem}	–2188.406974	–2188.140281	–2188.118794	153.9	–2188.188111	24.2	0
**K**·$AuCl_3$*5ext*_hy_{zw} *(postTS)*	–2188.425985	–2188.162284	–2188.138963	163.6	–2188.212565	32.5	0
**H'**	–749.776241	–749.472360	–749.453629	142.0	–749.516193	17.9	0
**H'**·$AuCl_3$ *(alkene)*	–2266.188848	–2265.879384	–2265.853709	176.5	–2265.931337	18.5	0
**H'**·$AuCl_3$*5ext (TS)*	–2266.180600	–2265.871726	–2265.846646	174.4	–2265.923277	–89.0	1
**H'**·$AuCl_3$*5ext*	–2266.222335	–2265.909240	–2265.884837	168.9	–2265.960095	23.1	0
**H'**·$AuCl_3$*5ext*_hy *(preTS)*	–2495.528248	–2495.141800	–2495.108096	212.5	–2495.201173	16.3	0
**H'**·$AuCl_3$*5ext*_hy *(TS)*	–2495.501746	–2495.116026	–2495.085331	196.7	–2495.172202	–496.8	1
**H'**·$AuCl_3$*5ext*_hy *(postTS)*	–2495.506595	–2495.118538	–2495.088140	197.6	–2495.174446	11.9	0
**H'**·$AuCl_3$*5ext*_hy_{zw}	–2342.185292	–2341.858279	–2341.832478	175.1	–2341.910341	20.6	0
**H'**·$AuCl_3$*5ext*_hy_{zw} *(TS)*	–2342.184434	–2341.858531	–2341.832979	174.4	–2341.910518	–210.8	1

**H'**·$AuCl_3$*5ext*_hy_{zw} *(postTS)*	–2342.199880	–2341.875170	–2341.848001	184.2	–2341.929283	15.0	0
**H'**·$AuCl_3$*5ext*_lac	–2207.092009	–2206.860389	–2206.839009	156.9	–2206.908963	25.5	0
**H'**·$AuCl_2$*5ext*_lacI	–1746.696076	–1746.465540	–1746.446297	146.2	–1746.511532	25.5	0
**H'**·$AuCl_2$*5ext*_lacII	–1746.676758	–1746.445567	–1746.426625	141.3	–1746.491366	43.1	0
**H'**·$AuCl_2$*5ext*_lacII *(TS)*	–1746.644705	–1746.417971	–1746.398607	144.4	–1746.464075	–212.3	1
**H'**·$AuCl_2$*5ext*_ lacβ_{elim} *(TS)*	–1746.664164	–1746.436278	–1746.416472	145.7	–1746.482618	34.3	0
**H'**·$AuCl_3$*6ent (TS)*	–2266.184222	–2265.874731	–2265.849868	170.1	–2265.926499	–75.8	1
**H'**·$AuCl_3$*6ent*	–2266.216901	–2265.904103	–2265.879405	170.1	–2265.955379	27.7	0
**H'**·$AuCl_3$*6ent*_hy_{zw}	–2342.184694	–2341.857066	–2341.831278	175.7	–2341.909103	21.0	0
**H'**·$AuCl_3$*6ent*_hy_{zw} *(TS)*	–2342.179481	–2341.854188	–2341.828186	178.0	–2341.906663	–134.9	1
**H'**·$AuCl_3$*6ent*_hy_{zw} *(postTS)*	–2342.193932	–2341.869438	–2341.842132	187.4	–2341.923192	19.6	0
**H'**·$AuCl_3$*6ent*_lac	–2207.089190	–2206.857392	–2206.836147	155.7	–2206.905411	21.4	0
**H'**·$AuCl_2$*6ent*_lacII	–1746.679311	–1746.448344	–1746.429217	144.4	–1746.494013	33.0	0

[a]Energy values calculated at the SMD(H_2O)/M06/6-311G(d,p)+SDD(Au) level of theory. 1 Hartree = 627.51 kcal mol^{-1}. Thermal corrections at 298.15 K.

Cartesian coordinates of the lowest energy structures calculated SMD(H_2O)/M06/6-311G(d,p)+SDD(Au)

```
Structure Na[AuCl4]
 Au      0.2745     0.0000     0.0000
 Cl      1.9304    -1.6516     0.0000
 Cl     -1.3953     1.6854     0.0000
 Cl      1.9302     1.6518     0.0000
 Cl     -1.3951    -1.6854     0.0000
 Na     -3.6254    -0.0002     0.0000

Structure NaCl
 Cl      0.0000     0.0000     1.0058
 Na      0.0000     0.0000    -1.5544

Structure (H2O)3
 O       1.2811     1.0154    -0.0916
 O      -1.5207     0.6002    -0.0918
 O       0.2392    -1.6160    -0.0912
 H      -0.6270     0.9899    -0.1029
 H       1.1773     0.0464    -0.1013
 H      -0.5468    -1.0394    -0.0989
 H      -1.7821     0.6535     0.8325
 H       1.4507     1.2183     0.8334
 H       0.3311    -1.8653     0.8334

Structure [H3O·H2O]+
 O      -1.1950    -0.0796     0.0618
 H      -1.5275    -0.2970    -0.8200
 H      -1.5837     0.7753     0.2946
 O       1.1937     0.0705     0.0700
 H       1.5362     0.4001    -0.7718
 H       1.5875    -0.8030     0.2032
 H      -0.0017    -0.0028     0.0396

Structure NHMe2
 H       0.0000     1.2945     0.5701
 C       1.1905    -0.2256     0.0194
 H       2.0902     0.3929    -0.0321
 H       1.2073    -0.7751     0.9766
 H       1.2545    -0.9702    -0.7819
 C      -1.1905    -0.2256     0.0194
 H      -1.2073    -0.7751     0.9766
 H      -2.0902     0.3929    -0.0321
 H      -1.2545    -0.9702    -0.7819
 N       0.0000     0.5882    -0.1612

Structure NHMePh
 C      -0.7810     1.3658    -0.0191
 N       1.7819    -0.6109    -0.1708
 C       2.8042     0.3665     0.1250
 H       2.8285     1.1558    -0.6334
 H       2.6717     0.8468     1.1060
 H       3.7769    -0.1268     0.1142
 C       0.4462    -0.2761    -0.0711
 C       0.0074     1.0544    -0.0670
 C      -0.5195    -1.2935    -0.0062
 C      -1.3501     1.3450    -0.0140
 H       0.7275     1.8654    -0.1089
 C      -1.8665    -0.9893     0.0510
 H      -0.1882    -2.3296    -0.0021
 C      -2.2991     0.3347     0.0457
 H      -1.6653     2.3849    -0.0143
 H      -2.5911    -1.7973     0.1032
 H      -3.3573     0.5708     0.0903
 H       1.9882    -1.5444     0.1603

Structure B'
 C      -0.7726    -0.4684    -0.0790
 O      -0.7555    -1.6994    -0.0350
 C       2.9877     0.2638    -0.3485
 C       1.7484    -0.5525    -0.1971
 C       0.5077     0.3253    -0.1801
 H       3.0367     0.8938    -1.2392
 H       1.6691    -1.2607    -1.0329
 H       1.8036    -1.1503     0.7205
 H       0.5579     1.0315     0.6598
 H       0.4767     0.9457    -1.0859
 C       3.9962     0.2760     0.5123
 H       4.8775     0.8910     0.3552
 H       3.9773    -0.3367     1.4121
 N      -1.9289     0.2371    -0.0467
 C      -1.9874     1.6841     0.0493
 H      -2.9332     2.0239    -0.3805
 H      -1.9463     2.0227     1.0925
 H      -1.1815     2.1606    -0.5076
 C      -3.1872    -0.4380     0.2080
 H      -3.5610    -0.1890     1.2088
 H      -3.9372    -0.1238    -0.5244
 H      -3.0503    -1.5150     0.1388
```

Structure **B'·AuCl$_3$** ***(carbonyl)***

C	-2.1333	0.2530	0.5188
O	-1.0142	0.1358	1.1338
C	-0.4738	2.5990	-1.0583
C	-1.5145	2.6803	0.0104
C	-2.5578	1.5717	-0.0501
H	-0.8466	2.4086	-2.0669
H	-1.0429	2.7189	0.9995
H	-2.0590	3.6257	-0.1136
H	-3.4541	1.8802	0.5039
H	-2.8930	1.4175	-1.0852
C	0.8306	2.7574	-0.8669
H	1.5381	2.7103	-1.6903
H	1.2388	2.9478	0.1246
N	-2.9567	-0.7728	0.4651
C	-4.2558	-0.7038	-0.1941
H	-4.8731	-1.5193	0.1831
H	-4.7596	0.2388	0.0165
H	-4.1348	-0.8195	-1.2768
C	-2.5550	-2.1027	0.9051
H	-3.2005	-2.4237	1.7271
H	-2.6791	-2.7979	0.0701
H	-1.5192	-2.1021	1.2345
Au	0.7675	-0.2913	0.0868
Cl	1.9338	0.4068	2.0066
Cl	2.7750	-0.7587	-0.9749
Cl	-0.4255	-1.0173	-1.8201

Structure **B'·AuCl$_3$*(alkene)***

C	2.2078	-0.3538	1.1503
O	1.2892	-0.0730	1.9297
C	0.1333	-2.2367	-0.1630
C	1.5838	-2.2296	-0.4012
C	2.4388	-1.8028	0.7909
H	-0.2025	-2.5474	0.8285
H	1.7972	-3.2960	-0.5994
H	1.8371	-1.6982	-1.3251
H	2.1435	-2.3849	1.6689
H	3.4860	-2.0267	0.5829
C	-0.8267	-2.0062	-1.1367
H	-0.5147	-1.8480	-2.1662
H	-1.8455	-2.3466	-0.9704
Au	-0.9462	0.0317	-0.2512
Cl	-1.3939	2.2658	0.4089
Cl	0.5375	0.8174	-1.9059
Cl	-2.4371	-0.7678	1.3911
N	2.9953	0.5970	0.6089
C	3.8797	0.3983	-0.5261
H	3.4261	0.8186	-1.4328
H	4.8287	0.9120	-0.3464
H	4.0878	-0.6549	-0.7020
C	2.6824	1.9906	0.8808
H	3.5884	2.5867	0.7521
H	1.9148	2.3595	0.1875
H	2.3204	2.1084	1.9014

Structure **B'·AuCl$_3$*5ext*** ***(TS)***

C	-2.8042	0.0491	0.1548
O	-1.6183	0.3624	0.3593
C	-0.7914	-1.8260	0.7730
C	-1.8723	-2.1958	-0.1343
C	-3.1491	-1.4194	0.0871
H	-1.0514	-1.5291	1.7890
H	-1.5233	-2.1564	-1.1734
H	-2.0413	-3.2674	0.0800
H	-3.8733	-1.6315	-0.7021
H	-3.6197	-1.6983	1.0390
C	0.5711	-2.0731	0.4800
H	1.1990	-2.2995	1.3405
H	0.7627	-2.6631	-0.4151
Au	1.2487	-0.0982	-0.0164
Cl	2.1269	2.0673	-0.5776
Cl	1.5259	0.3335	2.2878
Cl	0.9320	-0.6034	-2.2986
N	-3.7767	0.9515	0.0184
C	-5.1747	0.6041	-0.1859
H	-5.7908	1.3786	0.2765
H	-5.4220	0.5557	-1.2520
H	-5.4251	-0.3446	0.2872
C	-3.4785	2.3741	0.0006
H	-3.9265	2.8261	-0.8895
H	-3.8973	2.8632	0.8858
H	-2.4023	2.5327	-0.0222

Structure **B'·AuCl$_3$*5ext***

C	-2.4151	-0.5795	-0.3297
O	-2.1752	-0.9700	0.8893
C	-1.0993	-1.9738	0.8968
C	-1.0962	-2.5081	-0.5265
C	-1.6600	-1.3536	-1.3488
H	-1.4276	-2.7292	1.6197
H	-0.0959	-2.8060	-0.8436
H	-1.7597	-3.3728	-0.5923
H	-0.8768	-0.6996	-1.7606
H	-2.2995	-1.6704	-2.1742
C	0.1597	-1.3658	1.4393
H	-0.0100	-0.8781	2.4008
H	0.9305	-2.1341	1.5275
Au	1.0127	0.1282	0.2387
Cl	1.9910	1.9115	-1.2216
Cl	-0.3357	1.7150	1.3655
Cl	2.3679	-1.5289	-0.7741
N	-3.2327	0.4024	-0.5487
C	-3.4289	0.9565	-1.8847
H	-4.4897	1.1753	-2.0177
H	-2.8583	1.8863	-1.9718
H	-3.1071	0.2580	-2.6538
C	-3.8514	1.1643	0.5331
H	-3.4299	2.1732	0.5394
H	-4.9247	1.2269	0.3423
H	-3.6752	0.6850	1.4923

Structure **B'·AuCl$_3$*5ext*_hy** ***(preTS)***

C	3.0935	-0.5025	0.2065
O	2.2488	-1.0552	-0.6146
C	1.3745	-1.9767	0.1254
C	1.4563	-1.4801	1.5543
C	2.8601	-0.8925	1.6208
H	1.8605	-2.9608	0.0340
H	0.7111	-0.6976	1.7229
H	1.2900	-2.2862	2.2695
H	2.9568	-0.0439	2.2995
H	3.6151	-1.6404	1.8958
C	0.0587	-2.0843	-0.5593
H	0.1698	-2.1882	-1.6405
H	-0.5064	-2.9226	-0.1499
Au	-1.2411	-0.4501	-0.3478
Cl	-2.9851	1.3702	-0.2495

Cl	-0.1649	0.6136	-2.1794
Cl	-2.2358	-1.5938	1.4669
N	4.0443	0.2497	-0.2547
C	4.9135	0.9929	0.6504
H	5.8644	1.1658	0.1453
H	4.4529	1.9560	0.8924
H	5.0992	0.4288	1.5630
C	4.0477	0.7018	-1.6426
H	3.6837	1.7339	-1.6759
H	5.0700	0.6708	-2.0221
H	3.4104	0.0681	-2.2550
O	1.5890	1.7741	0.4413
H	1.1237	1.4881	-0.3564
O	-0.3994	1.7686	2.4689
H	-0.7050	2.5453	1.9572
H	0.0844	2.1420	3.2122
H	0.9218	1.6896	1.1471
O	-1.0951	3.7509	0.6523
H	-1.7230	3.1403	0.2279
H	-1.6554	4.4365	1.0297

Structure **B'·AuCl$_3$**5*ext*_**hy** ***(TS)***

C	2.8023	-0.3618	0.1476
O	2.1077	-1.0344	-0.8153
C	1.2075	-1.9984	-0.2216
C	1.3073	-1.7855	1.2830
C	2.6721	-1.1327	1.4395
H	1.6162	-2.9891	-0.4837
H	0.5151	-1.1162	1.6294
H	1.2197	-2.7272	1.8272
H	2.7615	-0.4993	2.3241
H	3.4731	-1.8785	1.4585
C	-0.1349	-1.9436	-0.8718
H	-0.0656	-1.8545	-1.9580
H	-0.7379	-2.8081	-0.5899
Au	-1.3435	-0.3009	-0.3423
Cl	-2.9633	1.5854	0.1651
Cl	-0.2216	1.0339	-1.9672
Cl	-2.3572	-1.6870	1.2814
N	4.0768	0.0018	-0.2856
C	4.8635	0.6936	0.7271
H	5.8701	0.8541	0.3353
H	4.4364	1.6735	0.9872
H	4.9503	0.0925	1.6338
C	4.0937	0.7321	-1.5502
H	3.7093	1.7567	-1.4387
H	5.1242	0.7919	-1.9066
H	3.5022	0.2117	-2.3038
O	1.9708	0.9758	0.3695
H	1.3638	1.0905	-0.3944
O	0.5389	1.3779	2.3046
H	0.1015	2.2029	1.9690
H	1.0294	1.6272	3.0970
H	1.3793	1.0944	1.2769
O	-0.5122	3.4279	0.9647
H	-1.3305	3.0005	0.6516
H	-0.8133	4.1890	1.4716

Structure **B'·AuCl$_3$**5*ext*_**hy** ***(postTS)***

C	2.8129	-0.2511	0.1813
O	2.1526	-1.0488	-0.7633
C	1.2668	-1.9693	-0.1146
C	1.3605	-1.6687	1.3766
C	2.7254	-1.0103	1.4920
H	1.6706	-2.9812	-0.3053
H	0.5710	-0.9732	1.6762
H	1.2619	-2.5741	1.9786
H	2.8181	-0.3516	2.3587
H	3.5255	-1.7565	1.5226
C	-0.0802	-1.9868	-0.7601
H	-0.0199	-1.9606	-1.8502
H	-0.6696	-2.8399	-0.4202
Au	-1.3314	-0.3401	-0.3393
Cl	-3.0390	1.5366	0.0139
Cl	-0.2311	0.9195	-2.0274
Cl	-2.3352	-1.6426	1.3617
N	4.1445	0.0072	-0.2907
C	4.9311	0.7438	0.6896
H	5.9394	0.8901	0.2939
H	4.5091	1.7372	0.9127
H	5.0208	0.1847	1.6235
C	4.1494	0.7301	-1.5583
H	3.7805	1.7637	-1.4585
H	5.1738	0.7763	-1.9365
H	3.5395	0.2119	-2.3004
O	2.0531	0.9624	0.3385
H	1.5647	1.1186	-0.4883
O	0.3615	1.6661	2.2511
H	-0.1789	2.5355	1.6638
H	0.7992	2.0015	3.0450
H	1.0692	1.3342	1.6338
O	-0.7337	3.3398	0.9375
H	-1.5359	2.8873	0.5817
H	-1.0446	4.1052	1.4394

Structure **B'·AuCl$_3$**5*ext*_**hy**$_{\mathbf{hem}}$

C	-2.6569	0.3180	0.2403
O	-2.0481	0.3779	-1.0268
C	-1.1378	1.4815	-1.0880
C	-1.2396	2.1943	0.2591
C	-2.5944	1.7364	0.7743
H	-1.5162	2.1543	-1.8799
H	-0.4443	1.8639	0.9336
H	-1.1611	3.2778	0.1477
H	-2.6806	1.7753	1.8618
H	-3.4078	2.3182	0.3288
C	0.2152	1.0611	-1.5748
H	0.1534	0.4151	-2.4530
H	0.8480	1.9281	-1.7740
Au	1.3059	-0.0877	-0.1873
Cl	2.5724	-1.4391	1.5336
Cl	0.2360	-2.0527	-1.0062
Cl	2.4365	1.8993	0.4398
N	-3.9895	-0.2084	0.0628
C	-4.7353	-0.2050	1.3137
H	-5.7416	-0.5893	1.1265
H	-4.2717	-0.8385	2.0875
H	-4.8370	0.8083	1.7088
C	-3.9742	-1.5605	-0.4843
H	-3.5474	-2.2996	0.2141
H	-5.0003	-1.8667	-0.7041
H	-3.4068	-1.5937	-1.4162
O	-1.8512	-0.4864	1.0948
H	-1.4585	-1.1816	0.5451

Structure **B'·AuCl$_3$**5*ext*_**hy**$_{\mathbf{zw}}$

C	2.9485	0.4239	0.5413
O	1.5747	0.8055	0.8492
C	1.1140	1.8250	-0.0362
C	2.3816	2.3550	-0.7107
C	3.2296	1.0995	-0.8080
H	0.6226	2.5925	0.5709

H	2.1797	2.8318	-1.6735
H	2.8594	3.0893	-0.0527
H	2.9033	0.4848	-1.6554
H	4.2990	1.2942	-0.9087
C	0.1496	1.3066	-1.0719
H	-0.4402	2.1094	-1.5210
H	0.6216	0.6941	-1.8431
Au	-1.2667	0.0229	-0.2096
Cl	-2.9389	-1.5176	0.8781
Cl	-2.2394	1.9351	0.7763
Cl	-0.2158	-1.8018	-1.3231
N	2.7120	-1.2126	0.2109
C	3.9659	-1.8154	-0.2599
H	4.7235	-1.6757	0.5139
H	3.8231	-2.8827	-0.4433
H	4.2907	-1.3312	-1.1818
C	2.2051	-1.9093	1.4016
H	2.1627	-2.9844	1.2114
H	2.8874	-1.7092	2.2305
H	1.2083	-1.5414	1.6463
O	3.7604	0.5444	1.4939
H	1.9964	-1.2698	-0.5233

Structure **B'·AuCl$_3$*5ext*_hy$_{zw}$** ***(TS)***

C	2.9245	0.3754	0.5934
O	1.6640	0.9781	0.8357
C	1.2780	1.8316	-0.2472
C	2.5915	2.1348	-0.9714
C	3.3564	0.8314	-0.8018
H	0.8392	2.7259	0.2024
H	2.4392	2.4249	-2.0142
H	3.1029	2.9548	-0.4560
H	3.0683	0.1130	-1.5753
H	4.4413	0.9491	-0.8273
C	0.2892	1.2010	-1.1965
H	-0.2716	1.9577	-1.7522
H	0.7455	0.4853	-1.8840
Au	-1.1926	0.0812	-0.2211
Cl	-2.9286	-1.2821	0.9883
Cl	-1.8232	2.0516	0.9234
Cl	-0.5366	-1.8077	-1.5148
N	2.3378	-1.3933	0.2863
C	3.5072	-2.2303	0.0418
H	4.1752	-2.1552	0.9042
H	3.2239	-3.2794	-0.0959
H	4.0375	-1.8852	-0.8491
C	1.5805	-1.8482	1.4477
H	1.3751	-2.9232	1.3885
H	2.1709	-1.6487	2.3476
H	0.6322	-1.3084	1.5113
O	3.6965	0.2962	1.5584
H	1.7262	-1.3949	-0.5340

Structure **B'·AuCl$_3$*5ext*_hy$_{zw}$** ***(postTS)***

C	-3.1171	0.3152	-0.5252
O	-2.0716	0.8878	-1.1520
C	-1.4793	1.9127	-0.3153
C	-1.9684	1.5996	1.0913
C	-3.3076	0.9451	0.8202
H	-1.9174	2.8699	-0.6472
H	-1.2946	0.8903	1.5770
H	-2.0238	2.5024	1.7025
H	-3.6272	0.2047	1.5551
H	-4.1122	1.6842	0.7108
C	-0.0109	2.0306	-0.5689
H	0.2104	2.2198	-1.6205
H	0.4227	2.8087	0.0629
Au	1.0731	0.2927	-0.1197
Cl	2.2840	-1.8481	0.4524
Cl	0.9314	-0.3289	-2.4012
Cl	1.3500	1.0943	2.0954
N	-1.3491	-1.8874	0.7094
C	-2.2337	-2.2590	1.7942
H	-3.2779	-2.1547	1.4720
H	-2.0994	-3.3012	2.1357
H	-2.0801	-1.5989	2.6545
C	-1.4559	-2.7941	-0.4160
H	-1.2649	-3.8495	-0.1503
H	-2.4662	-2.7396	-0.8394
H	-0.7494	-2.5041	-1.2018
O	-3.7837	-0.5313	-1.0648
H	-0.3847	-1.8874	1.0371

Structure **B'·AuCl$_3$*5ext*_lac**

C	-2.7200	-0.5214	0.8280
O	-2.7247	-0.3490	-0.5067
C	-2.1449	0.9263	-0.8690
C	-2.2170	1.7598	0.4084
C	-2.1916	0.7084	1.5025
H	-2.7966	1.3413	-1.6498
H	-1.3995	2.4801	0.4689
H	-3.1645	2.3041	0.4301
H	-1.1706	0.4767	1.8385
H	-2.7862	0.9417	2.3870
C	-0.8037	0.7381	-1.5160
H	-0.8471	0.0110	-2.3291
H	-0.4148	1.6939	-1.8725
Au	0.6910	-0.0102	-0.2446
Cl	2.4558	-0.8991	1.3111
Cl	0.0220	-2.2044	-0.8329
Cl	1.3467	2.2268	0.1911
O	-3.0960	-1.5517	1.3253

Structure **B'·AuCl$_3$*6ent*** ***(TS)***

C	3.6234	-0.0292	-0.1073
O	3.0296	0.2056	-1.1760
C	0.7415	0.2248	0.2696
C	1.4491	-0.9349	0.8863
C	2.8998	-0.5220	1.1263
H	0.7737	1.1541	0.8402
H	1.0052	-1.2087	1.8477
H	1.4061	-1.8072	0.2285
H	2.9458	0.2698	1.8860
H	3.4409	-1.3764	1.5450
C	0.7363	0.3377	-1.1172
H	0.7928	-0.5481	-1.7418
Au	-1.4655	0.0334	-0.0044
Cl	-1.5973	2.3734	0.2461
Cl	-1.3135	-2.2870	-0.3956
Cl	-3.8601	-0.1421	0.1427
N	4.9474	0.1587	0.0048
C	5.7075	-0.1747	1.2000
H	5.9752	-1.2372	1.2244
H	6.6283	0.4113	1.1912
H	5.1628	0.0784	2.1093
C	5.7453	0.5356	-1.1502
H	6.3148	1.4433	-0.9299
H	6.4517	-0.2653	-1.3933
H	5.1004	0.7151	-2.0072
H	0.5955	1.2971	-1.6085

Structure **B'·AuCl$_3$*6ent***

C	3.4309	0.0255	0.0162

O	2.7824	-0.3375	-1.0500
C	0.6776	0.3206	-0.0277
C	1.2746	0.0713	1.3288
C	2.7477	0.4649	1.2645
H	0.7753	1.3724	-0.3136
H	0.7872	0.6668	2.1050
H	1.1802	-0.9855	1.6015
H	2.8483	1.5595	1.2945
H	3.2948	0.0784	2.1283
C	1.3302	-0.5555	-1.0512
H	1.1900	-1.6200	-0.8523
Au	-1.4069	0.0257	-0.0264
Cl	-1.6371	2.3627	-0.2979
Cl	-1.1005	-2.2943	0.3290
Cl	-3.8800	-0.2617	0.0020
N	4.7332	0.0358	-0.0788
C	5.5859	0.5396	0.9975
H	5.8225	-0.2594	1.7051
H	6.5098	0.9018	0.5468
H	5.1120	1.3690	1.5199
C	5.4295	-0.4337	-1.2760
H	5.7364	0.4196	-1.8862
H	6.3176	-0.9813	-0.9564
H	4.7896	-1.0909	-1.8591
H	1.0302	-0.3264	-2.0734

Structure **B'·AuCl$_3$*6ent*_hy$_{zw}$**

C	3.2367	-0.2020	0.2175
O	2.5967	-0.1947	-1.1033
C	0.5030	0.3873	-0.2109
C	1.0725	0.5451	1.1742
C	2.5622	0.8910	1.0463
H	0.5154	1.3383	-0.7531
H	0.5564	1.3346	1.7299
H	0.9693	-0.3901	1.7395
H	2.6736	1.8728	0.5678
H	3.0162	0.9330	2.0414
C	1.2535	-0.6525	-1.0118
H	1.2248	-1.6380	-0.5355
Au	-1.5762	0.0199	-0.0575
Cl	-1.8649	2.3649	-0.2948
Cl	-1.2105	-2.2989	0.2799
Cl	-4.0836	-0.3176	0.1567
N	4.6886	0.4333	-0.2897
C	5.5162	0.8262	0.8625
H	6.5222	1.0793	0.5232
H	5.0781	1.6880	1.3645
H	5.5645	-0.0220	1.5493
C	5.4002	-0.5404	-1.1354
H	4.7891	-0.7876	-2.0013
H	6.3517	-0.1137	-1.4577
H	5.5810	-1.4379	-0.5421
H	4.4553	1.2582	-0.8470
O	3.3996	-1.3333	0.7481
H	0.8699	-0.7446	-2.0315

Structure **B'·AuCl$_3$*6ent*_hy$_{zw}$** ***(TS)***

C	3.1235	-0.4232	0.2804
O	2.5852	-0.3629	-1.0192
C	0.4834	0.3126	-0.1537
C	1.0323	0.3834	1.2488
C	2.5475	0.6545	1.1543
H	0.5597	1.2828	-0.6541
H	0.5534	1.1798	1.8266
H	0.8673	-0.5660	1.7751
H	2.7118	1.6408	0.7091
H	3.0075	0.6202	2.1453
C	1.1931	-0.7315	-0.9832
H	1.0988	-1.7363	-0.5572
Au	-1.6112	0.0381	-0.0579
Cl	-1.7931	2.3940	-0.2671
Cl	-1.3530	-2.3011	0.2213
Cl	-4.1182	-0.1913	0.1072
N	4.9132	0.6071	-0.3462
C	5.6883	0.9251	0.8389
H	6.7084	1.2554	0.5966
H	5.1965	1.7123	1.4153
H	5.7632	0.0282	1.4661
C	5.6025	-0.3205	-1.2220
H	5.0346	-0.4611	-2.1440
H	6.6163	0.0184	-1.4770
H	5.6850	-1.2895	-0.7150
H	4.6471	1.4513	-0.8463
O	3.5476	-1.4843	0.7005
H	0.8460	-0.7503	-2.0181

Structure **B'·AuCl$_3$*6ent*_hy$_{zw}$** ***(postTS)***

C	3.2093	1.3887	-0.0820
O	2.4757	1.2558	-1.2052
C	0.4349	0.9349	0.0660
C	1.2024	0.5996	1.3134
C	2.5124	1.3791	1.2473
H	0.1611	1.9940	0.0486
H	0.6490	0.8864	2.2128
H	1.4112	-0.4724	1.3672
H	2.3206	2.4415	1.4579
H	3.2291	1.0367	1.9983
C	1.2006	0.5758	-1.1766
H	1.3988	-0.4983	-1.2360
Au	-1.4409	-0.0315	0.0063
Cl	-2.4296	2.1189	-0.0519
Cl	-0.3677	-2.1485	0.0535
Cl	-3.6960	-1.1407	-0.0301
N	3.5968	-1.2746	0.0285
C	4.5101	-1.4072	1.1445
H	3.9647	-1.3410	2.0914
H	5.0744	-2.3559	1.1400
H	5.2429	-0.5905	1.1188
C	4.2587	-1.4650	-1.2455
H	4.9590	-0.6382	-1.4216
H	4.8332	-2.4054	-1.3050
H	3.5253	-1.4610	-2.0580
H	0.6836	0.8969	-2.0833
H	2.8375	-1.9436	0.1247
O	4.3713	1.7096	-0.2036

Structure **B'·AuCl$_3$*6ent*_lac**

C	3.9995	0.0533	-0.0325
O	3.3150	-0.5104	-1.0392
C	1.2092	0.2620	-0.1391
C	1.7833	0.3318	1.2483
C	3.2587	0.6967	1.1044
H	1.3031	1.2203	-0.6602
H	1.2823	1.0839	1.8646
H	1.6776	-0.6390	1.7474
H	3.3550	1.7740	0.9118
H	3.8244	0.4968	2.0183
C	1.8983	-0.8147	-0.9256
H	1.8171	-1.7925	-0.4453
Au	-0.8821	0.0142	-0.0443
Cl	-1.0325	2.3692	-0.2606
Cl	-0.6613	-2.3243	0.2513

Cl	-3.3748	-0.1740	0.1116
O	5.2048	0.0910	-0.1180
H	1.5402	-0.8886	-1.9536

Structure **B'·AuCl$_3$*6ent*_acid**

C	2.4520	-0.6386	1.0363
O	1.4353	-0.2745	1.6841
C	1.1660	1.9698	-0.5470
C	2.4578	1.7363	0.0896
C	3.0726	0.3573	0.0489
H	0.6272	2.8532	-0.2067
H	3.0946	2.4556	-0.4682
H	2.4364	2.1409	1.1069
H	3.0225	-0.0881	-0.9523
H	4.1391	0.4298	0.2844
C	0.5979	1.2567	-1.6006
H	-0.1968	1.7435	-2.1636
H	1.2034	0.5315	-2.1368
Au	-0.6220	0.0224	-0.2377
Cl	-2.0742	-1.4228	0.9872
Cl	-1.6627	1.8870	0.7923
Cl	0.2493	-1.8607	-1.3810
O	3.0173	-1.7482	1.1131

Structure **K**

C	-0.7810	1.3658	-0.0191
O	-1.4728	2.3804	-0.0036
C	-3.4648	-1.3504	-0.4103
C	-2.9059	0.0221	-0.2400
C	-1.3890	-0.0087	-0.1513
H	-3.1283	-1.8959	-1.2944
H	-3.1983	0.6498	-1.0925
H	-3.3280	0.4889	0.6580
H	-1.0720	-0.6223	0.7023
H	-0.9662	-0.4999	-1.0382
C	-4.3071	-1.9329	0.4318
H	-4.6818	-2.9388	0.2656
H	-4.6579	-1.4181	1.3247
N	0.5737	1.4165	0.0866
C	1.2725	2.6859	0.2119
H	1.8918	2.6859	1.1150
H	1.9207	2.8532	-0.6554
H	0.5446	3.4932	0.2760
C	1.4090	0.2558	0.0446
C	1.9218	-0.1807	-1.1709
C	1.7468	-0.3968	1.2237
C	2.7629	-1.2840	-1.2071
H	1.6535	0.3476	-2.0819
C	2.5877	-1.5002	1.1833
H	1.3430	-0.0363	2.1659
C	3.0946	-1.9449	-0.0312
H	3.1600	-1.6288	-2.1566
H	2.8476	-2.0143	2.1032
H	3.7521	-2.8082	-0.0609

Structure **K·AuCl$_3$** ***(alkene)***

C	1.2872	-0.6131	1.3525
O	0.2061	-0.4296	1.9170
C	-0.5765	-2.1607	-0.5508
C	0.8898	-2.0919	-0.6002
C	1.5904	-1.9617	0.7508
H	-1.0175	-2.6855	0.2989
H	1.1579	-3.0775	-1.0234
H	1.2362	-1.3510	-1.3304
H	1.2000	-2.7177	1.4388
H	2.6567	-2.1407	0.6141
C	-1.4208	-1.7280	-1.5635
H	-0.9914	-1.3501	-2.4882
H	-2.4374	-2.1104	-1.6042
Au	-1.7348	0.0723	-0.2990
Cl	-2.3444	2.1110	0.7564
Cl	-0.1642	1.2312	-1.6253
Cl	-3.3488	-1.0874	0.9650
N	2.1762	0.3931	1.1948
C	1.7683	1.7385	1.5945
H	2.6400	2.3924	1.5646
H	0.9977	2.1305	0.9200
H	1.3695	1.7271	2.6111
C	3.4153	0.2588	0.4900
C	3.5396	0.7977	-0.7852
C	4.4991	-0.3616	1.0992
C	4.7512	0.7030	-1.4556
H	2.6838	1.2861	-1.2441
C	5.7048	-0.4621	0.4201
H	4.3867	-0.7678	2.1003
C	5.8328	0.0707	-0.8562
H	4.8484	1.1211	-2.4525
H	6.5494	-0.9520	0.8940
H	6.7784	-0.0047	-1.3837

Structure **K·AuCl$_3$*5ext*** ***(TS)***

C	-1.7723	0.4144	-0.4160
O	-0.5951	0.2927	-0.7899
C	0.2996	2.1382	0.2996
C	-0.8837	2.0392	1.1498
C	-2.1313	1.6310	0.3979
H	0.1848	2.5645	-0.6962
H	-0.6795	1.3917	2.0107
H	-1.0060	3.0598	1.5555
H	-2.9667	1.4501	1.0761
H	-2.4383	2.4198	-0.3000
C	1.6117	1.9665	0.8105
H	2.3772	2.5721	0.3283
H	1.6823	1.8979	1.8957
Au	2.1360	-0.0018	0.1457
Cl	2.8447	-2.1910	-0.5542
Cl	2.9110	0.9522	-1.8683
Cl	1.2720	-0.8883	2.1582
N	-2.6925	-0.5136	-0.7092
C	-2.2502	-1.7166	-1.4160
H	-3.1046	-2.3787	-1.5484
H	-1.8471	-1.4574	-2.3986
H	-1.4754	-2.2354	-0.8441
C	-4.0766	-0.3752	-0.3728
C	-4.8277	0.6608	-0.9132
C	-4.6671	-1.3065	0.4735

C	-6.1704	0.7795	-0.5826
H	-4.3578	1.3688	-1.5896
C	-6.0122	-1.1889	0.7894
H	-4.0678	-2.1153	0.8819
C	-6.7642	-0.1439	0.2668
H	-6.7556	1.5924	-1.0003
H	-6.4731	-1.9137	1.4528
H	-7.8156	-0.0519	0.5196

Structure **K·AuCl$_3$*5ext***

C	-1.4290	1.5477	0.1229
O	-0.6037	2.4033	-0.3950
C	0.5710	2.5607	0.4844
C	0.0656	2.0883	1.8389
C	-1.0409	1.1005	1.4843
H	0.7816	3.6359	0.4741
H	0.8625	1.6369	2.4316
H	-0.3479	2.9358	2.3893
H	-0.6736	0.0663	1.4158
H	-1.8967	1.1051	2.1628
C	1.7554	1.8745	-0.1294
H	1.8909	2.1639	-1.1731
H	2.6513	2.0999	0.4521
Au	1.6517	-0.2251	-0.1741
Cl	1.4651	-2.7212	-0.1924
Cl	0.5122	-0.0779	-2.2473
Cl	2.8354	-0.2616	1.8735
N	-2.4793	1.1659	-0.5413
C	-2.7797	1.6314	-1.8971
H	-2.5797	0.8214	-2.6040
H	-3.8378	1.8975	-1.9404
H	-2.1708	2.4995	-2.1405
C	-3.2876	0.0830	-0.0359
C	-4.5139	0.3524	0.5490
C	-2.8212	-1.2133	-0.1949
C	-5.2846	-0.7096	1.0010
H	-4.8528	1.3789	0.6502
C	-3.6001	-2.2668	0.2605
H	-1.8572	-1.3819	-0.6702
C	-4.8282	-2.0139	0.8582
H	-6.2445	-0.5162	1.4682
H	-3.2465	-3.2862	0.1461
H	-5.4357	-2.8399	1.2138

Structure **K·AuCl$_3$*5ext*_hy** ***(preTS)***

C	-1.9885	1.6304	-0.6446
O	-1.5260	0.6307	-1.3253
C	-0.3389	1.0494	-2.0904
C	0.1166	2.3160	-1.3894
C	-1.1744	2.8608	-0.7888
H	-0.7241	1.2804	-3.0952
H	0.8226	2.0843	-0.5866
H	0.5899	3.0076	-2.0872
H	-1.0398	3.3751	0.1651
H	-1.7101	3.5376	-1.4678
C	0.5897	-0.1072	-2.2359
H	0.0671	-1.0125	-2.5499
H	1.3871	0.1409	-2.9376
Au	1.5759	-0.6570	-0.4697
Cl	2.7824	-1.3454	1.6295
Cl	-0.2787	-2.0269	0.1020
Cl	3.4131	0.6397	-1.2011
N	-3.0883	1.4951	0.0413
C	-3.5534	2.5836	0.9079
H	-4.6027	2.4071	1.1400
H	-2.9695	2.6066	1.8322
H	-3.4645	3.5380	0.3887
O	-0.6053	0.8991	1.7954
H	-0.5090	0.0839	1.2864
O	1.6383	2.6056	1.5929
H	1.9310	2.1619	2.4165
H	1.1632	3.3878	1.8901
H	0.1914	1.4235	1.5905
O	2.2942	1.0303	3.7561
H	2.3668	0.2446	3.1876
H	3.2021	1.2013	4.0265
C	-3.7049	0.1978	0.1760
C	-3.6980	-0.4102	1.4230
C	-4.2842	-0.4147	-0.9244
C	-4.2794	-1.6616	1.5648
H	-3.2319	0.0866	2.2679
C	-4.8576	-1.6687	-0.7716
H	-4.2809	0.0859	-1.8873
C	-4.8549	-2.2921	0.4694
H	-4.2762	-2.1470	2.5352
H	-5.3123	-2.1574	-1.6269
H	-5.3059	-3.2725	0.5839

Structure **K·AuCl$_3$*5ext*_hy** ***(TS)***

C	-1.8751	1.4876	0.3869
O	-1.4109	1.2139	-0.8616
C	-0.2261	1.9916	-1.1543
C	0.1043	2.7344	0.1329
C	-1.2401	2.7777	0.8464
H	-0.5288	2.7140	-1.9312
H	0.8369	2.1782	0.7244
H	0.5097	3.7269	-0.0693
H	-1.1664	2.8541	1.9330
H	-1.8631	3.5983	0.4761
C	0.8161	1.1385	-1.7983
H	0.3959	0.4877	-2.5684
H	1.6197	1.7530	-2.2066
Au	1.8068	-0.2222	-0.5330
Cl	3.1074	-1.8963	0.8569
Cl	-0.0190	-1.7462	-0.7223
Cl	3.5693	1.3397	-0.3934
N	-3.2683	1.3540	0.4770
C	-3.8098	1.7487	1.7757
H	-4.8889	1.5894	1.7658
H	-3.3785	1.1874	2.6146
H	-3.6312	2.8131	1.9417
O	-1.2532	0.3616	1.3089
H	-0.9751	-0.3858	0.7335
O	0.6597	0.6880	2.8058
H	0.7827	-0.2673	3.0394
H	0.4306	1.1463	3.6229

H	-0.4279	0.5964	1.9670
O	0.7454	-1.9766	2.9702
H	1.4448	-2.1128	2.3049
H	1.1027	-2.3636	3.7761
C	-3.7832	0.1223	-0.0615
C	-4.0769	-0.9606	0.7634
C	-3.9881	0.0114	-1.4341
C	-4.5575	-2.1431	0.2167
H	-3.9257	-0.8859	1.8356
C	-4.4577	-1.1753	-1.9768
H	-3.7788	0.8622	-2.0734
C	-4.7417	-2.2578	-1.1540
H	-4.7819	-2.9815	0.8689
H	-4.6113	-1.2503	-3.0489
H	-5.1130	-3.1848	-1.5796

Structure **K·AuCl$_3$*5ext*_hy** ***(postTS)***

C	-1.8877	1.4490	0.3891
O	-1.4547	1.2063	-0.9161
C	-0.2755	1.9685	-1.2027
C	0.0484	2.7419	0.0702
C	-1.3020	2.7952	0.7704
H	-0.5522	2.6838	-1.9993
H	0.7768	2.1968	0.6778
H	0.4595	3.7288	-0.1503
H	-1.2279	2.9258	1.8524
H	-1.9342	3.5863	0.3548
C	0.7830	1.1203	-1.8271
H	0.3795	0.4469	-2.5867
H	1.5881	1.7327	-2.2360
Au	1.7881	-0.2193	-0.5450
Cl	3.1635	-1.9128	0.7892
Cl	-0.0205	-1.7594	-0.7059
Cl	3.5368	1.3632	-0.4112
N	-3.3307	1.3640	0.4263
C	-3.8589	1.7766	1.7239
H	-4.9393	1.6202	1.7326
H	-3.4227	1.2339	2.5742
H	-3.6785	2.8439	1.8725
O	-1.2911	0.4708	1.2521
H	-1.1444	-0.3397	0.7324
O	0.7905	0.6316	2.8884
H	0.9551	-0.5272	3.0457
H	0.5816	1.0702	3.7241
H	-0.0086	0.7062	2.2999
O	1.0964	-1.7364	3.0581
H	1.7635	-1.9411	2.3597
H	1.4767	-2.0315	3.8964
C	-3.8226	0.0960	-0.0356
C	-4.1231	-0.9463	0.8405
C	-4.0055	-0.1019	-1.4037
C	-4.5849	-2.1630	0.3547
H	-3.9943	-0.8122	1.9097
C	-4.4575	-1.3206	-1.8865
H	-3.7939	0.7131	-2.0874
C	-4.7469	-2.3586	-1.0094
H	-4.8130	-2.9649	1.0506
H	-4.5944	-1.4560	-2.9552
H	-5.1057	-3.3108	-1.3874

Structure **K·AuCl$_3$*5ext*_hy$_{hem}$**

C	-1.6398	1.5283	0.3655
O	-1.2365	1.1567	-0.9258
C	-0.0093	1.8125	-1.2675
C	0.3155	2.7422	-0.0991
C	-1.0350	2.9026	0.5827
H	-0.2244	2.4220	-2.1641
H	1.0335	2.2748	0.5805
H	0.7400	3.6875	-0.4434
H	-0.9594	3.1644	1.6397
H	-1.6545	3.6460	0.0707
C	1.0309	0.8354	-1.7244
H	0.6287	0.1132	-2.4381
H	1.8997	1.3496	-2.1396
Au	1.8103	-0.3765	-0.1878
Cl	2.7211	-1.7866	1.7020
Cl	-0.0089	-1.8889	-0.4785
Cl	3.6275	1.1356	-0.0418
N	-3.0923	1.4825	0.4196
C	-3.5926	2.0432	1.6715
H	-4.6761	1.9139	1.7113
H	-3.1553	1.5860	2.5702
H	-3.3876	3.1160	1.7025
O	-1.0436	0.6563	1.3121
H	-1.0318	-0.2328	0.9216
C	-3.6221	0.1866	0.1054
C	-3.8218	-0.1583	-1.2317
C	-3.9524	-0.7412	1.0930
C	-4.3205	-1.4057	-1.5735
H	-3.5860	0.5672	-2.0030
C	-4.4611	-1.9872	0.7481
H	-3.8125	-0.4941	2.1406
C	-4.6413	-2.3280	-0.5847
H	-4.4703	-1.6543	-2.6200
H	-4.7125	-2.6969	1.5307
H	-5.0381	-3.3024	-0.8524

Structure **K·AuCl$_3$*5ext*_hy$_{zw}$** ***(postTS)***

C	1.9623	-2.2923	-0.7306
O	0.8174	-1.7490	-1.1889
C	-0.3400	-2.5066	-0.7479
C	0.2378	-3.8536	-0.2947
C	1.6509	-3.4868	0.1192
H	-0.9906	-2.6035	-1.6166
H	-0.3544	-4.3016	0.5056
H	0.2515	-4.5454	-1.1416
H	1.7110	-3.1677	1.1677
H	2.3960	-4.2681	-0.0347
C	-1.0357	-1.8300	0.3945
H	-1.9906	-2.3192	0.6098
H	-0.4110	-1.8040	1.2919
Au	-1.5945	0.1766	0.1195
Cl	-2.2409	2.5904	-0.1306
Cl	-2.1616	-0.2323	-2.1358
Cl	-1.1480	0.4904	2.4354
N	2.0768	0.0758	1.3323

C	2.8356	-0.6815	2.2911
H	3.5272	-1.3764	1.7972
H	3.4260	-0.0491	2.9717
H	2.1423	-1.2718	2.8960
O	3.0353	-1.8352	-1.0270
H	1.0937	0.2112	1.5334
C	2.6490	0.9758	0.4708
C	4.0386	1.1039	0.3362
C	1.8263	1.7827	-0.3357
C	4.5732	2.0063	-0.5745
H	4.7015	0.4937	0.9416
C	2.3743	2.6794	-1.2327
H	0.7448	1.6877	-0.2430
C	3.7561	2.8022	-1.3653
H	5.6535	2.0865	-0.6615
H	1.7116	3.2910	-1.8398
H	4.1838	3.5060	-2.0718

Structure **H'**

C	3.5794	0.1281	0.2520
O	3.6255	0.7382	1.3217
C	1.0794	2.0213	-0.3042
C	2.4825	2.1465	-0.7978
C	3.2043	0.8357	-1.0321
H	0.5595	2.9658	-0.1331
H	2.4661	2.7189	-1.7346
H	3.0518	2.7662	-0.0936
H	2.6016	0.1838	-1.6729
H	4.1358	1.0184	-1.5842
C	0.4184	0.8954	-0.0588
H	2.7507	-2.2530	-1.2825
N	3.8895	-1.1856	0.1575
C	3.7843	-1.9479	-1.0752
H	4.3910	-2.8504	-0.9745
H	0.8801	-0.0812	-0.2143
H	4.1678	-1.3898	-1.9303
C	4.1601	-1.9644	1.3514
H	3.4061	-2.7510	1.4701
H	5.1436	-2.4408	1.2835
H	4.1381	-1.3167	2.2254
C	-0.9739	0.8671	0.4558
H	-1.3742	1.8851	0.5609
H	-1.0203	0.3771	1.4397
O	-1.7655	0.1194	-0.4711
C	-3.0751	-0.0910	-0.1718
C	-3.7137	0.4113	0.9588
C	-3.7905	-0.8624	-1.0891
C	-5.0627	0.1335	1.1592
H	-3.1792	1.0153	1.6827
C	-5.1306	-1.1283	-0.8758
H	-3.2703	-1.2421	-1.9634
C	-5.7780	-0.6321	0.2524
H	-5.5540	0.5289	2.0433
H	-5.6759	-1.7298	-1.5970
H	-6.8295	-0.8418	0.4189

Structure **H'·AuCl$_3$**

C	-3.4310	0.8425	0.8544
O	-2.9026	0.1475	1.7299
C	-0.5590	2.0108	0.2400
C	-1.8693	2.5068	-0.2215
C	-3.0391	2.2966	0.7378
H	-0.3266	2.1430	1.2998
H	-1.6922	3.5951	-0.2915
H	-2.0853	2.1778	-1.2434
H	-2.7403	2.6149	1.7411
H	-3.8734	2.9278	0.4273
C	0.4649	1.6057	-0.6043
H	0.2709	1.5874	-1.6771
Au	-0.3483	-0.4463	0.0211
Cl	-0.8341	-2.7471	0.3235
Cl	-1.4956	-0.5026	-2.0401
Cl	0.7991	-0.4563	2.0833
N	-4.3377	0.3344	-0.0054
C	-4.7869	0.9901	-1.2212
H	-4.2854	0.5525	-2.0940
H	-5.8651	0.8468	-1.3372
H	-4.5841	2.0589	-1.2055
C	-4.6003	-1.0950	0.0314
H	-5.5973	-1.2820	-0.3739
H	-3.8642	-1.6414	-0.5736
H	-4.5570	-1.4635	1.0555
C	1.8951	1.6809	-0.2025
H	2.2845	2.6403	-0.5799
H	2.0113	1.6716	0.8880
O	2.5767	0.5981	-0.8029
C	3.8967	0.4302	-0.4918
C	4.6371	1.3300	0.2662
C	4.4908	-0.7282	-0.9887
C	5.9778	1.0575	0.5216
H	4.1916	2.2387	0.6546
C	5.8247	-0.9832	-0.7259
H	3.8886	-1.4141	-1.5769
C	6.5777	-0.0916	0.0326
H	6.5535	1.7629	1.1130
H	6.2806	-1.8883	-1.1152
H	7.6235	-0.2943	0.2383

Structure **H'·AuCl$_3$*5ext*** ***(TS)***

C	-3.4403	-1.3039	-0.2972
O	-2.4124	-0.7312	-0.7074
C	-0.8334	-1.8715	0.5360
C	-1.9535	-2.1403	1.4341
C	-3.2602	-2.4022	0.7212
H	-0.7630	-2.4491	-0.3869
H	-1.6344	-3.0449	1.9838
H	-2.0377	-1.3470	2.1863
H	-3.2300	-3.3558	0.1783
H	-4.0843	-2.4560	1.4337
C	0.3153	-1.1441	0.9554
H	0.3194	-0.9088	2.0221
Au	-0.1866	0.8228	0.1419
Cl	-0.6888	3.0341	-0.6718
Cl	-0.9830	1.4229	2.2848
Cl	0.6154	0.2385	-2.0057
N	-4.6531	-0.9767	-0.7364
C	-5.8679	-1.7162	-0.4333
H	-6.5299	-1.1257	0.2078
H	-6.3924	-1.9279	-1.3697
H	-5.6497	-2.6657	0.0507
C	-4.8088	0.1128	-1.6881
H	-4.8046	-0.2609	-2.7177
H	-5.7654	0.6055	-1.4994
H	-4.0052	0.8391	-1.5679
C	1.6447	-1.6026	0.4344
H	1.9292	-2.5237	0.9668
H	1.5970	-1.8276	-0.6377
O	2.5869	-0.5816	0.7023
C	3.8540	-0.7359	0.2222

C	4.7182	0.3348	0.4477
C	4.3016	-1.8663	-0.4533
C	6.0241	0.2732	-0.0033
H	4.3438	1.2051	0.9783
C	5.6186	-1.9112	-0.9012
H	3.6463	-2.7112	-0.6319
C	6.4838	-0.8514	-0.6828
H	6.6898	1.1119	0.1761
H	5.9638	-2.7956	-1.4282
H	7.5085	-0.8975	-1.0366

Structure **H'·AuCl$_3$*5ext***

C	-2.3819	2.0256	0.4872
O	-1.2371	2.1756	1.0908
C	-0.9958	1.0583	2.0108
C	-2.3751	0.4592	2.2375
C	-3.1610	0.8690	0.9957
H	-0.5973	1.5298	2.9171
H	-2.3284	-0.6228	2.3711
H	-2.8229	0.9017	3.1292
H	-3.1652	0.0938	0.2154
H	-4.1986	1.1396	1.1989
C	0.0863	0.1598	1.4725
H	0.2968	-0.5880	2.2434
Au	-0.5318	-1.0881	-0.1428
Cl	-1.3012	-2.5980	-1.9799
Cl	-0.3641	0.7028	-1.6952
Cl	-0.6677	-2.8606	1.4207
N	-2.7316	2.8469	-0.4517
C	-3.9392	2.6268	-1.2407
H	-4.3494	3.5978	-1.5213
H	-3.6790	2.0703	-2.1469
H	-4.6852	2.0757	-0.6712
C	-1.8261	3.8666	-0.9738
H	-1.6082	3.6325	-2.0196
H	-2.3211	4.8386	-0.9222
H	-0.9017	3.8917	-0.4024
C	1.3459	0.8987	1.1266
H	1.1905	1.6224	0.3178
H	1.6707	1.4541	2.0236
O	2.3276	-0.0545	0.7612
C	3.5377	0.4076	0.3342
C	4.4430	-0.5660	-0.0863
C	3.8952	1.7519	0.3080
C	5.6983	-0.1941	-0.5321
H	4.1404	-1.6083	-0.0547
C	5.1619	2.1088	-0.1449
H	3.2079	2.5222	0.6382
C	6.0668	1.1478	-0.5659
H	6.3959	-0.9595	-0.8582
H	5.4359	3.1593	-0.1631
H	7.0518	1.4376	-0.9168

Structure **H'·AuCl$_3$*5ext*_hy** ***(preTS)***

C	2.3156	2.5962	-0.4956
O	1.0565	2.2625	-0.4823
C	0.7552	1.4643	-1.6776
C	2.0979	0.8904	-2.0730
C	3.0551	1.9916	-1.6336
H	0.4221	2.1974	-2.4302
H	2.2981	-0.0284	-1.5133
H	2.1441	0.6759	-3.1411
H	4.0427	1.6356	-1.3391
H	3.1807	2.7638	-2.4041
C	-0.4039	0.5565	-1.4242
H	-0.6444	0.0627	-2.3708
Au	0.0228	-1.1349	-0.2005
Cl	0.2280	-3.2477	1.1569
Cl	-0.1493	0.1419	1.7970
Cl	0.2399	-2.3724	-2.2046
N	2.7693	3.4233	0.3939
C	4.1959	3.7074	0.4948
H	4.3170	4.7196	0.8823
H	4.6611	2.9978	1.1861
H	4.6747	3.6446	-0.4808
C	1.9727	3.7905	1.5611
H	2.2935	3.1787	2.4108
H	2.1492	4.8423	1.7904
H	0.9136	3.6302	1.3719
O	3.1049	0.5887	1.1601
H	2.1790	0.5241	1.4315
O	3.5586	-1.9790	0.0290
H	3.4067	-2.2293	0.9629
H	4.5142	-2.0104	-0.0798
H	3.2675	-0.2516	0.6936
O	2.8296	-2.3147	2.6924
H	1.9415	-2.5911	2.4053
H	3.1958	-3.1019	3.1081
C	-1.6135	1.2782	-0.9026
H	-1.8633	2.0794	-1.6196
H	-1.4240	1.7421	0.0717
O	-2.6794	0.3510	-0.8145
C	-3.8384	0.7672	-0.2269
C	-4.8161	-0.2118	-0.0547
C	-4.0787	2.0747	0.1830
C	-6.0273	0.1184	0.5260
H	-4.6046	-1.2248	-0.3834
C	-5.3020	2.3891	0.7673
H	-3.3352	2.8516	0.0481
C	-6.2787	1.4224	0.9431
H	-6.7824	-0.6508	0.6565
H	-5.4847	3.4114	1.0846
H	-7.2290	1.6786	1.3998

Structure **H'·AuCl$_3$*5ext*_hy** ***(TS)***

C	2.5828	2.0158	-0.3126
O	1.2461	2.0829	-0.5865
C	0.9199	1.2791	-1.7427
C	2.1932	0.5287	-2.1006
C	3.2763	1.4216	-1.5152
H	0.6760	1.9945	-2.5472
H	2.2101	-0.4582	-1.6302
H	2.2874	0.3950	-3.1793
H	4.1949	0.8921	-1.2559
H	3.5235	2.2451	-2.1925
C	-0.3353	0.4883	-1.5194
H	-0.5844	-0.0373	-2.4464
Au	-0.0945	-1.1685	-0.1844
Cl	0.0466	-3.2118	1.3052
Cl	-0.2919	0.2084	1.7567
Cl	0.1220	-2.5240	-2.1078
N	3.0672	3.2135	0.2102
C	4.4994	3.2007	0.4777
H	4.8037	4.2030	0.7868
H	4.7661	2.4986	1.2815
H	5.0632	2.9430	-0.4204
C	2.3226	3.7083	1.3652
H	2.5152	3.1071	2.2660
H	2.6320	4.7357	1.5680
H	1.2513	3.7083	1.1619
O	2.7006	0.9116	0.8244
H	1.8145	0.7678	1.2224

O	3.5294	-1.3502	0.4105
H	3.3115	-1.7371	1.2969
H	4.4875	-1.4000	0.3114
H	3.1117	-0.0627	0.5761
O	2.5721	-2.0130	2.8097
H	1.7375	-2.4105	2.5010
H	3.0142	-2.7214	3.2890
C	-1.4973	1.3227	-1.0672
H	-1.6789	2.0951	-1.8343
H	-1.2895	1.8292	-0.1190
O	-2.6267	0.4767	-0.9452
C	-3.7303	0.9635	-0.3093
C	-4.7312	0.0318	-0.0371
C	-3.8969	2.2955	0.0558
C	-5.8925	0.4331	0.5983
H	-4.5764	-1.0019	-0.3318
C	-5.0701	2.6816	0.6970
H	-3.1340	3.0354	-0.1578
C	-6.0696	1.7621	0.9720
H	-6.6660	-0.2996	0.8072
H	-5.1958	3.7226	0.9793
H	-6.9802	2.0746	1.4725

Structure **H'·AuCl$_3$*5ext*_hy** ***(postTS)***

C	-2.5961	2.0381	0.2294
O	-1.2319	2.1379	0.5455
C	-0.9215	1.3324	1.6871
C	-2.2005	0.5912	2.0479
C	-3.2704	1.5109	1.4821
H	-0.6685	2.0295	2.5083
H	-2.2339	-0.3869	1.5590
H	-2.2856	0.4362	3.1252
H	-4.2103	1.0005	1.2604
H	-3.4689	2.3485	2.1582
C	0.3303	0.5311	1.4817
H	0.5848	0.0224	2.4165
Au	0.0892	-1.1650	0.1895
Cl	0.0276	-3.2902	-1.2351
Cl	0.2470	0.1466	-1.7948
Cl	-0.1130	-2.4691	2.1527
N	-3.0513	3.3234	-0.2196
C	-4.4881	3.3360	-0.4590
H	-4.7836	4.3404	-0.7731
H	-4.7935	2.6324	-1.2503
H	-5.0414	3.0991	0.4523
C	-2.3534	3.7626	-1.4237
H	-2.6162	3.1616	-2.3090
H	-2.6232	4.8011	-1.6320
H	-1.2721	3.7175	-1.2803
O	-2.7354	1.0313	-0.7886
H	-1.8867	0.9638	-1.2598
O	-3.5412	-1.4863	-0.5190
H	-3.1332	-1.9656	-1.5219
H	-4.5006	-1.5823	-0.4543
H	-3.3344	-0.5133	-0.5521
O	-2.6064	-2.4014	-2.5244
H	-1.7360	-2.7747	-2.2461
H	-3.1204	-3.1325	-2.8927
C	1.4916	1.3455	0.9968
H	1.6724	2.1444	1.7369
H	1.2819	1.8192	0.0324
O	2.6230	0.4983	0.9038
C	3.7375	0.9808	0.2840
C	4.7556	0.0540	0.0632
C	3.8992	2.3045	-0.1125
C	5.9289	0.4518	-0.5520
H	4.6045	-0.9731	0.3815
C	5.0851	2.6873	-0.7320
H	3.1227	3.0408	0.0599
C	6.1016	1.7726	-0.9559
H	6.7156	-0.2772	-0.7206
H	5.2069	3.7218	-1.0386
H	7.0220	2.0824	-1.4399

Structure **H'·AuCl$_3$*5ext*_hy$_{zw}$**

C	3.0078	-2.0039	-0.2930
O	2.0800	-1.2169	-1.0921
C	0.7720	-1.7768	-1.0758
C	0.9847	-3.2170	-0.6039
C	2.1247	-3.0606	0.3872
H	0.3822	-1.7374	-2.0998
H	0.0846	-3.6656	-0.1758
H	1.3010	-3.8250	-1.4586
H	1.7414	-2.7203	1.3562
H	2.7008	-3.9744	0.5441
C	-0.1708	-1.0300	-0.1502
H	0.0078	-1.2817	0.8999
Au	0.3031	1.0450	-0.1411
Cl	0.9287	3.4834	-0.1020
Cl	-0.2175	1.1491	-2.4483
Cl	0.7600	0.8554	2.1965
N	3.3798	-0.8672	0.9206
C	4.2902	-1.4691	1.9024
H	5.2083	-1.7606	1.3885
H	4.5247	-0.7464	2.6879
H	3.8274	-2.3502	2.3500
C	3.9579	0.3492	0.3348
H	4.3099	1.0128	1.1294
H	4.7987	0.0581	-0.2991
H	3.2026	0.8642	-0.2618
C	-1.6138	-1.2357	-0.4885
H	-1.8587	-0.8515	-1.4877
H	-1.8159	-2.3215	-0.4881
O	-2.4046	-0.5996	0.5019
C	-3.7601	-0.7167	0.4127
C	-4.4888	-0.0853	1.4204
C	-4.4212	-1.4107	-0.5957
C	-5.8702	-0.1510	1.4185
H	-3.9499	0.4496	2.1968
C	-5.8119	-1.4661	-0.5831
H	-3.8733	-1.9069	-1.3883
C	-6.5429	-0.8428	0.4152
H	-6.4279	0.3429	2.2083
H	-6.3222	-2.0089	-1.3731
H	-7.6268	-0.8930	0.4151
H	2.4994	-0.6154	1.3843
O	4.0653	-2.3277	-0.8869

Structure **H'·AuCl$_3$*5ext*_hy$_{zw}$** ***(TS)***

C	-3.0097	2.0593	-0.3682
O	-2.1059	1.2684	-1.1203
C	-0.7808	1.8006	-1.0719
C	-0.9744	3.2474	-0.6047
C	-2.1711	3.1282	0.3247
H	-0.3820	1.7539	-2.0911
H	-0.0860	3.6636	-0.1239
H	-1.2191	3.8690	-1.4724
H	-1.8510	2.8105	1.3217
H	-2.7510	4.0476	0.4213
C	0.1370	1.0365	-0.1375
H	-0.0598	1.2778	0.9122
Au	-0.3020	-1.0448	-0.1645

Cl	-0.9054	-3.4850	-0.1679
Cl	0.1712	-1.0948	-2.4842
Cl	-0.6430	-0.9304	2.1930
N	-3.3367	0.8163	1.0683
C	-4.2574	1.4316	2.0153
H	-5.1601	1.7370	1.4777
H	-4.5421	0.7329	2.8105
H	-3.8000	2.3141	2.4691
C	-3.8826	-0.4089	0.5002
H	-4.2032	-1.1049	1.2849
H	-4.7479	-0.1514	-0.1191
H	-3.1295	-0.8987	-0.1225
C	1.5834	1.2579	-0.4605
H	1.8333	0.8905	-1.4651
H	1.7822	2.3438	-0.4430
O	2.3748	0.6103	0.5207
C	3.7301	0.7254	0.4285
C	4.4606	0.0682	1.4183
C	4.3896	1.4406	-0.5660
C	5.8422	0.1294	1.4123
H	3.9228	-0.4835	2.1836
C	5.7805	1.4914	-0.5576
H	3.8405	1.9568	-1.3448
C	6.5132	0.8424	0.4228
H	6.4014	-0.3851	2.1879
H	6.2895	2.0509	-1.3366
H	7.5973	0.8889	0.4195
H	-2.4453	0.6094	1.5250
O	-4.1225	2.2555	-0.8639

Structure **H'·AuCl$_3$5*ext*_hy$_{zw}$** ***(postTS)***

C	2.9614	-2.3376	-0.6707
O	1.9486	-1.7512	-1.3444
C	0.6800	-2.1107	-0.7533
C	1.0173	-2.5642	0.6606
C	2.4122	-3.1277	0.4768
H	0.3074	-2.9761	-1.3291
H	1.0375	-1.7081	1.3397
H	0.2902	-3.2897	1.0311
H	3.0660	-3.0567	1.3471
H	2.3901	-4.1768	0.1538
C	-0.3283	-1.0156	-0.9664
Au	0.2966	0.8420	-0.1306
Cl	1.1877	3.0033	0.8067
Cl	0.9908	1.4799	-2.3035
Cl	-0.5637	0.3052	2.0189
N	3.3846	-0.0238	1.0891
C	4.1128	-0.6087	2.1946
H	4.8099	-1.3672	1.8150
H	4.7066	0.1233	2.7711
H	3.4212	-1.1029	2.8856
C	4.2461	0.7304	0.2022
H	4.7941	1.5460	0.7074
H	4.9912	0.0605	-0.2431
H	3.6586	1.1665	-0.6138
C	-1.6909	-1.4192	-0.4879
H	-1.9814	-2.3420	-1.0191
H	-1.7046	-1.6305	0.5879
H	-0.3695	-0.7524	-2.0274
O	-2.5968	-0.3754	-0.7988
C	-3.8662	-0.4669	-0.3109
C	-4.6717	0.6555	-0.5015
C	-4.3751	-1.5901	0.3336
C	-5.9790	0.6512	-0.0497
H	-4.2512	1.5199	-1.0066
C	-5.6919	-1.5768	0.7836
H	-3.7665	-2.4745	0.4830
C	-6.4989	-0.4660	0.5977
H	-6.5980	1.5301	-0.2022
H	-6.0842	-2.4566	1.2848
H	-7.5243	-0.4668	0.9524
H	2.6573	0.5943	1.4440
O	4.0990	-2.2505	-1.0560

Structure **H'·AuCl$_3$5*ext*_lac**

C	-2.3072	3.8394	-0.2393
O	-1.1408	3.1827	-0.0744
C	-1.3829	1.8157	0.3832
C	-2.8237	1.5443	-0.0215
C	-3.4489	2.9219	0.0728
H	-1.2694	1.8298	1.4741
H	-2.8503	1.1809	-1.0557
H	-3.2908	0.7965	0.6222
H	-4.2873	3.1061	-0.5995
H	-3.7765	3.1514	1.0947
C	-0.3358	0.9522	-0.2683
Au	-0.7985	-1.0967	0.0255
Cl	-1.3331	-3.5167	0.3392
Cl	-0.9416	-1.3185	-2.3252
Cl	-0.6476	-0.7793	2.3663
C	1.0546	1.2423	0.2111
H	1.2793	2.2967	-0.0196
H	1.1486	1.1069	1.2975
H	-0.4009	1.0557	-1.3561
O	1.9576	0.3940	-0.4779
C	3.2900	0.5404	-0.2306
C	3.8267	1.4968	0.6254
C	4.1305	-0.3463	-0.9033
C	5.2066	1.5569	0.7970
H	3.1903	2.1939	1.1581
C	5.4994	-0.2735	-0.7208
H	3.6874	-1.0847	-1.5649
C	6.0484	0.6803	0.1317
H	5.6199	2.3062	1.4655
H	6.1449	-0.9690	-1.2486
H	7.1226	0.7359	0.2739
O	-2.3294	4.9933	-0.5781

H'·AuCl$_2$5*ext*_lacI

C	-2.5767	2.3764	-0.1074
O	-1.4454	2.3663	0.6434
C	-1.4593	1.2828	1.5781
C	-2.9053	0.7933	1.6375
C	-3.4698	1.2468	0.3036
H	-1.1507	1.6964	2.5531
H	-2.9635	-0.2844	1.8009
H	-3.4147	1.2960	2.4618
H	-3.3787	0.4727	-0.4705
H	-4.5091	1.5753	0.3302
C	-0.3770	0.2900	1.2965
H	-0.3846	-0.5187	2.0322
Au	-0.8462	-0.9128	-0.4679
Cl	-0.2142	0.7022	-2.0506
Cl	-1.5008	-2.6916	0.9114
C	0.9842	0.8524	1.0747
H	1.0072	1.5789	0.2561
H	1.2221	1.3922	2.0114
O	1.8915	-0.2034	0.8615
C	3.1761	0.1234	0.5219
C	3.9925	-0.9456	0.1594
C	3.6785	1.4195	0.5346
C	5.3103	-0.7150	-0.1923

H	3.5742	-1.9475	0.1605
C	5.0058	1.6336	0.1749
H	3.0601	2.2617	0.8239
C	5.8256	0.5778	-0.1890
H	5.9407	-1.5528	-0.4743
H	5.3950	2.6471	0.1850
H	6.8589	0.7565	-0.4675
O	-2.7412	3.2054	-0.9583

Structure **H'·AuCl$_2$5*ext*_lacII**

C	2.4725	1.8844	0.9868
O	1.2508	1.3364	0.8329
C	0.3587	2.2122	0.1135
C	1.2417	3.3420	-0.4410
C	2.4654	3.2846	0.4560
H	-0.3698	2.6032	0.8308
H	1.5186	3.1447	-1.4814
H	0.7130	4.2944	-0.4076
H	3.4102	3.5116	-0.0390
H	2.3715	3.9487	1.3236
C	-0.3167	1.4270	-0.9697
Au	-1.3827	-0.1796	-0.1525
Cl	-2.4649	-2.2683	0.6540
Cl	-3.0847	1.2049	0.6057
C	0.5822	0.5631	-1.8286
H	0.2219	0.4721	-2.8528
H	1.6283	0.8810	-1.8328
H	-1.0360	2.0305	-1.5257
O	0.4759	-0.7778	-1.2313
C	1.6163	-1.3311	-0.6412
C	2.8116	-1.3281	-1.3424
C	1.5087	-1.9127	0.6087
C	3.9270	-1.9135	-0.7576
H	2.8735	-0.8907	-2.3337
C	2.6288	-2.5029	1.1753
H	0.5553	-1.8853	1.1314
C	3.8411	-2.4992	0.4980
H	4.8677	-1.9158	-1.2985
H	2.5521	-2.9560	2.1582
H	4.7179	-2.9529	0.9481
O	3.3649	1.2701	1.5065

Structure **H'·AuCl$_2$5*ext*_lacII** ***(TS)***

C	-2.6618	1.7926	-0.8295
O	-1.4249	1.2910	-0.6374
C	-0.5295	2.2902	-0.1034
C	-1.4458	3.4011	0.4131
C	-2.6678	3.2474	-0.4742
H	0.0973	2.6471	-0.9280
H	-1.7114	3.2137	1.4594
H	-0.9667	4.3781	0.3481
H	-3.6151	3.5320	-0.0159
H	-2.5625	3.8039	-1.4140
C	0.3149	1.6454	0.9579
Au	1.3312	-0.1098	0.1661
Cl	2.4119	-2.1502	-0.6191
Cl	2.9642	1.2380	-0.8455
C	-0.3235	0.8867	1.9571
H	0.2059	0.6299	2.8680
H	-1.3987	0.7348	1.9433
H	1.1868	2.2185	1.2783
O	-0.1718	-1.1729	1.2275
C	-1.2874	-1.5749	0.5947
C	-2.4617	-1.6742	1.3553
C	-1.3034	-1.9606	-0.7514
C	-3.6067	-2.2063	0.7910

H	-2.4425	-1.3584	2.3944
C	-2.4591	-2.4799	-1.3066
H	-0.4051	-1.8369	-1.3502
C	-3.6131	-2.6084	-0.5413
H	-4.5054	-2.3013	1.3922
H	-2.4623	-2.7792	-2.3498
H	-4.5183	-3.0101	-0.9845
O	-3.5522	1.1011	-1.2451

Structure **H'·AuCl$_2$5*ext*_lacβ$_{elim}$**

C	0.3298	2.9585	0.2389
O	1.2249	2.7588	-0.7615
C	2.3636	2.0294	-0.2989
C	2.2962	2.0567	1.2357
C	0.8268	2.3287	1.5028
H	3.2601	2.5549	-0.6569
H	2.6564	1.1226	1.6716
H	2.9161	2.8741	1.6086
H	0.2491	1.4073	1.6512
H	0.6333	2.9869	2.3509
C	2.4524	0.6754	-0.8963
H	3.3120	0.1054	-0.5447
Au	0.6038	-0.7674	-0.1320
Cl	-0.8160	-1.8514	1.4384
Cl	2.3640	-2.3322	0.3401
C	1.6888	0.1621	-1.9114
H	0.9145	0.7592	-2.3813
H	2.0148	-0.7416	-2.4195
O	-0.8909	0.6076	-0.6705
C	-2.1614	0.3285	-0.4354
C	-2.8305	0.9460	0.6371
C	-2.8732	-0.5353	-1.2879
C	-4.1849	0.7381	0.8161
H	-2.2639	1.5963	1.2975
C	-4.2226	-0.7454	-1.0906
H	-2.3328	-1.0276	-2.0921
C	-4.8836	-0.1086	-0.0408
H	-4.7039	1.2290	1.6333
H	-4.7708	-1.4084	-1.7525
H	-5.9448	-0.2771	0.1113
O	-0.6793	3.5790	0.0449

Structure **H'·AuCl$_3$6*ent*** ***(TS)***

C	-4.0503	0.2258	-0.1825
O	-3.2767	1.1989	-0.2203
C	-1.2373	-0.5785	0.2754
C	-2.0776	-1.2717	-0.7539
C	-3.5439	-1.1874	-0.3458
H	-1.3812	-0.9261	1.3010
H	-1.8027	-2.3284	-0.8246
H	-1.9289	-0.8195	-1.7387
H	-3.7049	-1.7252	0.5981
H	-4.1505	-1.7036	-1.0971
C	-0.9824	0.7917	0.1315
H	-0.9163	1.2123	-0.8715
Au	0.9265	-0.9720	-0.0314
Cl	0.8249	-0.4531	-2.3357
Cl	1.0456	-1.4817	2.2728
Cl	3.1602	-1.8237	-0.3303
N	-5.3683	0.3857	0.0129
C	-6.3228	-0.7117	-0.0029
H	-6.6614	-0.9349	-1.0213
H	-7.1909	-0.4178	0.5907
H	-5.9076	-1.6152	0.4423
C	-5.9577	1.7130	0.0672
H	-6.4872	1.8511	1.0148

H	-6.6766	1.8324	-0.7503
H	-5.1834	2.4709	-0.0252
C	-0.7258	1.7030	1.2647
H	-0.2727	1.1710	2.1118
H	-1.7128	2.0432	1.6060
O	-0.0078	2.8555	0.8817
C	1.2941	2.6381	0.4623
C	1.6594	3.1514	-0.7737
C	2.2174	1.9689	1.2560
C	2.9640	2.9935	-1.2207
H	0.9134	3.6682	-1.3698
C	3.5158	1.8054	0.7930
H	1.9283	1.5847	2.2294
C	3.8933	2.3139	-0.4433
H	3.2510	3.3951	-2.1876
H	4.2372	1.2760	1.4084
H	4.9105	2.1835	-0.7984

Structure **H'·AuCl$_3$*6ent***

C	2.8904	1.3564	0.2578
O	1.6784	1.8000	0.4432
C	1.0282	-0.2249	1.6454
C	2.4775	-0.6550	1.7448
C	3.4725	0.3846	1.2100
H	0.4538	-0.5592	2.5104
H	2.6399	-1.6118	1.2448
H	2.7003	-0.8249	2.8040
H	4.3423	-0.1029	0.7728
H	3.8410	1.0033	2.0425
C	0.9024	1.2708	1.5506
H	1.3380	1.6832	2.4755
Au	-0.0169	-1.2266	0.0870
Cl	-1.7808	-1.5768	1.6450
Cl	1.7874	-0.8980	-1.4337
Cl	-1.2880	-2.4569	-1.6940
N	3.5435	1.8884	-0.7377
C	4.8823	1.4234	-1.1062
H	5.2840	2.1078	-1.8506
H	4.8346	0.4177	-1.5341
H	5.5471	1.4278	-0.2421
C	2.8390	2.7978	-1.6455
H	2.0722	2.2511	-2.2025
H	3.5620	3.2200	-2.3393
H	2.3651	3.6036	-1.0844
C	-0.4783	1.8492	1.4144
H	-0.4349	2.9414	1.5100
H	-1.0912	1.4481	2.2327
O	-1.0008	1.4861	0.1527
C	-2.3277	1.6820	-0.0874
C	-2.8032	1.1543	-1.2866
C	-3.1861	2.3534	0.7751
C	-4.1380	1.2973	-1.6188
H	-2.1092	0.6226	-1.9328
C	-4.5265	2.4888	0.4250
H	-2.8289	2.7748	1.7080
C	-5.0100	1.9656	-0.7634
H	-4.5033	0.8795	-2.5521
H	-5.1953	3.0140	1.1002
H	-6.0574	2.0753	-1.0247

Structure **H'·AuCl$_3$*6ent*_hy$_{zw}$**

C	-3.7165	0.2647	-0.2079
O	-2.6531	1.2544	0.0296
C	-1.0767	-0.4132	0.5502
C	-2.1289	-1.4914	0.4946
C	-3.4940	-0.8629	0.7972
H	-0.9648	-0.0363	1.5735
H	-1.9129	-2.2816	1.2210
H	-2.1651	-1.9493	-0.4992
H	-3.5175	-0.4822	1.8264
H	-4.2762	-1.6202	0.6849
C	-1.3825	0.7595	-0.3667
H	-1.4128	0.4454	-1.4200
Au	0.8402	-1.2259	0.1625
Cl	0.1116	-1.9110	-1.9966
Cl	1.5065	-0.4696	2.3154
Cl	3.1293	-2.2323	-0.2454
N	-4.9532	1.1943	0.3982
C	-6.1743	0.3881	0.5647
H	-6.3721	-0.1268	-0.3783
H	-7.0142	1.0413	0.8071
H	-6.0399	-0.3382	1.3652
C	-5.2043	2.3435	-0.4888
H	-4.3103	2.9611	-0.5491
H	-6.0380	2.9284	-0.0967
H	-5.4555	1.9584	-1.4782
C	-0.4272	1.9198	-0.2079
H	-0.2531	2.1099	0.8611
H	-0.8620	2.8218	-0.6574
O	0.7825	1.5746	-0.8601
C	1.9208	2.2466	-0.5390
C	3.1065	1.6835	-1.0098
C	1.9501	3.4225	0.2026
C	4.3167	2.2924	-0.7307
H	3.0540	0.7601	-1.5812
C	3.1761	4.0217	0.4759
H	1.0342	3.8787	0.5616
C	4.3601	3.4658	0.0177
H	5.2359	1.8445	-1.0960
H	3.1950	4.9392	1.0564
H	5.3105	3.9403	0.2389
H	-4.6353	1.5326	1.3094
O	-3.9253	-0.0372	-1.4117

Structure **H'·AuCl$_3$*6ent*_hy$_{zw}$** ***(TS)***

C	-3.6454	0.0711	-0.4382
O	-2.6945	1.0949	-0.2493
C	-1.0615	-0.4942	0.4118
C	-2.0618	-1.6188	0.3109
C	-3.4619	-1.0311	0.5650
H	-1.0460	-0.0878	1.4295
H	-1.8512	-2.4006	1.0470
H	-2.0383	-2.0753	-0.6848
H	-3.5180	-0.6398	1.5853
H	-4.2317	-1.7945	0.4244
C	-1.3647	0.6367	-0.5537
H	-1.3307	0.2913	-1.5982
Au	0.9128	-1.2132	0.1899
Cl	0.3814	-2.0437	-1.9715
Cl	1.3882	-0.3142	2.3357
Cl	3.2560	-2.1171	0.0016
N	-5.1504	1.1873	0.6185
C	-6.3218	0.3368	0.7245
H	-6.5477	-0.0815	-0.2640
H	-7.2081	0.8866	1.0712
H	-6.1319	-0.4876	1.4160
C	-5.4047	2.3775	-0.1706
H	-4.5438	3.0477	-0.1320
H	-6.2942	2.9235	0.1729
H	-5.5690	2.0819	-1.2140
C	-0.4838	1.8524	-0.3831
H	-0.3718	2.0778	0.6871

H	-0.9476	2.7157	-0.8770
O	0.7691	1.5551	-0.9704
C	1.8578	2.2852	-0.6040
C	3.0922	1.7602	-0.9835
C	1.7906	3.4842	0.0965
C	4.2560	2.4322	-0.6551
H	3.1136	0.8178	-1.5251
C	2.9707	4.1470	0.4202
H	0.8353	3.9099	0.3832
C	4.2029	3.6300	0.0526
H	5.2142	2.0149	-0.9496
H	2.9151	5.0832	0.9676
H	5.1169	4.1543	0.3119
H	-4.7930	1.4301	1.5390
O	-4.1313	-0.0840	-1.5430

Structure **H'·$AuCl_3$*6ent*_hy$_{zw}$** ***(postTS)***

C	2.9778	-0.0076	-1.0371
O	2.0422	0.9022	-0.7248
C	1.0922	-0.4467	1.0985
C	2.2750	-1.3794	1.0430
C	3.1324	-1.2474	-0.2092
H	0.7457	-0.3361	2.1276
H	1.9696	-2.4190	1.1926
H	2.8806	-1.1192	1.9247
H	2.9300	-2.0615	-0.9118
H	4.1958	-1.3230	0.0375
C	1.4522	0.9179	0.5813
H	2.2274	1.2902	1.2763
Au	-0.6923	-1.2475	0.2407
Cl	-1.7765	-0.6741	2.2861
Cl	0.3533	-1.7821	-1.8250
Cl	-2.8681	-2.2186	-0.6324
N	4.7157	1.4742	0.7058
C	5.9741	0.8764	0.3001
H	6.8384	1.5529	0.4184
H	5.9225	0.5926	-0.7580
H	6.1711	-0.0297	0.8811
C	4.4892	2.7399	0.0330
H	4.3699	2.5681	-1.0435
H	5.3170	3.4581	0.1638
H	3.5702	3.2076	0.4008
C	0.3581	1.9498	0.5679
H	0.7781	2.9357	0.3283
H	-0.0860	1.9915	1.5716
O	-0.6068	1.5813	-0.3995
C	-1.8170	2.2028	-0.3937
C	-2.7710	1.6631	-1.2556
C	-2.1284	3.3027	0.3978
C	-4.0341	2.2224	-1.3214
H	-2.5000	0.7975	-1.8547
C	-3.4043	3.8537	0.3186
H	-1.3945	3.7391	1.0660
C	-4.3600	3.3226	-0.5327
H	-4.7729	1.7940	-1.9920
H	-3.6444	4.7136	0.9368
H	-5.3519	3.7596	-0.5846
H	4.7365	1.6394	1.7080
O	3.6035	0.1580	-2.0609

Structure **H'·$AuCl_3$*6ent*_lac**

C	2.8895	2.0580	-0.7311
O	1.5836	2.2873	-0.4996
C	1.4621	0.5756	1.2512
C	2.9689	0.3950	1.2139
C	3.6736	1.4874	0.4051
H	1.0588	0.4175	2.2525
H	3.2418	-0.5884	0.8250
H	3.3400	0.4230	2.2444
H	4.6340	1.1502	0.0126
H	3.8783	2.3460	1.0612
C	1.0442	1.9477	0.7873
H	1.4677	2.6480	1.5293
Au	0.4505	-0.9858	0.2082
Cl	-0.9724	-1.1978	2.1094
Cl	1.9009	-0.7575	-1.6663
Cl	-0.7499	-2.8855	-0.9449
C	-0.4328	2.2302	0.7418
H	-0.5996	3.2978	0.5497
H	-0.8624	1.9771	1.7205
O	-1.0150	1.4515	-0.2860
C	-2.3711	1.3466	-0.3384
C	-2.8665	0.4090	-1.2437
C	-3.2431	2.1029	0.4364
C	-4.2319	0.2290	-1.3696
H	-2.1592	-0.1770	-1.8255
C	-4.6145	1.9097	0.2964
H	-2.8729	2.8416	1.1384
C	-5.1165	0.9785	-0.5985
H	-4.6104	-0.5067	-2.0728
H	-5.2930	2.5032	0.9018
H	-6.1875	0.8348	-0.6975
O	3.3519	2.3845	-1.7966

Structure **H'·$AuCl_2$*6ent*_lacII**

C	3.8088	2.0318	-0.0068
O	2.5658	2.3344	-0.4407
C	1.7023	0.1879	0.3625
C	3.0646	-0.4141	0.2285
C	4.0651	0.6813	0.5869
H	1.4625	0.4864	1.3941
H	3.2109	-1.2644	0.8958
H	3.2258	-0.7555	-0.8011
H	4.0548	0.8506	1.6725
H	5.0880	0.3984	0.3269
C	1.5437	1.3370	-0.6093
H	1.6125	0.9659	-1.6407
Au	-0.0034	-0.9599	-0.0698
Cl	1.0572	-2.9566	0.4498
Cl	-2.1690	-2.0574	-0.5980
C	0.2334	2.0462	-0.3942
H	0.1831	2.4507	0.6238
H	0.0473	2.8357	-1.1266
O	-0.7744	1.0240	-0.5645
C	-2.0534	1.3753	-0.0998
C	-3.0039	1.7162	-1.0404
C	-2.3173	1.3409	1.2566
C	-4.2805	2.0372	-0.5969
H	-2.7437	1.7176	-2.0938
C	-3.5965	1.6633	1.6849
H	-1.5326	1.0517	1.9514
C	-4.5752	2.0095	0.7597
H	-5.0471	2.3038	-1.3169
H	-3.8313	1.6379	2.7438
H	-5.5759	2.2555	1.0995
O	4.6424	2.8999	-0.0551

3. References

1. Lopp, J. M.; Schmidt, V. A. Intermolecular Phosphite-Mediated Radical Desulfurative Alkene Alkylation Using Thiols. *Org. Lett.* **2019***, 21*, 8031–8036.
2. Jankins, C. T.; Martin-Montero, R.; Cooper, P.; Martin, R.; K. M. Low-Valent Tungsten Catalysis Enables Site-Selective Isomerization–Hydroboration of Unactivated Alkenes. *J. Am. Chem. Soc.* **2021**, *143*, 14981–14986.
3. Veronica Tona, V.; Torre, A.; Padmanaban, M.; Ruider, S.; González, L.; Maulide, N. Chemo- and Stereoselective Transition-Metal-Free Amination of Amides with Azides. *J. Am. Chem. Soc.* **2016**, *138*, 8348–8351.
4. Zhu, M.; Wang, L.; He, J. Repurposing the 3-Isocyanobutanoic Acid Adenylation Enzyme SfaB for Versatile Amidation and Thioesterification. *Angew. Chem. Int. Ed.* **2021**, *60* (4), 2030–2035.
5. Rodrigalvarez, J.; Wang, H.; Martin, R. Native Amides as Enabling Vehicles for Forging sp3–sp3 Architectures via Interrupted Deaminative Ni-Catalyzed Chain-Walking. *J. Am. Chem. Soc.* **2023**, *145*, 3869–3874.
6. Psotta, K.; Wiechers, A. The Total Synthesis of Joubertinamine. *Tetrahedron* **1979**, *35*, 255–257.
7. Oliveira, B. L.; Stenton, B. J.; Unnikrishnan, V.; de Almeida, C. t. R.; Conde, J.; Negrão, M.; Schneider, F. S.; Cordeiro, C.; Ferreira, M. G.; Caramori, G. F.; Domingos, J. B.; Fior, R.; Bernardes, G. J. L. Platinum-triggered Bond Cleavage of Pentynoyl Amide and N-Propargyl Handles for Drug Activation. *J. Am. Chem. Soc.,* **2020**, *142*, 10869– 10880.
8. Gaussian 16, Revision C.01, Frisch, M. J.; Trucks, G. W.; Schlegel, H. B.; Scuseria, G. E.; Robb, M. A.; Cheeseman, J. R.; Scalmani, G.; Barone, V.;

Petersson, G. A.; Nakatsuji, H.; Li, X.; Caricato, M.; Marenich, A. V.; Bloino, J.; Janesko, B. G.; Gomperts, R.; Mennucci, B.; Hratchian, H. P.; Ortiz, J. V.; Izmaylov, A. F.; Sonnenberg, J. L.; Williams-Young, D.; Ding, F.; Lipparini, F.; Egidi, F.; Goings, J.; Peng, B.; Petrone, A.; Henderson, T.; Ranasinghe, D.; Zakrzewski, V. G.; Gao, J.; Rega, N.; Zheng, G.; Liang, W.; Hada, M.; Ehara, M.; Toyota, K.; Fukuda, R.; Hasegawa, J.; Ishida, M.; Nakajima, T.; Honda, Y.; Kitao, O.; Nakai, H.; Vreven, T.; Throssell, K.; Montgomery, J. A., Jr.; Peralta, J. E.; Ogliaro, F.; Bearpark, M. J.; Heyd, J. J.; Brothers, E. N.; Kudin, K. N.; Staroverov, V. N.; Keith, T. A.; Kobayashi, R.; Normand, J.; Raghavachari, K.; Rendell, A. P.; Burant, J. C.; Iyengar, S. S.; Tomasi, J.; Cossi, M.; Millam, J. M.; Klene, M.; Adamo, C.; Cammi, R.; Ochterski, J. W.; Martin, R. L.; Morokuma, K.; Farkas, O.; Foresman, J. B.; Fox, D. J. Gaussian, Inc., Wallingford CT, 2016.

9. Zhao, Y.; Truhlar, D. G. The M06 Suite of Density Functionals for Main Group Thermochemistry, Thermochemical Kinetics, Noncovalent Interactions, Excited States, and Transition Elements: Two New Functionals and Systematic Testing of Four M06-Class Functionals and 12 Other Functionals. *Theor. Chem. Acc.* **2008**, *120*, 215–241.
10. Fuentealba, P.; Preuss, H.; Stoll, H.; Von Szentpály, L. A Proper Account of Core-polarization with Pseudopotentials – Single Valence-Electron Alkali Compounds. *Chem. Phys. Lett.* **1982**, *89*, 418–422.
11. T. H. Dunning, and P. J. Hay, in Modern Theoretical Chemistry: Methods of Electronic Structure Theory, ed. H. F. Schaefe, Plenum, New York, 1977, vol. 3, pp. 1–28.

12. Marenich, A. V.; Cramer, C. J.; Truhlar, D. G. Universal solvation model based on solute electron density and a continuum model of the solvent defined by the bulk dielectric constant and atomic surface tensions. *J. Phys. Chem. B*, **2009**, *113*, 6378-6396.
13. Ribeiro, R. F.; Marenich, A. V.; Cramer, C. J. &; Truhlar, D. G. Use of Solution-Phase Vibrational Frequencies in Continuum Models for the Free Energy of Solvation. *J. Phys. Chem. B* **2011**, *115*, 14556–14562.
14. Hratchian, H. P.; Schlegel, H. B. Accurate reaction paths using a Hessian based predictor-corrector integrator. *J. Chem. Phys.* **2004**, *120*, 9918–9924.
15. Hratchian, H. P.; Schlegel, H. B. Using Hessian updating to increase the efficiency of a Hessian based predictor-corrector reaction path following method. *J. Chem. Theory Comput.* **2005**, *1*, 61–69.
16. Controlled in-cell generation of active palladium(0) species for bioorthogonal decaging. Konč, J.; Sabatino, V.; Jiménez-Moreno, E.; Latocheski, E.; Rodríguez Pérez, L.; Day, J.; Domingos, J. B.; Bernardes, G. J. L**.** *Angew. Chem. Int. Ed.* **2022**, *61*,e2021135.
17. Fior, R.; Póvoa, V.; Mendes, R. V.; Carvalho, T.; Gomes, A.; Figueiredo, N.; Ferreira, M. G. Single-cell functional and chemosensitive profiling of combinatorial colorectal therapy in zebrafish xenografts. *Proc. Natl. Acad. Sci. U S A* **2017**, *114*, E8234.
18. Allyson M. Freedy, A. M.; Matos, M. J.; Omar Boutureira, O.; Francisco Corzana, F.; Ana Guerreiro, A.; Padma Akkapeddi, P.; Víctor J. Somovilla, V.J.; Tiago Rodrigues, T.; Karl Nicholls, K.; Bangwen Xie, B.; Gonzalo Jiménez-

Osés, G.; Brindle, K. Neves, A. M.; Bernardes, G. J. L. *J. Am. Chem. Soc.* **2017**, *139* (50), 18365-18375.
